# Copper-catalyzed methylative difunctionalization of alkenes

**DOI:** 10.1038/s41467-018-06246-6

**Published:** 2018-09-13

**Authors:** Xu Bao, Takayuki Yokoe, Tu M. Ha, Qian Wang, Jieping Zhu

**Affiliations:** Laboratory of Synthesis and Natural Products, Institute of Chemical Sciences and Engineering, Ecole Polytechnique Fédérale de Lausanne, EPFL-SB-ISIC-LSPN, BCH5304, Lausanne, CH-1015 Switzerland

## Abstract

Trifluoromethylative difunctionalization and hydrofunctionalization of unactivated alkenes have been developed into powerful synthetic methodologies. On the other hand, methylative difunctionalization of olefins remains an unexplored research field. We report in this paper the Cu-catalyzed alkoxy methylation, azido methylation of alkenes using dicumyl peroxide (DCP), and di-*tert*-butyl peroxide (DTBP) as methyl sources. Using functionalized alkenes bearing a tethered nucleophile (alcohol, carboxylic acid, and sulfonamide), methylative cycloetherification, lactonization, and cycloamination processes are subsequently developed for the construction of important heterocycles such as 2,2-disubstituted tetrahydrofurans, tetrahydropyrans, γ-lactones, and pyrrolidines with concurrent generation of a quaternary carbon center. The results of control experiments suggest that the 1,2-alkoxy methylation of alkenes goes through a radical-cation crossover mechanism, whereas the 1,2-azido methylation proceeds via a radical addition and Cu-mediated azide transfer process.

## Abstract (web summary)

While the trifluoromethylative difunctionalization of unactivated alkenes has been largely explored, methylative difunctionalization remains underinvestigated. Here, the authors report copper-catalyzed alkoxy- and azido-methylation reactions of alkenes leading to important synthetic building blocks and valuable O- and N-heterocycles.

## Introduction

The so-called magic methyl effect has long been known in medicinal chemistry and has been frequently used to optimize the biological and pharmacological properties of a drug candidate^1^. In addition to traditional nucleophilic substitution reaction, transition metal-catalyzed cross-coupling reaction has recently been developed into a powerful tool for the methylation of (hetero)aromatics^2,3^. However, in comparison to the recent advances in the field of trifluoromethylation of organic compounds^4,5^, progress on the development of new methylation protocols has been much slower. While the importance of the CF_3_ group in pharmaceuticals and crop science is undeniable, the CH_3_ group deserved certainly equal attention. In fact, it was estimated that over 67% of 200 top-selling drugs bore at least one CH_3_ group, while <5% of the small molecule drugs in the same list contained a CF_3_ group^6^.

**Fig. 1:**
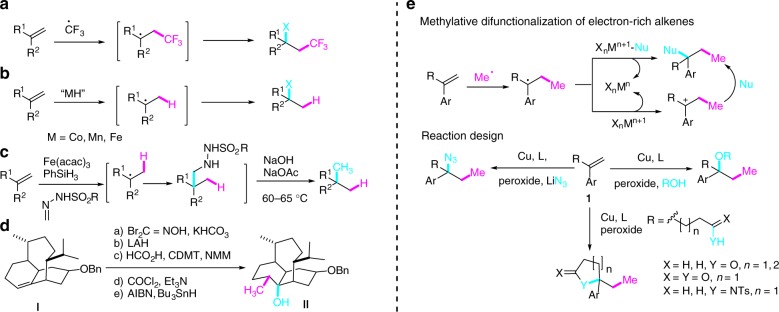
Functionalization of unactivated alkenes. **a** Trifluoromethylative difunctionalization of alkenes; **b** hydrofunctionalization of alkenes. **c** hydromethylation of alkenes; **d** Example of 1,2-hydroxy methylation of alkene in natural product synthesis. Five steps were required to accomplish this transformation; **e** methylative difunctionalization of electron-rich alkenes: radical-metal mediated ligand transfer and radical-cation crossover processes. Abbreviations: Fe(acac)_3_ iron (III) acetylacetonate; LAH lithium aluminum hydride; CDMT 2-chloro-4,6-dimethoxy-1,3,5-triazine; NMM N-methyl morpholine; AIBN azobisisobutyronitrile

Most of the trifluoromethylative difunctionalization of alkenes involved the generation of electrophilic CF_3_ radical from the hypervalent iodine reagents^7^ followed by its addition to the electron-rich alkenes (Fig. 1a)^3^. Similarly, metal-catalyzed hydrofunctionalization of alkenes, pioneered by Mukaiyama in 1980s^8^, has been extensively investigated (Fig. 1b)^9–16^. By choosing an appropriate radical acceptor, Baran and co-workers developed a protocol for the hydromethylation of unactivated alkenes for the one-pot conversion of alkenes to alkanes (Fig. 1c)^15^. Interestingly, in spite of the known magic methyl effect in medicinal chemistry and its utility in natural product synthesis, the related methylative difunctionalization of unactivated olefins was, to the best of our knowledge, far less developed and a multistep sequence was generally needed to accomplish this endeavor. As illustrated in Fig. 1d, five steps were needed to convert alkene **I** to hydroxymethylated derivative **II**, an advanced intermediate on the way to vinigrol ^17,18^.

Peroxides undergo homolytic cleavage of the O–O bond to generate acyloxy or alkoxy radicals, which can act as oxidants and radical initiators. These oxygen-centered radicals can also undergo further fragmentation to produce the alkyl radicals^19^. The groups of Kawazoe^20^ and Wong^21^ demonstrated in 1970s that the methyl radical generated from *tert*-butyl hydroperoxide and *tert*-butyl peracetate can methylate the protonated nucleobases via homolytic aromatic substitution (HAS) reaction. These pioneering studies, akin to Minisci reaction^22^, is in line with the nucleophilic nature of the methyl radical. Since then, conditions allowing the methylation of (hetero)arenes^23–26^, amides/carboxylic acids^27–30^, and isocyanides^31–34^ have been exploited. In addition, methylation of electron-deficient olefins such as N-arylacrylamides have also been developed^35–38^. In this latter case, the resulting electrophilic radical adduct underwent rapid intramolecular HAS with the tethered aromatic ring to afford 2,2-disubstituted oxindoles. On the other hand, methylative difunctionalization of unactivated double bonds using peroxide as methyl source has, to the best of our knowledge, not been reported^39^. This was probably due to the perception that methyl radical is nucleophilic, therefore, its addition to electron-rich alkenes would be polarity mismatched process^40–43]^. We report herein the realization of this endeavor by developing three-component 1,2-alkoxy methylation, 1,2-azido methylation, and methylative cycloetherification, lactonization, cycloamination of unactivated alkenes (Fig. 1e). The results of control experiments suggested that the 1,2-alkoxy methylation of alkenes went through a radical-cation crossover mechanism, whereas the azido methylation proceeded via a radical addition and Cu-mediated redox azide transfer process.

## Results

### Three-component 1,2-alkoxy methylation of alkenes

Examples of alkoxy alkylation of unactivated alkenes are rare. Wang and co-workers reported a rhenium-catalyzed 1,2-acetoxy methylation of styrene derivatives using phenyliodine diacetate (PIDA) as both the methyl and the acetoxy sources^44^, while Glorius^45^ and Bao^46,47^ reported the alkoxy alkylation of alkenes via decarboxylative generation of alkyl radicals.

We began our studies by examining the 1,2-alkoxy methylation of α-methylstyrene (**1a**). After extensive survey of the reaction parameters varying the Cu sources, the ligands, the Cu/ligand ratio, the peroxides, the bases, the solvents, the concentration, and the reaction temperature, the optimum conditions found consisted of heating a MeOH solution of **1a** (*c* 0.1 M) in a sealed tube in the presence of a catalytic amount of Cu(BF_4_)_2_•6H_2_O (0.2 equiv), 4,4-dimethoxy-2,2’-bipyridine (**L1**, 0.3 equiv) and Na_2_HPO_4_ (0.2 equiv) at 120 °C for 4 h. Under these conditions, **2a** was isolated in 96% yield. We note that reaction using 2-hydroperoxy-2-methylbutane as ethyl donor under otherwise standard conditions provided a complex reaction mixture.

**Fig. 2:**
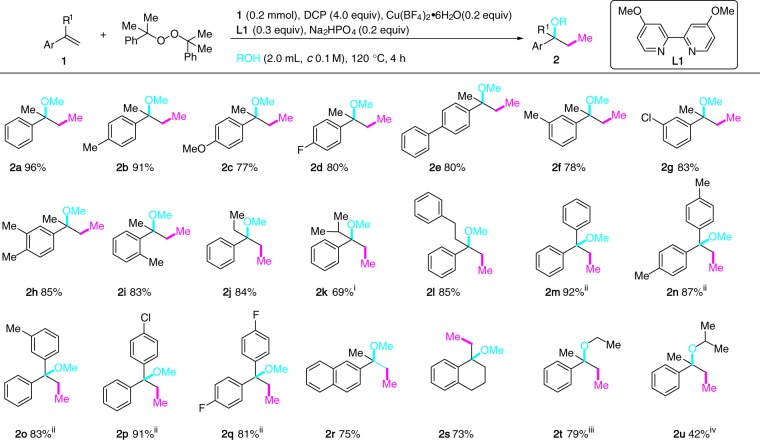
1,2-Alkoxy methylation of unactivated alkenes. Unless specified, MeOH was used as solvent. (i) 140 °C; (ii) DTBP (4.0 equiv) was used instead of DCP; (iii) EtOH (2.0 mL, *c* 0.1 M); (iv) *i*PrOH (2.0 mL, *c* 0.1 M). Abbreviations: DTBP di-*tert*-butyl peroxide; DCP dicumyl peroxide

The scope of this 1,2-alkoxy methylation of alkenes is shown in Fig. 2. Electron-donating (Me and OMe) and electron-withdrawing (F and Cl) substituents at different positions of the phenyl ring of the α-methylstyrene derivatives were transformed into the corresponding methylated ethers (**2b**–**2i**) in excellent yields. Different alkyl groups (Et, *i*Pr, and CH_2_CH_2_Ph) at the α-position of styrene were compatible (**2j**–**2l**) and the 1,1-diarylethylenes were similarly difunctionalized to afford the desired products (**2m**–**2q**) regardless of the electronic nature of the substituents on the aromatic ring. 2-Vinylnaphthalene and 1-methylene-1,2,3,4-tetrahydronaphthalene took part in the reaction to afford the three-component adducts without event (**2r**, **2s**). However, styrene failed to give the desired 1,2-methoxy methylation product under standard conditions. Performing the reaction of **1a** in ethanol and isopropanol under otherwise standard conditions afforded the ethyl ether (**2t**) and the isopropyl ether (**2u**), respectively. A gram scale experiment converted **1a** to the three-component adduct **2a** in 93% isolated yield.

### Three-component 1,2-azido methylation of alkenes

**Fig. 3:**
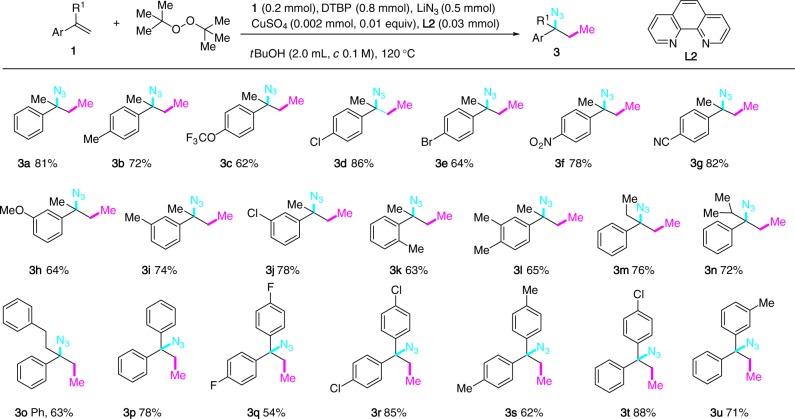
1,2-Azido methylation of unactivated alkenes. The reaction scheme is shown above the table

There were only few examples on the three-component carboazidation of alkenes with the concurrent formation of a C(sp^3^)-C(sp^3^) and a C(sp^3^)-N bond^48^. Renaud and co-workers developed a carboazidation of alkenes **1** employing electrophilic alkyl radicals generated from ethyl α-iodoacetate and phenylsulfonyl azide as the azide sources^49,50^. Three-component 1,2-azido alkylation of alkenes with nucleophilic alkyl radical is to the best of our knowledge unknown^51–53^. The α-methylstyrene (**1a**) was chosen as a test substrate for the optimization of the 1,2-azido methylation process. Using Cu(BF_4_)_2_•6H_2_O as catalyst (0.2 equiv), initial survey of the reaction conditions varying the methyl sources (DCP, DTBP, and *tert*-butyl peroxybenzoate), the azide sources (TMSN_3_, NaN_3_, KN_3_, and LiN_3_) and solvents (MeCN, *t*BuCN, DMF, DMSO, 1,4-dioxane, and *t*BuOH) prompted us to fix the following key parameters [LiN_3_, DTBP, *t*BuOH (*c* 0.1 M)] for further optimization. The 1,10-phenanthroline **L2** turned out to be a superior ligand than **L1** and CuSO_4_ stood out as the catalyst of choice among those copper salts screened [Cu(OAc)_2_, Cu(OTf)_2_, CuF_2_, and CuSO_4_]. Interestingly, reducing the loading of CuSO_4_ (0.01 equiv) gave a cleaner reaction mixture. Overall, under optimized conditions [CuSO_4_ (0.01 equiv), **L2** (0.03 equiv), LiN_3_ (2.5 equiv), DTBP (4.0 equiv), *t*BuOH (*c* 0.1 M)], the desired compound **3a** was isolated in 81% yield (Fig. 3). A similar yield of **3a** (79%) was obtained when the 1,2-azido methylation of **1a** was performed at 2.0 mmol scale. Once again, using 2-hydroperoxy-2-methylbutane as ethyl donor under otherwise standard conditions provided a complex reaction mixture.

The reaction was applicable to a variety of α-methylstyrene derivatives bearing electron-donating (Me and OMe) and -withdrawing groups (4-Cl, 4-Br, 4-F, 4-OCF_3_, 4-CN, and 4-NO_2_) on the phenyl ring. The presence of an o-methyl substituent in the substrate was also tolerated to afford **3k**. α-Ethyl, α-isopropyl and α-phenethyl styrenes participated in the reaction without event (**3m**–**3o**). The 1,1-diarylethylenes bearing substituents with different electronic properties were similarly converted to the three-component adducts (**3p**–**3u**).

### Methylative cycloetherification

Metal-catalyzed arylative cycloetherification and cycloamination has been well developed for the synthesis of functionalized oxa- and aza-heterocycles^54,55^. The alkylation-induced heterocyclization is, on the other hand, poorly documented. For instance, only few examples of alkylative cycloetherification have been reported in the literature ^56^.

**Fig. 4:**
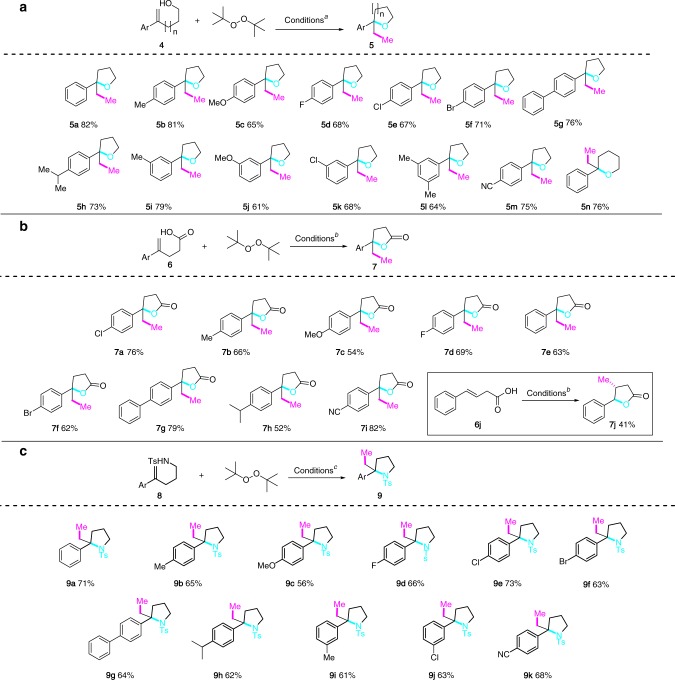
Methylative heterocyclization of alkenes. **a** Methylative cycloetherification: **4** (0.2 mmol), Cu(OTf)_2_ (0.2 equiv), **L1** (0.3 equiv), Na_3_PO_4_ (0.2 equiv), DTBP (4.0 equiv), CF_3_CH_2_OH (*c* 0.1 M), 120 °C. Yields refer to isolated products. **b** Methylative lactonization: **6** (0.2 mmol), CuSO_4_ (0.2 equiv), **L2** (0.3 equiv), Na_3_PO_4_ (0.3 equiv), DTBP (4.0 equiv), *t*BuOH (*c* 0.1 M), 120 °C. **c** Methylative cycloamination: **8**, Cu(OAc)_2_ (0.2 equiv), **L2** (0.3 equiv), Na_3_PO_4_ (0.2 equiv), DTBP (4.0 equiv), *t*BuOH (*c* 0.1 M), 120 °C

We investigated the methylative cycloetherification of alkenes using 4-phenylpent-4-en-1-ol (**4a**, R=H, *n* = 1, Fig. 4a) as a test substrate. Gratefully, treatment of a *t*BuOH solution of **4a** under conditions established for the 1,2-alkoxy methylation of alkenes afforded the tetrahydrofuran **5a** in 58% yield. Replacing DCP with DTBP gave a similar yield of **5a** (57%). Therefore, DTBP was used as a methyl source for further condition optimization as it provided a cleaner reaction mixture. Performing the reaction in *t*BuCN furnished **5a** in only 30% yield. Other solvents such as tetrahydrofuran (THF), dimethoxyethane (DME) and N,N-dimethylformamide (DMF) led to the decomposition or polymerization of **4a**. Finally, trifluoroethanol (TFE) turned out to be an optimum solvent and Cu(OTf)_2_ a slightly better catalyst than Cu(BF_4_)_2_•6H_2_O for this reaction. Overall, the reaction of **4a** with DTBP in trifluoroethanol (*c* 0.1 M) in the presence of Cu(OTf)_2_ (0.2 equiv), 4,4’-dimethoxy-2,2’-dipyridine (**L1**, 0.3 equiv), Na_3_PO_4_ (0.2 equiv) at 120 °C afforded 2-ethyl-2-phenyltetrahydrofuran (**5a**) in 82% yield.

Under the above-optimized conditions, a diverse set of 4-aryl substituted pent-4-en-1-ols **4** underwent methylative cycloetherification to afford the 2,2-disubstituted tetrahydrofurans (**5b**–**5m**) in good yields (Fig. 4a). The reaction tolerated the presence of both electron-donating (Me, OMe, Ph, and *i*Pr) and electron-withdrawing groups (F, Cl, Br, and CN) at different positions of the aryl ring. The 5-phenylhex-5-en-ol underwent the similar methylative cycloetherification to afford 2-ethyl-2-phenyltetrahydro-2H-pyran (**5n**) in 76% yield.

### Methylative lactonization

γ-Butyrolactones are found in many bioactive compounds^57^ and are also useful building blocks in organic synthesis^58^. Consequently, many different synthetic routes have been developed for the synthesis of this important heterocycle^59–64^. Encouraged by the aforementioned results, the methylative lactonization of alkenes was next investigated. The optimum reaction conditions we found consisted of heating a solution of **6** in *t*BuOH (*c* 0.1 M) in the presence of CuSO_4_ (0.2 equiv), 1,10-Phen (**L2**, 0.3 equiv), DTBP (4.0 equiv) and Na_3_PO_4_ (0.2 equiv) at 120 °C. As it is shown in Fig. 4b, electron-donating (Me, OMe, Ph, and *i*Pr) and electron-withdrawing groups (F, Br, Cl, and CN) on the phenyl ring of the α-methyl styrene derivatives were well tolerated leading to γ-lactones (**7a**–**7i**) in good yields. The (*E*)-4-phenylbut-3-enoic acid (**6j**), a 1,2-disubstituted alkene, underwent regioselective methylative lactonization to afford the 4,5-*trans*-disubstituted γ-lactone **7j** as a single isolable diastereomer in 41% yield together with the methyl ester of **6j** (24%).

### Methylative cycloamination

While trifluoromethylative cycloamination of alkenes have been reported recently^65,66^, the methylative counterpart is to the best of our knowledge unknown. We therefore set out to examine this reaction using sulfonamide as internal nucleophile. Optimum conditions found for the methylative cycloamination of **8a** with DTBP (4.0 equiv) consisted of heating a solution of **8a** in *t*BuOH (*c* 0.1 M) in the presence of Cu(OAc)_2_ (0.2 equiv), 1,10-Phen (**L2**) and Na_3_PO_4_ (0.2 equiv) at 120 °C. Under these conditions, the pyrrolidine **9a** was isolated in 71% yield. As it is shown in Fig. 4c, electron-donating (Me, OMe, Ph, and *i*Pr) and electron-withdrawing groups (F, Br, Cl, and CN) on the phenyl ring of the α-methyl styrene derivatives were well tolerated leading to 2,2-disubstituted pyrrolidines (**9a–9k**) in good yields.

### Mechanistic studies

**Fig. 5:**
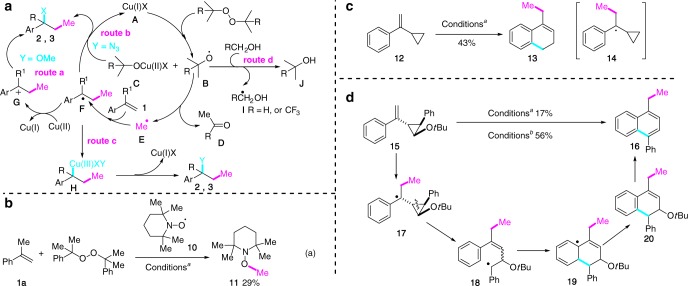
Mechanistic proposal and control experiments. **a** Possible reaction pathways. **b** Radical trapping experiment. **c** Radical clock experiment. **d** Super sensitive radical probe experiment. Conditions *a*: **1a** (0.2 mmol), Cu(BF_4_)_2_•6H_2_O (0.2 equiv), **L1** (0.3 equiv), DCP (4.0 equiv), Na_2_HPO_4_ (0.2 equiv), MeOH (2.0 mL, *c* 0.1 M), 120 °C, 4 h; Conditions *b*: **1a** (0.2 mmol), DTBP (0.8 mmol), LiN_3_ (0.5 mmol), CuSO_4_ (0.002 mmol, 0.1 equiv), **L2** (0.06 mmol), *t*BuOH (2.0 mL, *c* 0.1 M), 120 °C

Possible reaction pathways for the 1,2-alkoxy methylation and 1,2-azido methylation of alkenes are depicted in Fig. 5a. Reduction of peroxide (DCP or DTBP) by the in situ generated Cu(I)X salt **A** would produce the *tert*-alkoxy radical **B** and Cu(II) salt **C**. Alternatively, thermal decomposition of peroxide would generate two molecules of alkoxy radical **B**. β-Scission of **B** would generate ketone **D** and methyl radical **E**. Addition of **E** to the alkene would produce the benzyl radical **F** which would be oxidized by Cu(II) salt **C** to the carbenium **G** with the concurrent regeneration of the Cu(I)X salt. Trapping of the carbenium **G** by nucleophile would then afford the observed products (route a). Alternatively, radical **F** could be directly converted to the adduct via a Cu-centered redox transfer process (route b) or via radical rebound of **F** with **C** followed by reductive elimination of the resulting Cu(III) species **H** (route c) ^64^.

Several experimental observations and the results of control experiments were in line with the proposed reaction pathway. First, 1,2-methoxy methylation of **1a** was completely inhibited in the presence of 2,2,6,6-tetramethyl-1-piperidinyloxy (TEMPO, **10**). 1-(Methoxy)-2,2,6,6-tetramethylpiperidine (**11**) was instead isolated in 29% yield (Fig. 5b). Second, submitting (1-cyclopropylvinyl)benzene (**12**) to the standard 1,2-methoxy methylation conditions afforded dihydronaphthalene **13** in 43% yield (Fig. 5c). These two experiments clearly indicated the existence of both the methyl radical (Me•) and the adduct radical **14** in this three-component process. To gain further insight on the reaction mechanism, the 2-*tert*-butoxy-3-(1-phenylvinyl)cyclopropyl)benzene (**15**), developed by Newcomb as a supersensitive radical probe, was synthesized^67,68^. It has been demonstrated that the cyclopropane will be opened at the phenyl-bearing carbon in a radical mechanism and at the oxygen-bearing carbon in a cationic mechanism. Eventually, treatment of **15** under our methoxy methylation conditions afforded a quite complex reaction mixture from which 1,4-disubstituted naphthalene **16** was isolated in 17% yield. On the other hand, compound **15** was converted, under 1,2-azido methylation conditions, cleanly to **16** in 56% yield (Fig. 5d). Formation of benzyl radical **17** followed by regioselective ring opening to **18** and intramolecular HAS reaction would provide dihydronaphthalene **20** which, upon elimination of *t*BuOH, would afford naphthalene **16**. The observed regioselective ring opening of cyclopropane supported the involvement of the benzylic radical **17** as a possible reactive intermediate.

The significant difference in the yield of **16** from radical clock probe **15** under the methoxy methylation and azido methylation conditions was intriguing. We tentatively attributed to the different oxidation power of the copper salts. CuSO_4_ is known to be a weaker oxidant than Cu(BF_4_)_2_•6H_2_O, the benzylic radical generated under the azido methylation conditions (CuSO_4_-catalyzed) would, therefore, have a longer half-life than that generated under methoxy methylation conditions [Cu(BF_4_)_2_•6H_2_O-catalyzed], hence the clean formation of product **16**. This led us to hypothesize that the C–O bond formation in the present alkoxy methylation went through cationic intermediate (route a, Fig. 5a), whereas the C–N bond formation in the azido methylation proceeded via the Cu-mediated azide transfer process (route b or c, Fig. 5a).

**Fig. 6:**
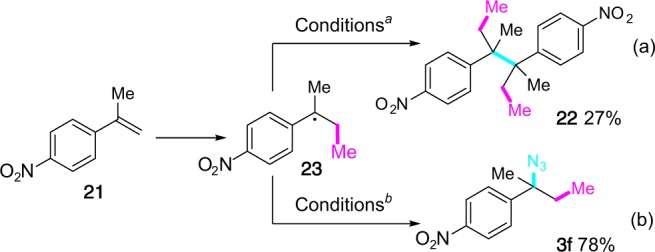
Mechanistic divergence between methoxy methylation and azido methylation. **a**: **21** (0.2 mmol), Cu(BF_4_)_2_•6H_2_O (0.2 equiv), **L1** (0.3 equiv), DCP (4.0 equiv), Na_2_HPO_4_ (0.2 equiv), MeOH (2.0 mL, *c* 0.1 M), 120 °C, 4 h; **b**: **21** (0.2 mmol), DTBP (0.8 mmol), LiN_3_ (0.5 mmol), CuSO_4_ (0.002 mmol, 0.1 equiv), **L2** (0.06 mmol), *t*BuOH (2.0 mL, *c* 0.1 M), 120 °C

In accordance with the aforementioned reaction manifolds, 1,2-methoxy methylation of 1-methyl-1-(4-nitrophenyl)ethylene (**21**) under standard conditions afforded a significant amount of dimer **22** and only a trace amount of the desired methoxy methylation product (Fig. 6a). The presence of the strong electron-withdrawing nitro group on the phenyl ring might significantly reduce the rate of the oxidation of benzyl radical **23** to carbenium, blocking therefore the methoxylation process. It underwent instead the dimerization to afford **22**. On the other hand, treatment of **21** under standard azido methylation conditions afforded the three-component adduct **3f** in 78% yield together with a small amount of dimer **22** (Fig. 6b). The result supported the notion that oxidation of radical to cation is not involved in the azidation step and the azido group was transferred directly to the radical **23** via presumably a Cu-mediated redox transfer process. The azide transfer reaction was apparently faster than the dimerization process under our optimized azido methylation conditions. It is also worth noting that dimer was rarely observed under the optimized methoxy methylation of alkenes due presumably to the rapid oxidation of benzyl radical to benzyl cation (except for **21**), while it was very often observed as a side product in the azido methylation process due to the relatively long-lived benzyl radical species. Finally, performing the azidomethylation of α-methylstyrene (**1a**) in MeOH and *t*BuOH/MeOH (v/v = 4:1) under otherwise standard conditions afforded the desired product **3a** in yields of 46 and 62%, respectively. The potential competitive reaction leading to the 1,2-methoxy methylated product **2a** was not observed. This result reinforced the hypothesis that benzyl cation might not be involved in the azidomethylation of alkenes.

At the outset of this research, we were concerned about the hydrogen abstraction of MeOH by *tert*-alkoxy radical **B** to generate the hydroxymethyl radical **I** (route d, Fig. 5a). This process has indeed been exploited in the difunctionalization of activated alkenes^69,70^. Two pathways, namely, thermal decomposition and reduction by Cu(I) salt, may contribute to the generation of the radical **B** from the peroxide. The formal process generates two molecules of alkoxy radical **B**, while the latter produces one molecule of **B** and one molecule of copper *tert*-butoxide **C**. Therefore, it was difficult to quantify the ratio of β-scission of **B** (generating Me•) vs H-abstraction of MeOH by **B** (leading to •CH_2_OH) based on the ratio of acetophenone (**D**) vs 2-phenylpropan-2-ol (**J**). Nevertheless, the high **J**/**D** ratio (3/1) we obtained for the methylative methoxylation of α-methylstyrene (**1a**) indicated that route d, a thermodynamically favorable process (BDE of H-CH_2_OH: 96.06 ± 0.15 kcal/mol; *t*BuO-H: 106.3 ± 0.7 kcal/mol), was indeed occurring in parallel. However, the so-generated hydroxymethyl radical **I** did not interfere with the methylation process probably due to the pronounced nucleophilic nature of this radical or its rapid oxidation to formaldehyde.

In summary, we reported the Cu-catalyzed carboalkoxylation, carboazidation, carbocycloetherification, carbolactonization, and carbocycloamination of alkenes using dicumyl peroxide (DCP) or di-*tert*-butyl peroxide (DTBP) as methyl sources. A diverse set of styrene derivatives were converted to the methylated ethers, azides, tetrahydrofurans, tetrahydropyrans, γ-lactones, and pyrrolidines with concurrent generation of a quaternary carbon in good to excellent yields. The results of control experiments suggested that the 1,2-alkoxy methylation of alkenes went through a radical-cation crossover mechanism, whereas the azido methylation proceeded via a radical addition and Cu-mediated redox azide transfer process. This mechanistic insight would serve as a guideline in our searching for new alkene difunctionalization protocols.

## Methods

### Three-component 1,2-alkoxy methylation of alkenes

A screw cap tube was charged with Cu(BF_4_)_2_•6H_2_O (13.8 mg, 0.0400 mmol), 4,4’-dimethoxy-2,2’-bipyridine **L1** (13.0 mg, 0.0601 mmol), Na_2_HPO_4_ (5.7 mg, 0.0402 mmol) and R^3^OH (2.0 mL). The mixture was stirred at room temperature for 30 min, then substrate **1** (0.2 mmol, 1.0 equiv) and DCP (216.2 mg, 0.800 mmol) or DTBP (0.15 mL, 4.0 equiv) were added to the above mixture. After being stirred for 4 h at 120 °C under N_2_ atmosphere, the reaction mixture was quenched with water and the aqueous phase was extracted with EtOAc. The organic extracts were washed with brine, dried over Na_2_SO_4_. The solvent was removed under reduced pressure. The residue was purified by flash chromatography to give **2**.

### Three-component 1,2-azido methylation of alkenes

A screw cap tube was charged with CuSO_4_ (0.32 mg, 0.002 mmol, 0.01 equiv), 1,10-phenanthroline **L2** (1.08 mg, 0.003 mmol, 0.03 equiv) and *t*BuOH (2.0 mL). The mixture was stirred at 40 °C for 30 min, then cooled to room temperature. Substrate **1** (0.2 mmol, 1.0 equiv), LiN_3_ (20% w/w, 0.12 mL, 2.5 equiv) and DTBP (0.15 mL, 4.0 equiv) were added to the above mixture, and the reaction mixture was stirred at 120 °C for 8 h under N_2_ atmosphere. The reaction was quenched with water and the aqueous phase was extracted with EtOAc. The organic extracts were washed with brine, dried over Na_2_SO_4_. The solvent was removed under reduced pressure. The residue was purified by flash chromatography to give **3**.

### Methylative cycloetherification

A screw cap tube was charged with Cu(OTf)_2_ (14.5 mg, 0.04 mmol, 0.2 equiv), **L1** (13.0 mg, 0.06 mmol, 0.03 equiv) and CF_3_CH_2_OH (2.0 mL). The mixture was stirred at room temperature for 30 min. Substrate **4** (0.2 mmol, 1.0 equiv), Na_3_PO_4_ (6.5 mg, 0.04 mmol, 0.2 equiv) and DTBP (0.15 mL, 4.0 equiv) were added to the above mixture, and the reaction mixture was stirred at 120 °C for 6 h under N_2_ atmosphere. The reaction was quenched with water, and the aqueous phase was extracted with EtOAc. The organic extracts were washed with brine, dried over Na_2_SO_4_. The solvent was removed under reduced pressure. The residue was purified by flash chromatography to give **5**.

### Methylative lactonization

A screw cap tube was charged with CuSO_4_ (6.4 mg, 0.04 mmol, 0.2 equiv), 1,10-phenanthroline **L2** (10.8 mg, 0.06 mmol, 0.03 equiv) and CF_3_CH_2_OH (2.0 mL). The mixture was stirred at room temperature for 30 min. Substrate **6** (0.2 mmol, 1.0 equiv), Na_3_PO_4_ (9.8 mg, 0.06 mmol, 0.3 equiv) and DTBP (0.15 mL, 4.0 equiv) were added to the above mixture, and the reaction mixture was stirred at 120 °C for 6 h under N_2_ atmosphere. The reaction was quenched with water and extracted with EtOAc. The organic extracts were washed with brine, dried over Na_2_SO_4_. The solvent was removed under reduced pressure. The residue was purified by flash chromatography to give **7**.

### Methylative cycloamination

A screw cap tube was charged with Cu(OAc)_2_ (7.3 mg, 0.04 mmol, 0.2 equiv), 1,10-phenanthroline **L2** (10.8 mg, 0.06 mmol, 0.03 equiv), and *t*BuOH (2.0 mL). The mixture was stirred at room temperature for 30 min. Substrate **8** (0.2 mmol, 1.0 equiv), Na_3_PO_4_ (6.5 mg, 0.04 mmol, 0.2 equiv) and DTBP (0.15 mL, 4.0 equiv) were added to the above mixture, and the reaction mixture was stirred at 120 °C for 3 h under N_2_ atmosphere. The reaction was quenched with water and the mixture was extracted with EtOAc. The organic extracts were washed with brine, dried over Na_2_SO_4_. The solvent was removed under reduced pressure. The residue was purified by flash chromatography to give **9**.

## Electronic supplementary material

Supplementary Information

## Data Availability

The authors declare that the data supporting the findings of this study are available within the paper and Supplementary Information, as well as from the authors upon request.
