# Supplementary material for: Copper-catalyzed methylative difunctionalization of alkenes
Source: Nat Commun. 2018 Sep 13;9:3725. doi: 10.1038/s41467-018-06246-6 (PMC6137206; doi:10.1038/s41467-018-06246-6)
Supplement: Supplementary file 1 — Supplementary Information [file 41467_2018_6246_MOESM1_ESM.pdf]

Supplementary Information

Copper-catalyzed methylative difunctionalization of alkenes

Xu Bao, Takayuki Yokoe, Tu M. Ha, Qian Wang and Jieping Zhu*

Laboratory of Synthesis and Natural Products, Institute of Chemical Sciences and Engineering, Ecole Polytechnique Fédérale de Lausanne, EPFL-SB-ISIC-LSPN, BCH5304, CH-1015 Lausanne (Switzerland)

E-mail: jieping.zhu@epfl.ch

Supplementary Figures

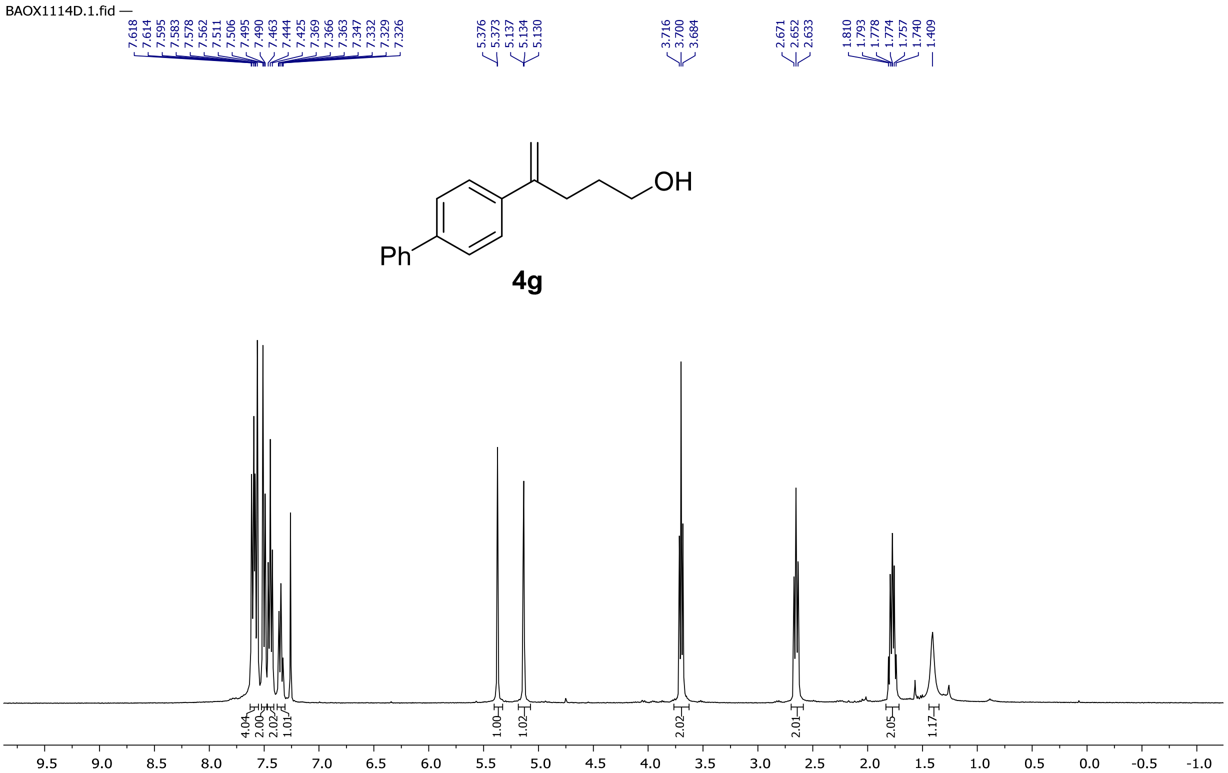

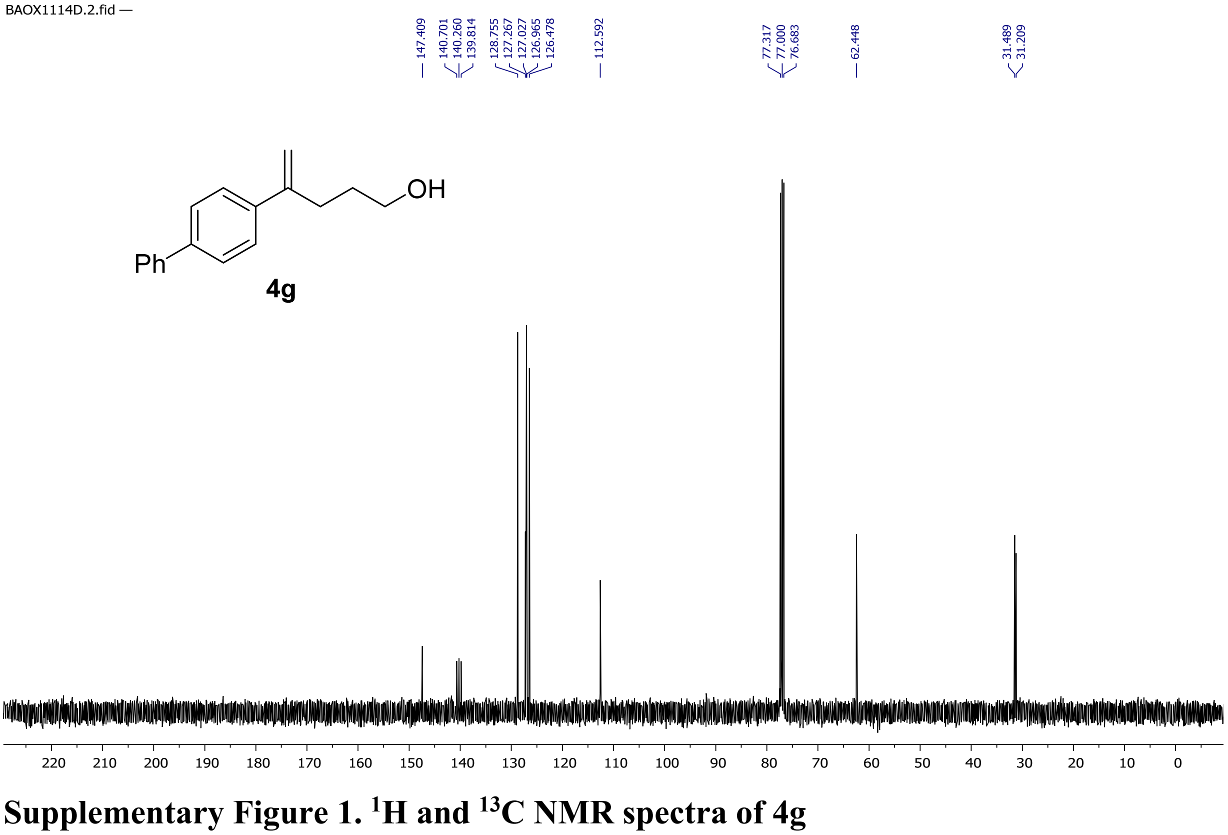

Supplementary Figure 1. ^{1}H and ^{13}C NMR spectra of 4g

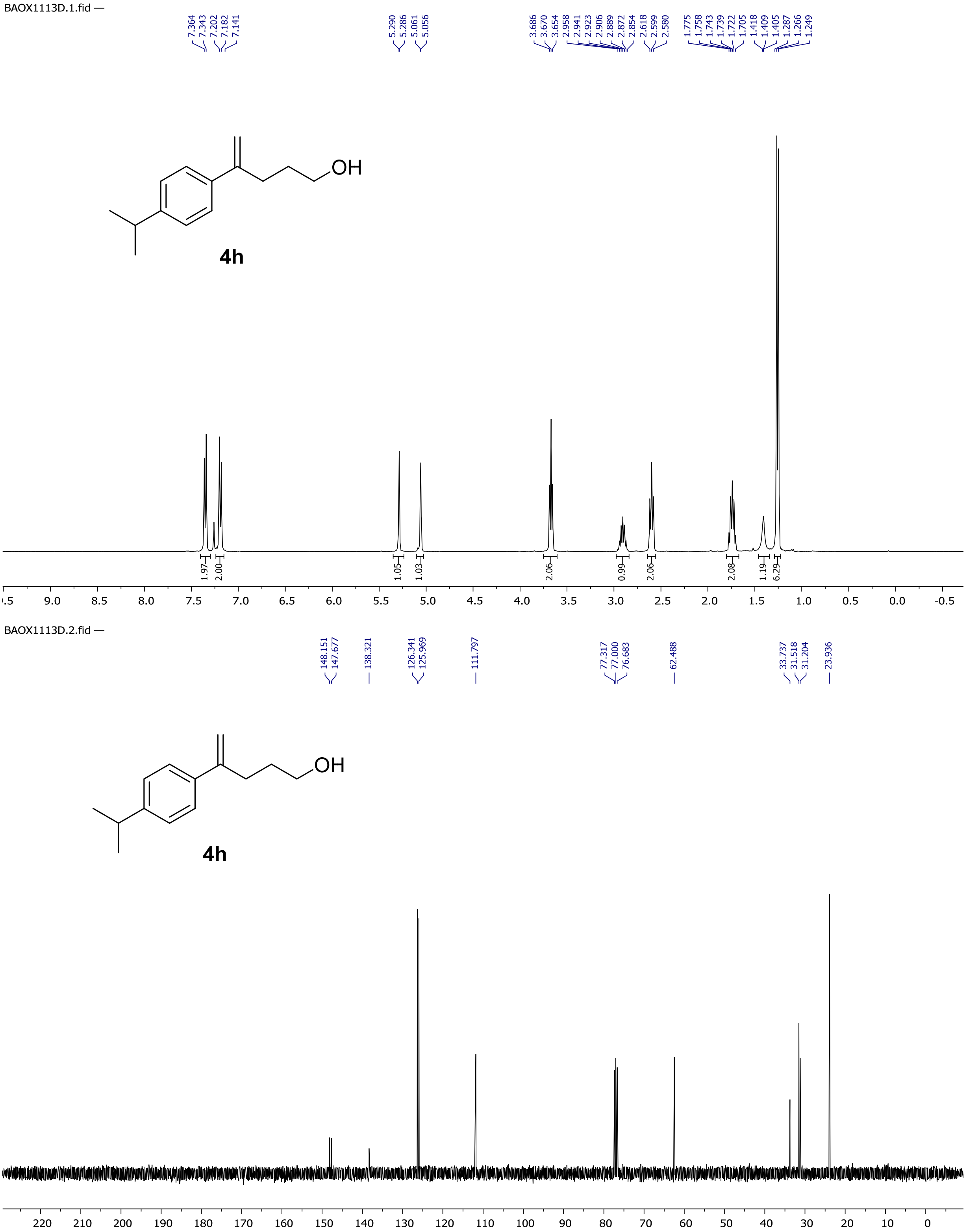

Supplementary Figure 2. ^{1}H and ^{13}C NMR spectra of 4h

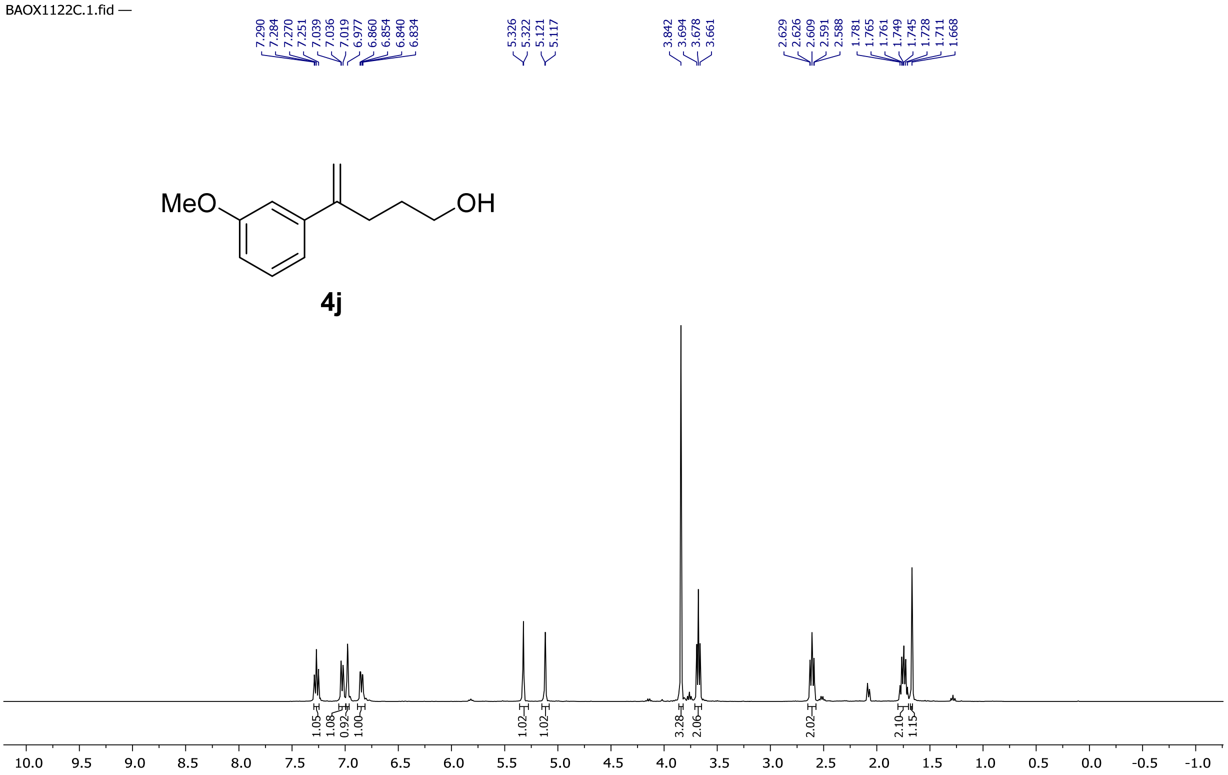

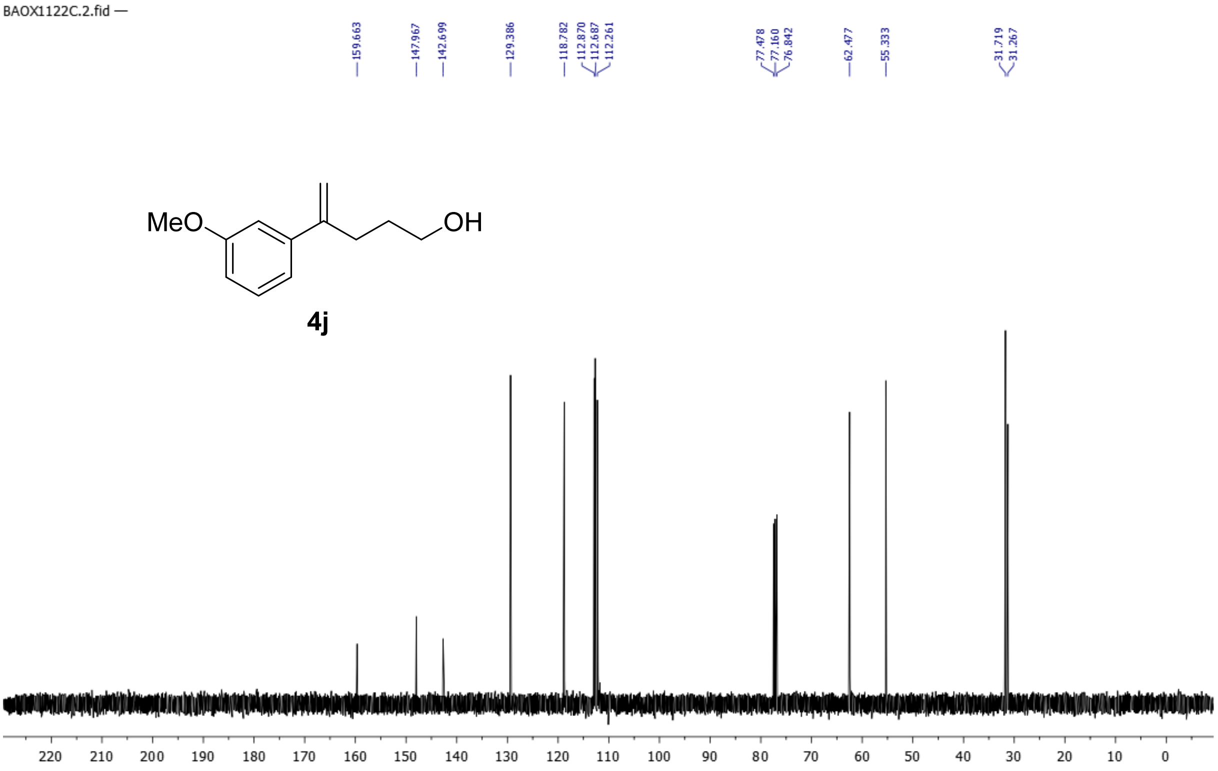

Supplementary Figure 3. ^{1}H and ^{13}C NMR spectra of 4j

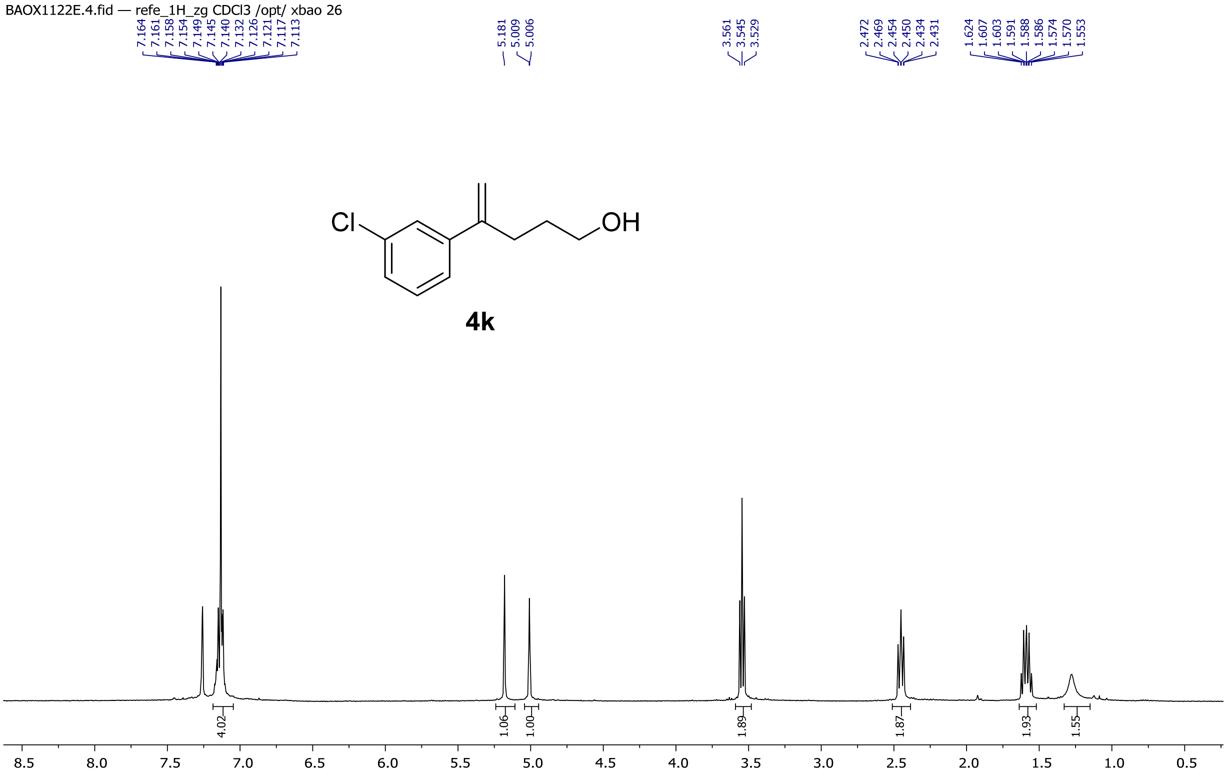

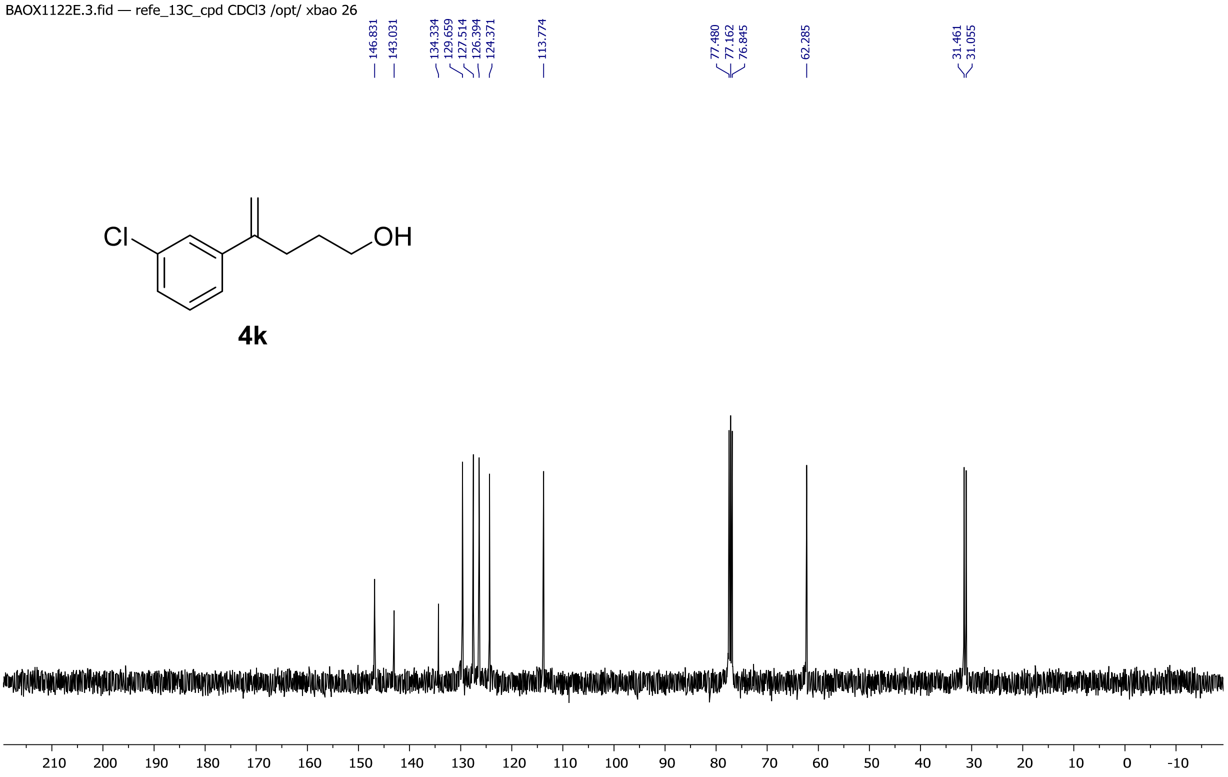

Supplementary Figure 4. ^{1}H and ^{13}C NMR spectra of 4k

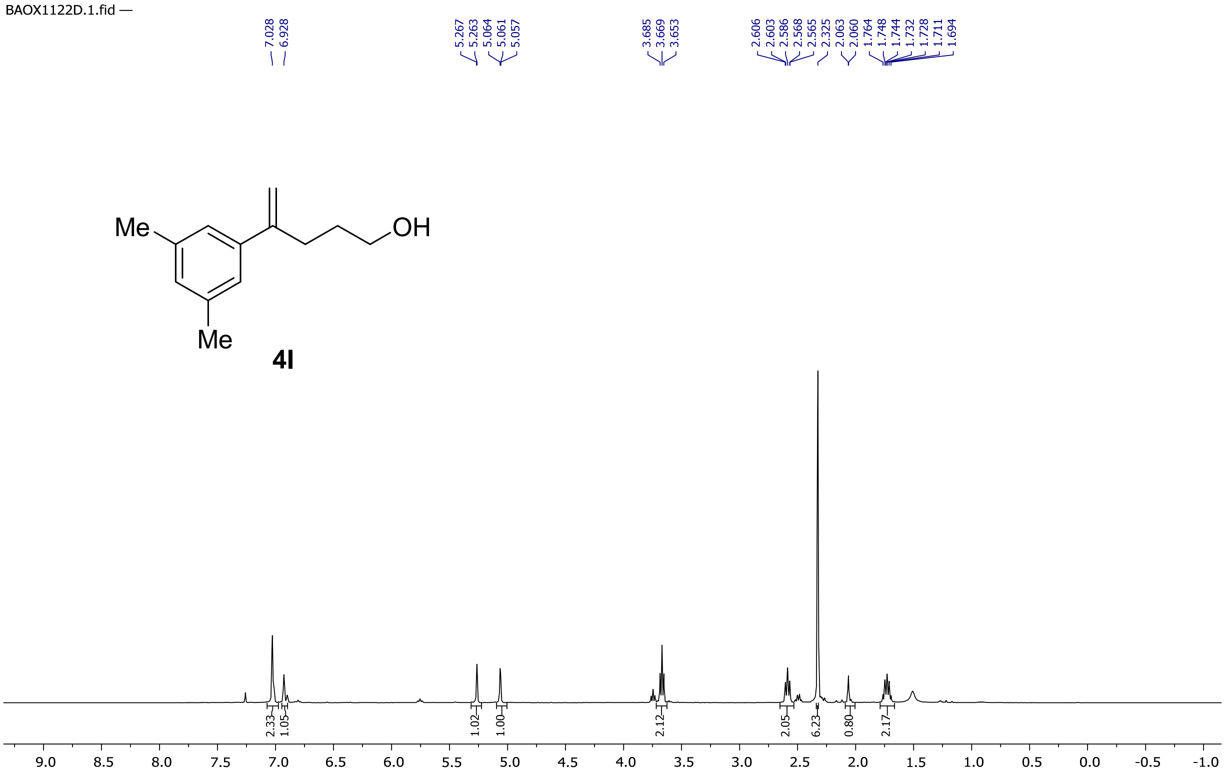

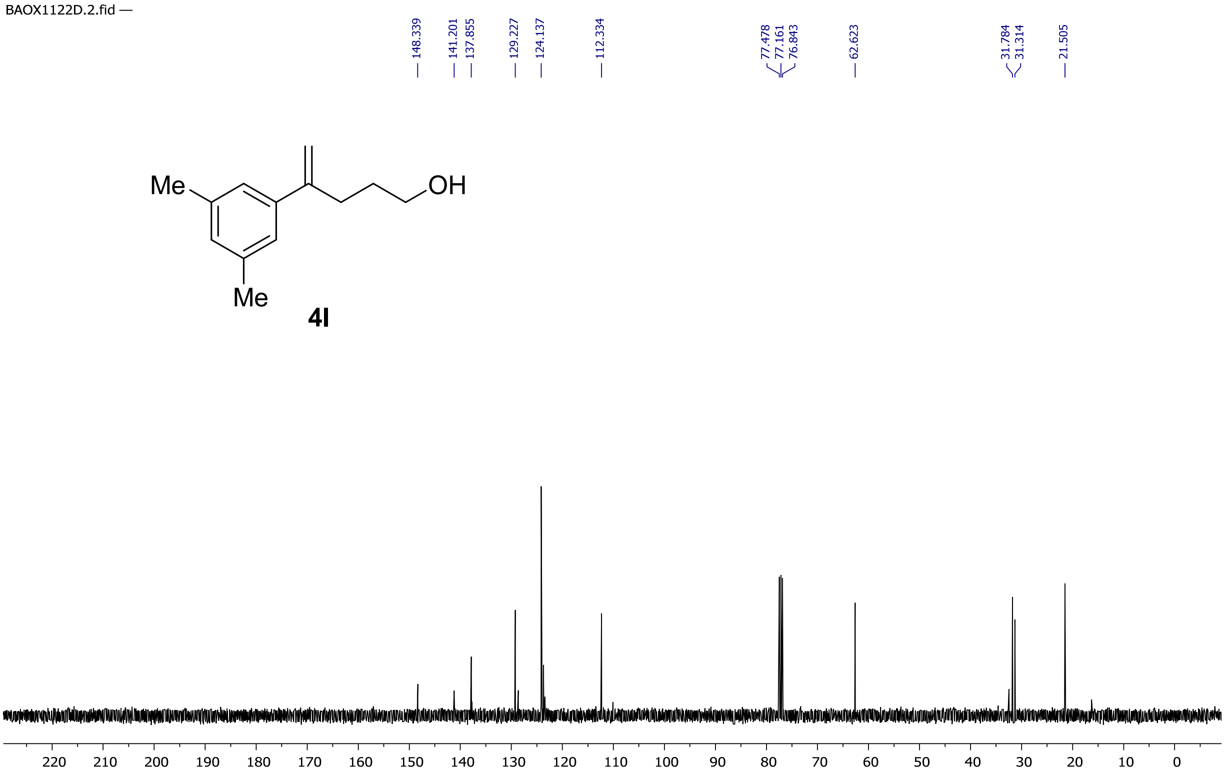

Supplementary Figure 5. ^{1}H and ^{13}C NMR spectra of 4l

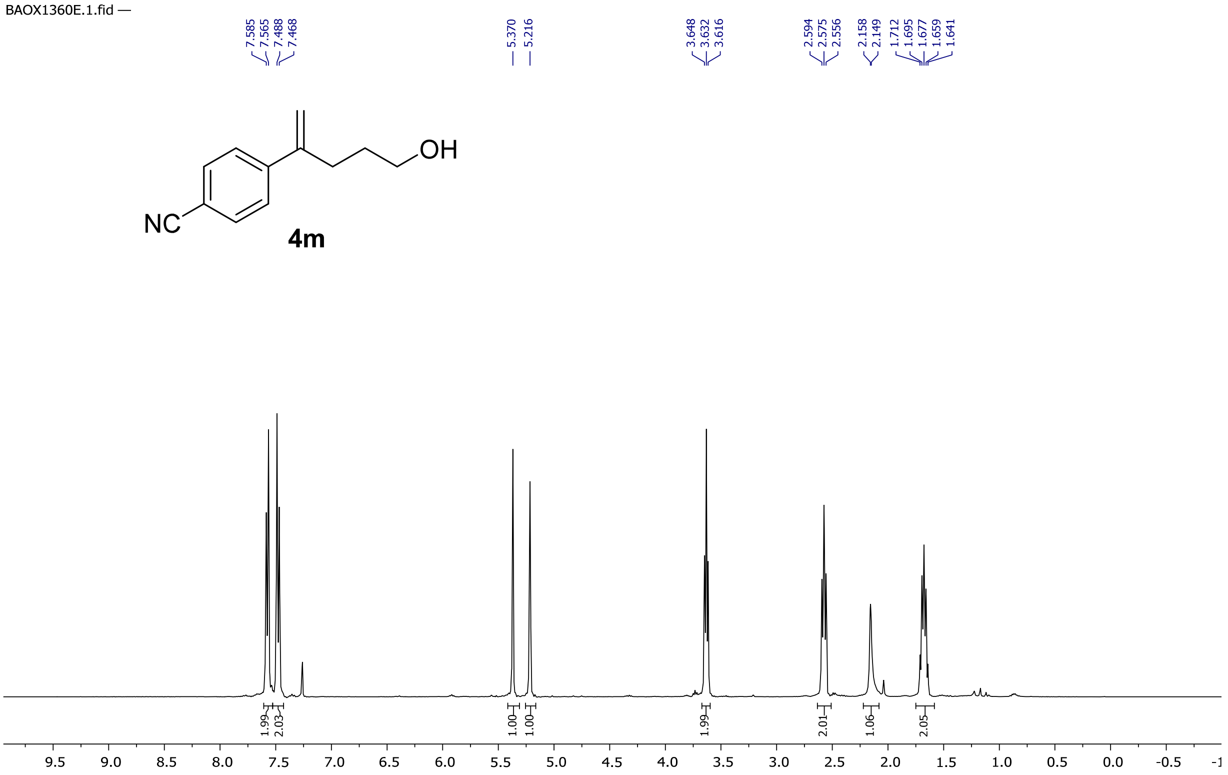

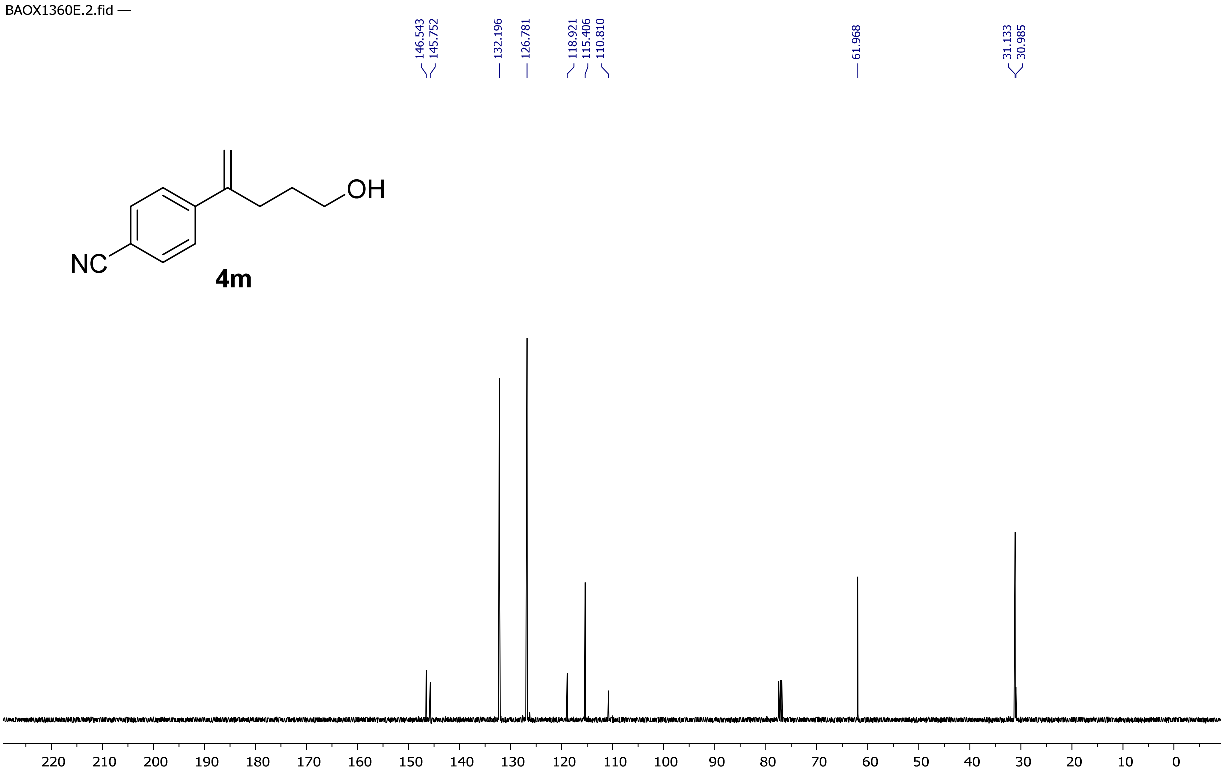

Supplementary Figure 6. ^{1}H and ^{13}C NMR spectra of 4m

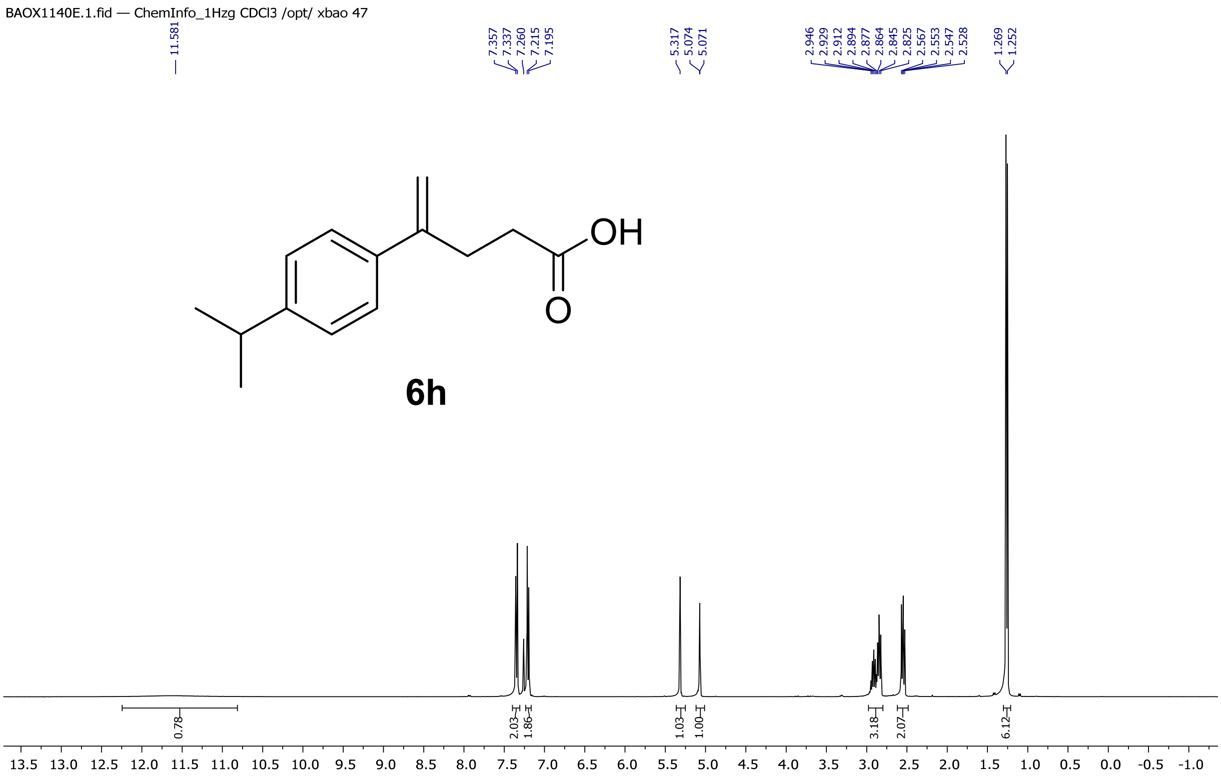

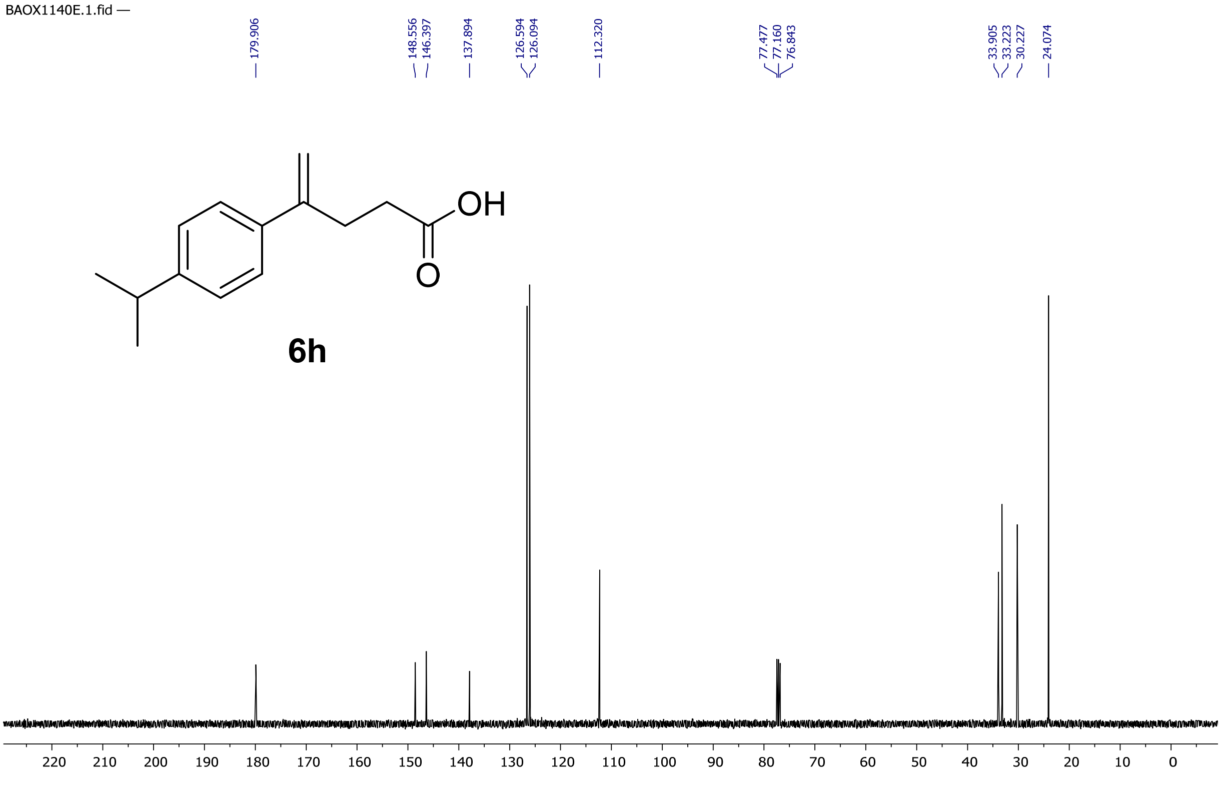

Supplementary Figure 7. ^{1}H and ^{13}C NMR spectra of 6h

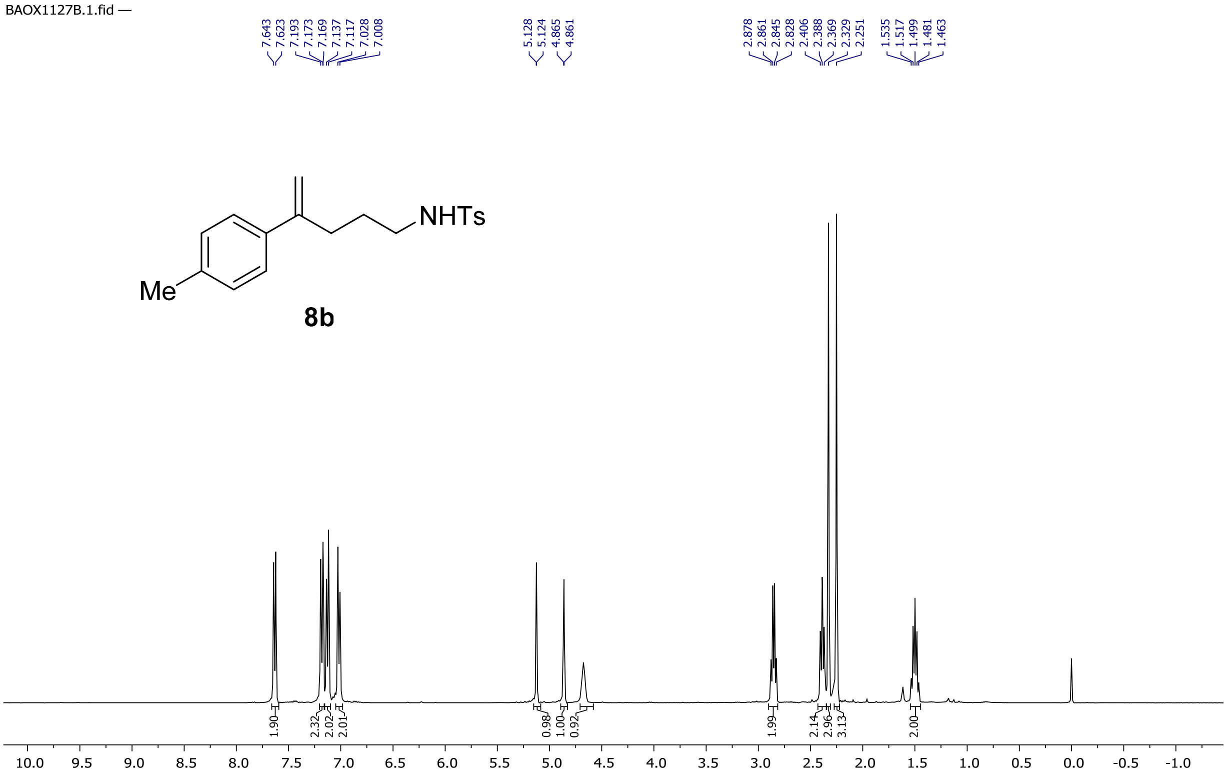

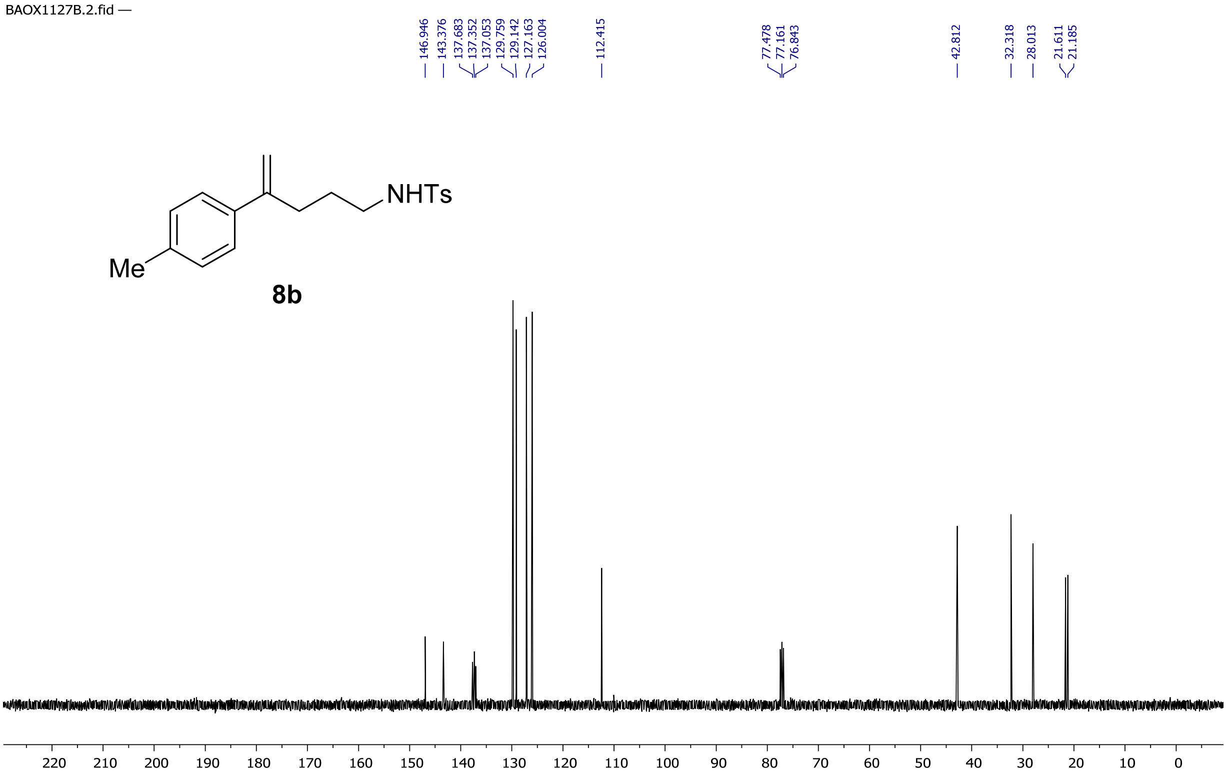

Supplementary Figure 8. ^{1}H and ^{13}C NMR spectra of 8b

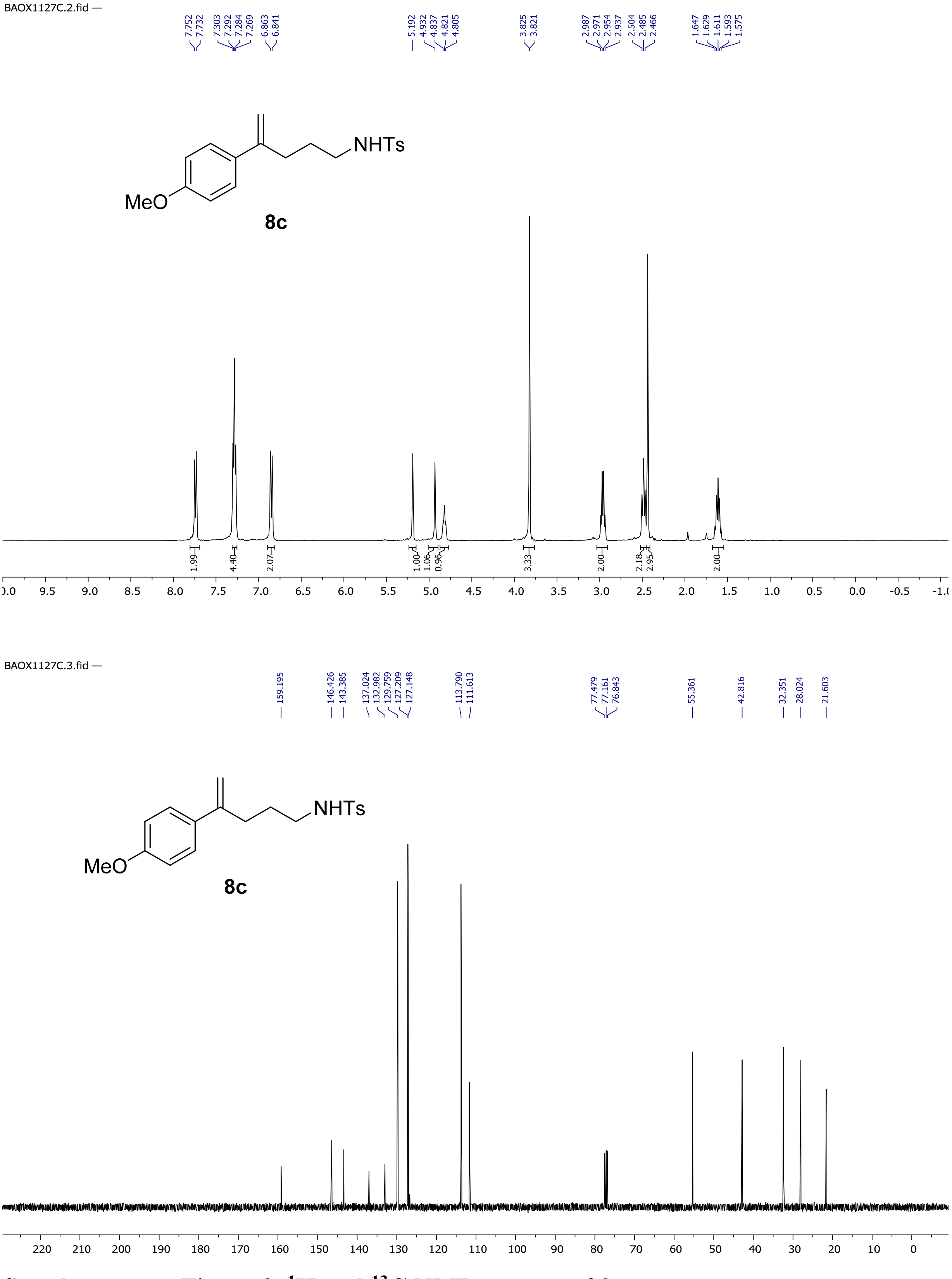

Supplementary Figure 9. ^{1}H and ^{13}C NMR spectra of 8c

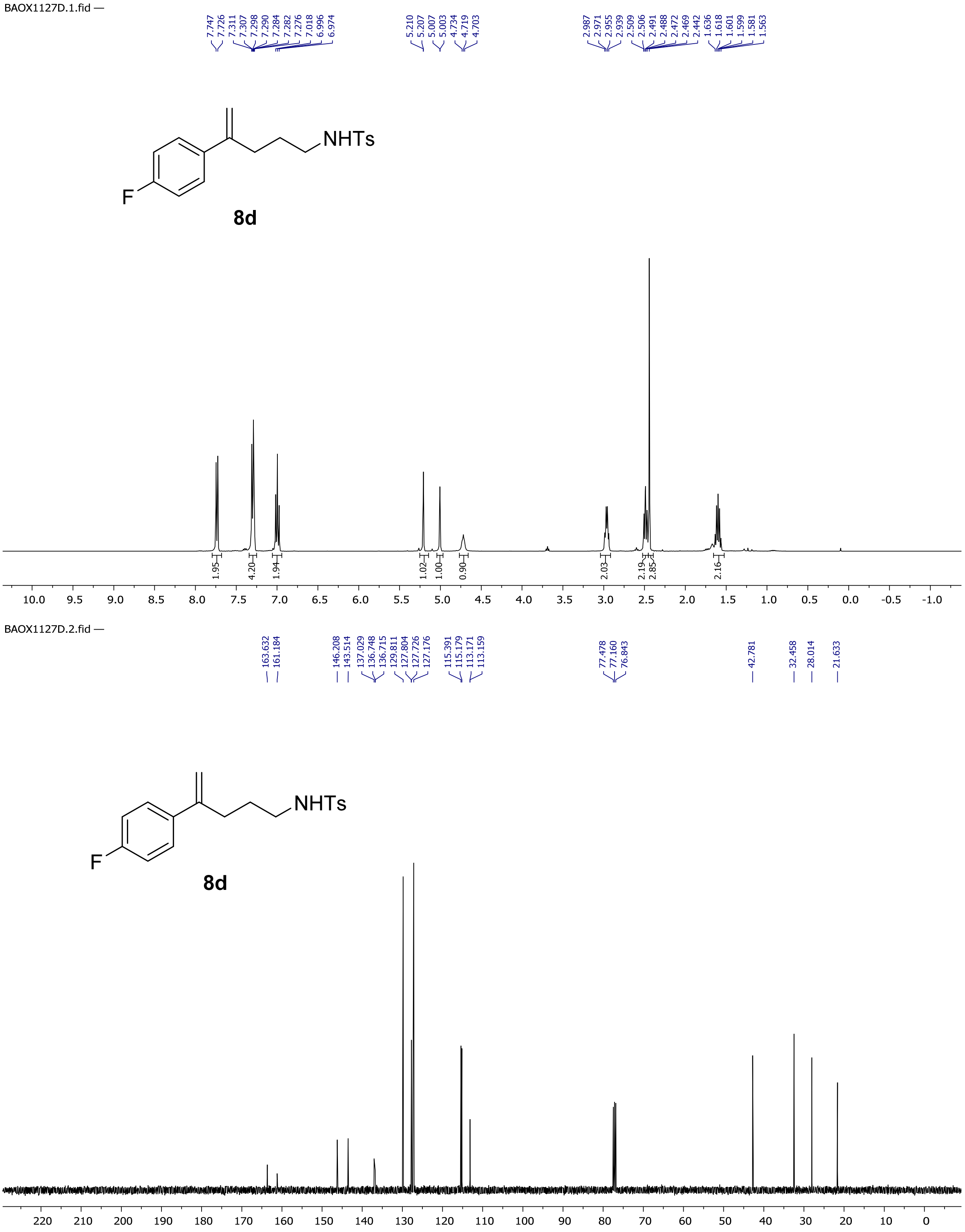

Supplementary Figure 10. ^{1}H and ^{13}C NMR spectra of 8d

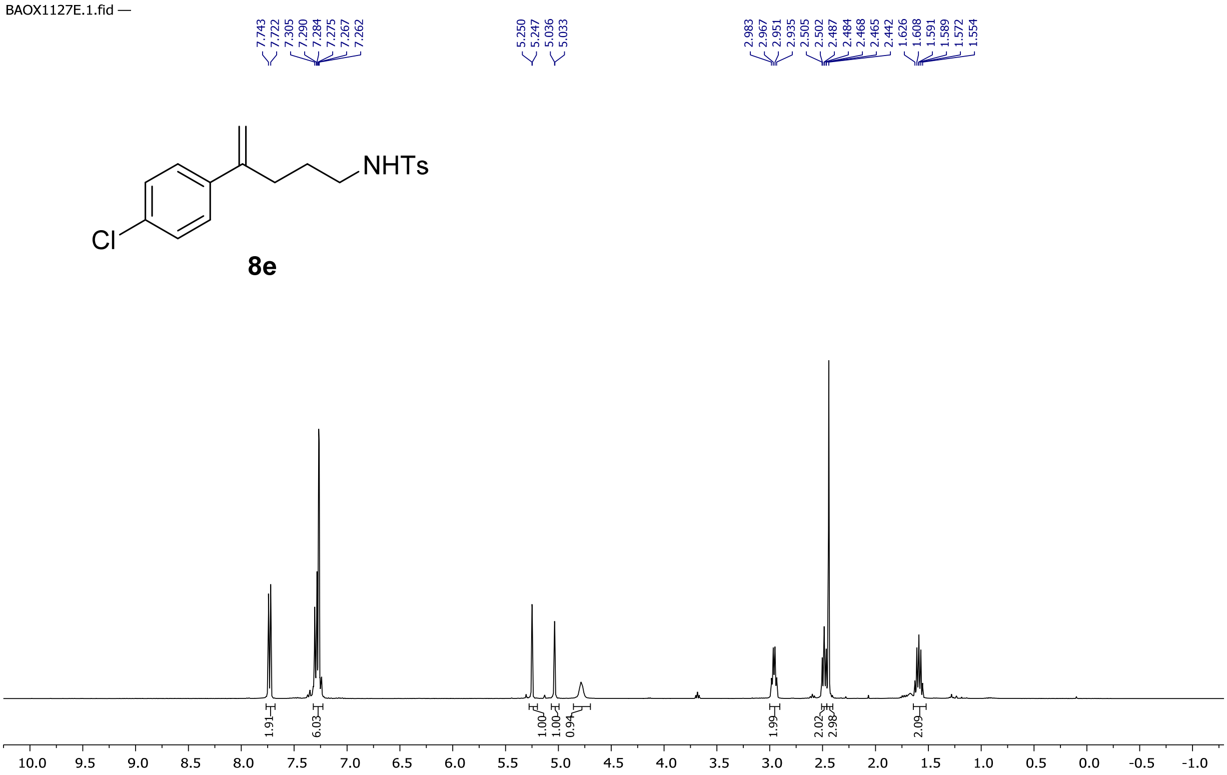

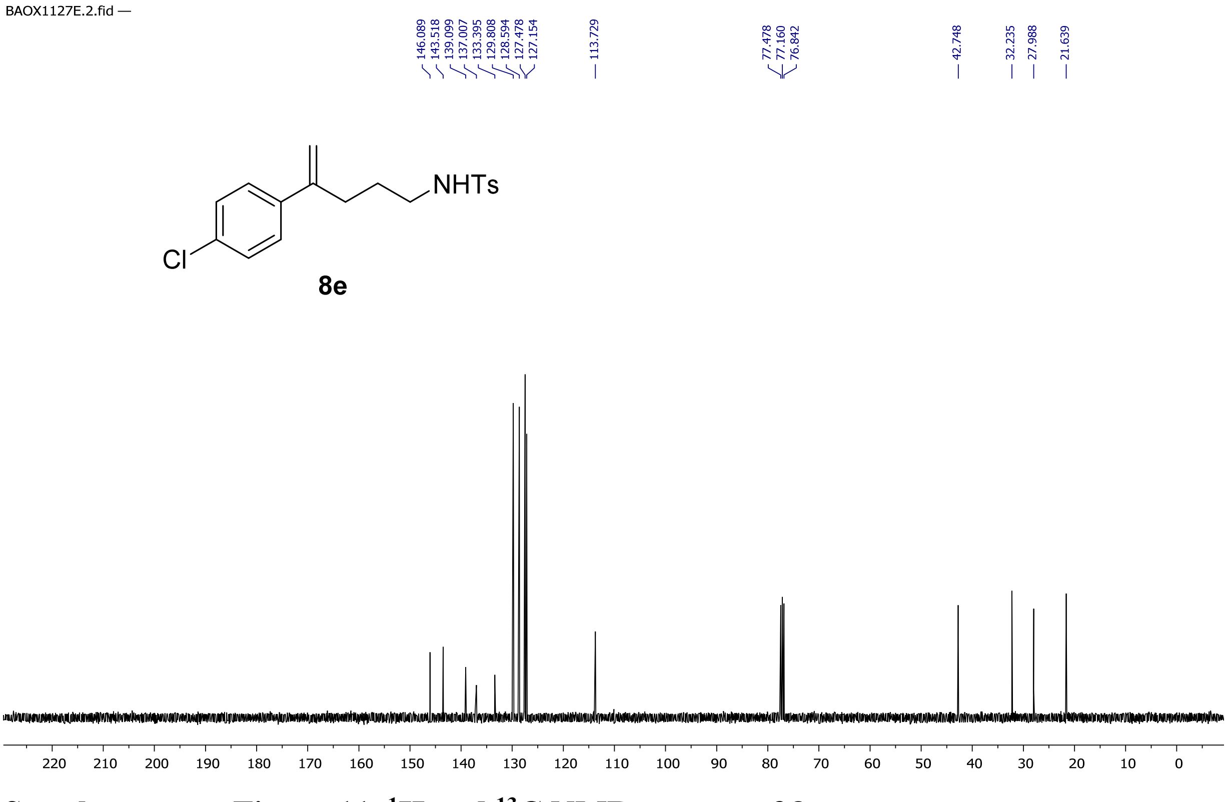

Supplementary Figure 11. ^{1}H and ^{13}C NMR spectra of 8e

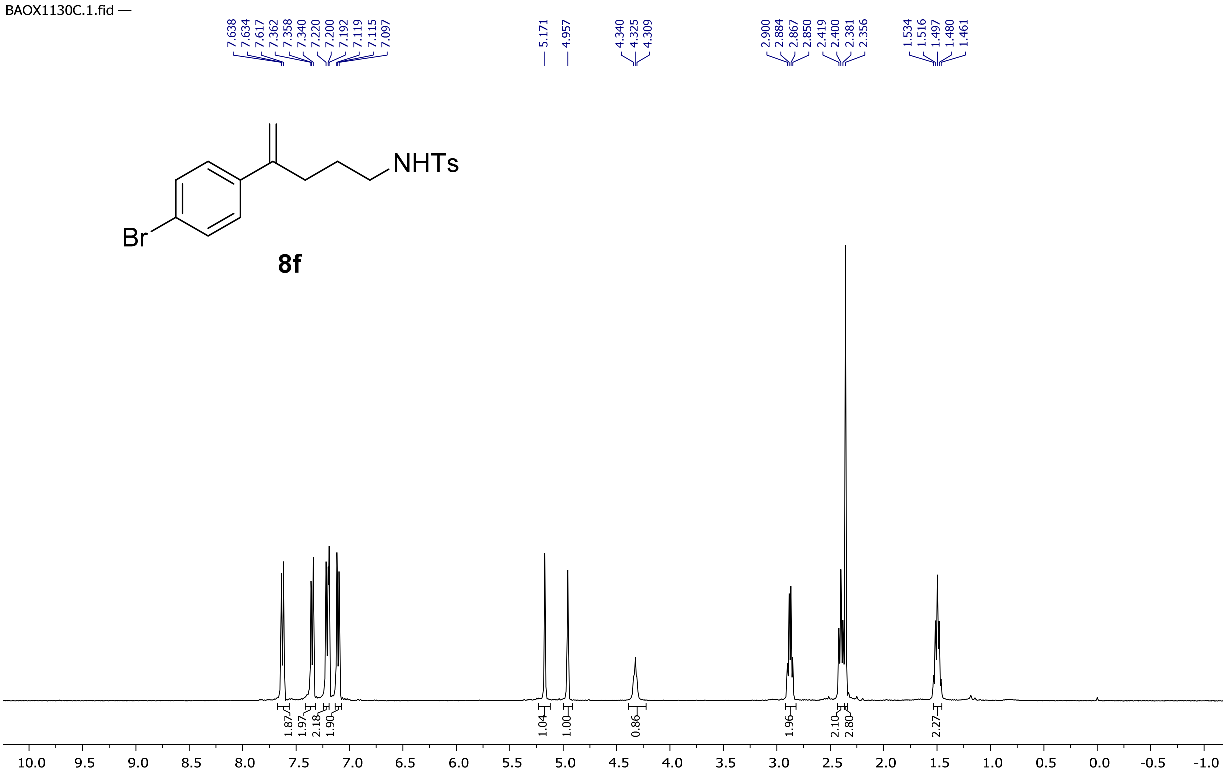

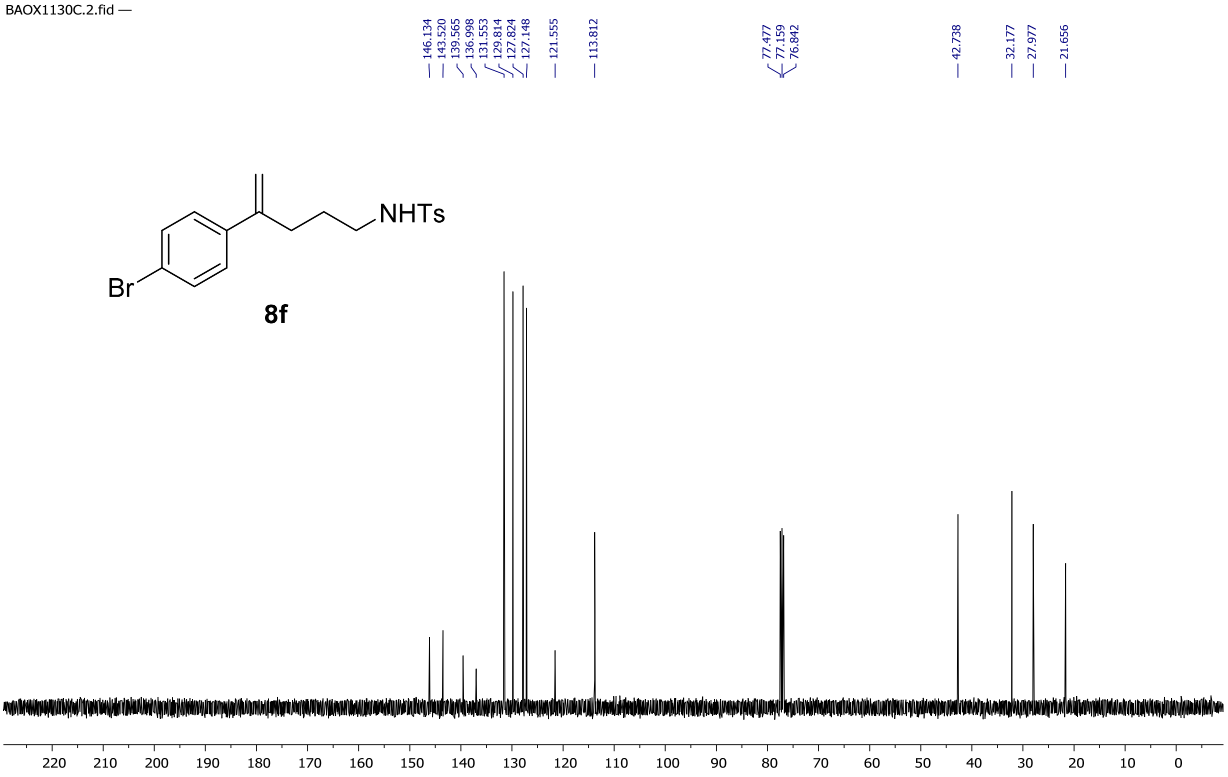

Supplementary Figure 12. ^{1}H and ^{13}C NMR spectra of 8f

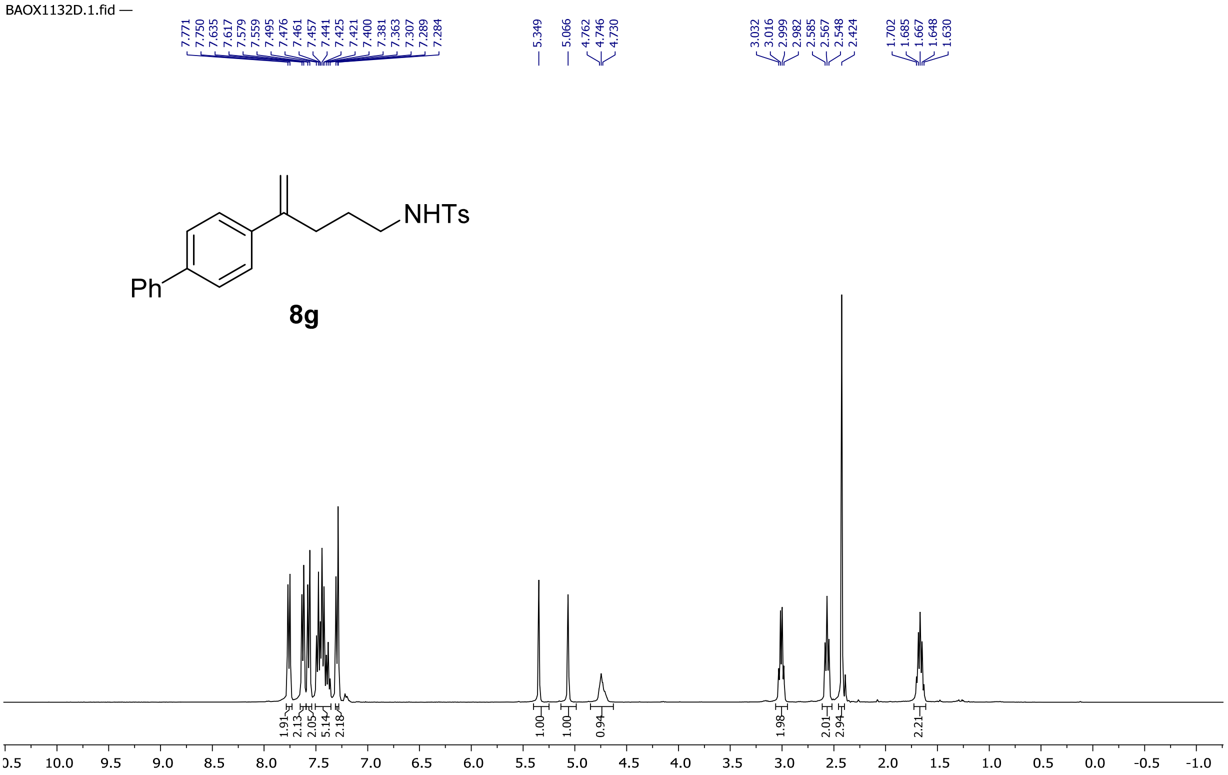

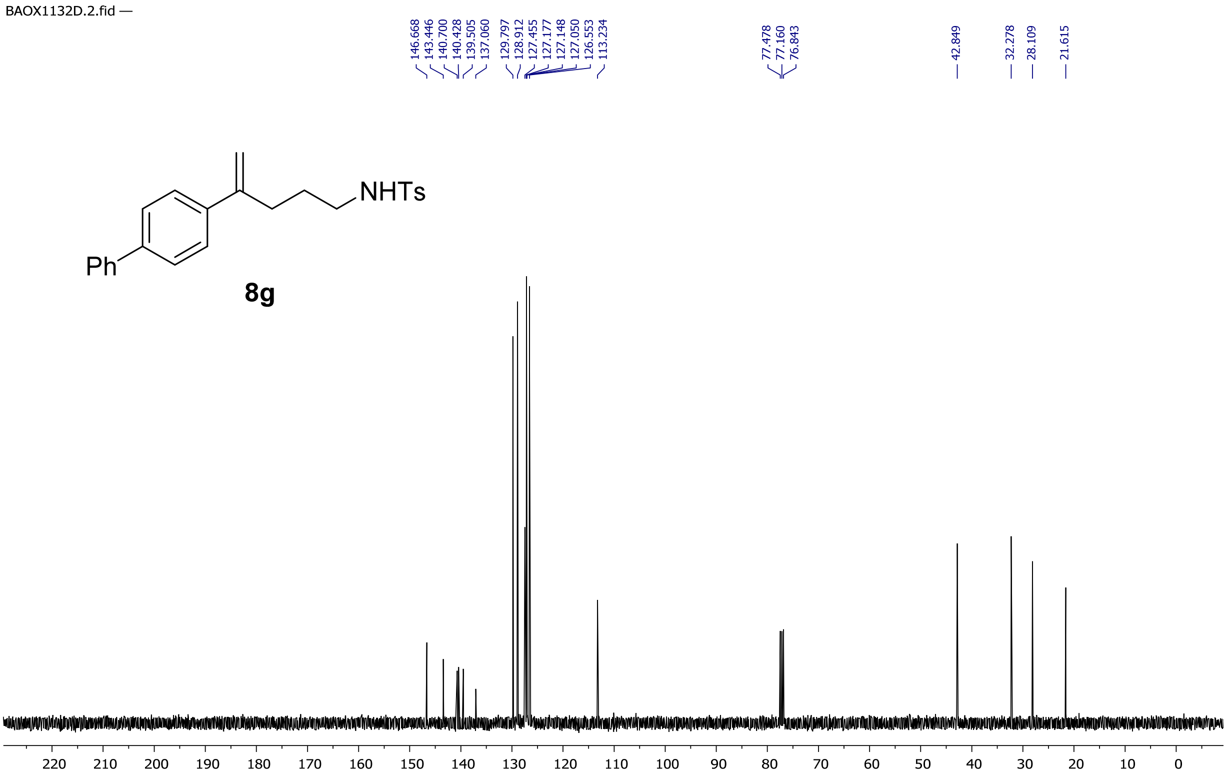

Supplementary Figure 13. ^{1}H and ^{13}C NMR spectra of 8g

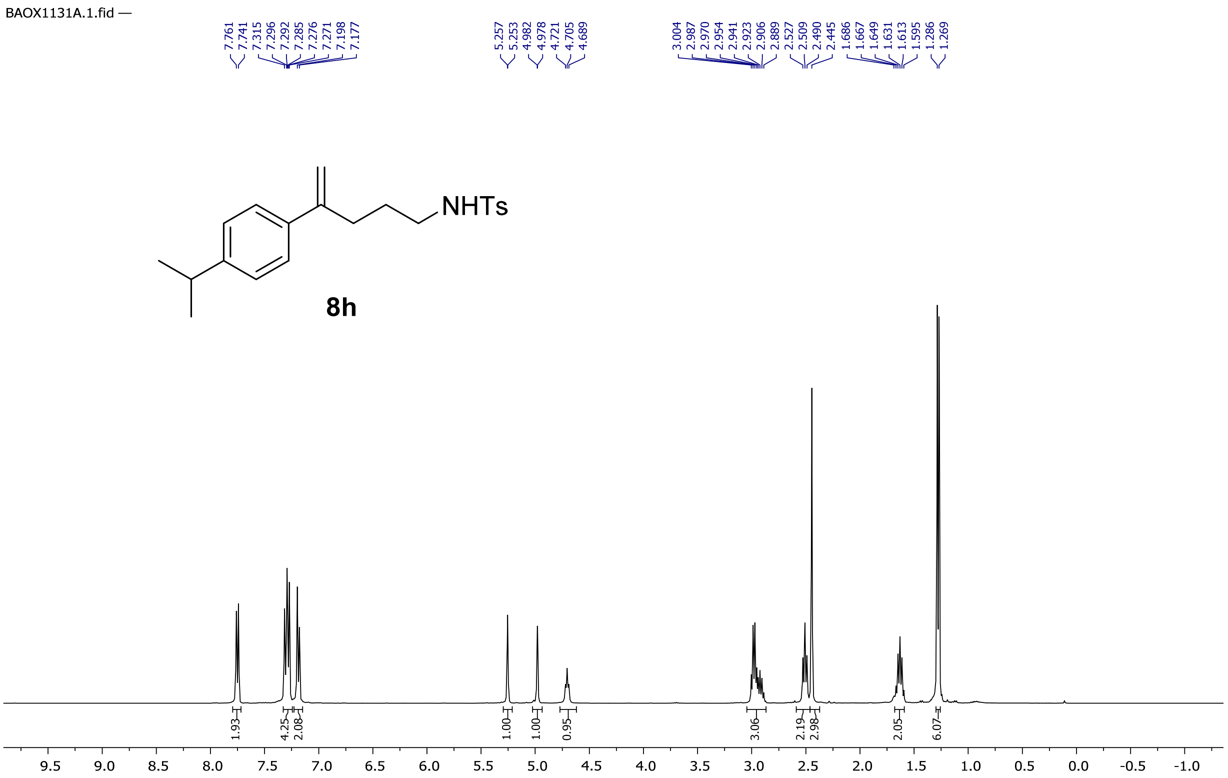

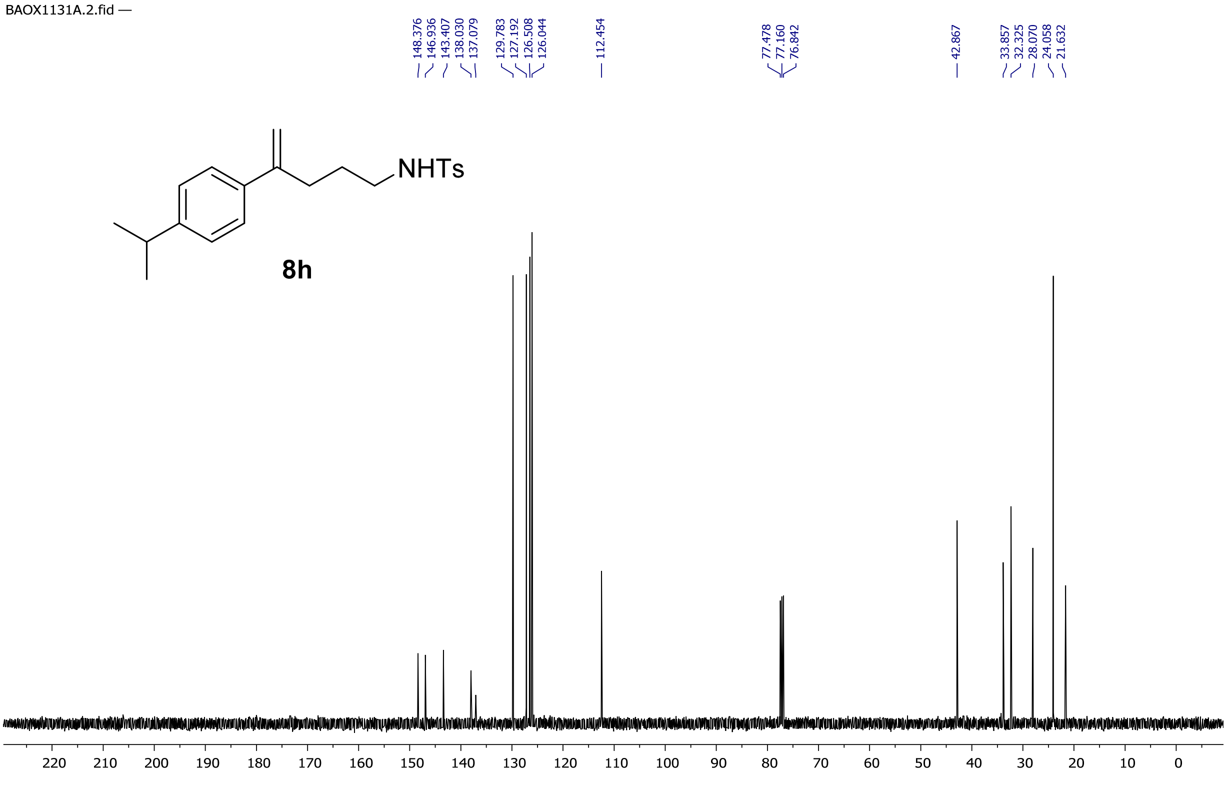

Supplementary Figure 14. ¹H and ¹³C NMR spectra of 8h

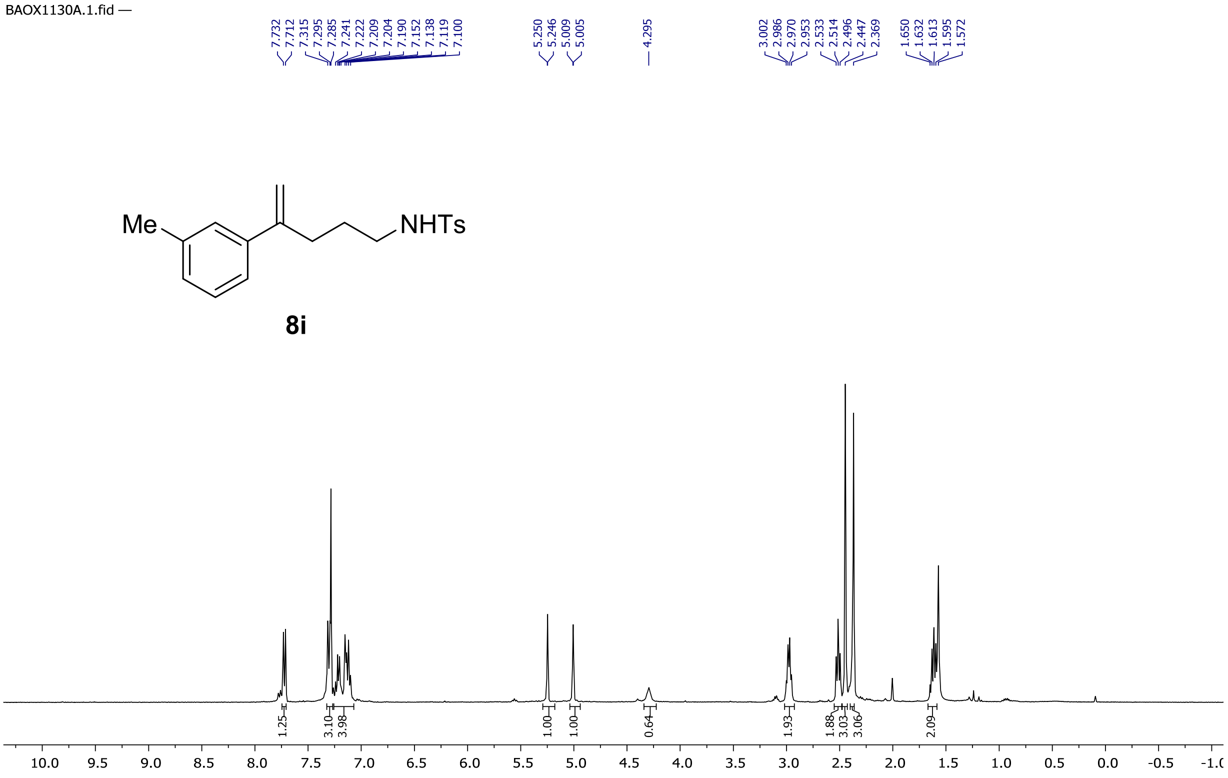

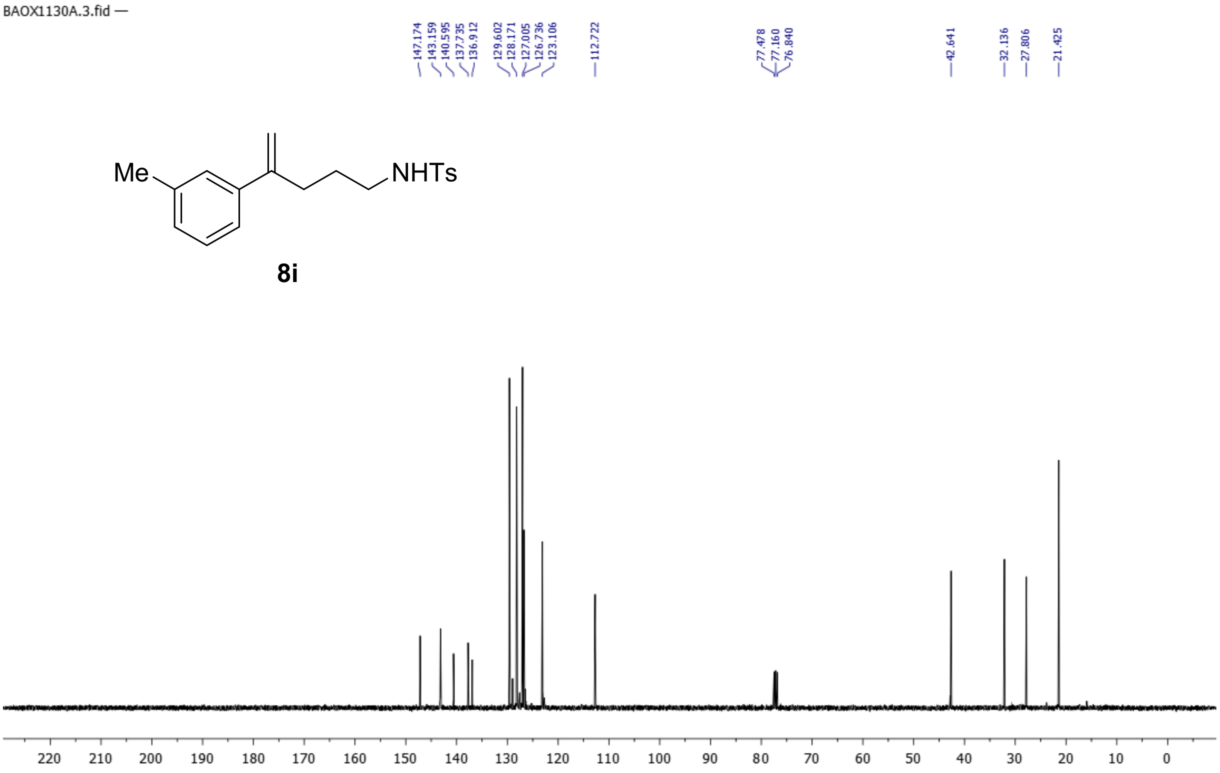

Supplementary Figure 15. ^{1}H and ^{13}C NMR spectra of 8i

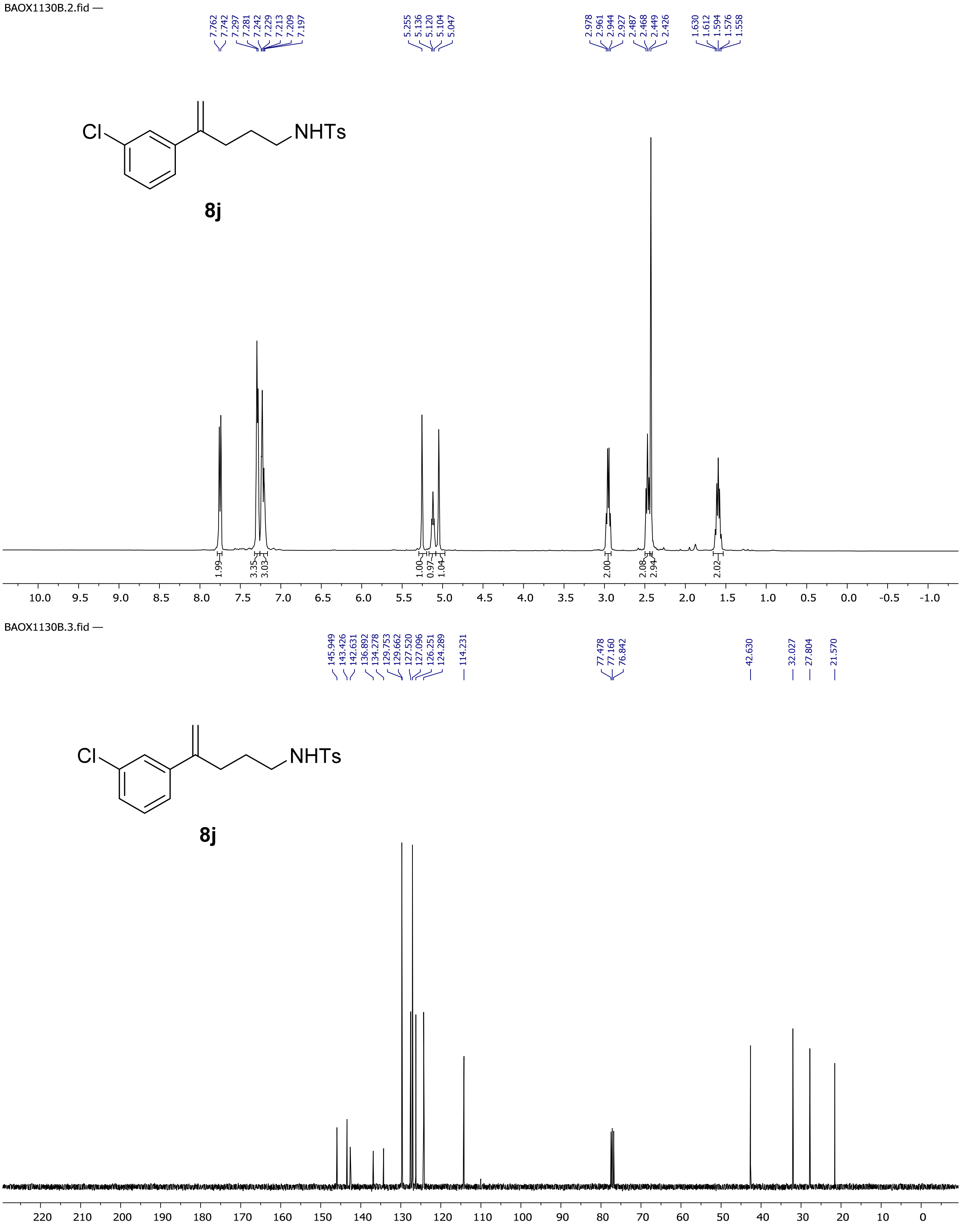

Supplementary Figure 16. ^{1}H and ^{13}C NMR spectra of 8j

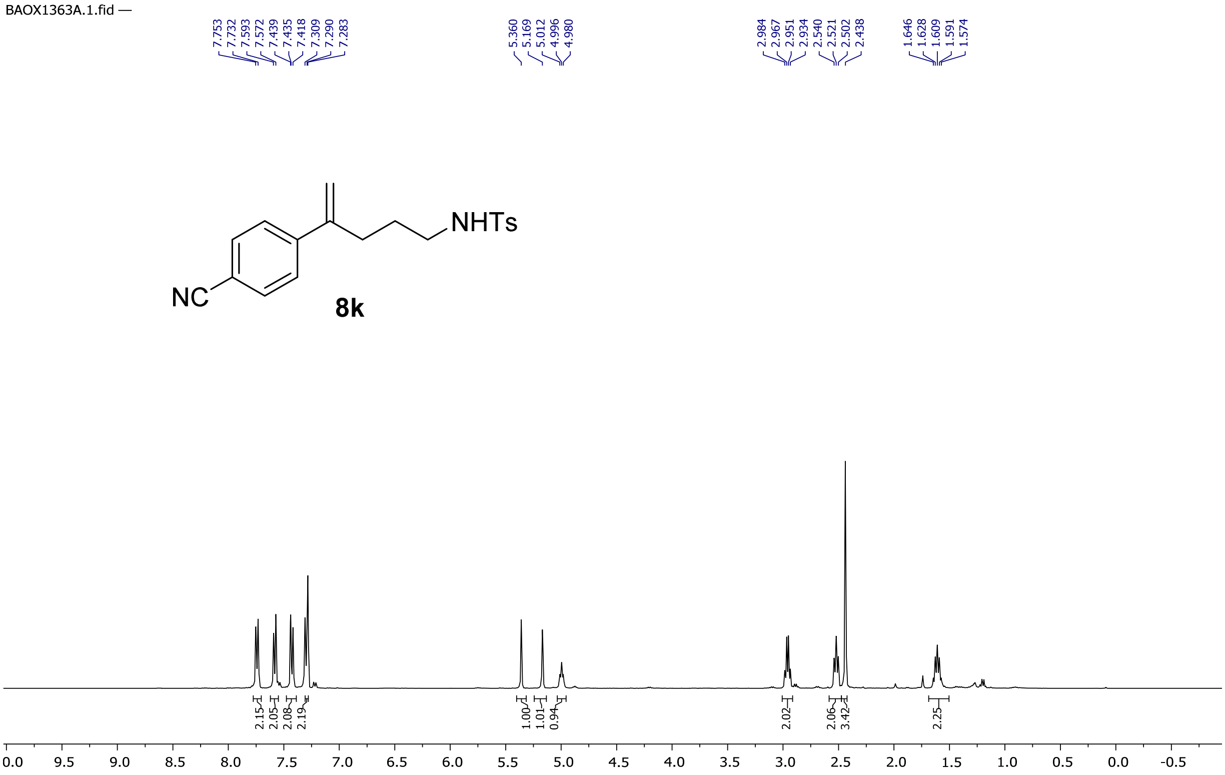

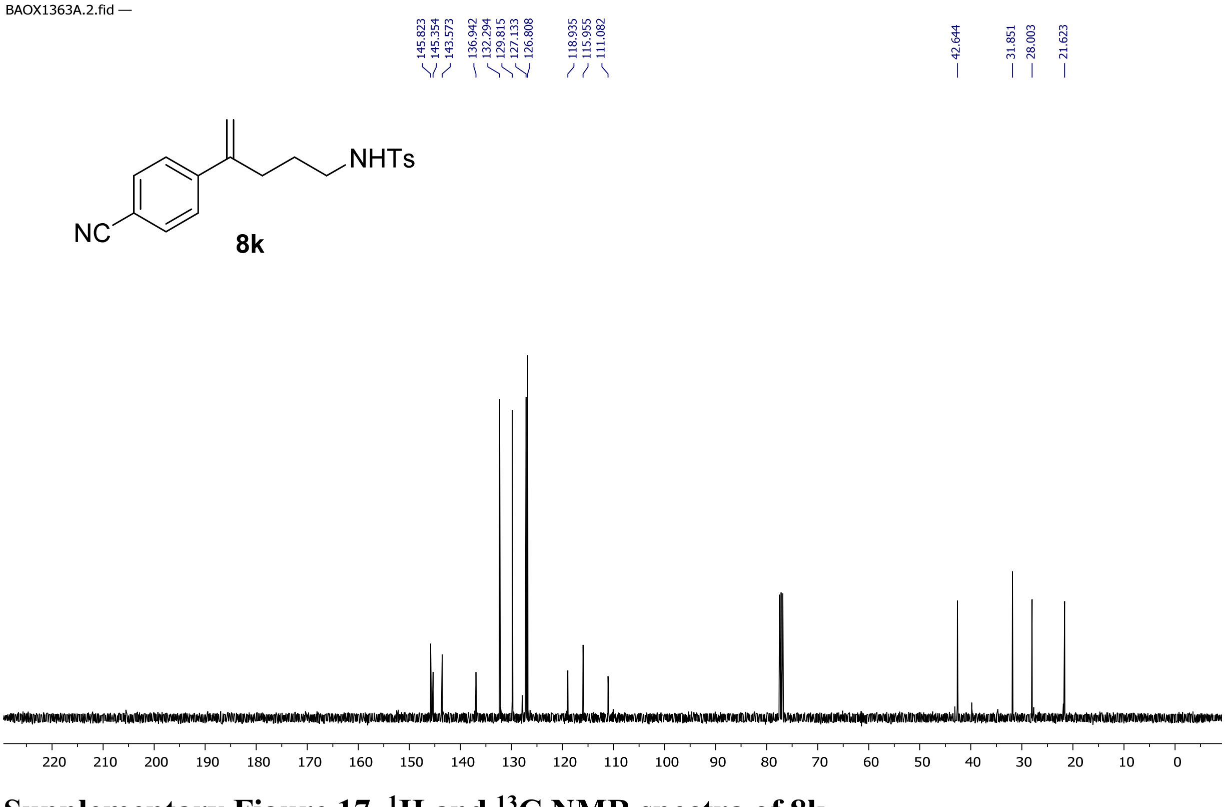

Supplementary Figure 17. ^{1}H and ^{13}C NMR spectra of 8k

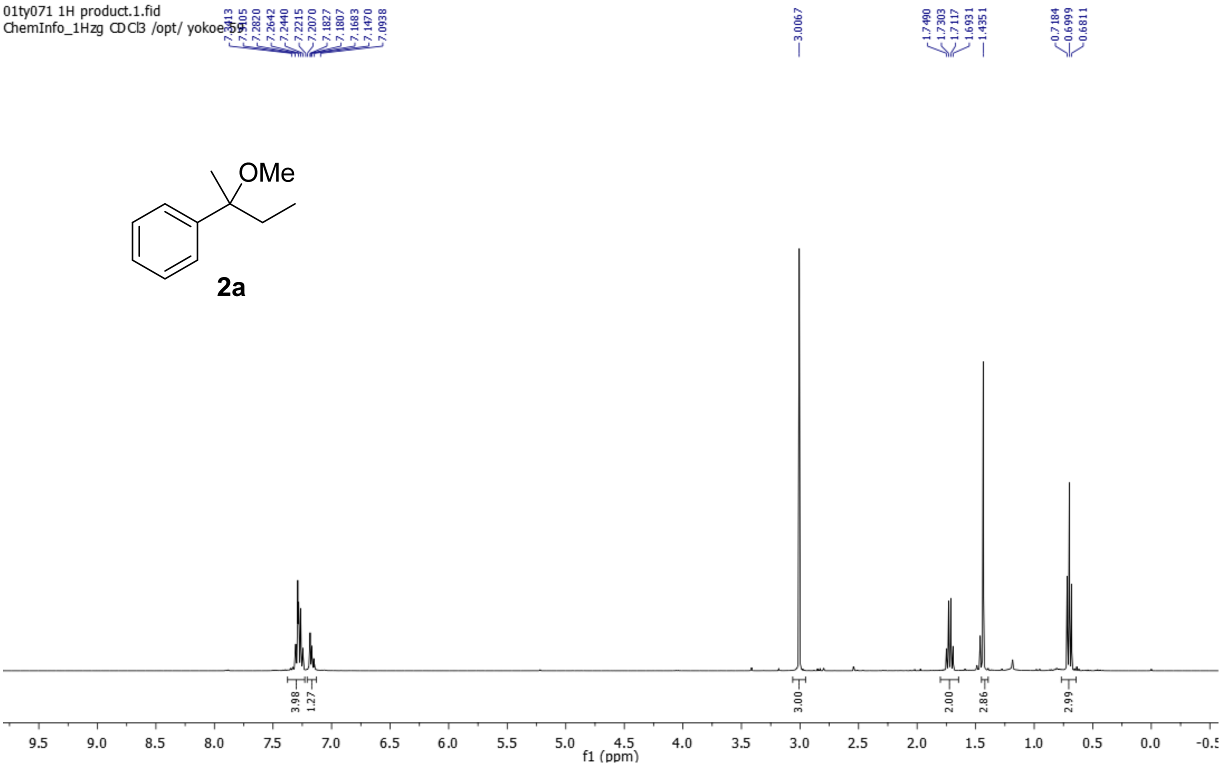

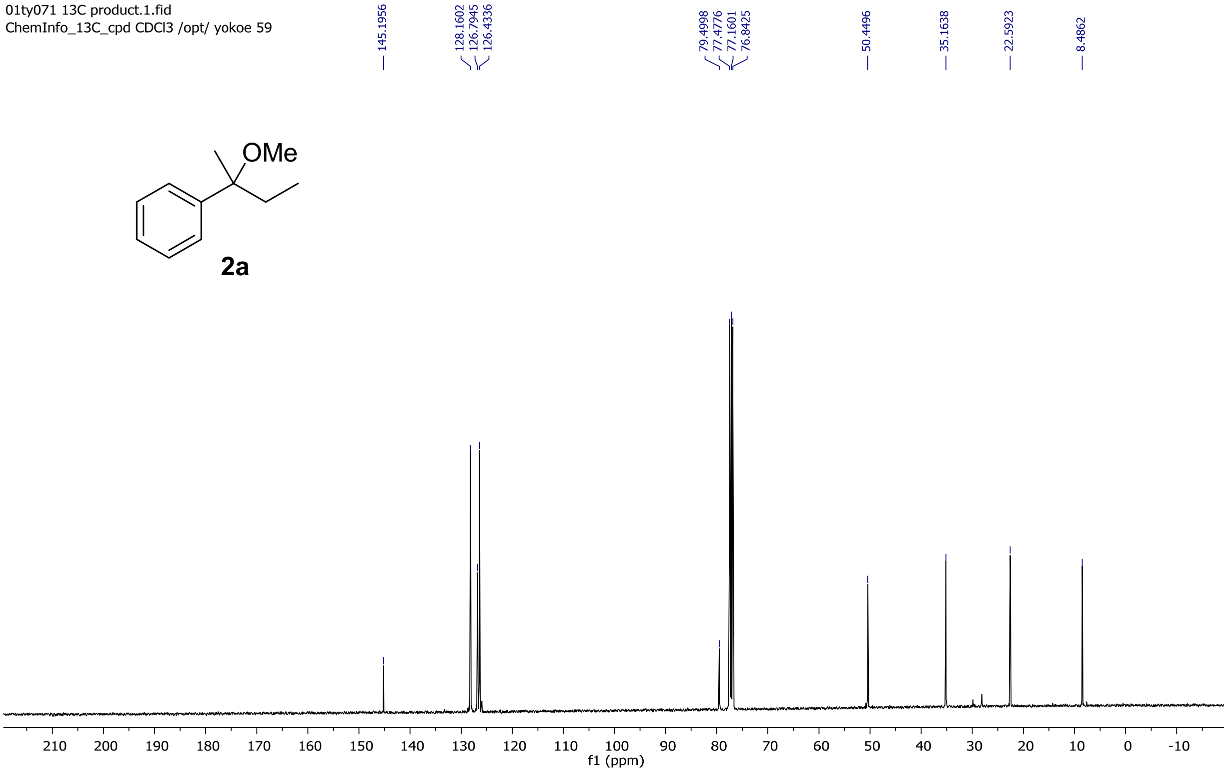

Supplementary Figure 18. ^{1}H and ^{13}C NMR spectra of 2a

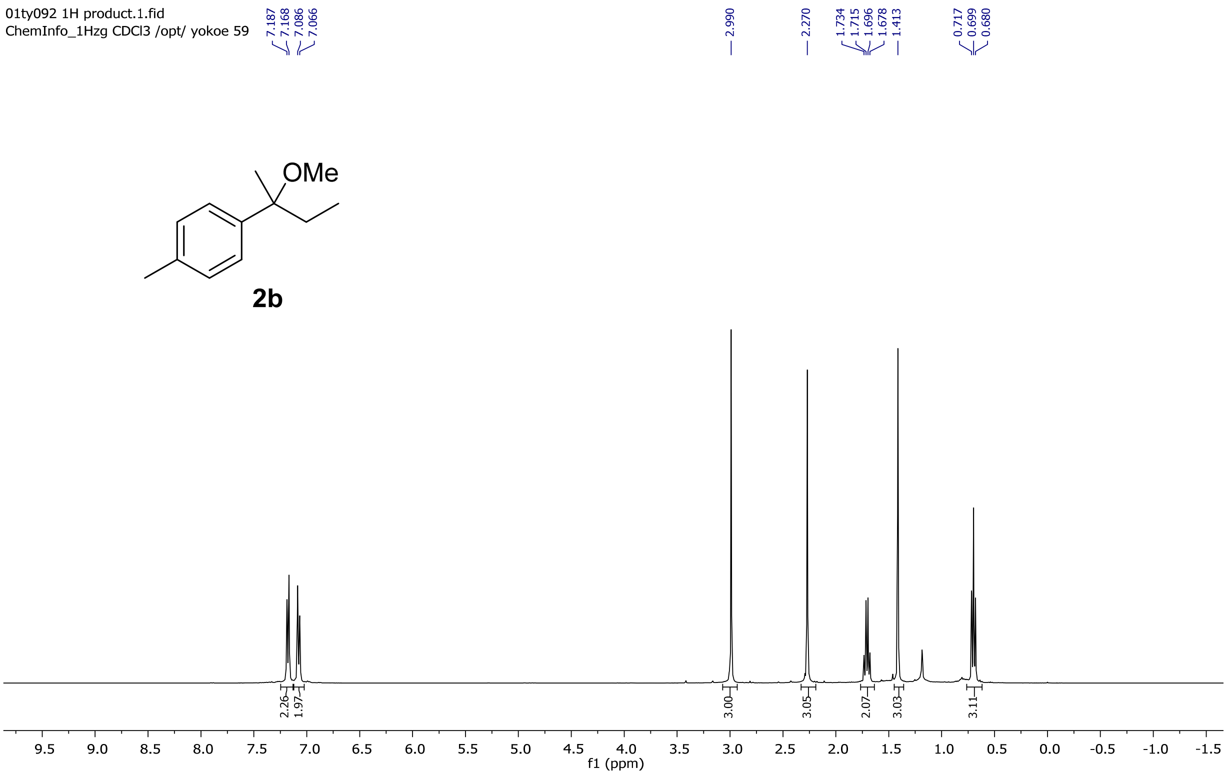

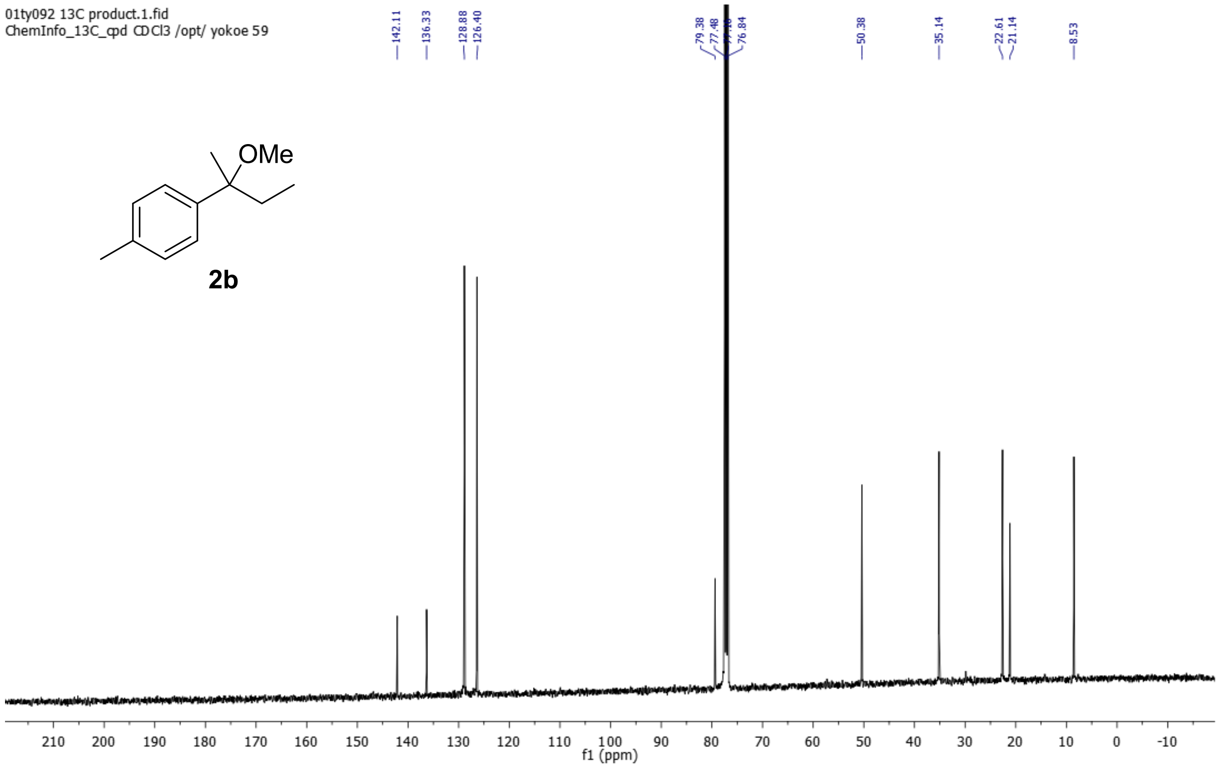

Supplementary Figure 19. ^{1}H and ^{13}C NMR spectra of 2b

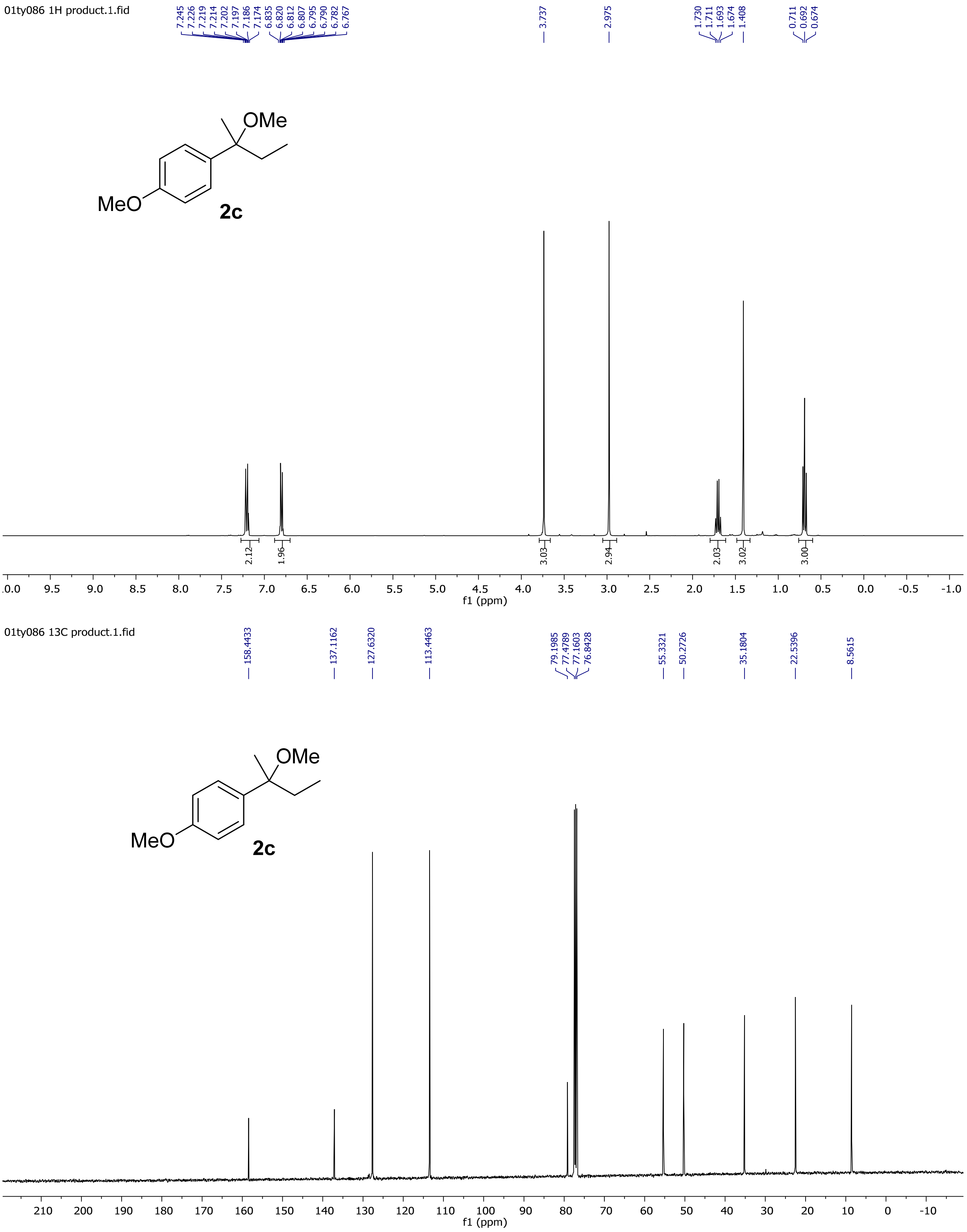

Supplementary Figure 20. ^{1}H and ^{13}C NMR spectra of 2c

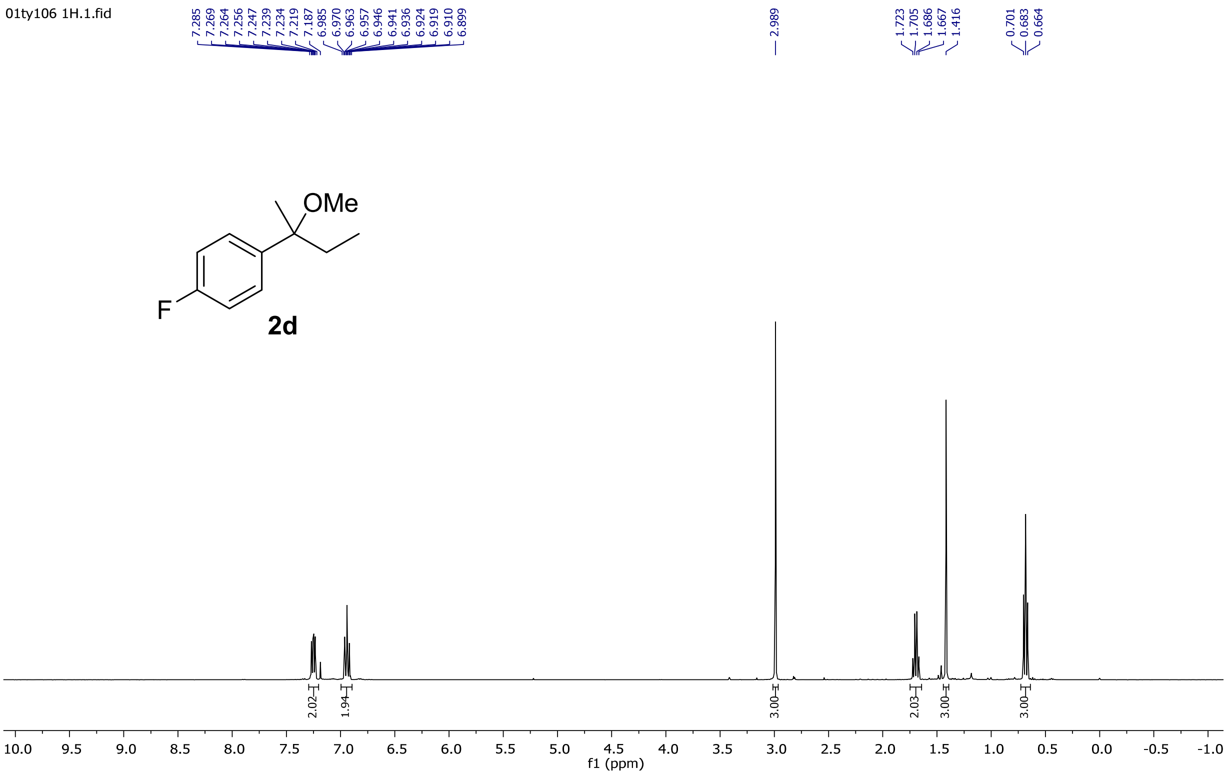

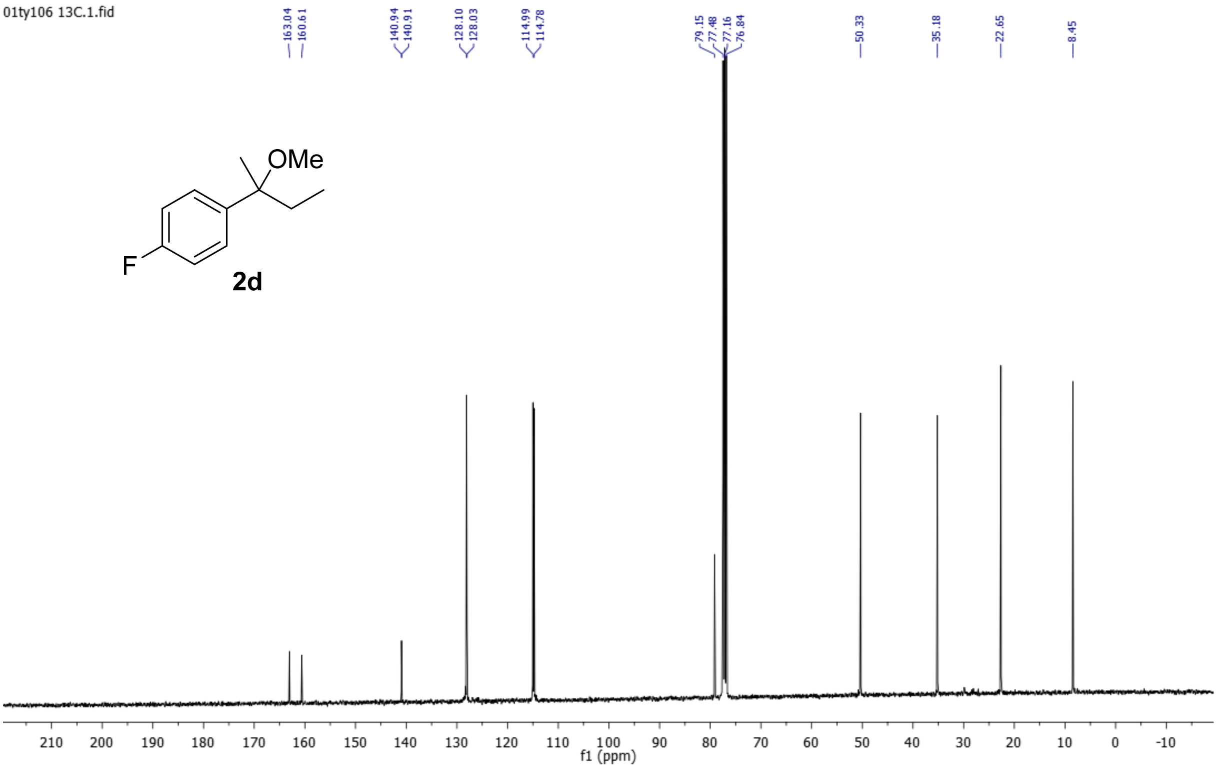

Supplementary Figure 21. ^{1}H and ^{13}C NMR spectra of 2d

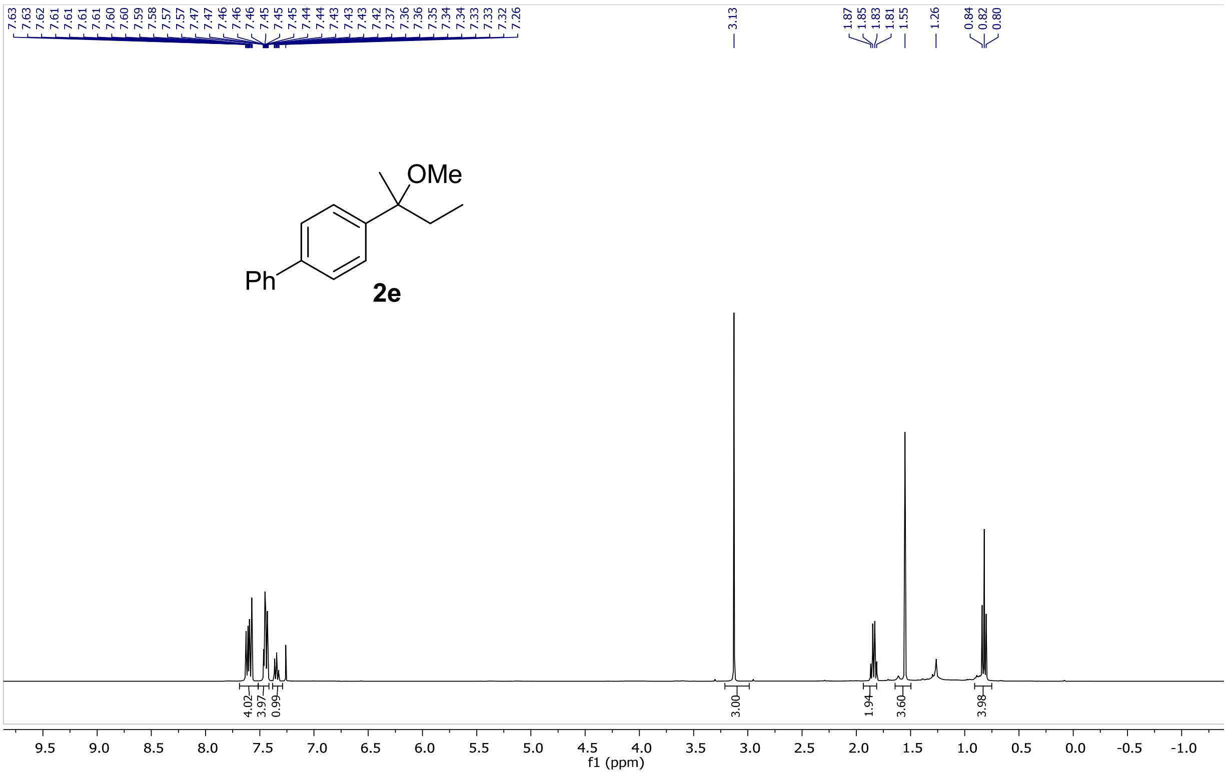

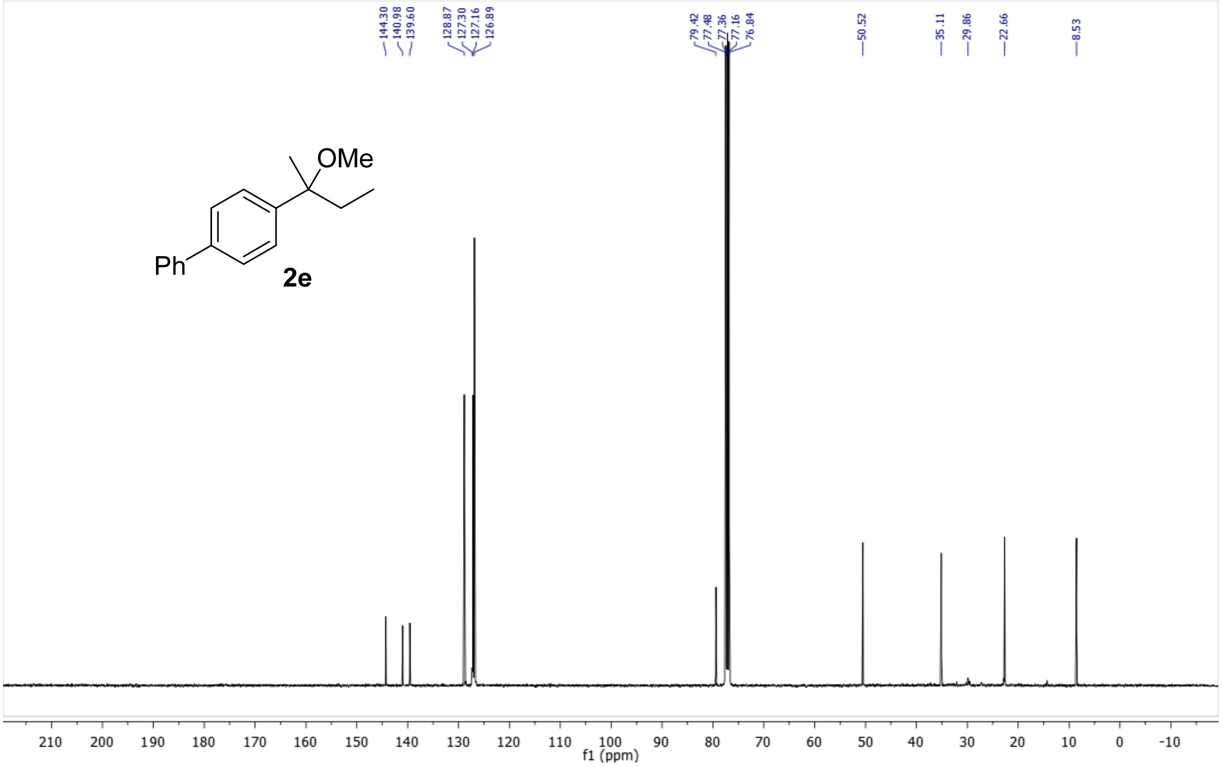

Supplementary Figure 22. ^{1}H and ^{13}C NMR spectra of 2e

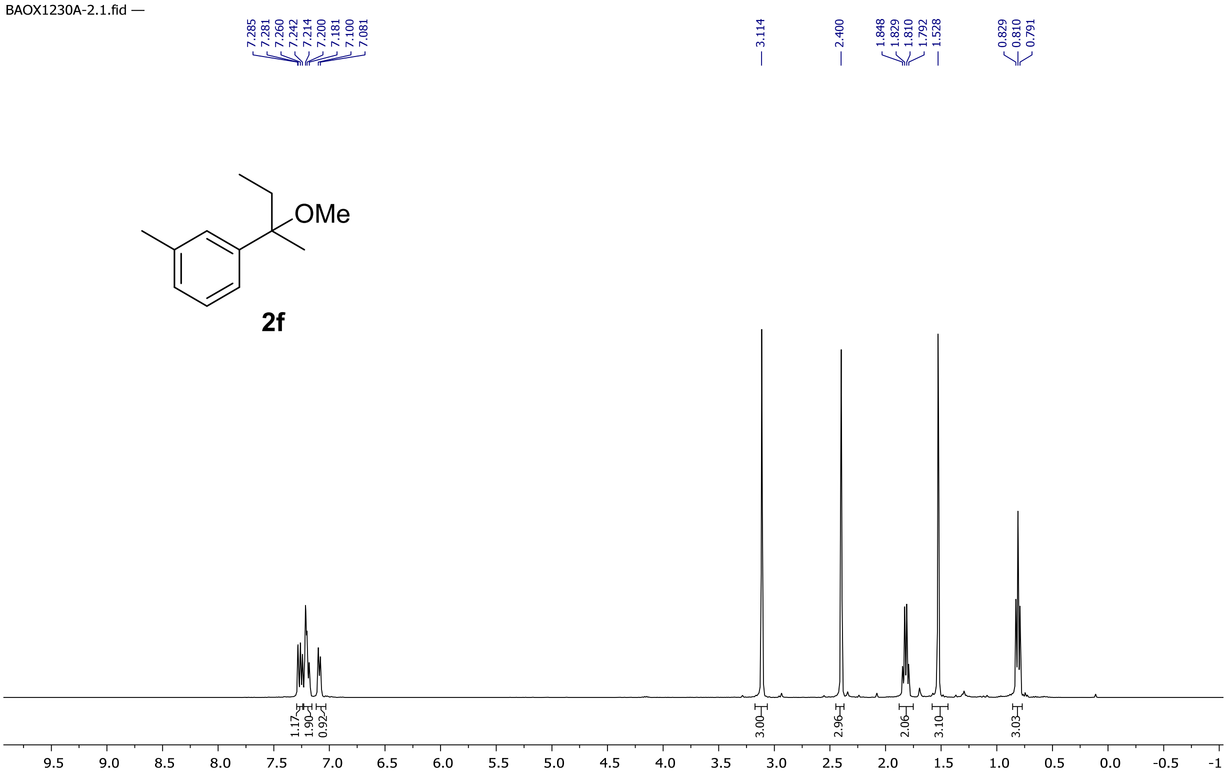

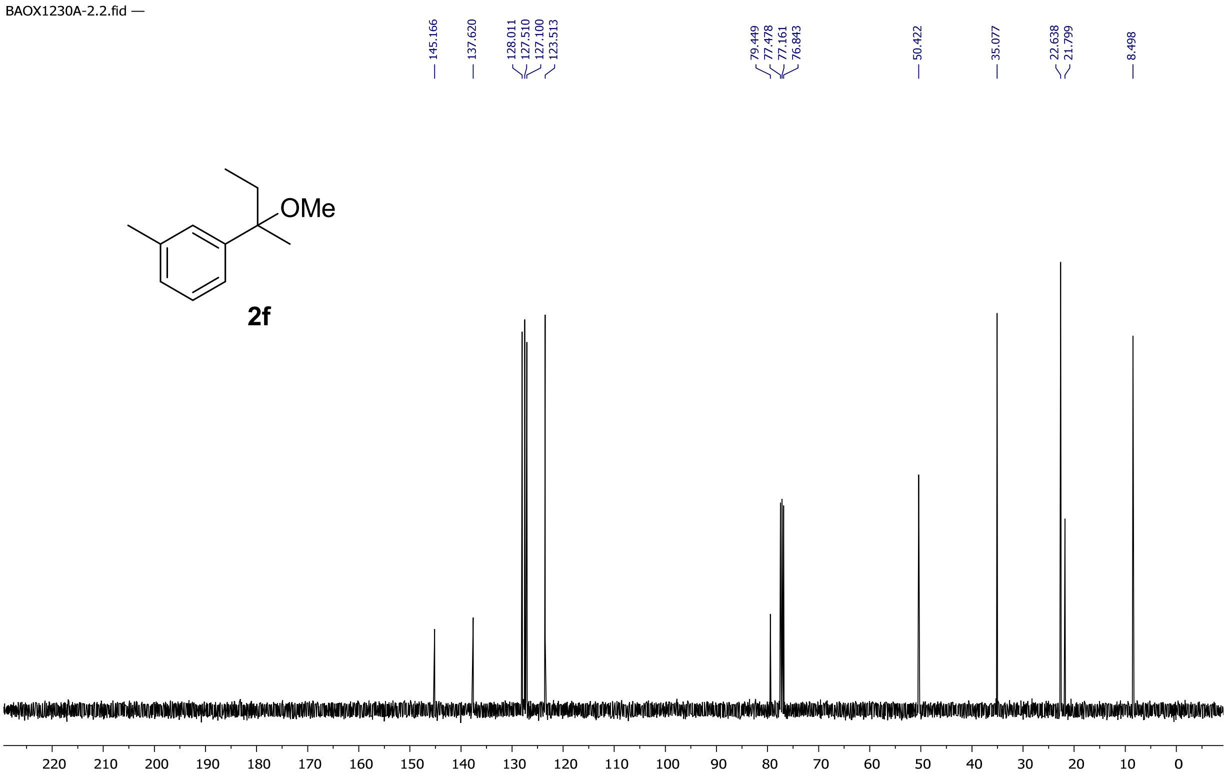

Supplementary Figure 23. ^{1}H and ^{13}C NMR spectra of 2f

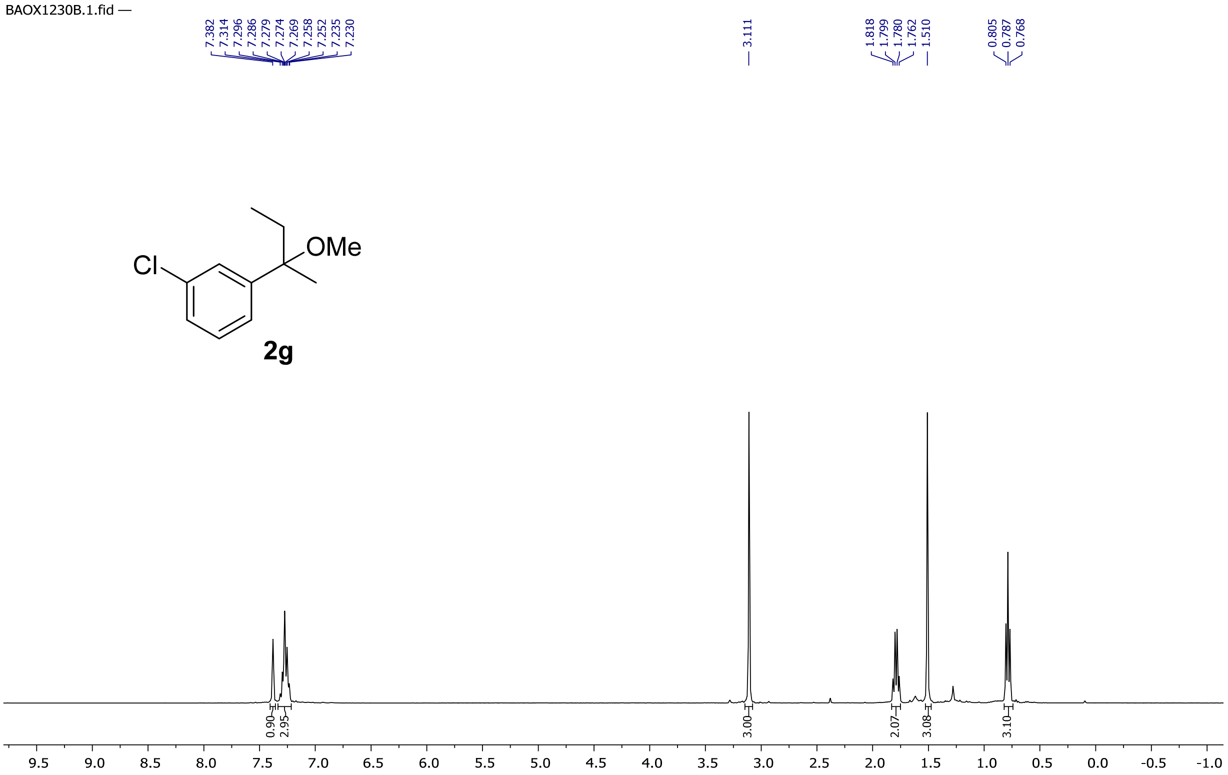

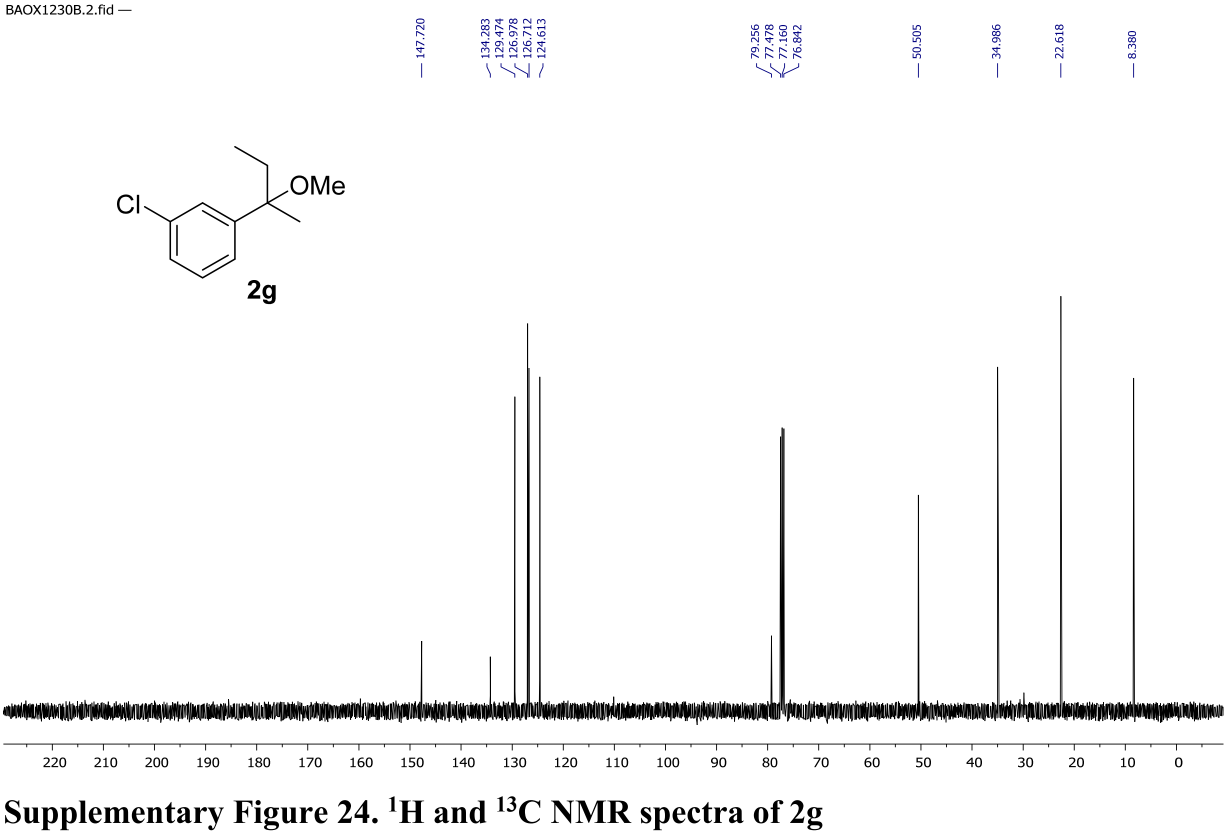

Supplementary Figure 24. ^{1}H and ^{13}C NMR spectra of 2g

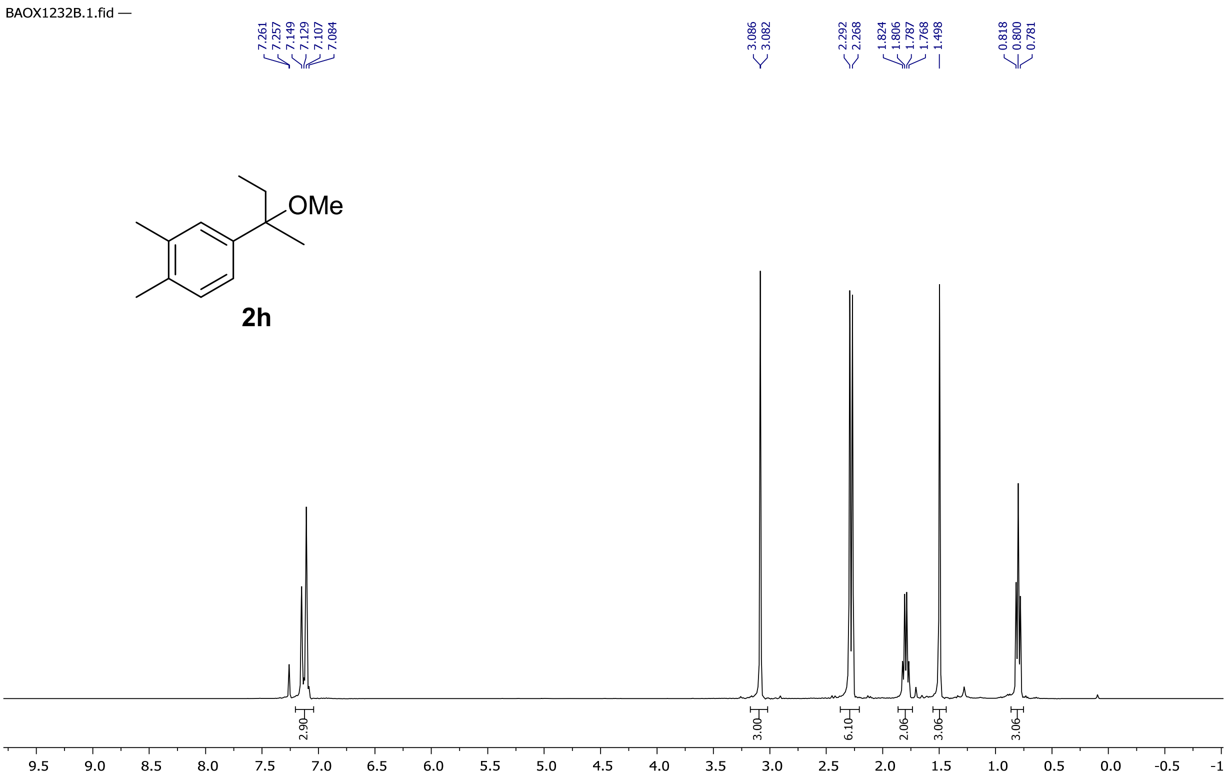

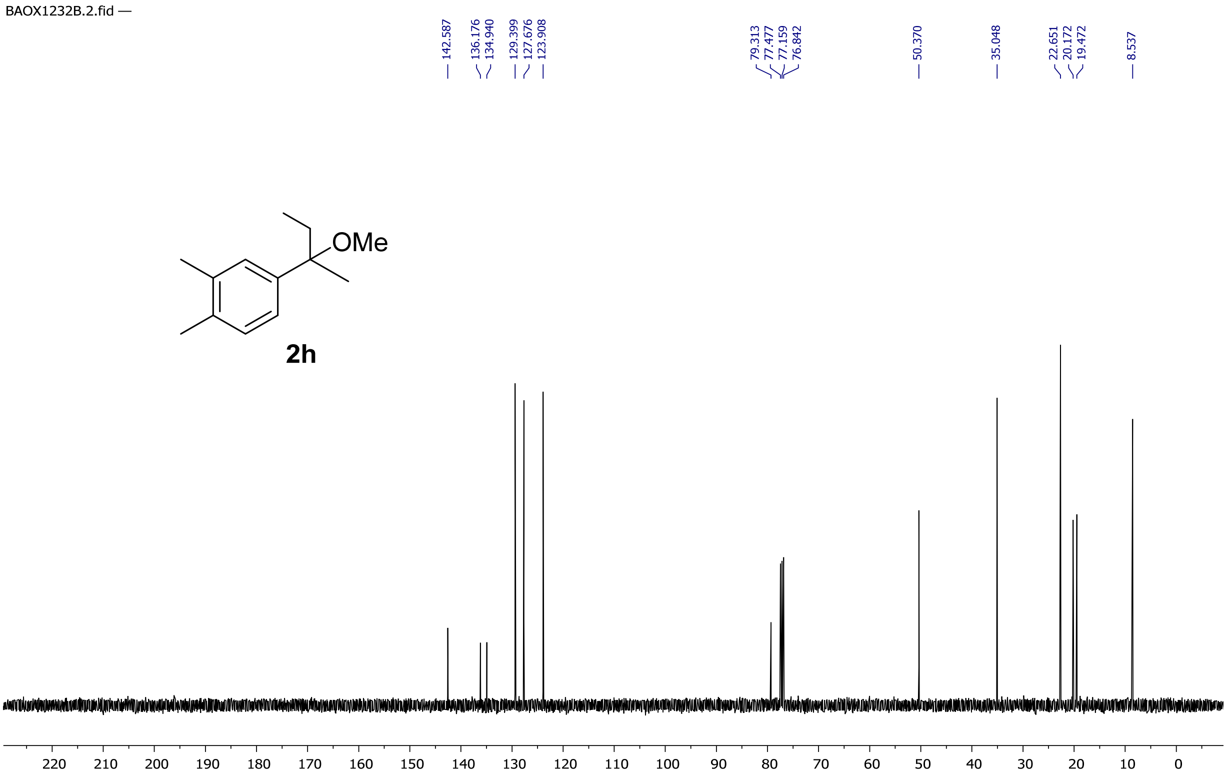

Supplementary Figure 25. ^{1}H and ^{13}C NMR spectra of 2h

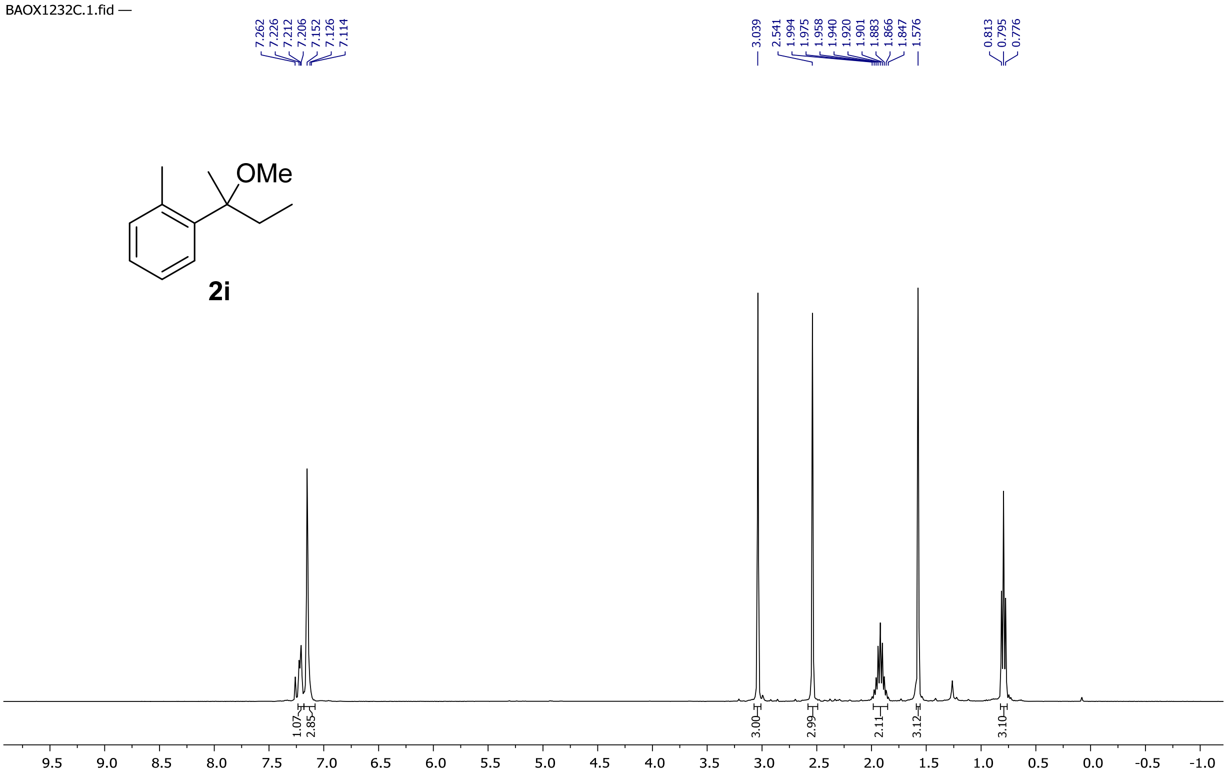

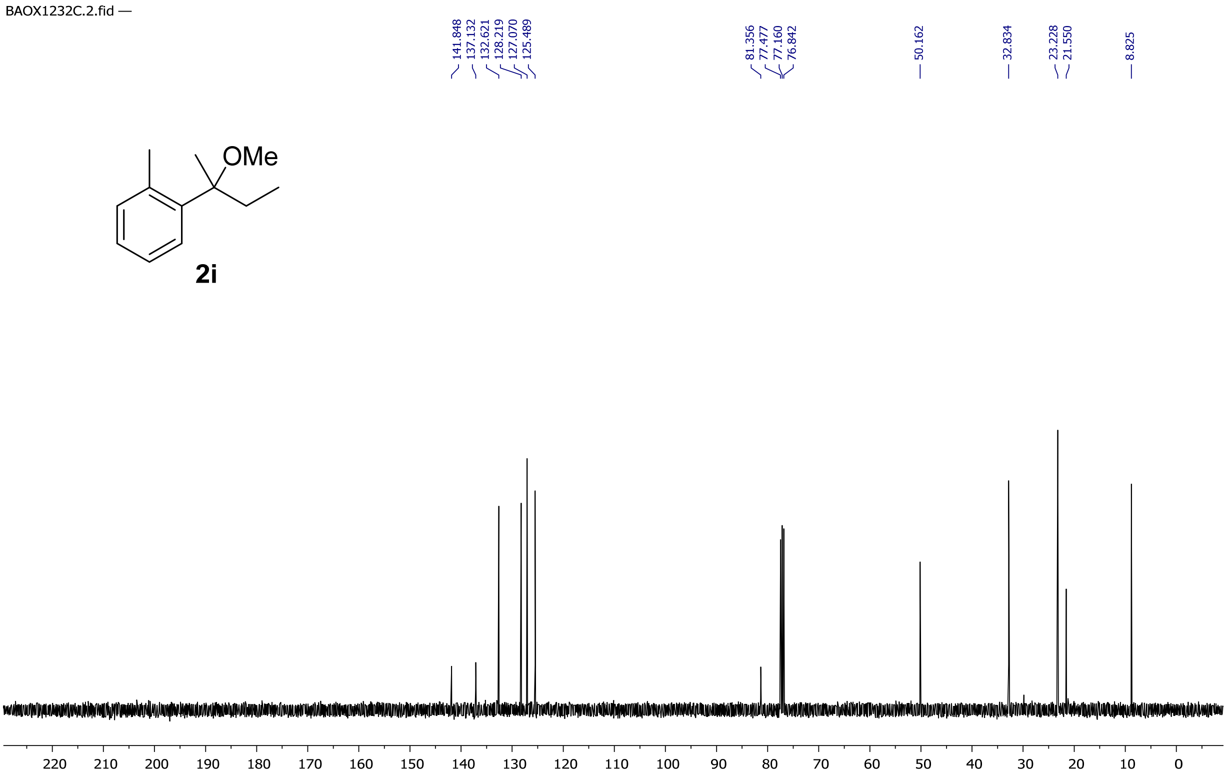

Supplementary Figure 26. ^{1}H and ^{13}C NMR spectra of 2i

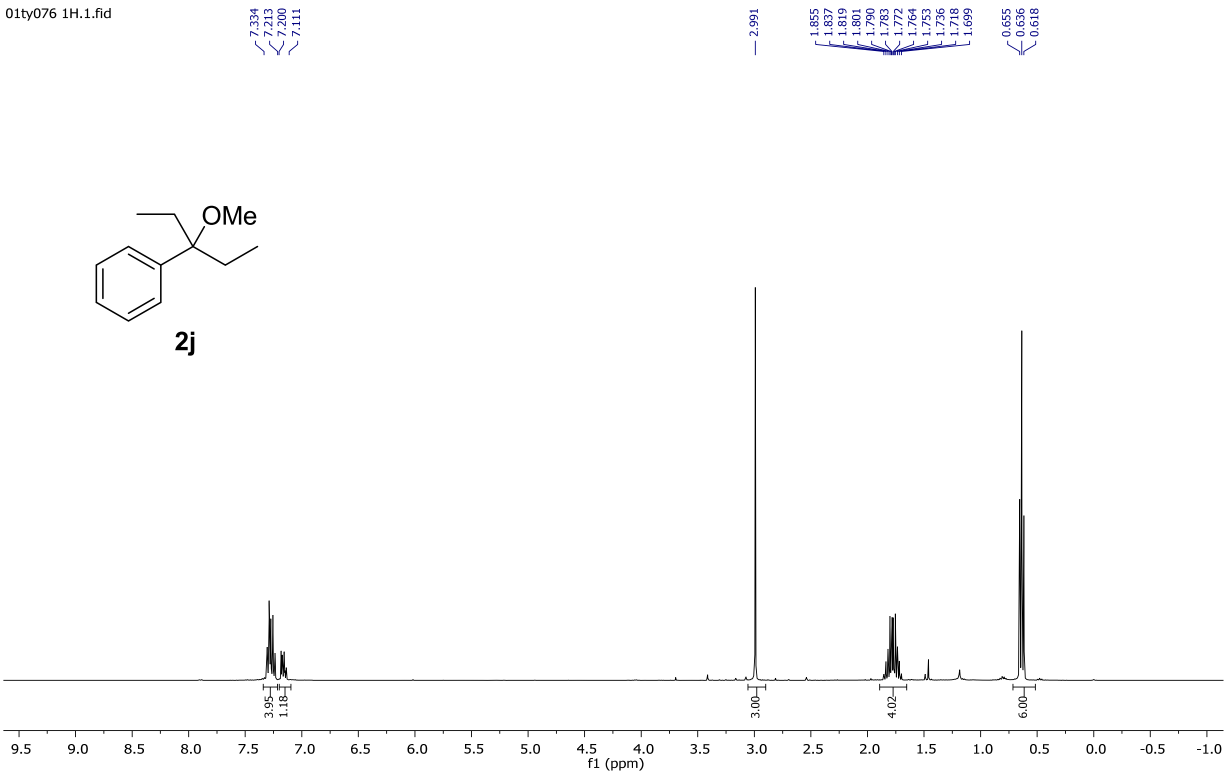

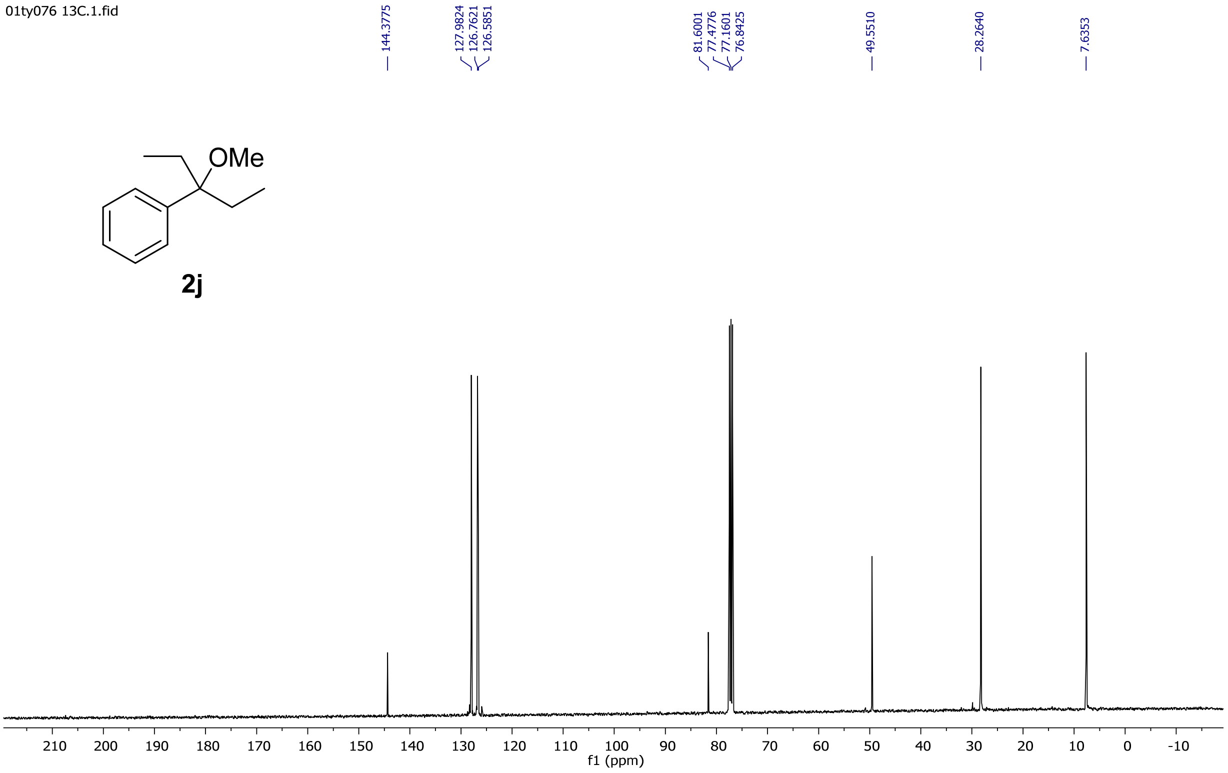

Supplementary Figure 27. ^{1}H and ^{13}C NMR spectra of 2j

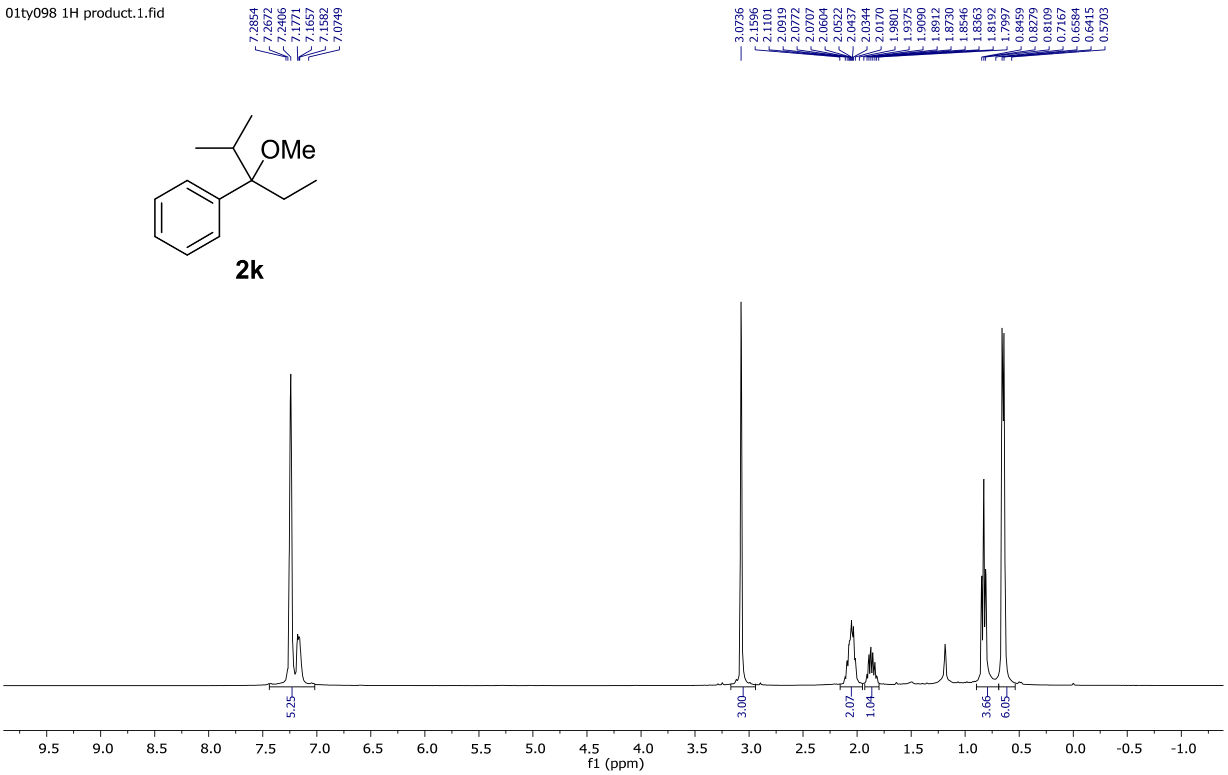

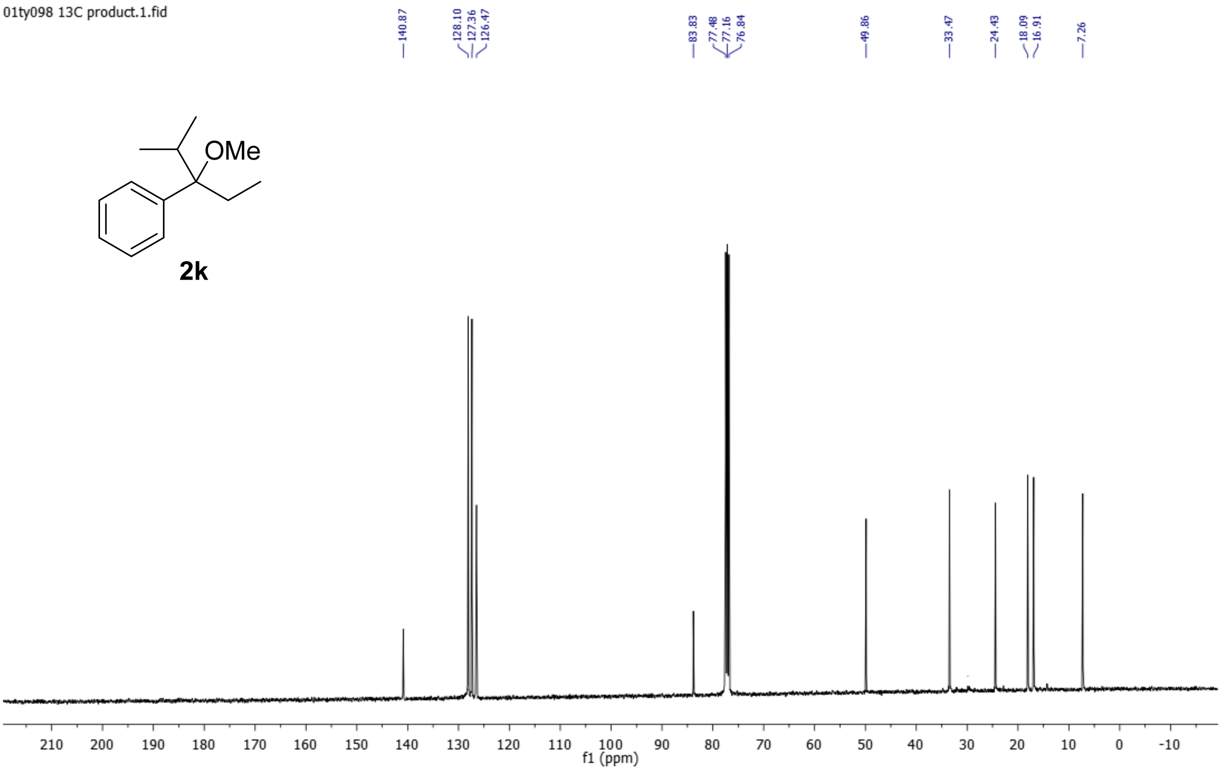

Supplementary Figure 28. ^{1}H and ^{13}C NMR spectra of 2k

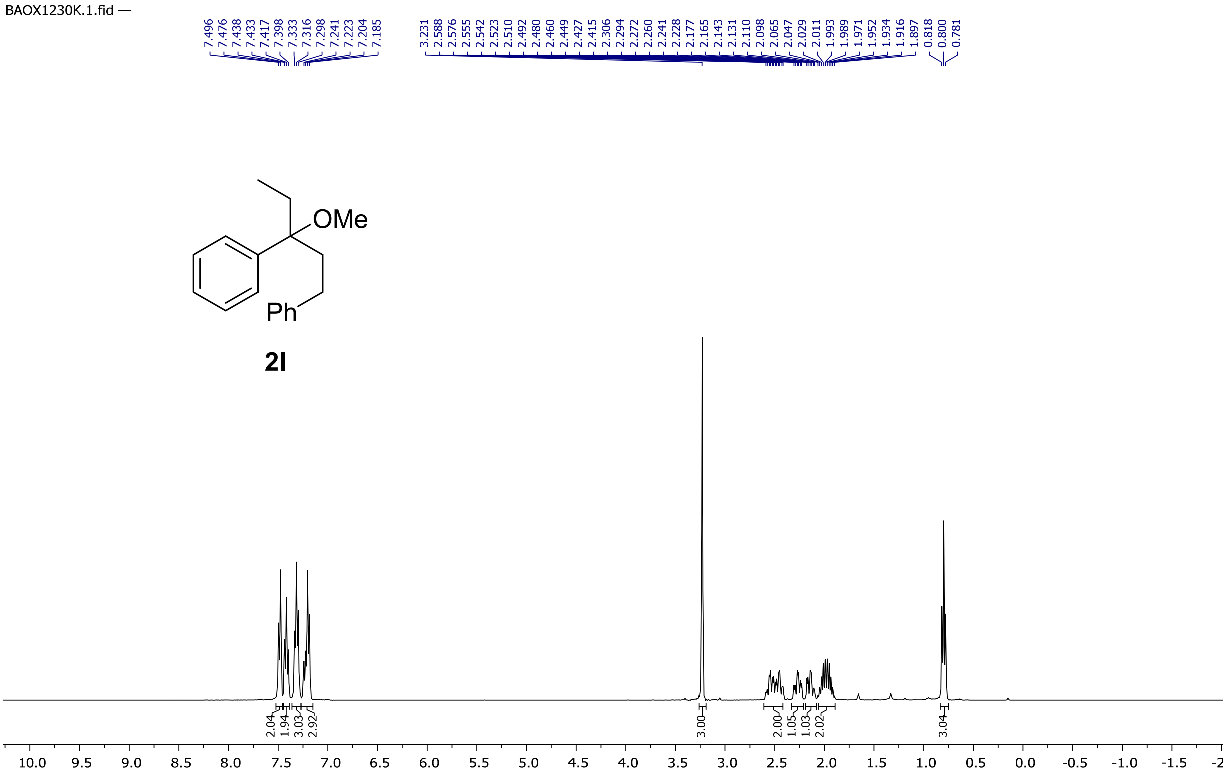

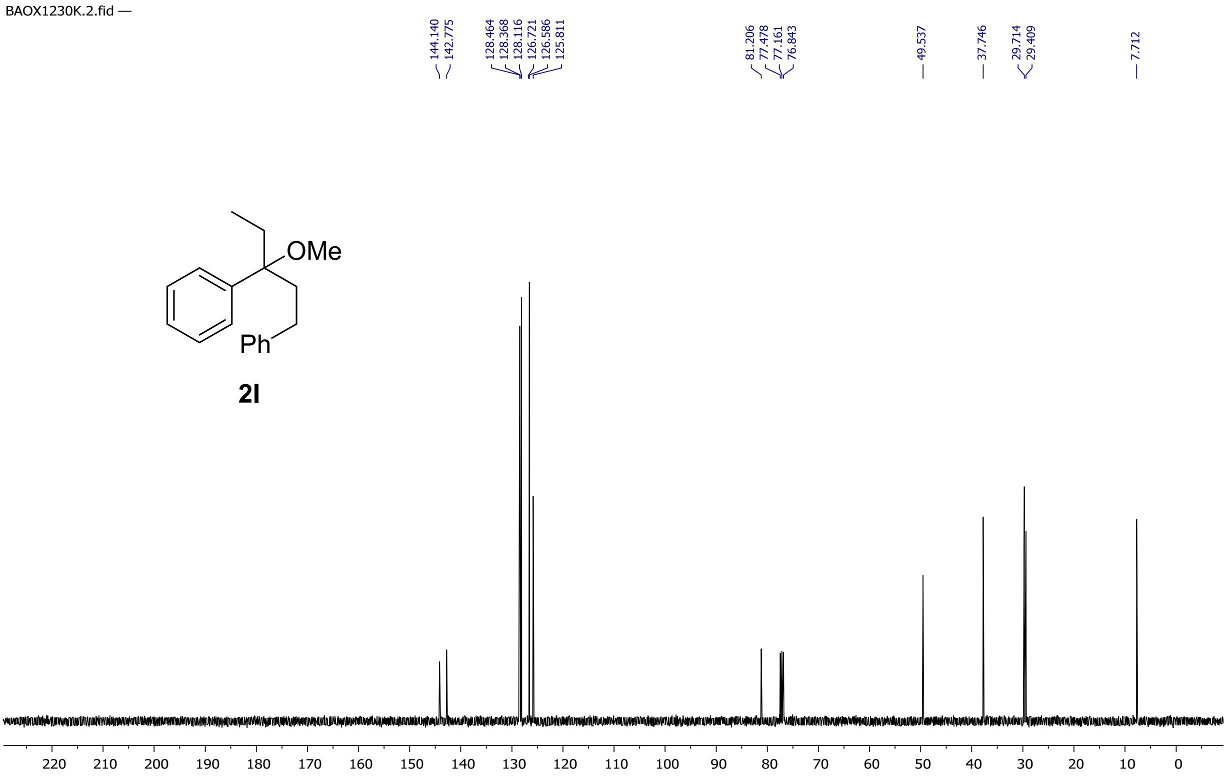

Supplementary Figure 29. ^{1}H and ^{13}C NMR spectra of 2l

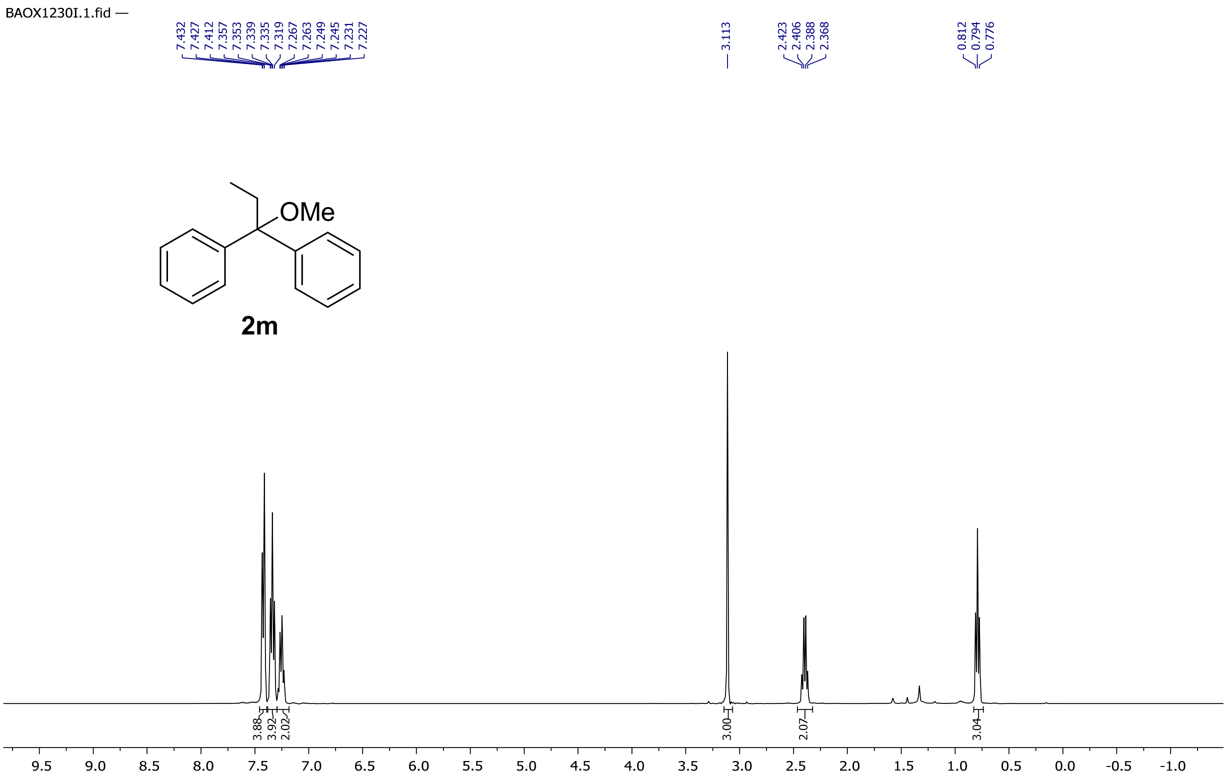

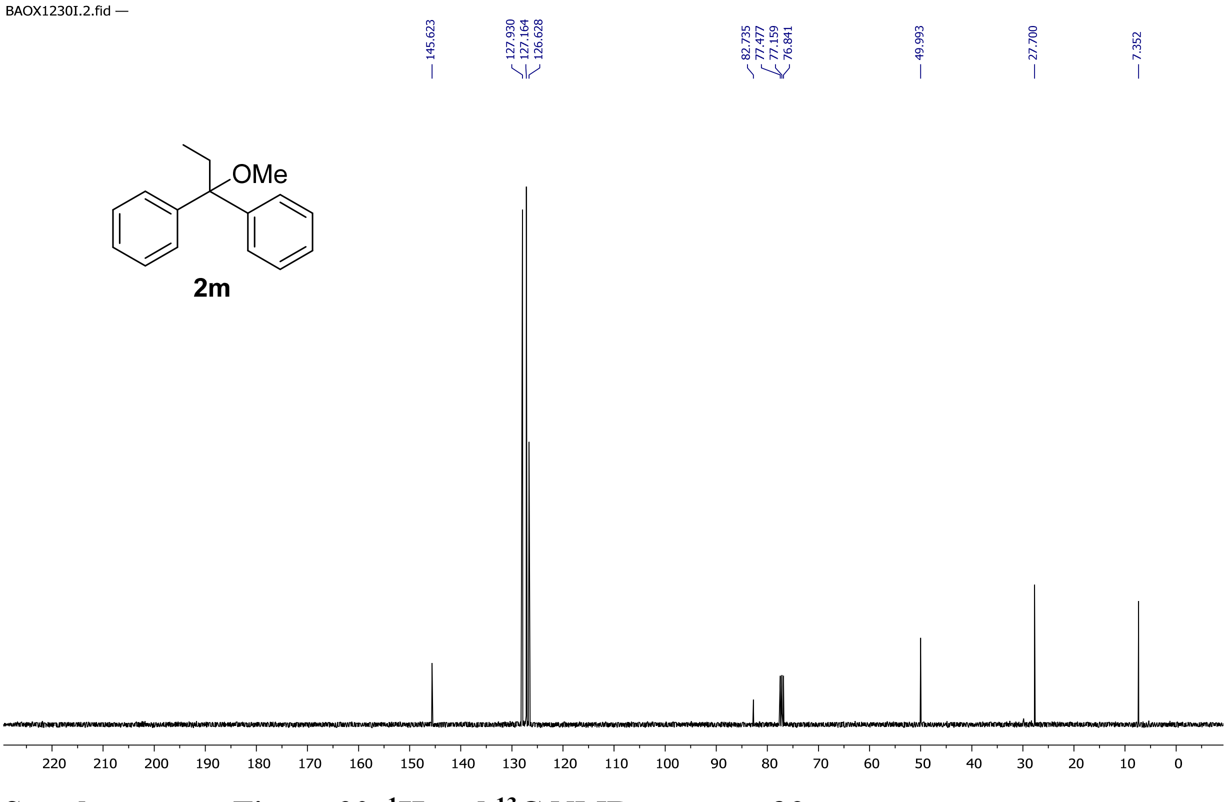

Supplementary Figure 30. ^{1}H **and** ^{13}C **NMR spectra of 2m**

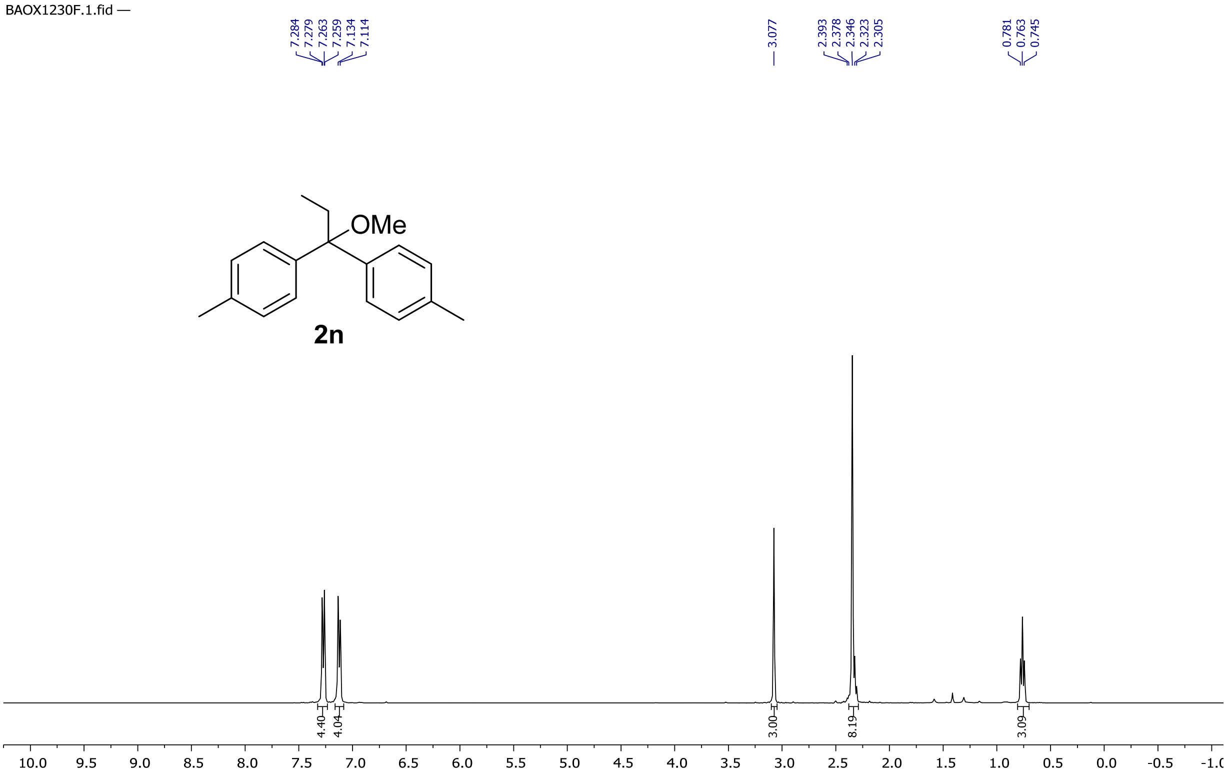

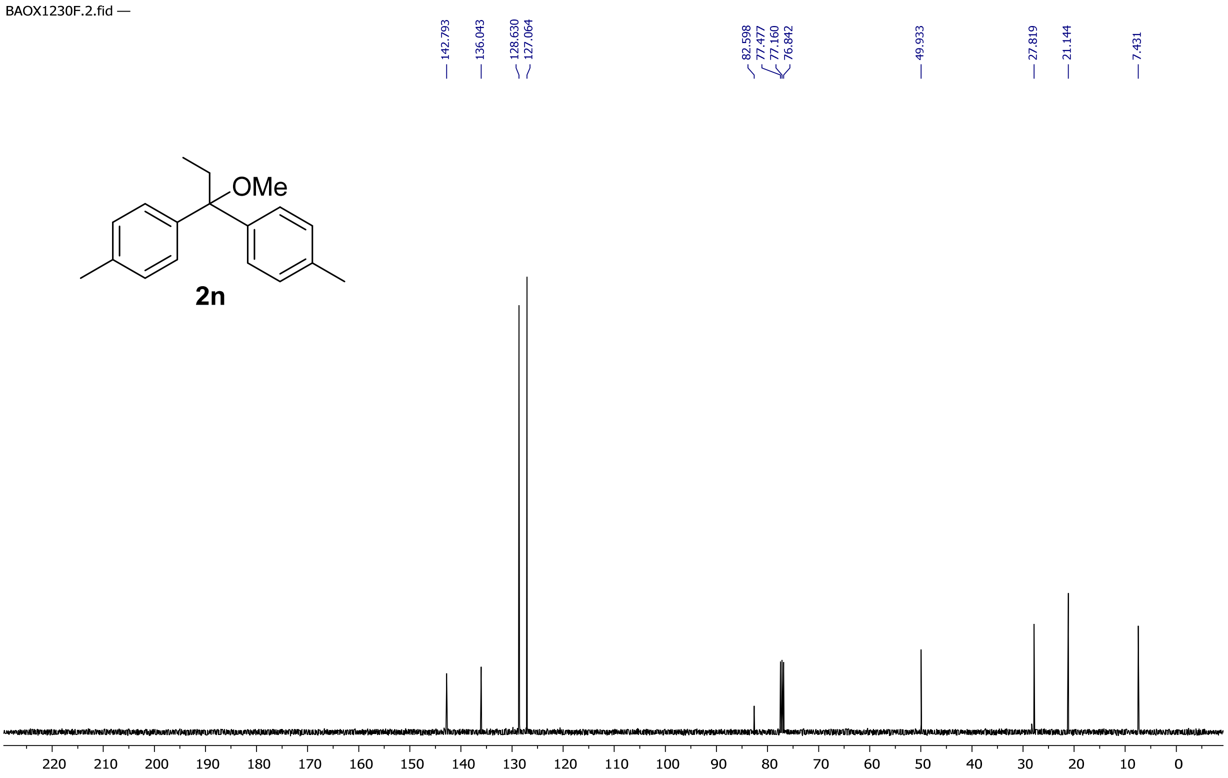

Supplementary Figure 31. ^{1}H and ^{13}C NMR spectra of 2n

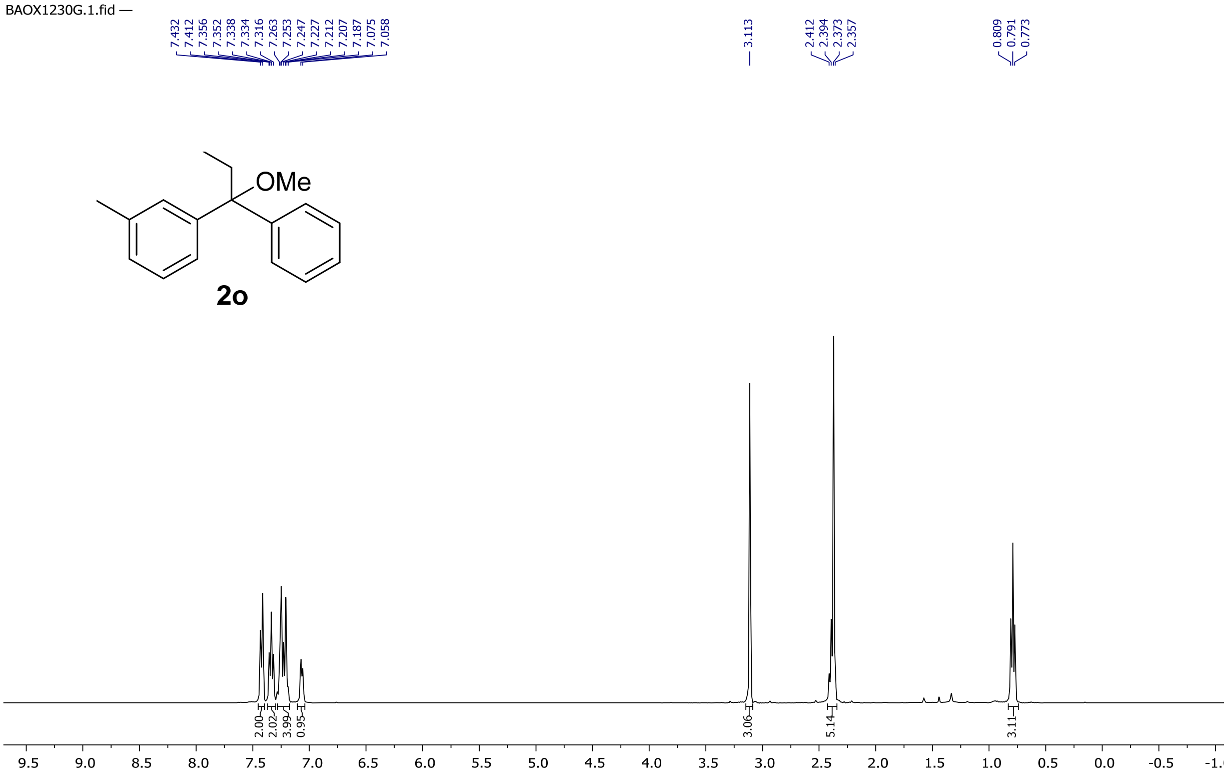

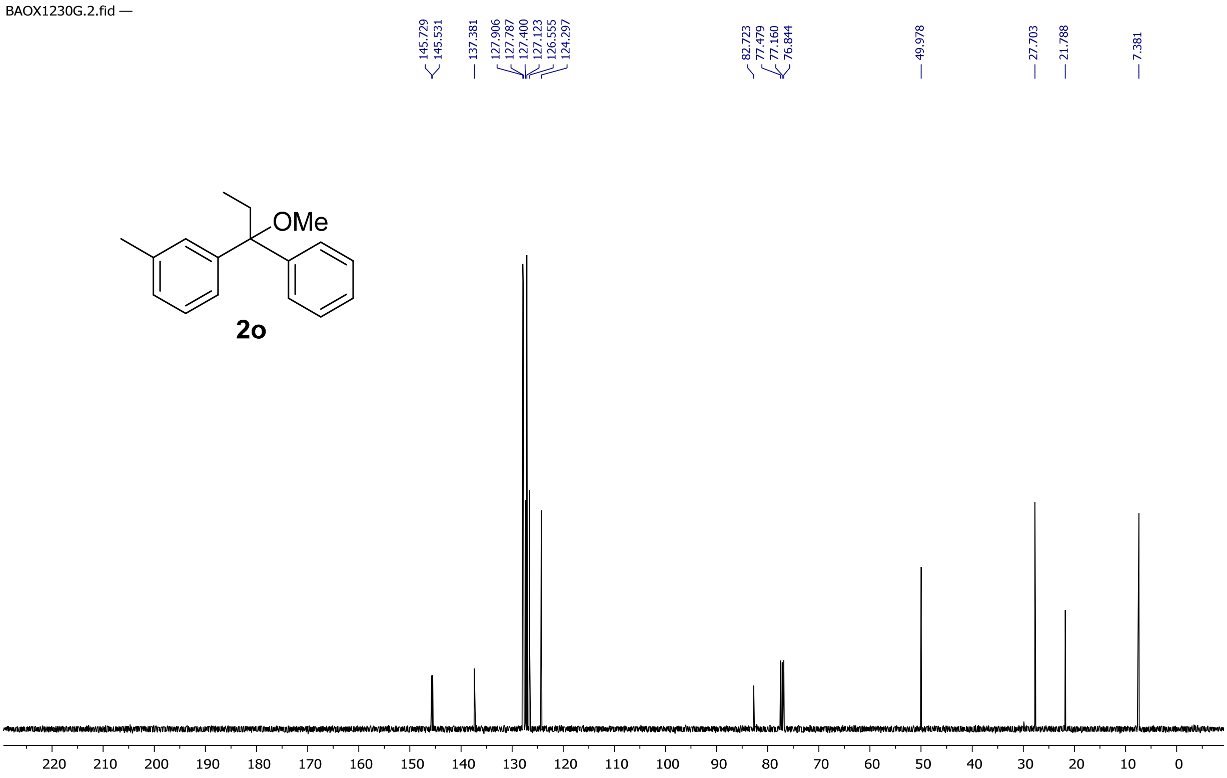

Supplementary Figure 32. ^{1}H and ^{13}C NMR spectra of 2o

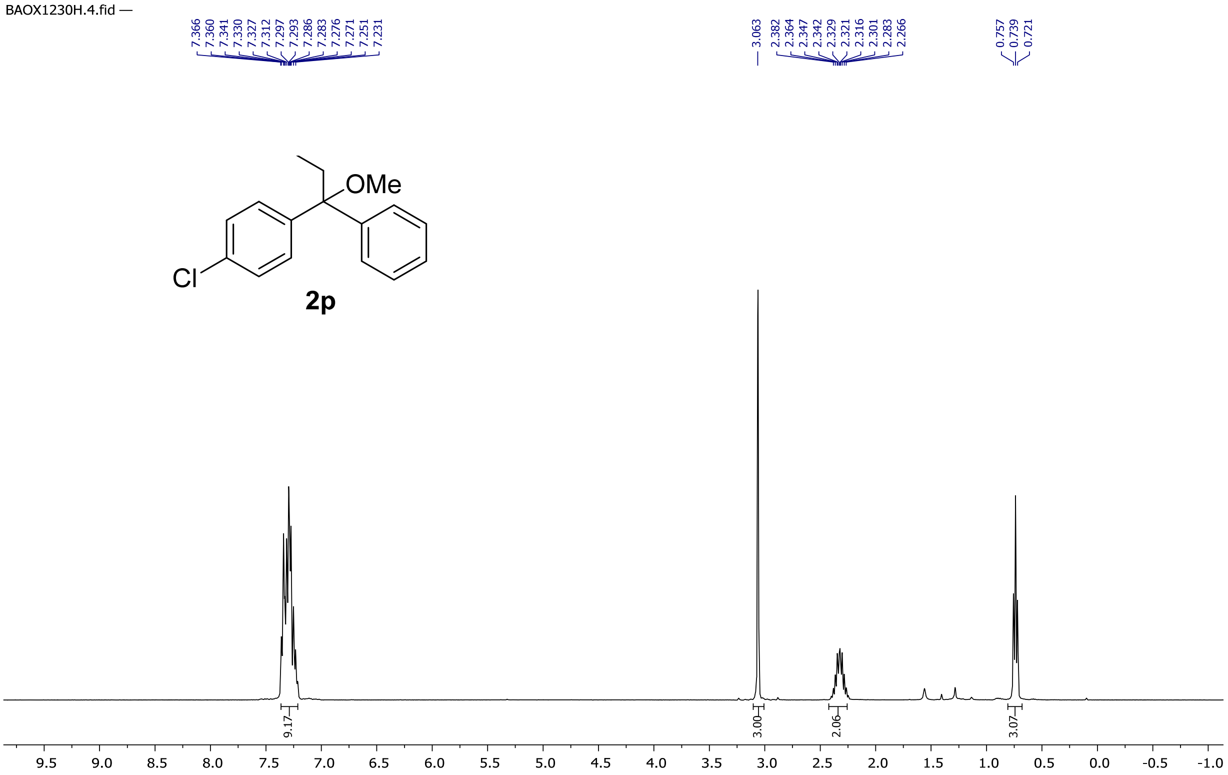

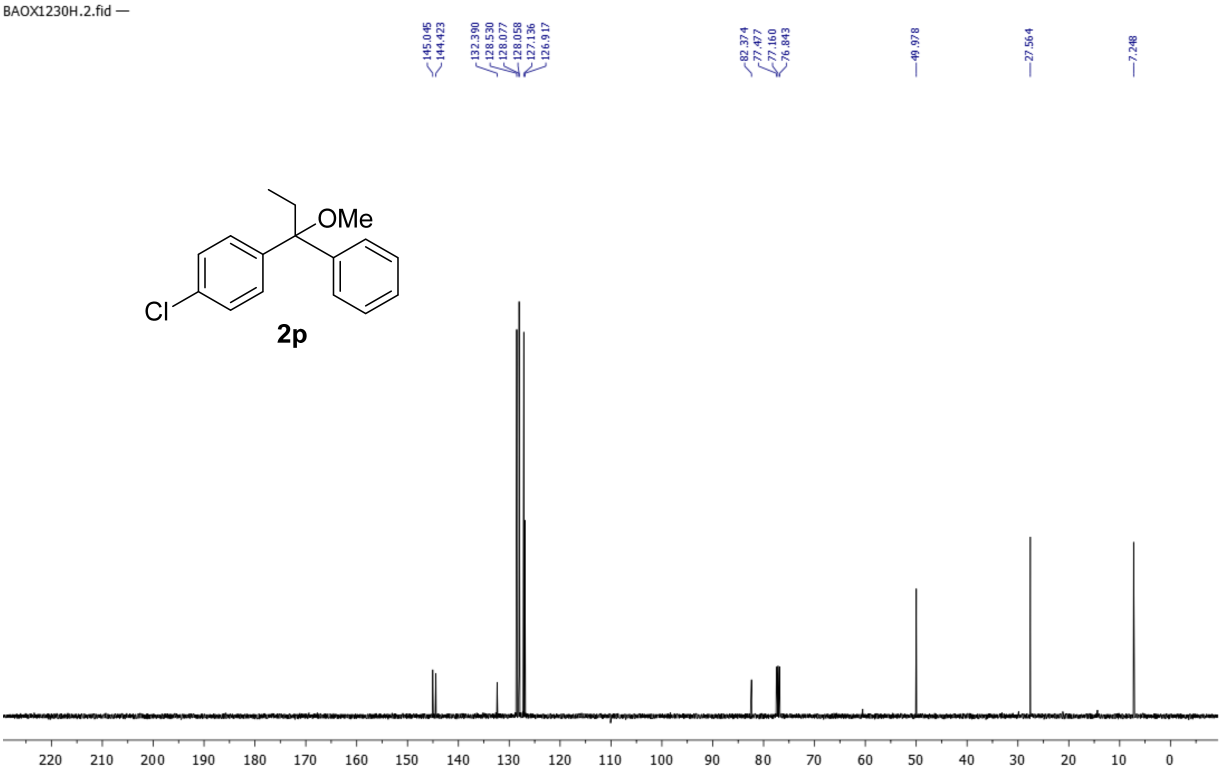

Supplementary Figure 33. ^{1}H and ^{13}C NMR spectra of 2p

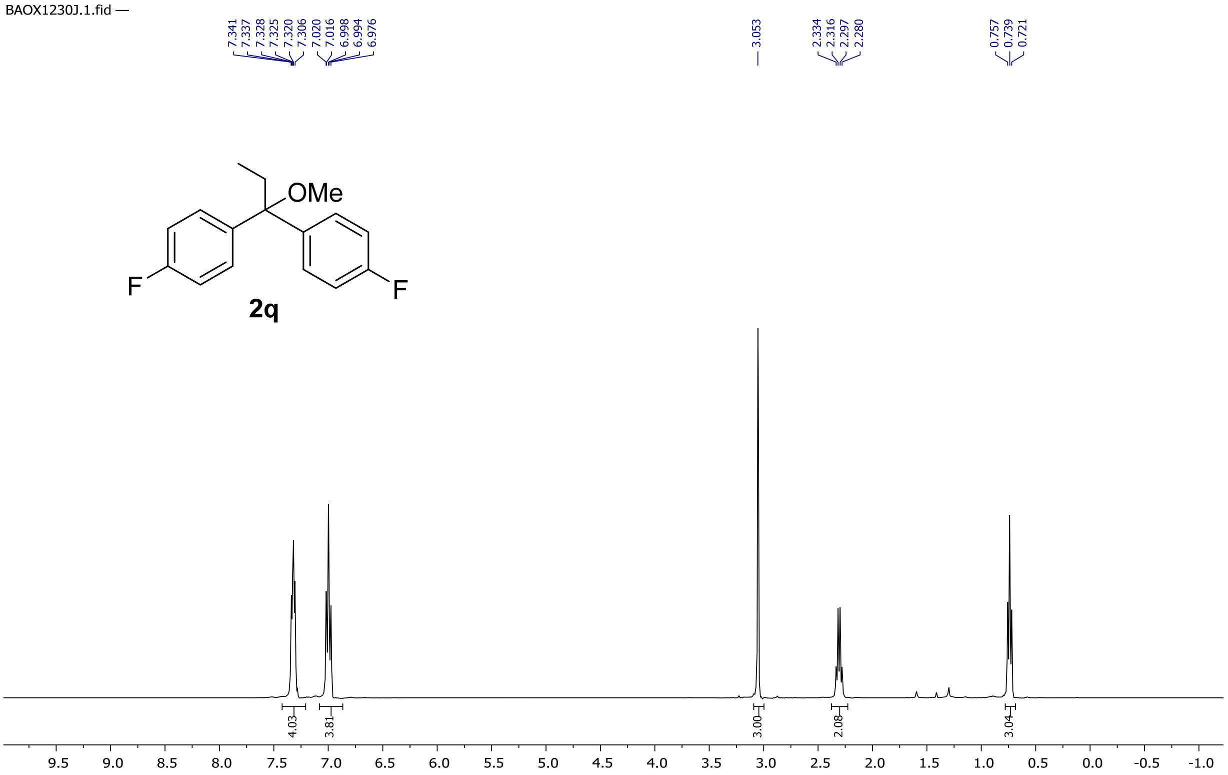

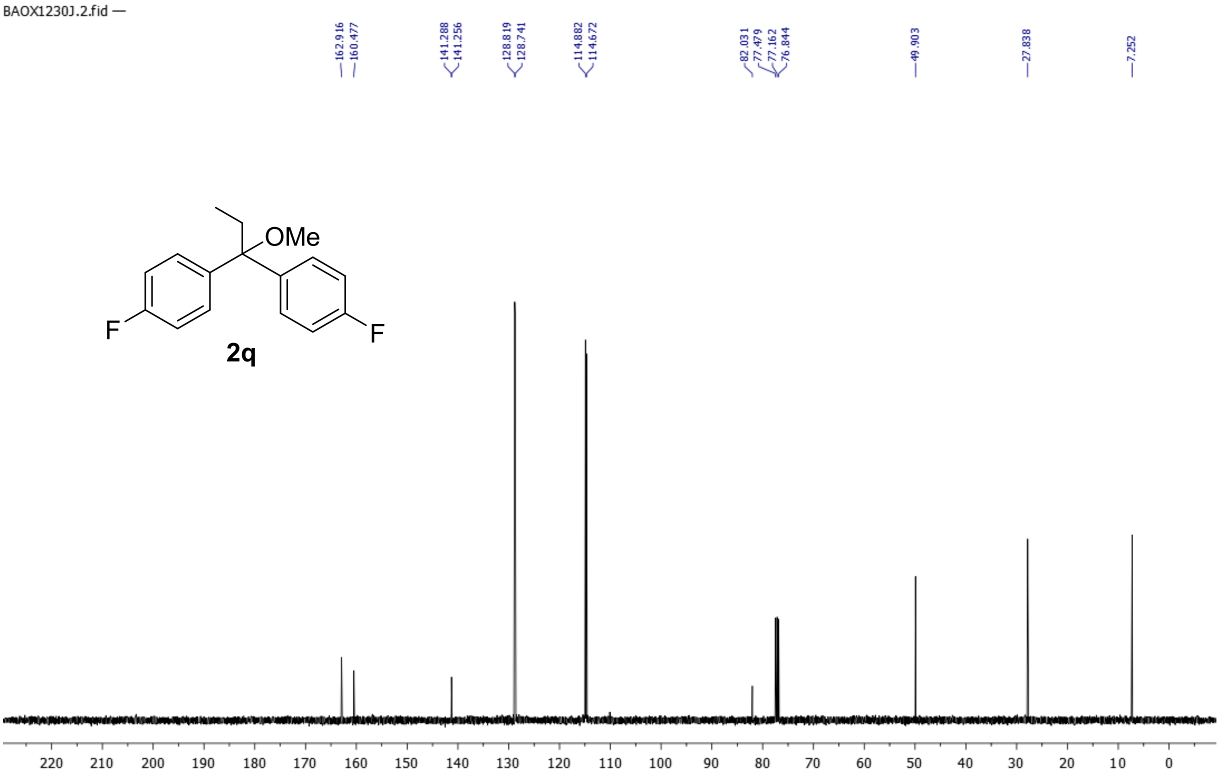

Supplementary Figure 34. ^{1}H and ^{13}C NMR spectra of 2q

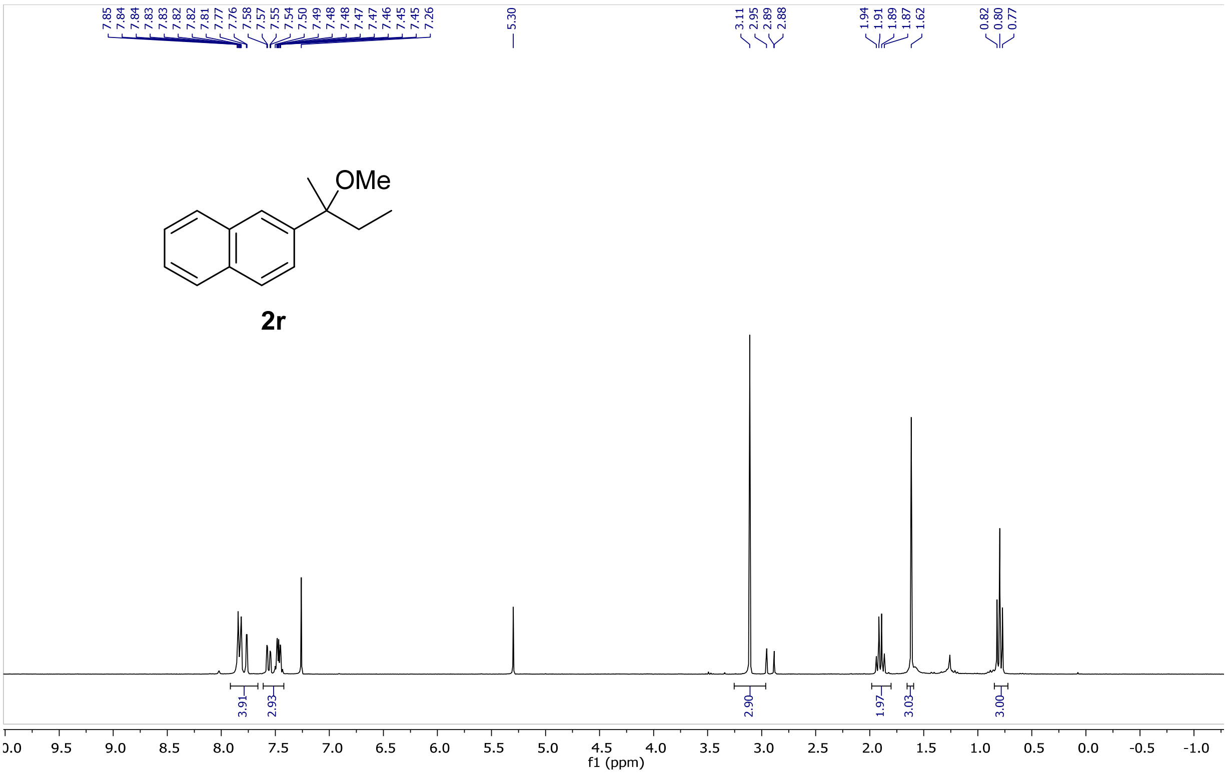

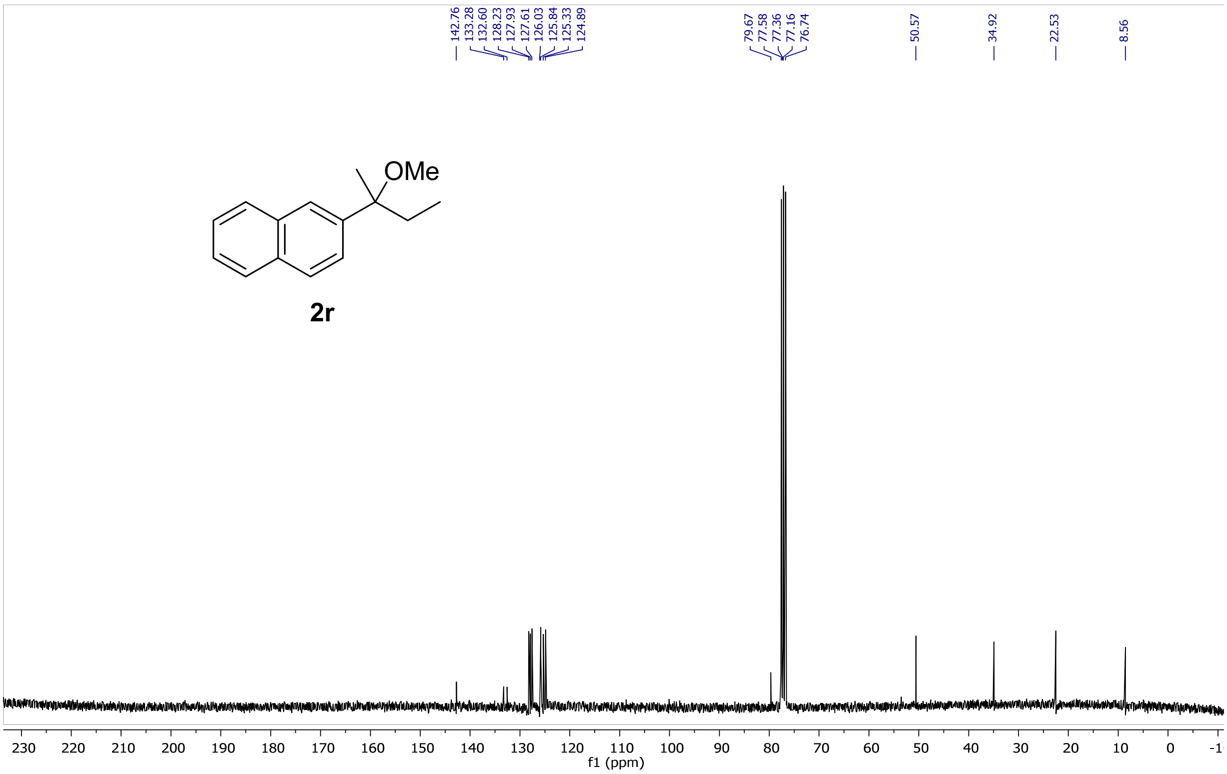

Supplementary Figure 35. ^{1}H and ^{13}C NMR spectra of 2r

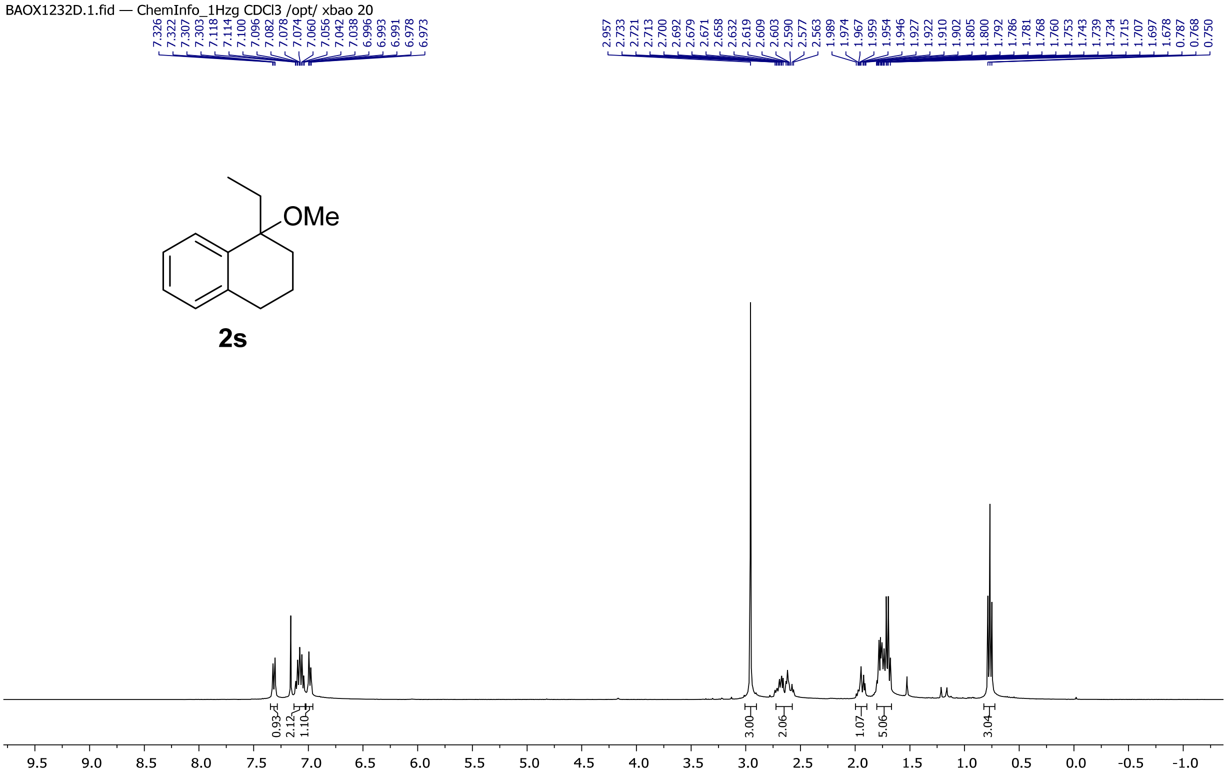

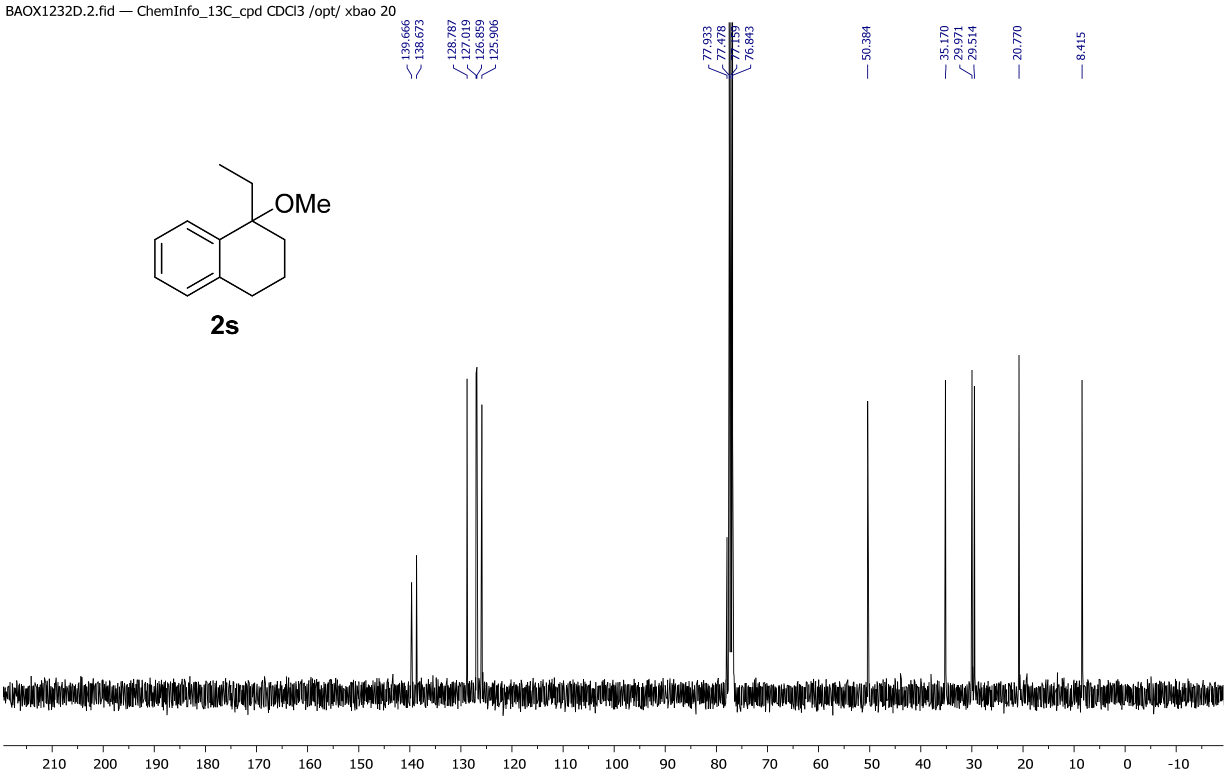

Supplementary Figure 36. ^{1}H and ^{13}C NMR spectra of 2s

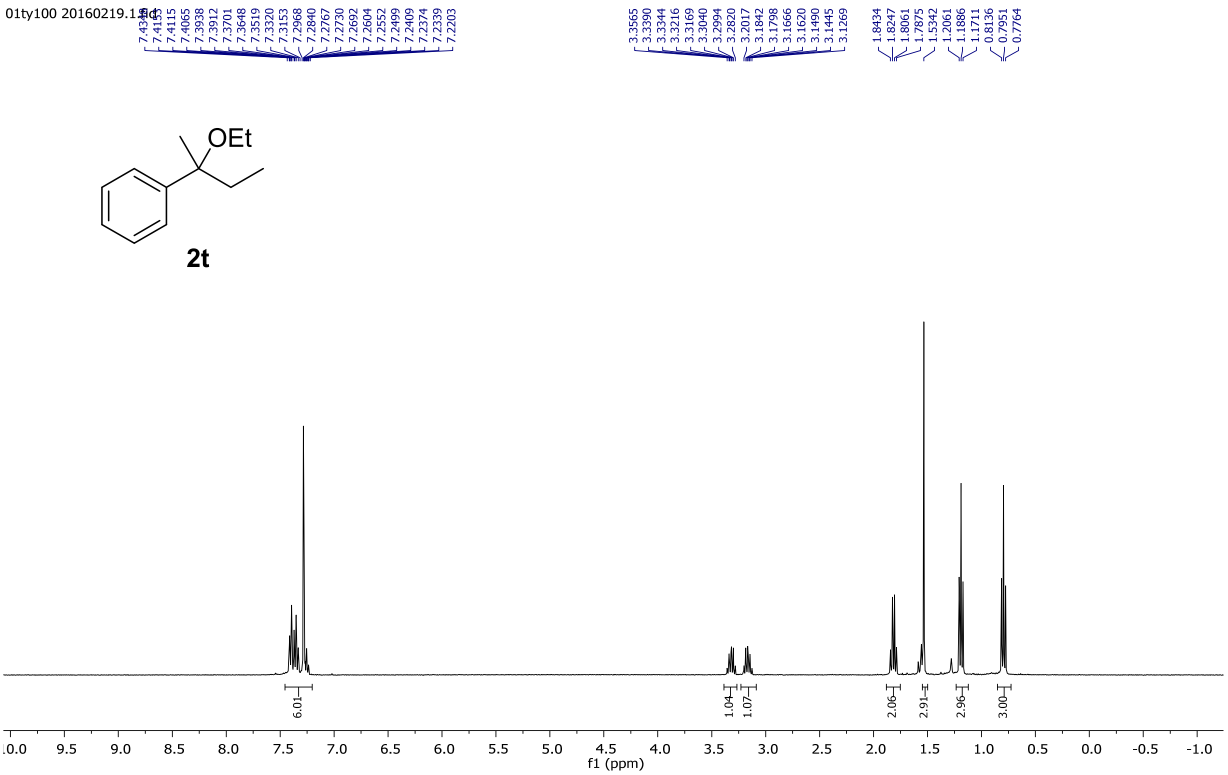

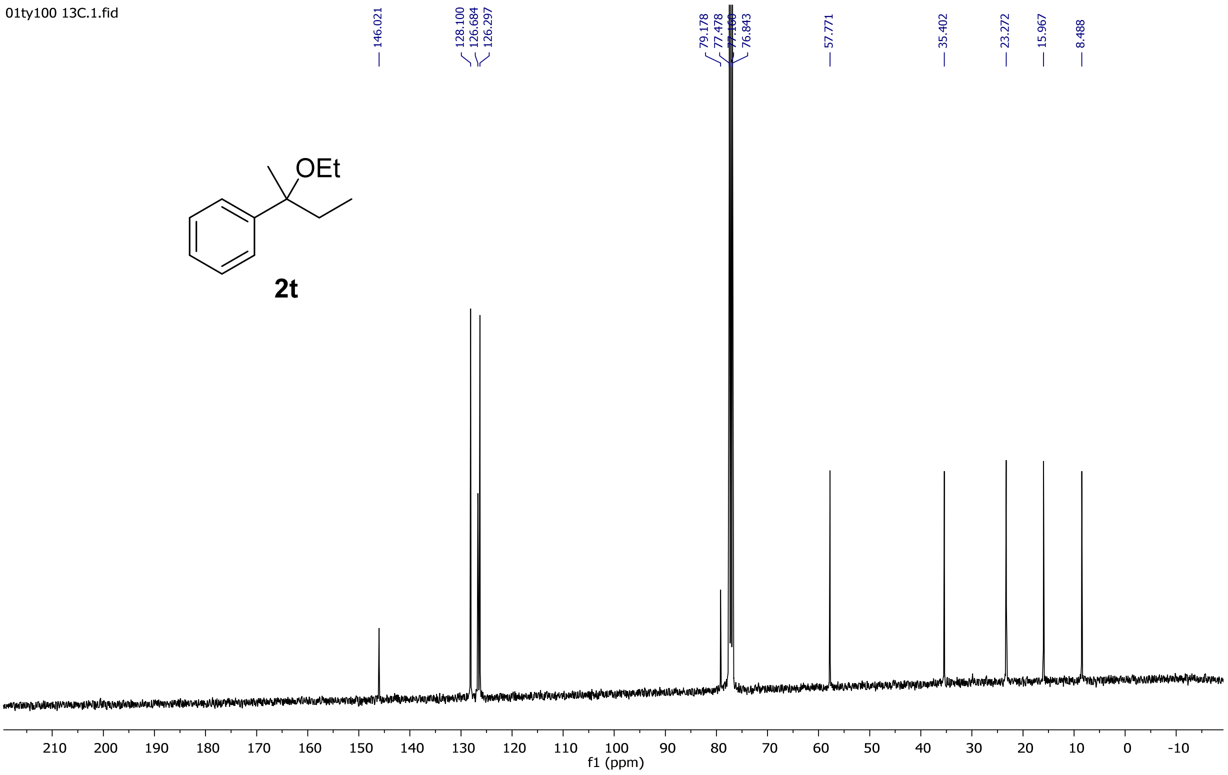

Supplementary Figure 37. ^{1}H and ^{13}C NMR spectra of 2t

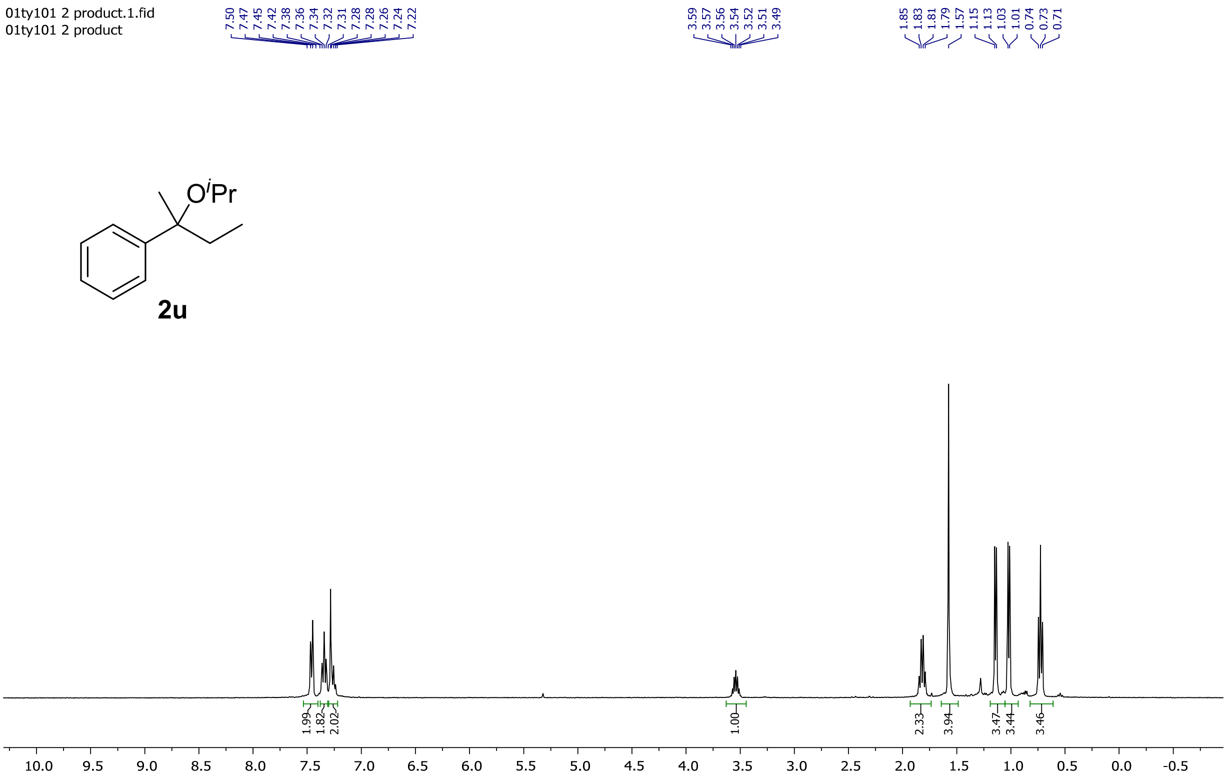

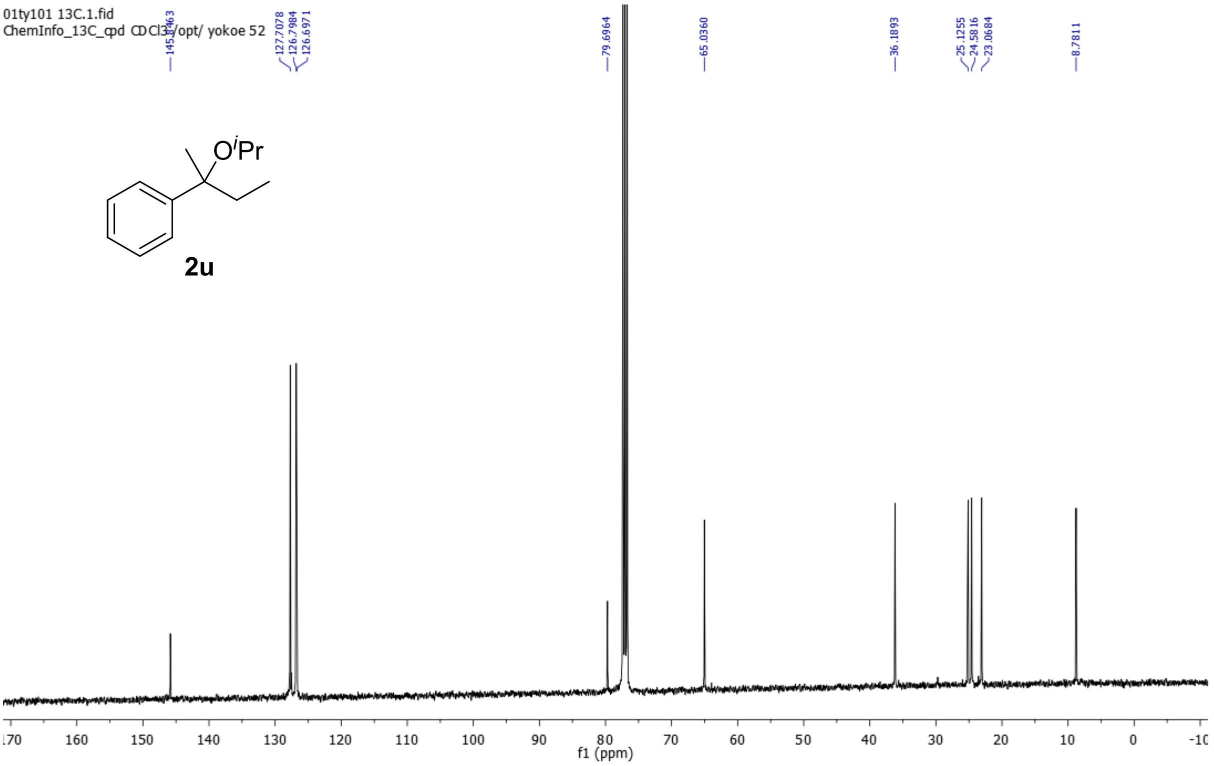

Supplementary Figure 38. ^{1}H and ^{13}C NMR spectra of 2t

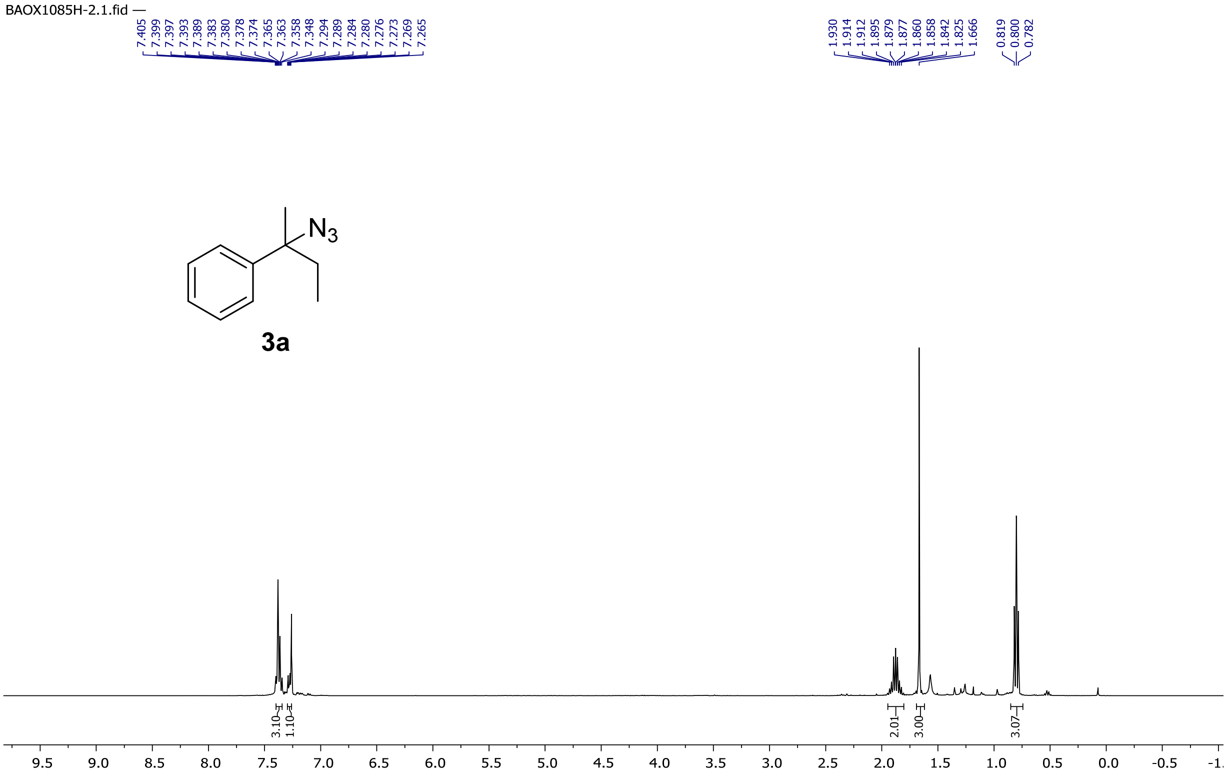

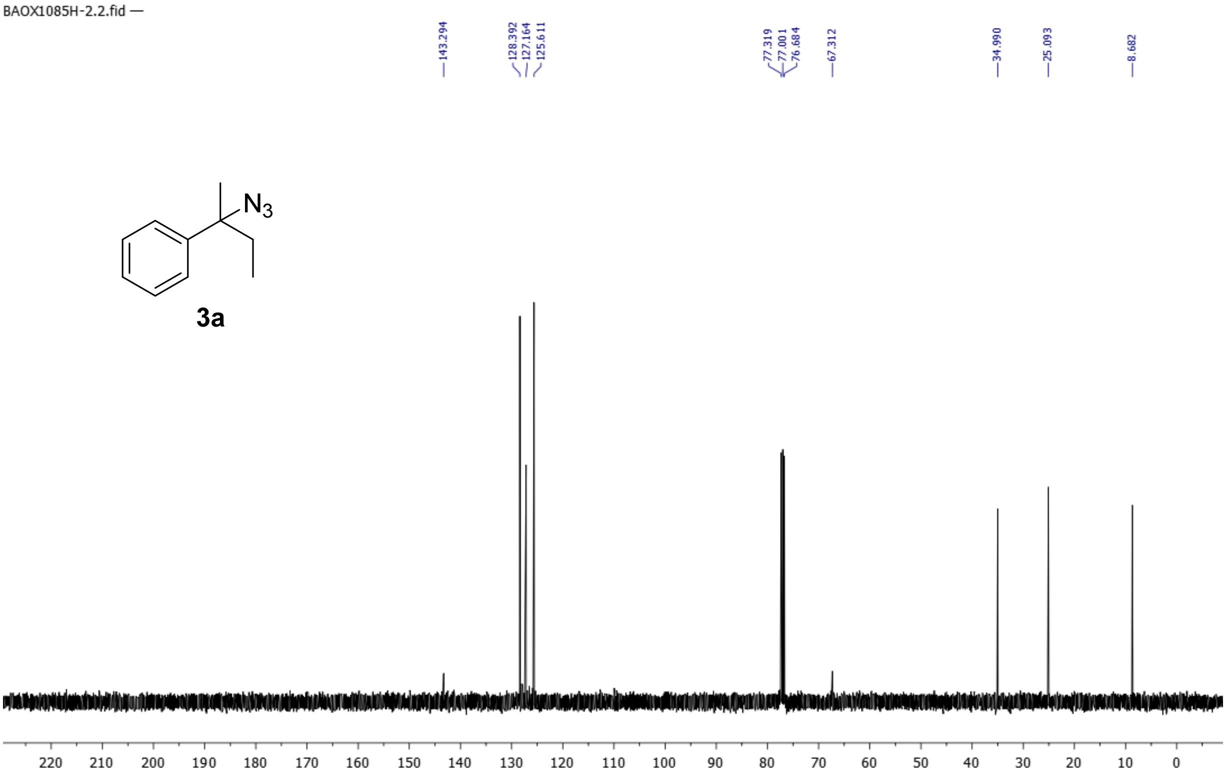

Supplementary Figure 39. ^{1}H and ^{13}C NMR spectra of 3a

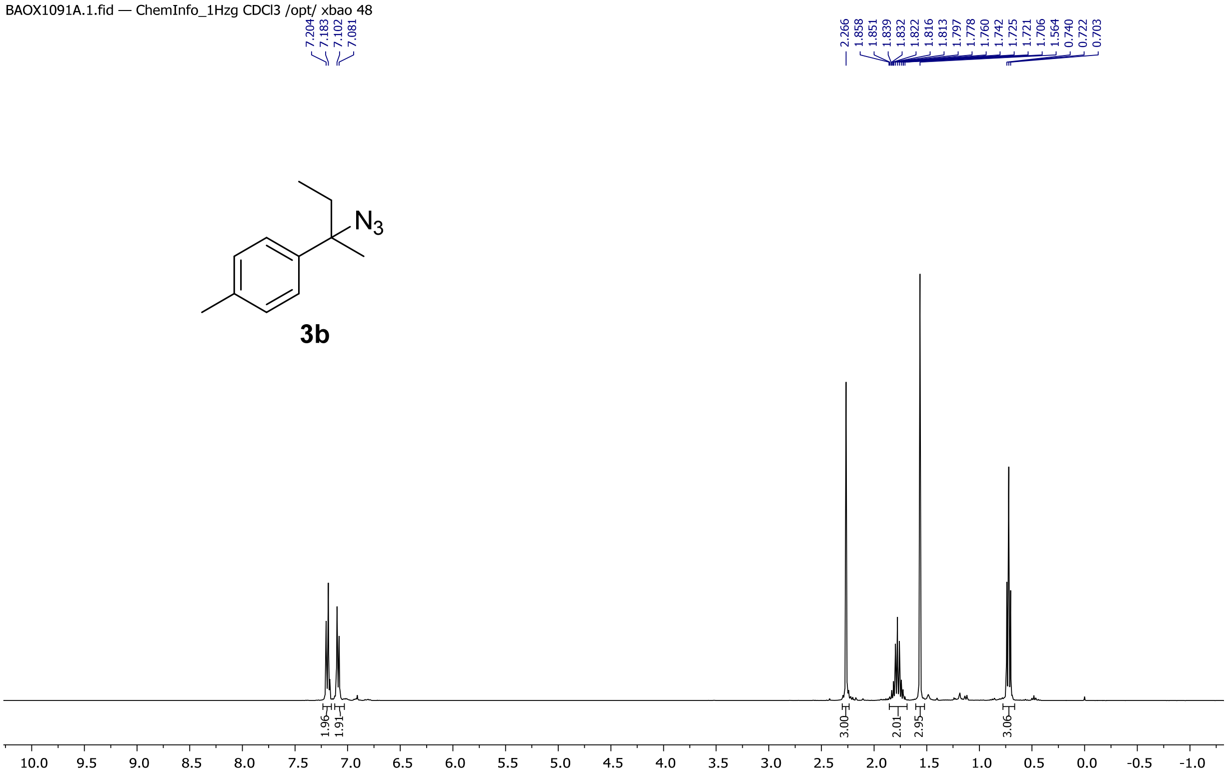

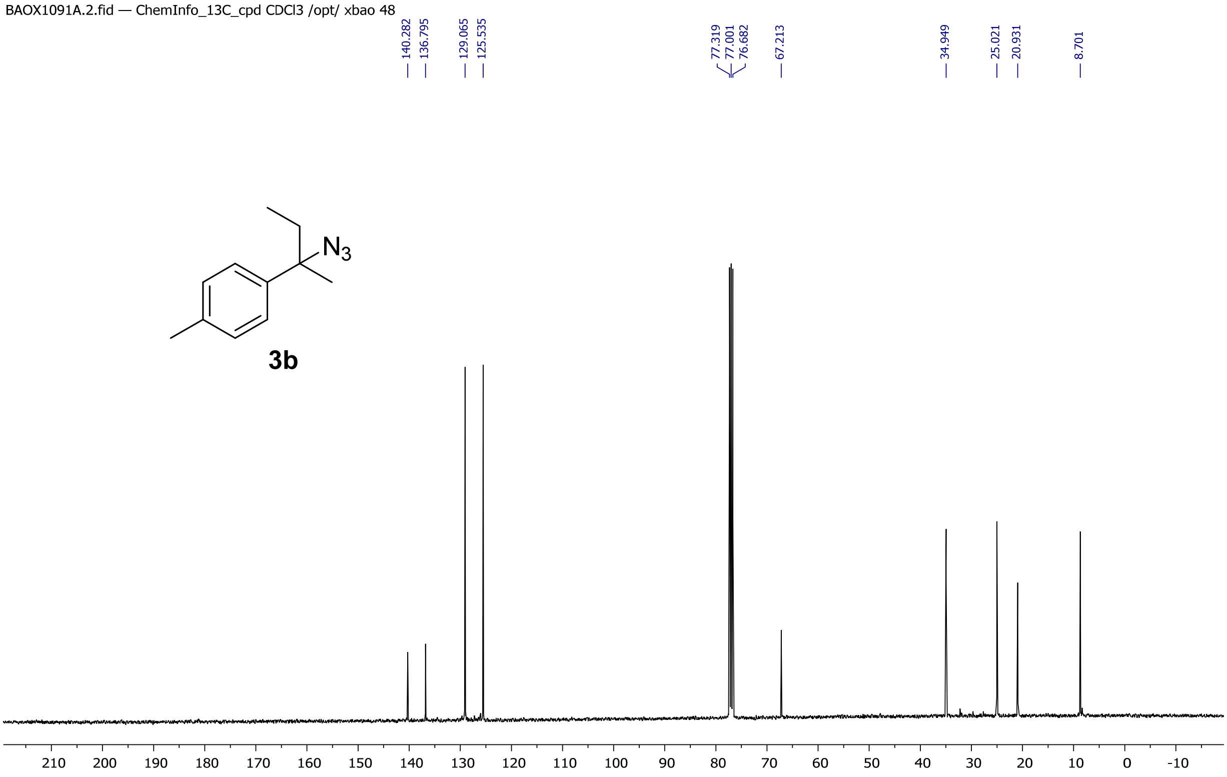

Supplementary Figure 40. ^{1}H and ^{13}C NMR spectra of 3b

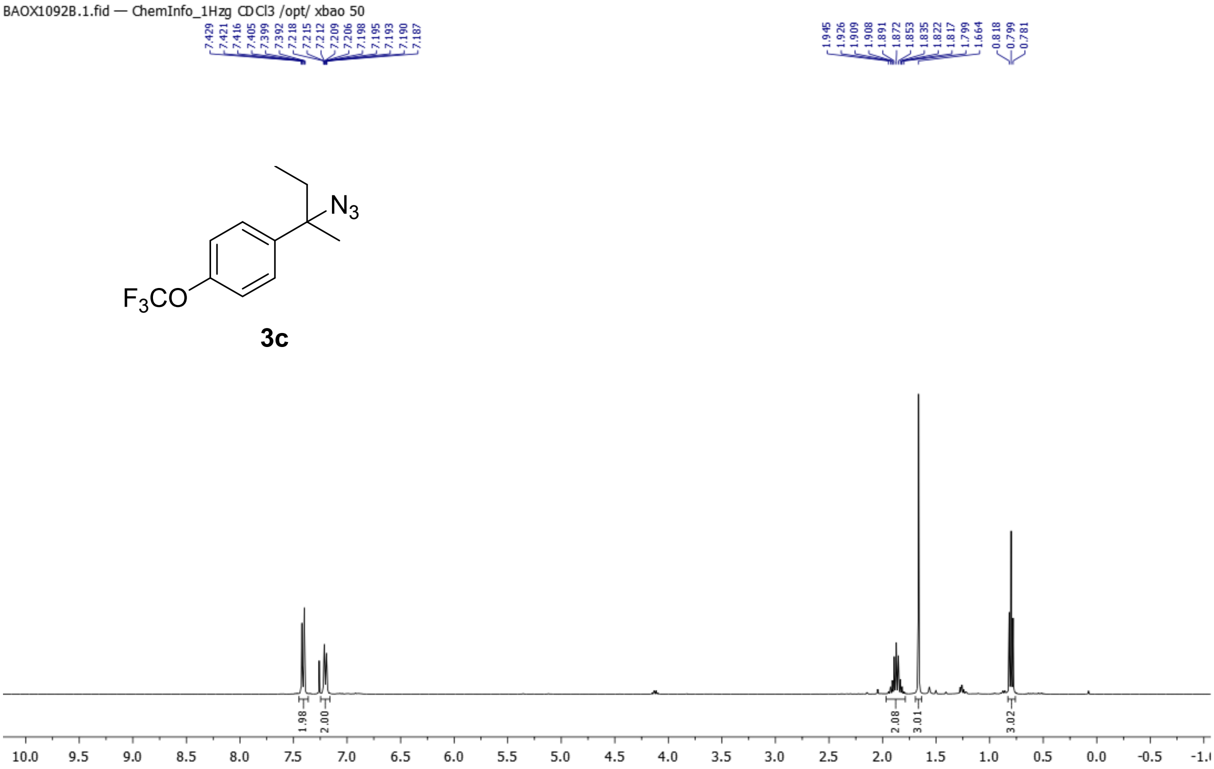

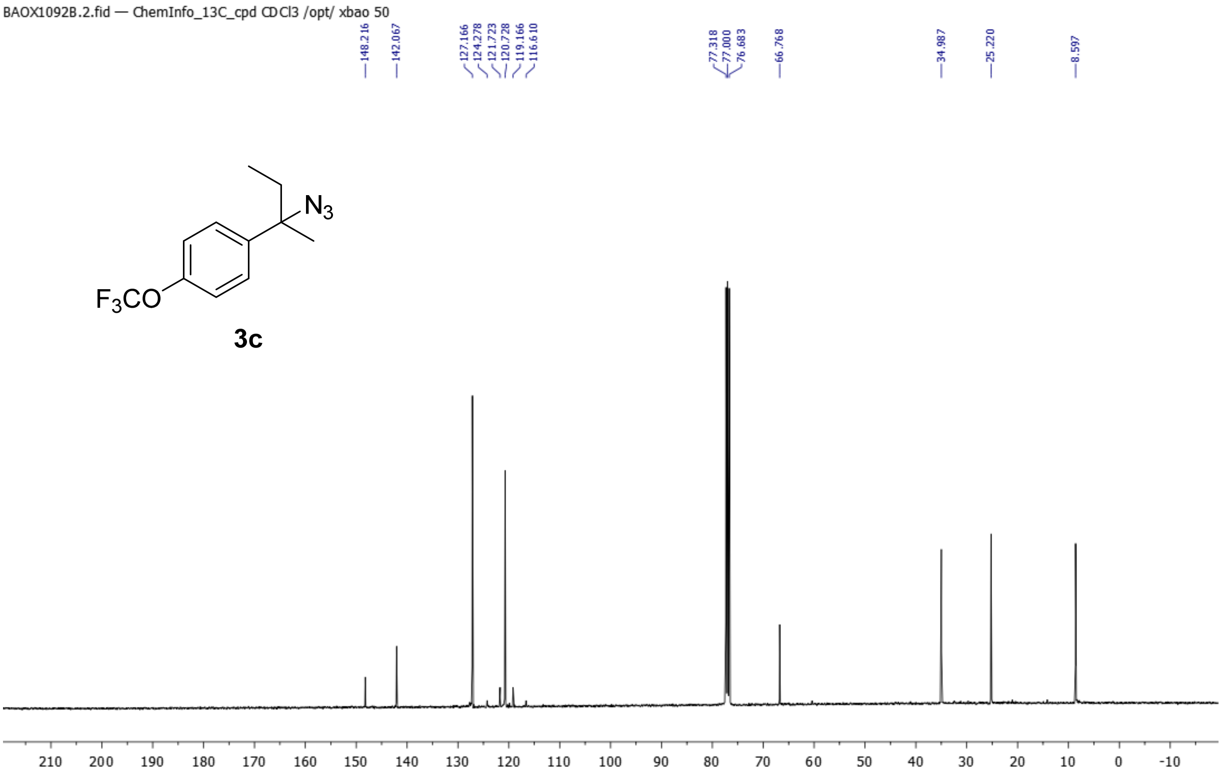

Supplementary Figure 41. ^{1}H and ^{13}C NMR spectra of 3c

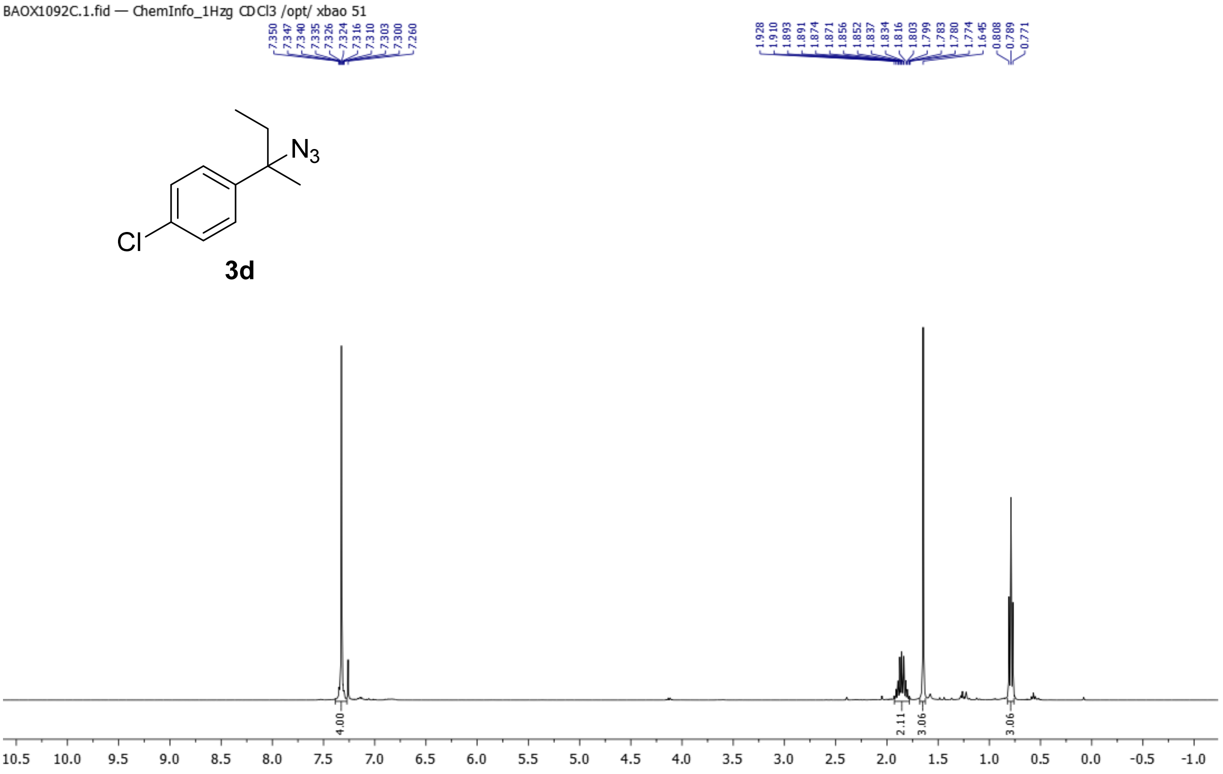

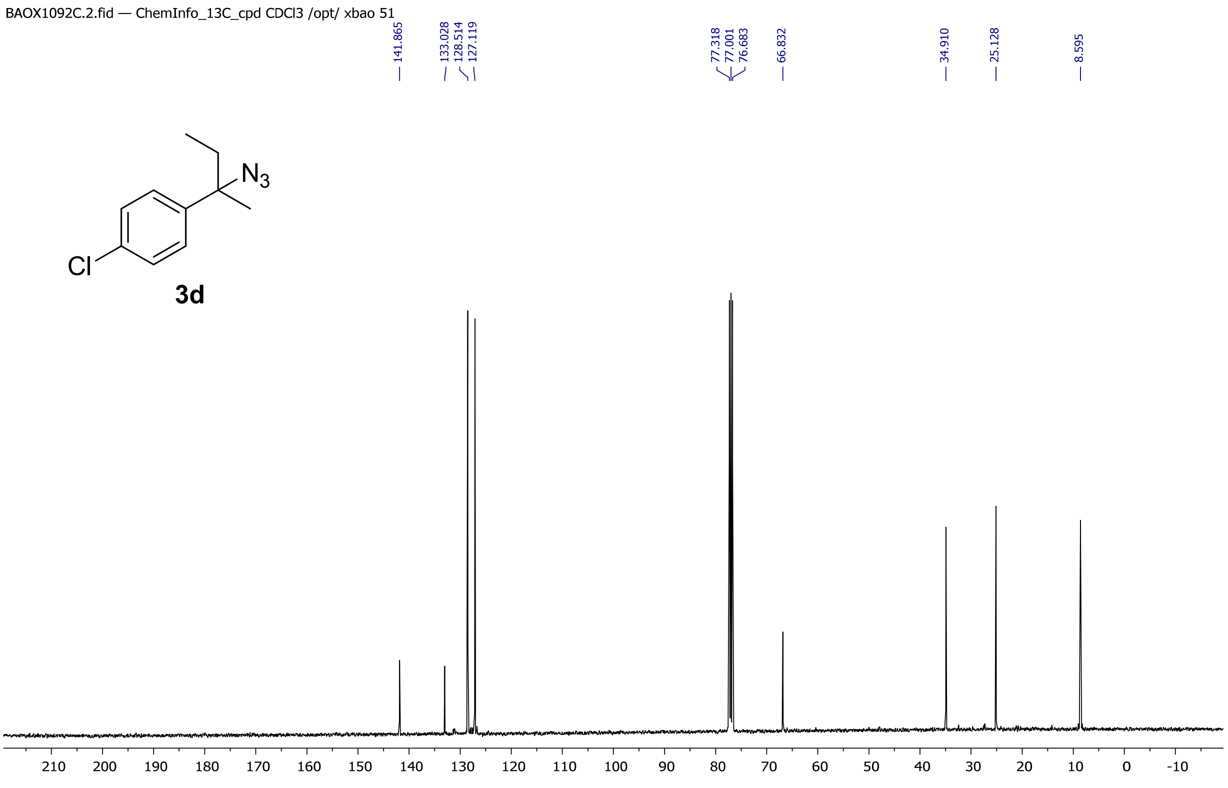

Supplementary Figure 42. ^{1}H and ^{13}C NMR spectra of 3d

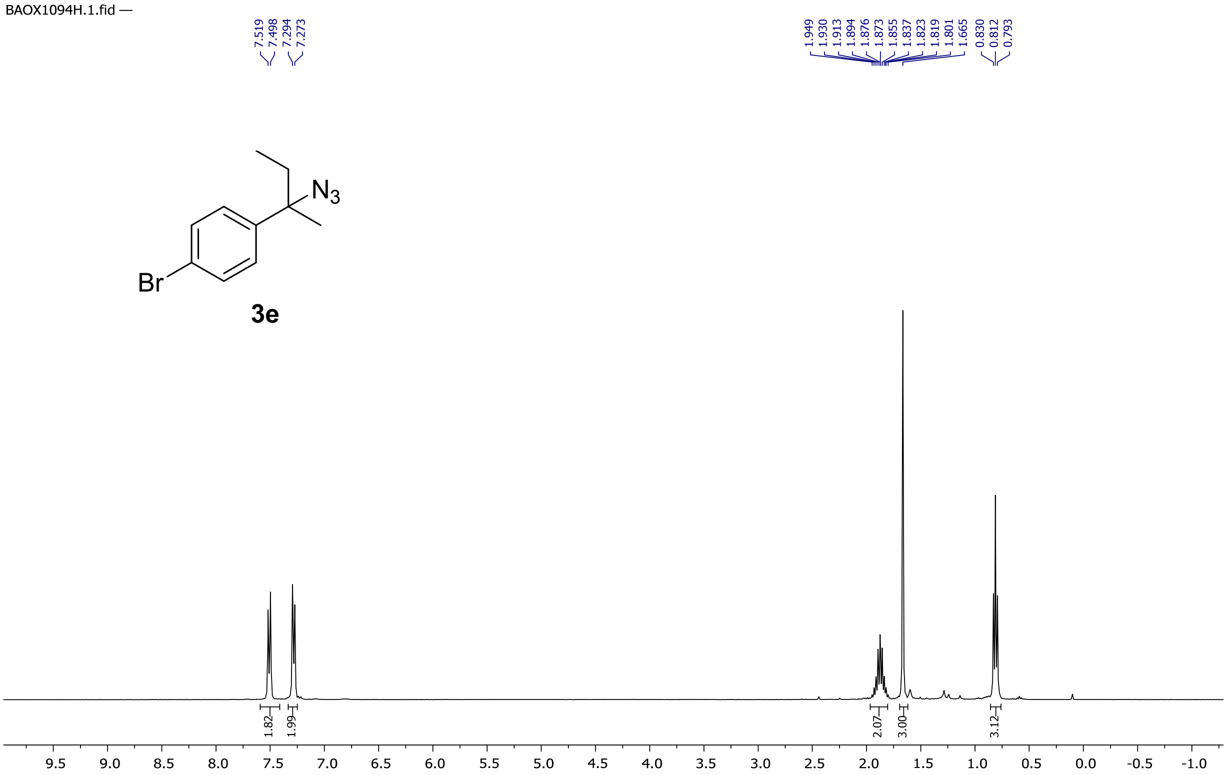

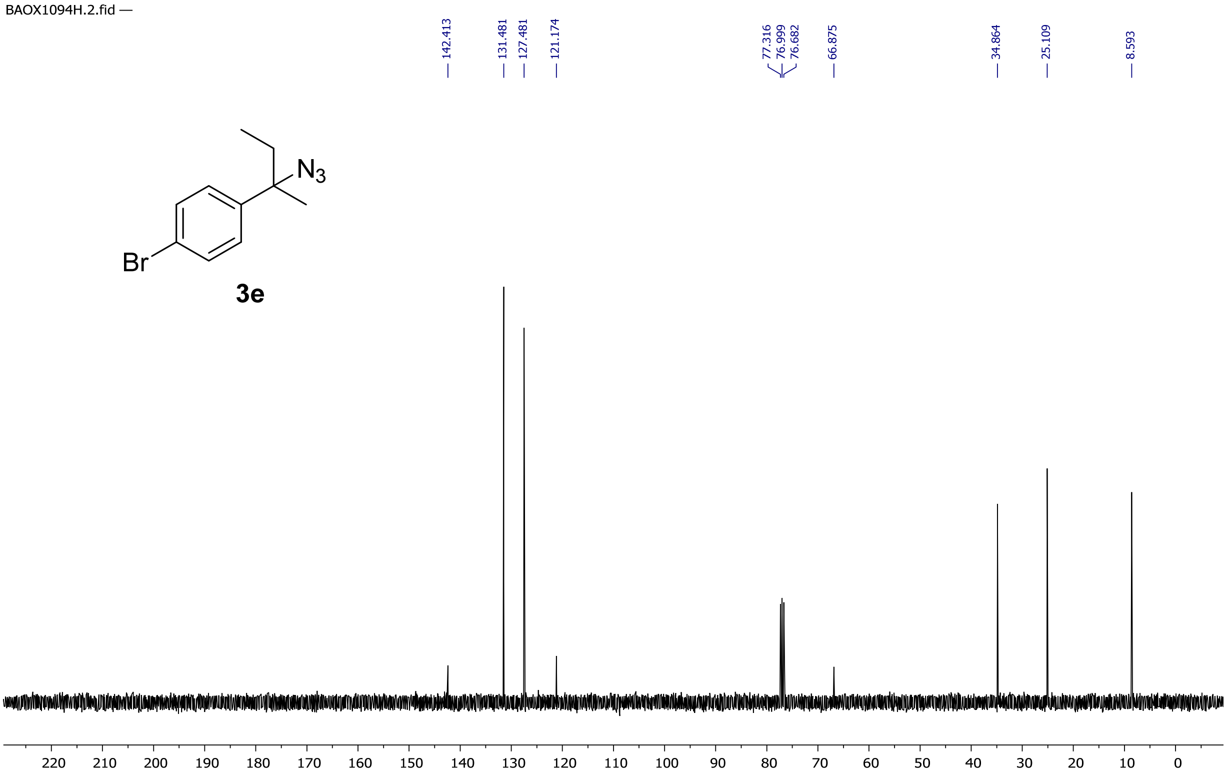

Supplementary Figure 43. ^{1}H and ^{13}C NMR spectra of 3e

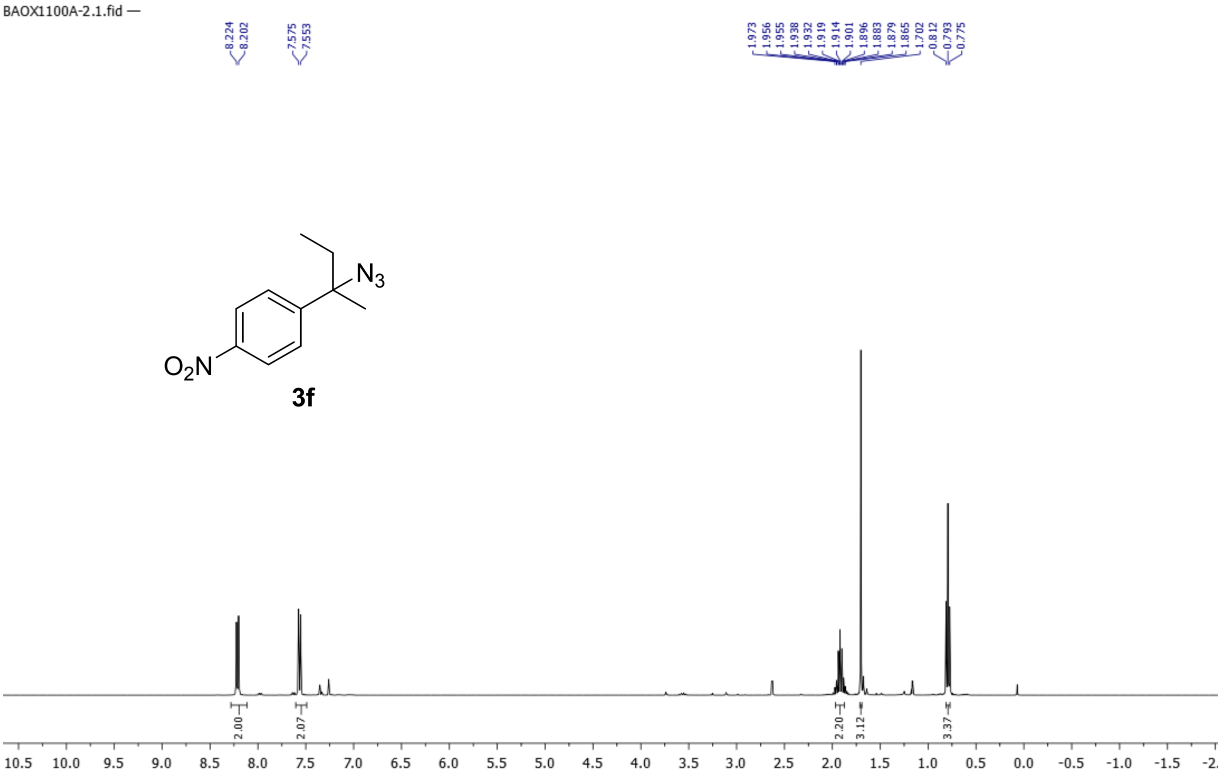

3f

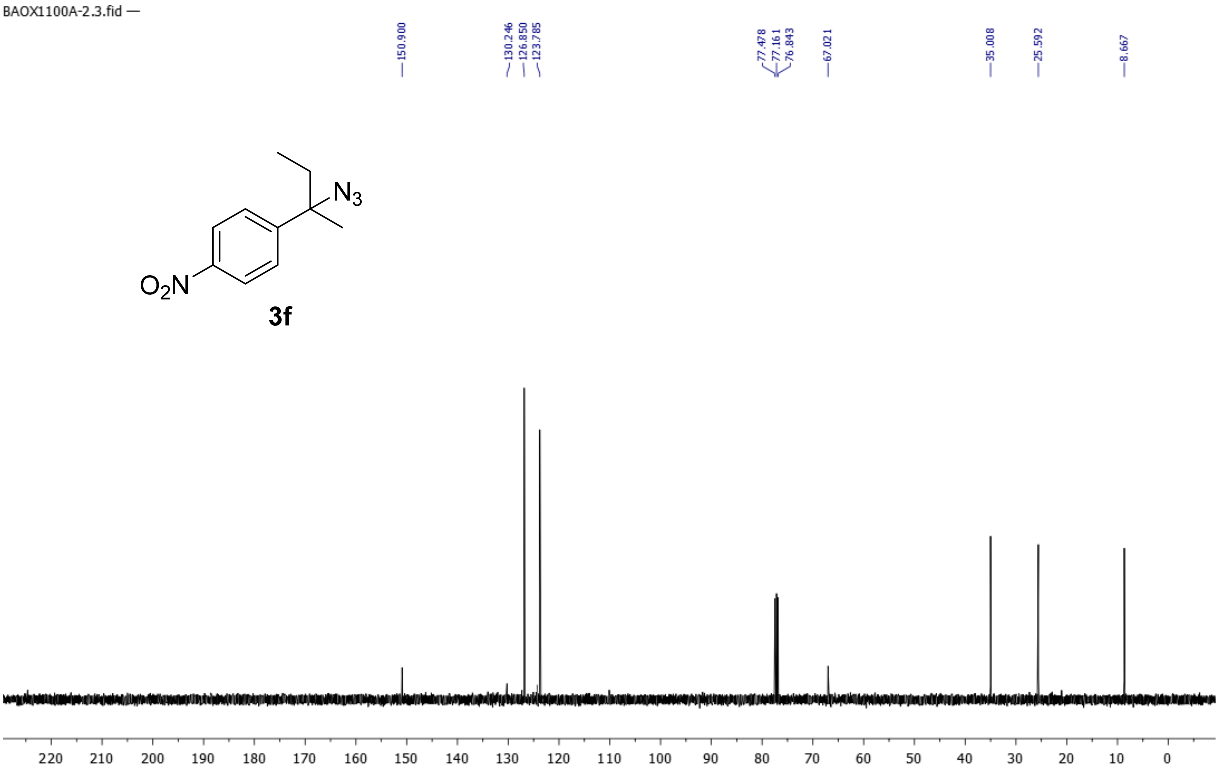

3f

Supplementary Figure 44. ^{1}H and ^{13}C NMR spectra of 3f

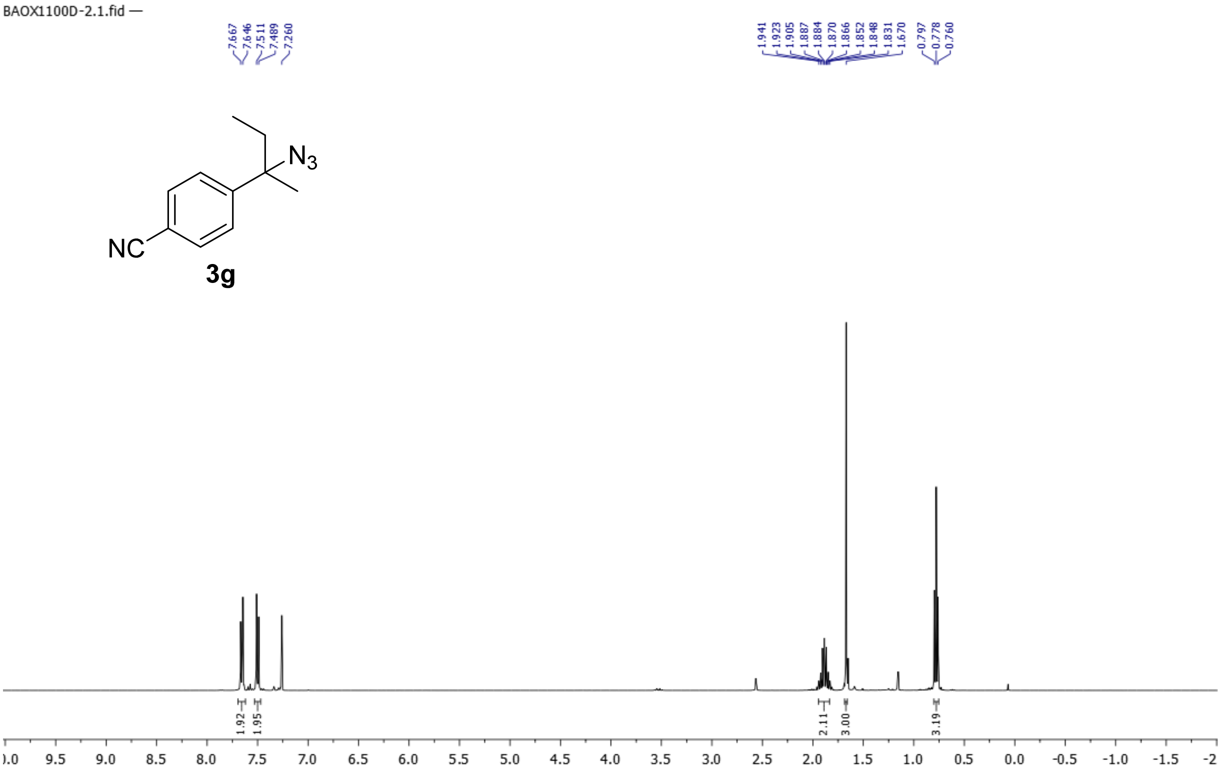

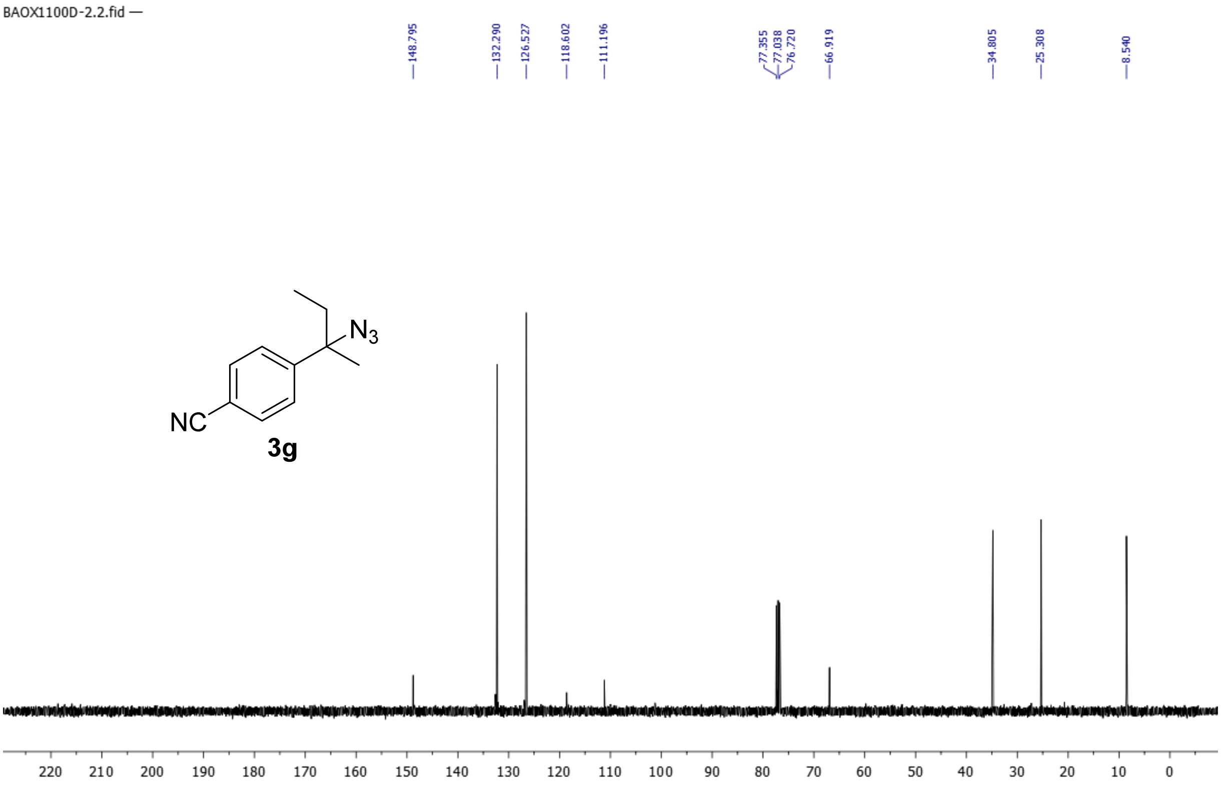

Supplementary Figure 45. ^{1}H and ^{13}C NMR spectra of 3g

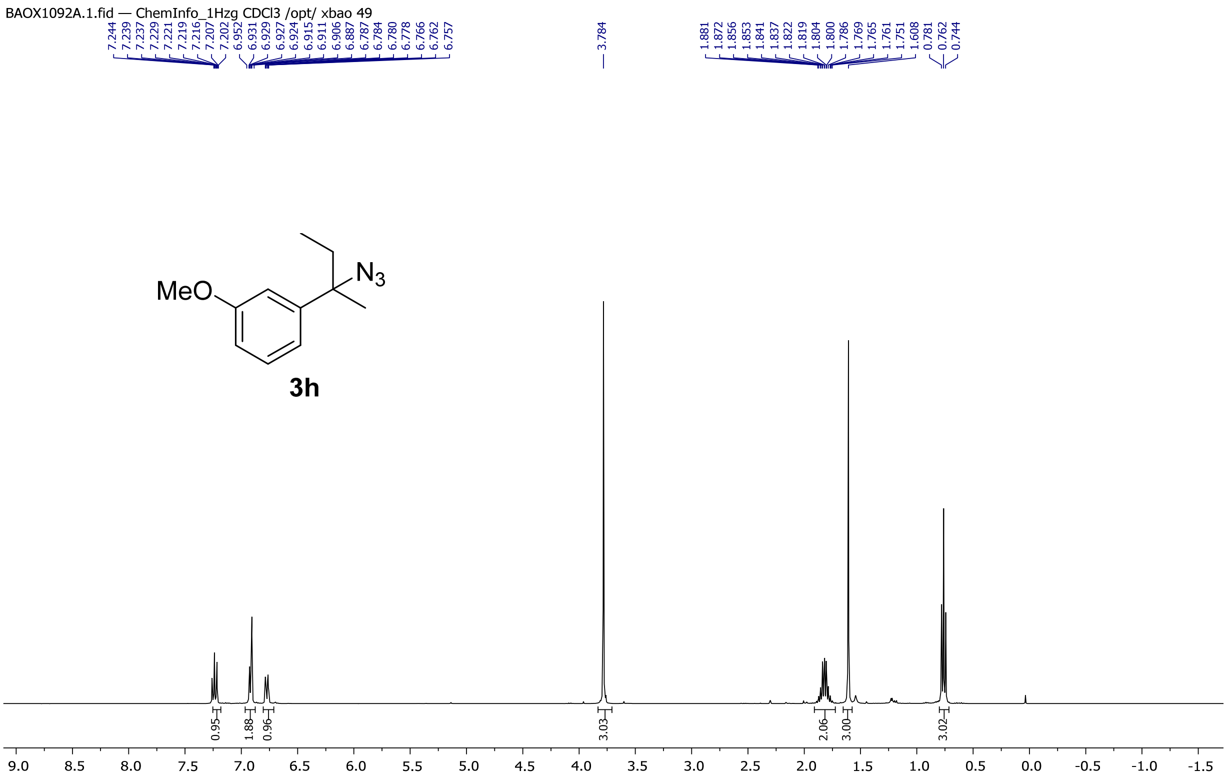

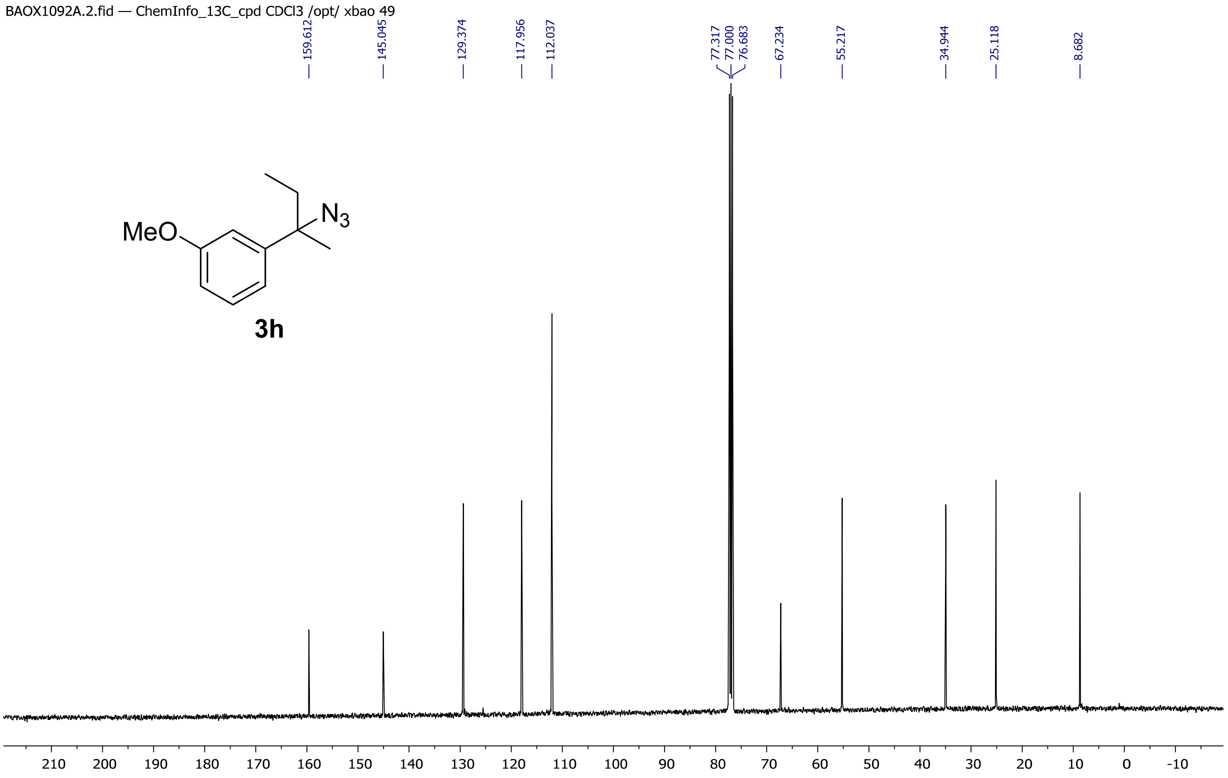

Supplementary Figure 46. ^{1}H and ^{13}C NMR spectra of 3h

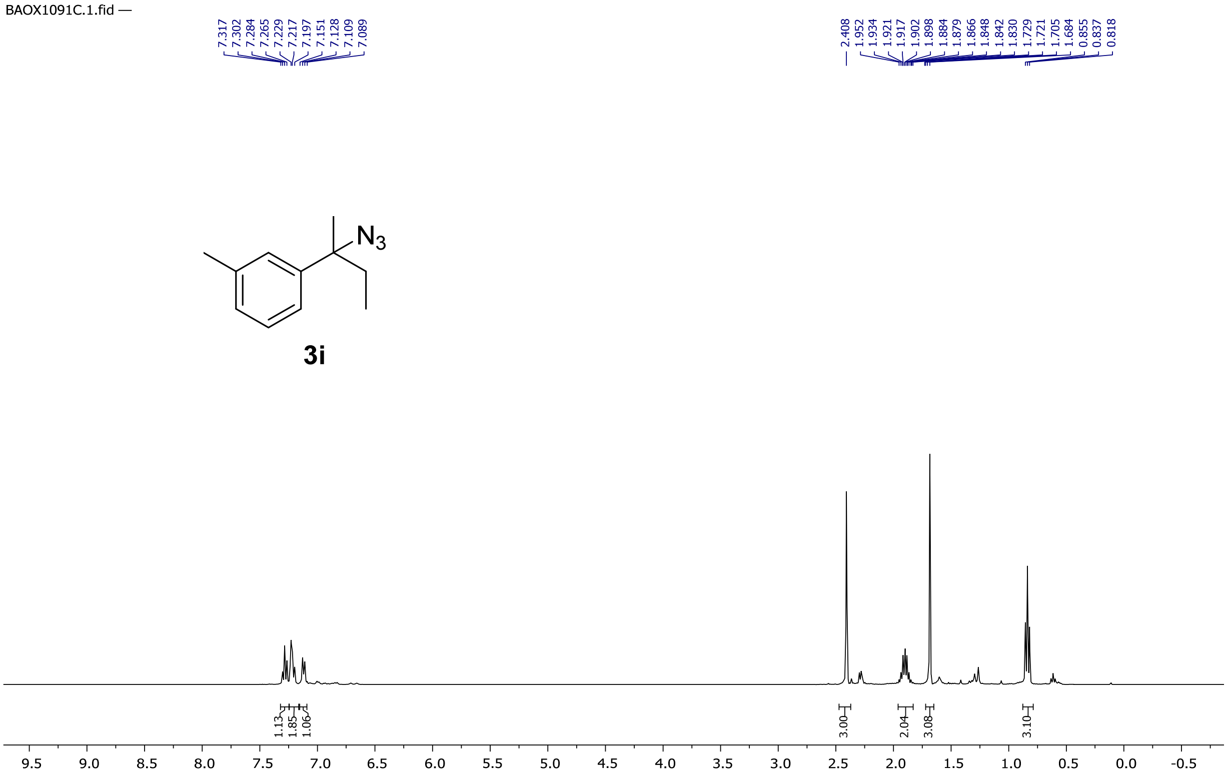

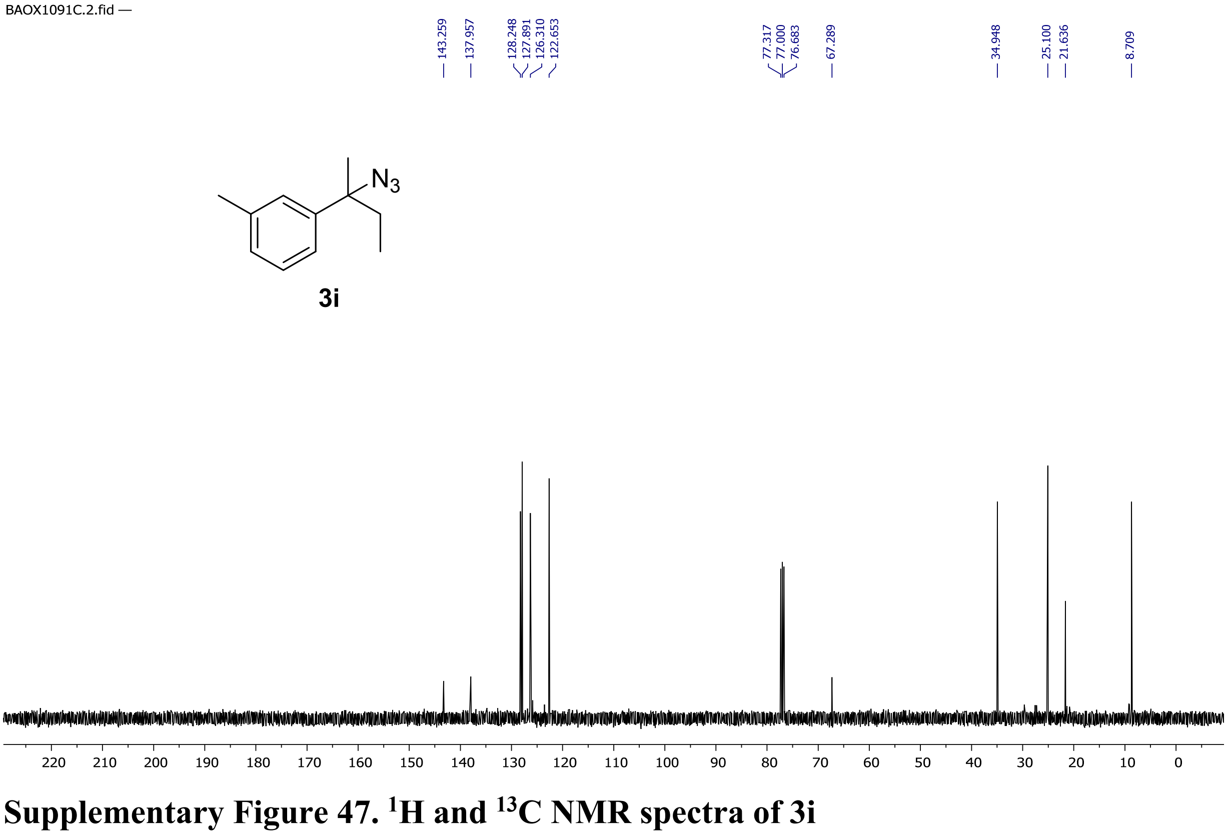

Supplementary Figure 47. ^{1}H and ^{13}C NMR spectra of 3i

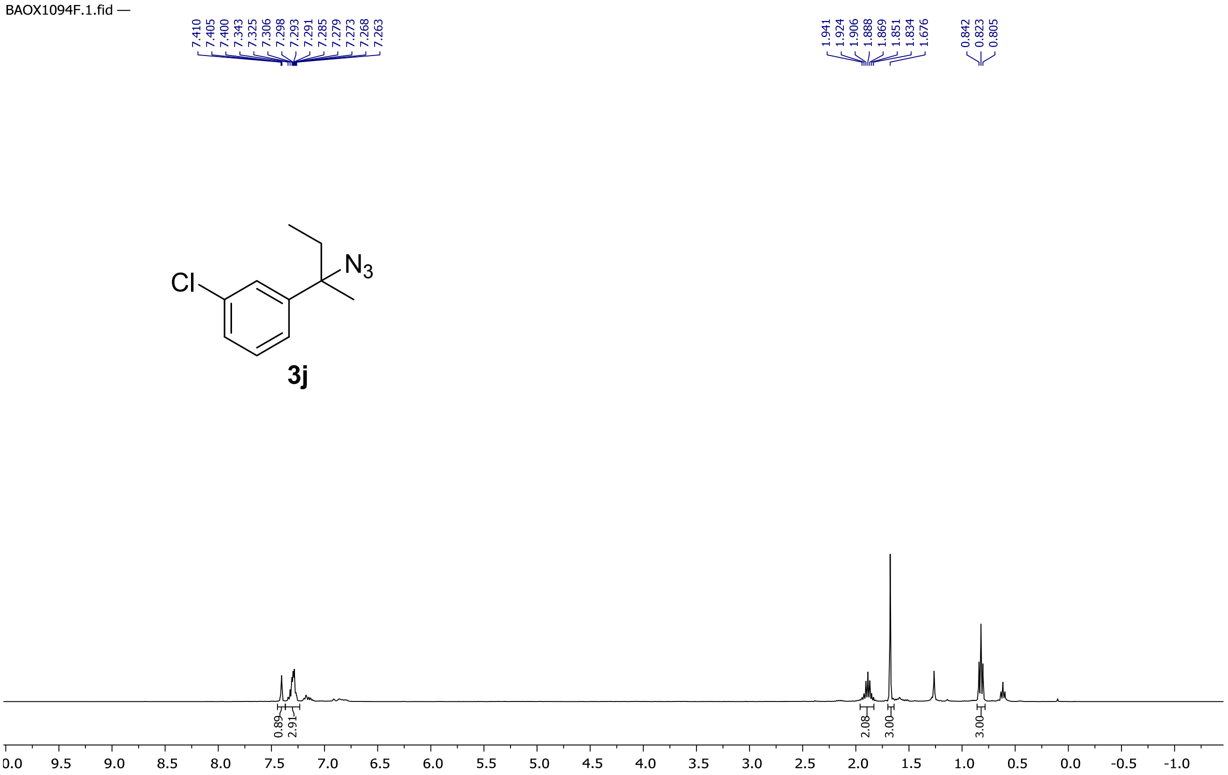

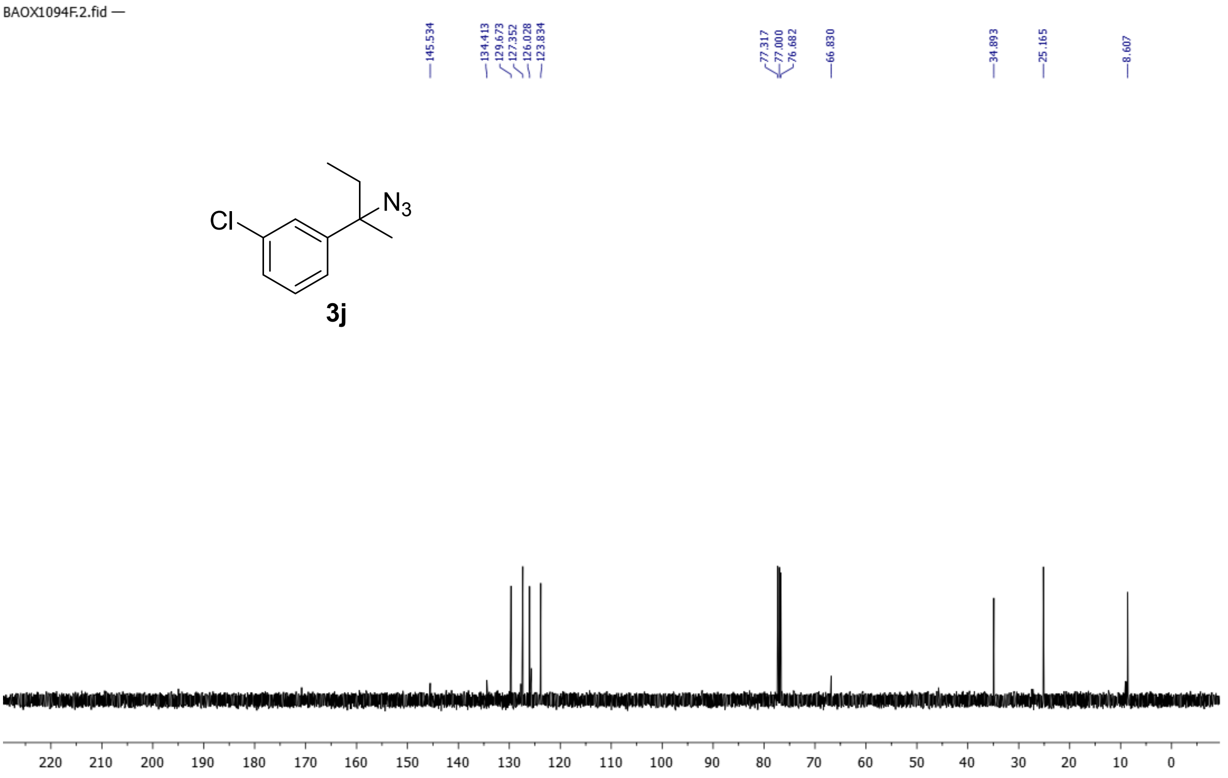

Supplementary Figure 48. ^{1}H and ^{13}C NMR spectra of 3j

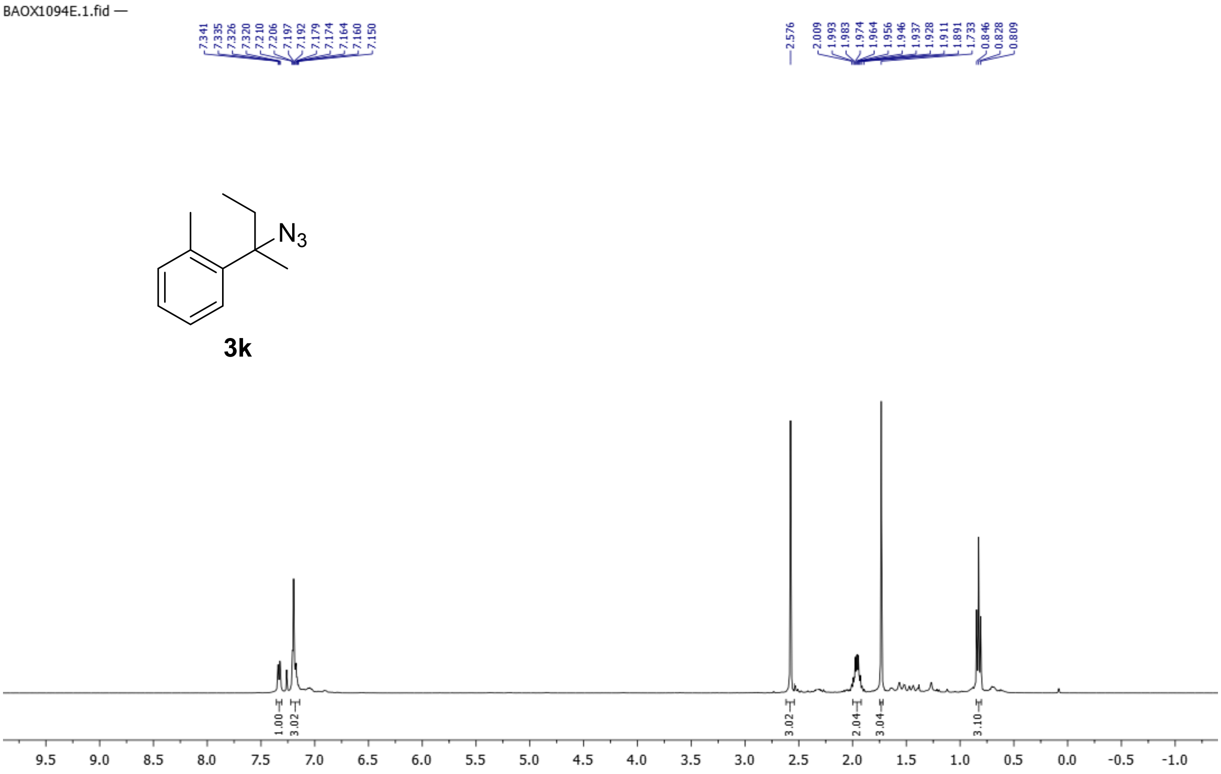

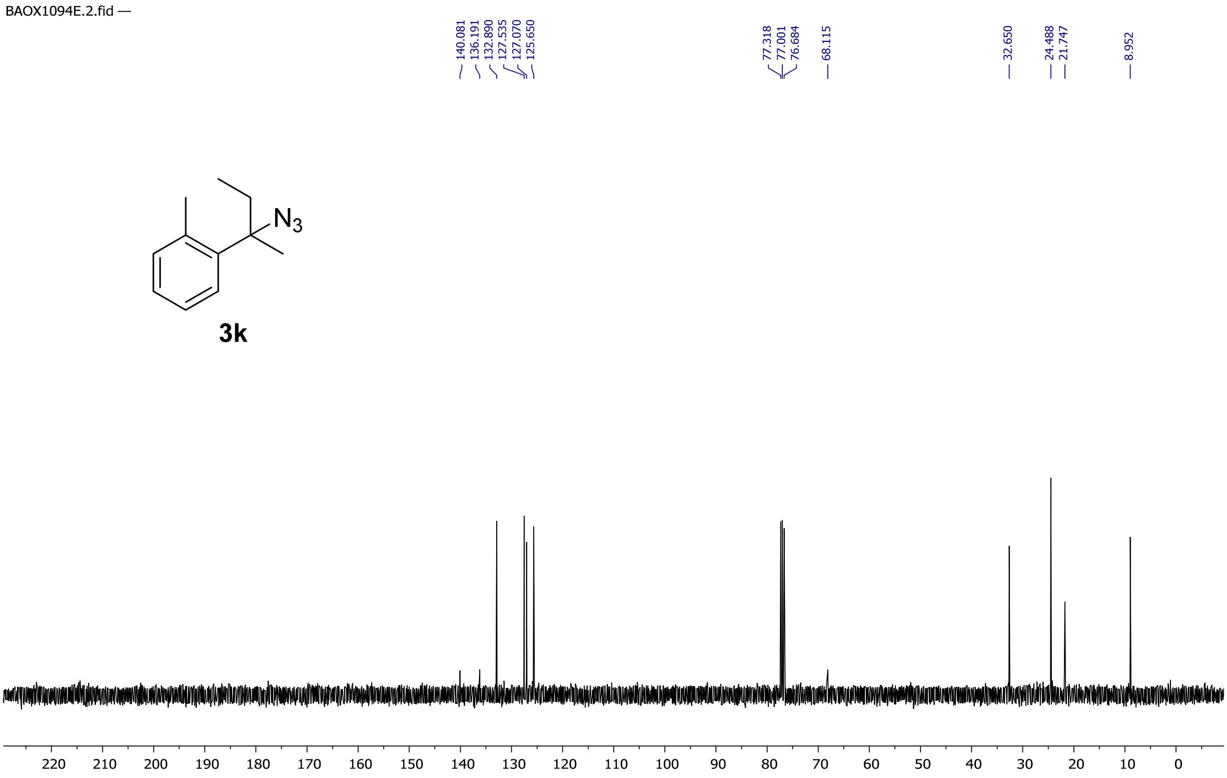

Supplementary Figure 49. ^{1}H and ^{13}C NMR spectra of 3k

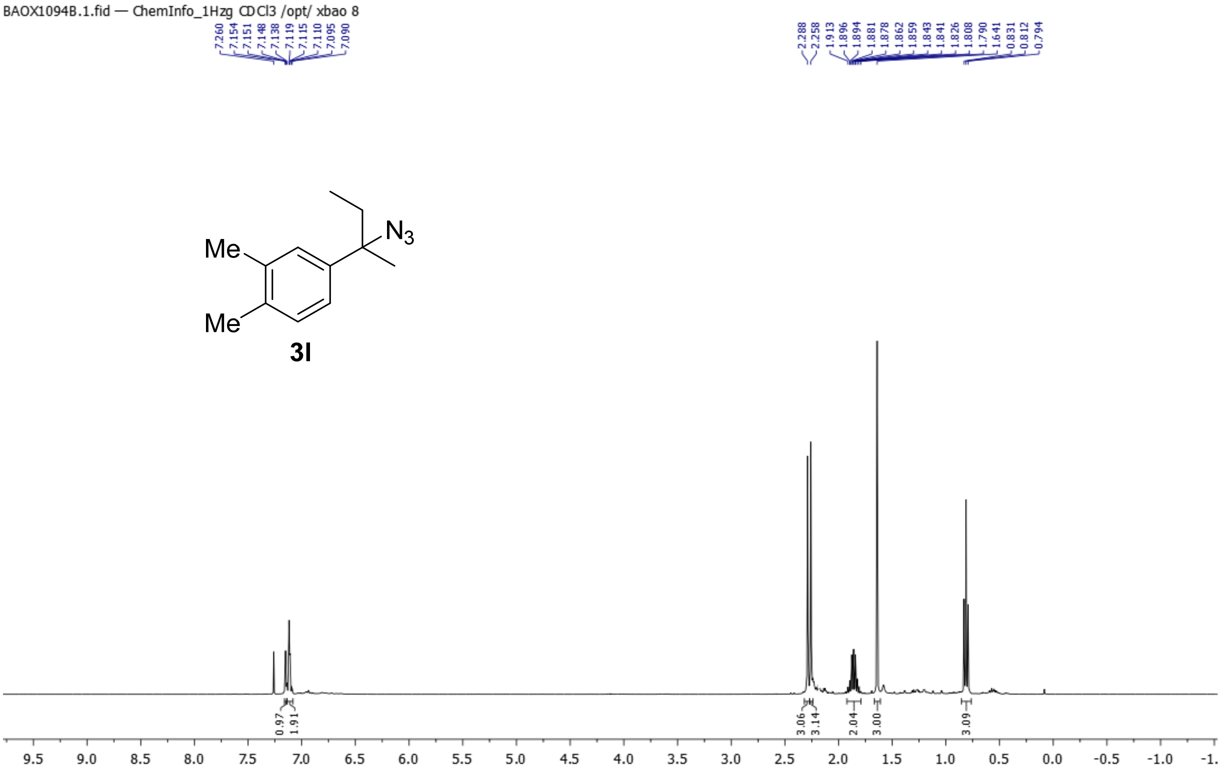

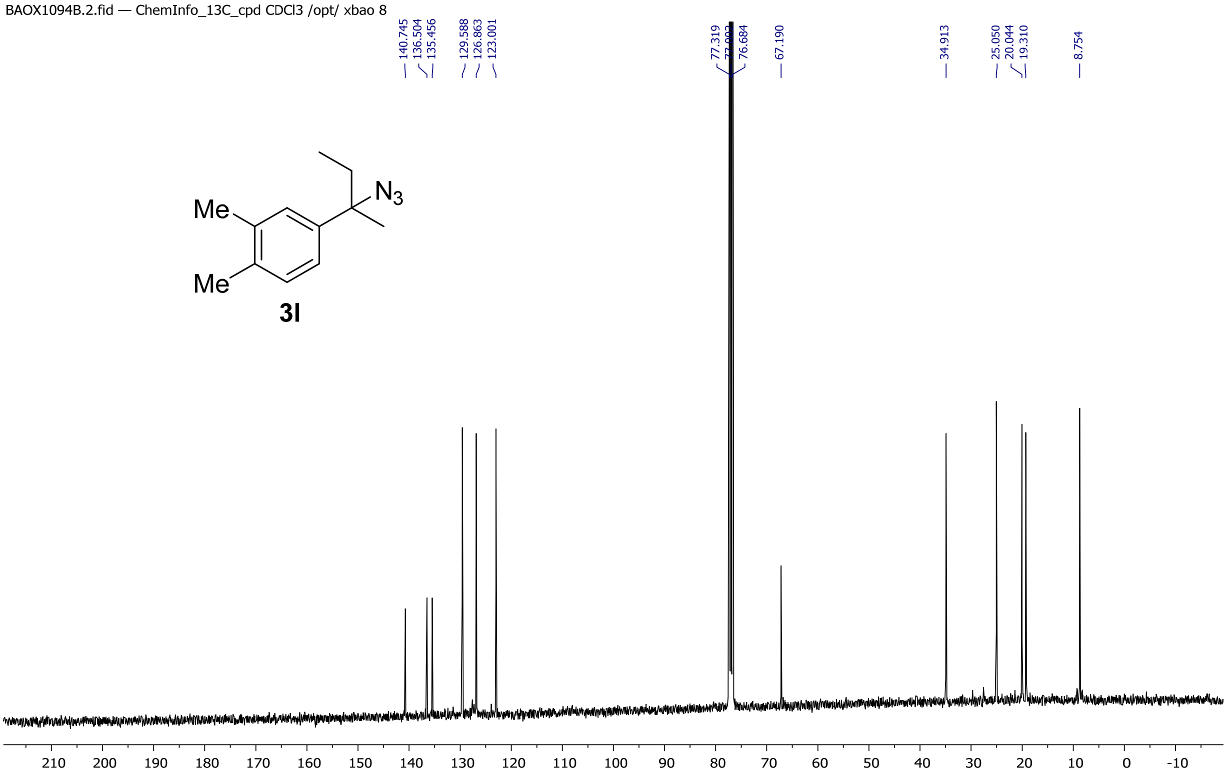

Supplementary Figure 50. ^{1}H and ^{13}C NMR spectra of 3l

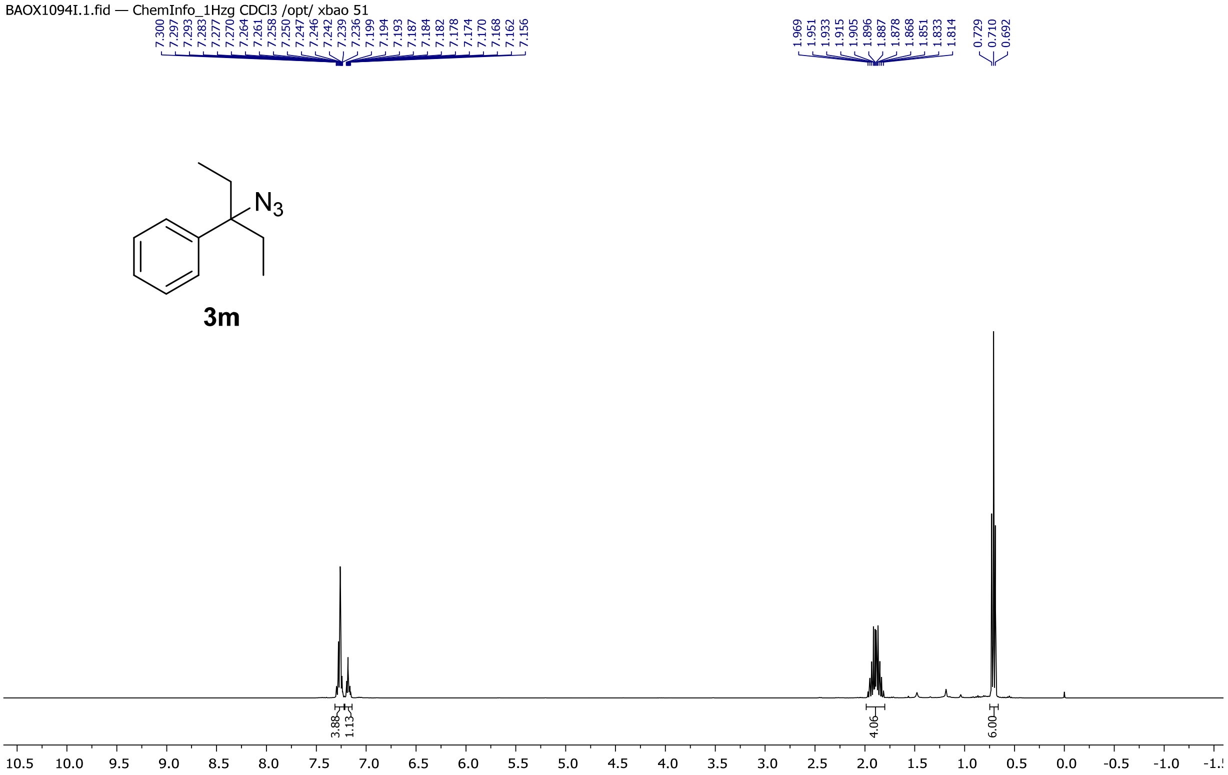

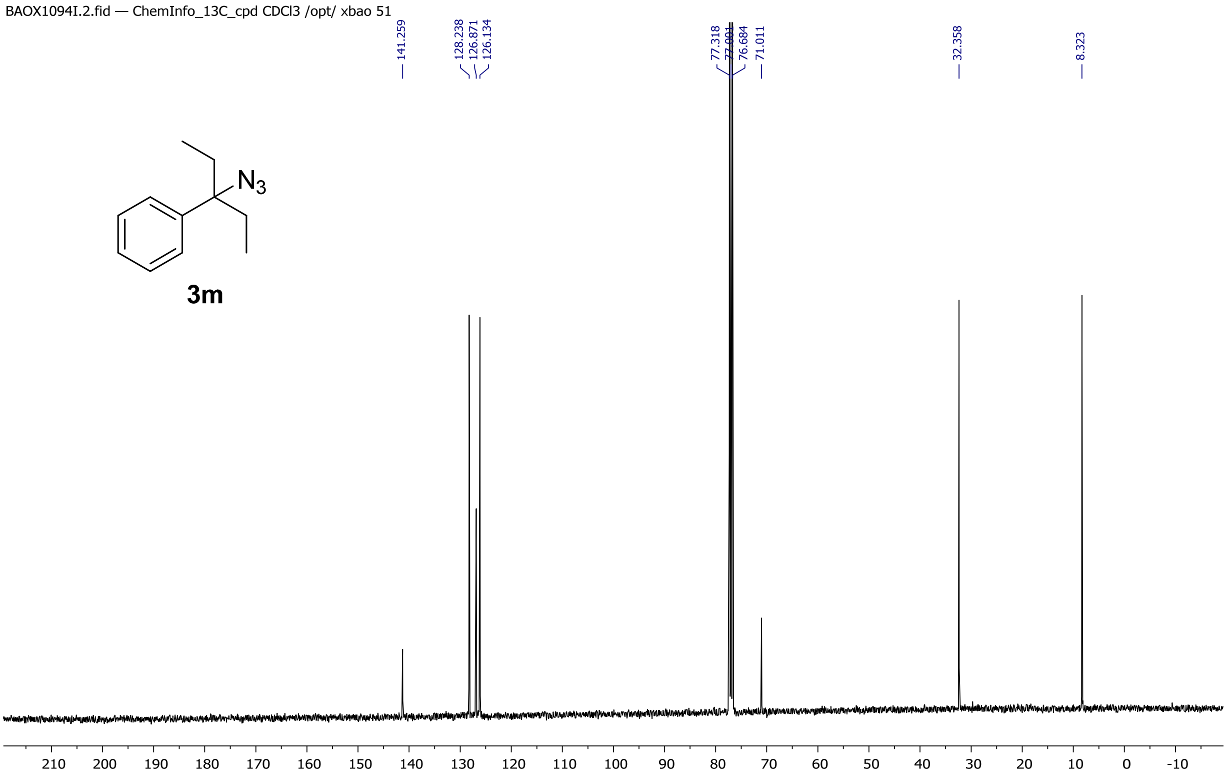

Supplementary Figure 51. ^{1}H and ^{13}C NMR spectra of 3m

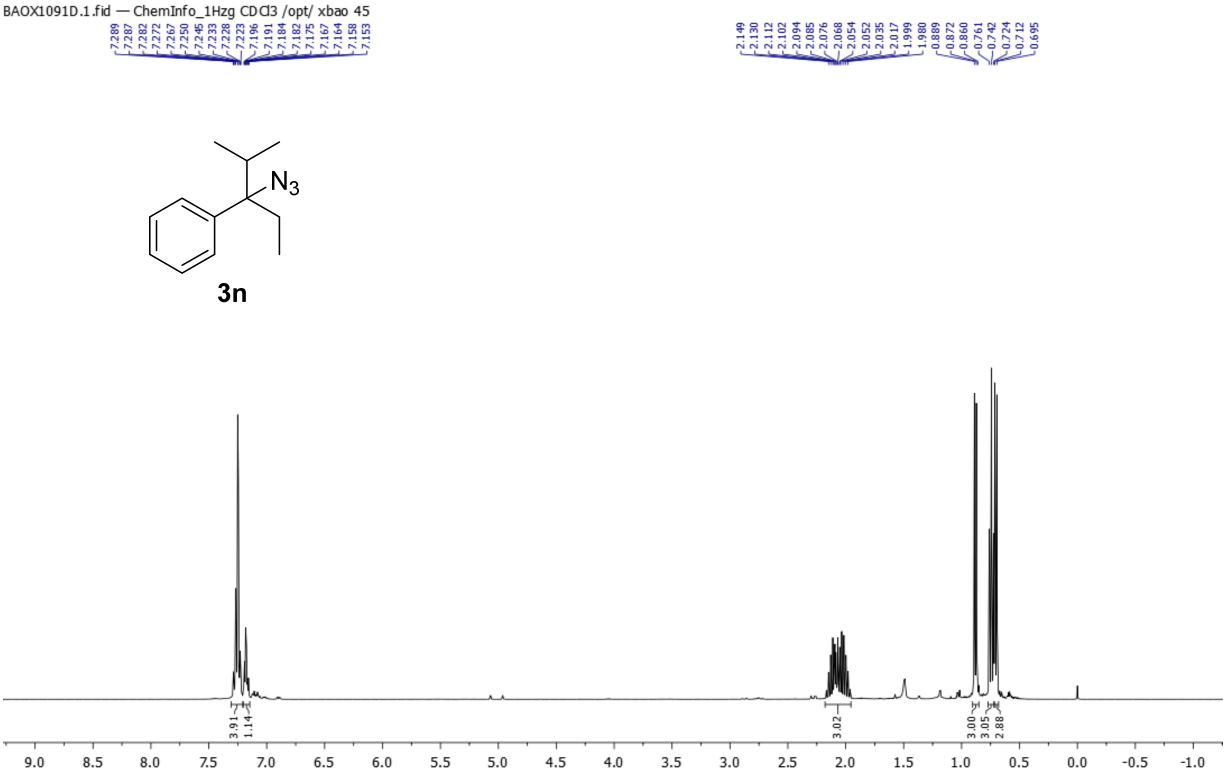

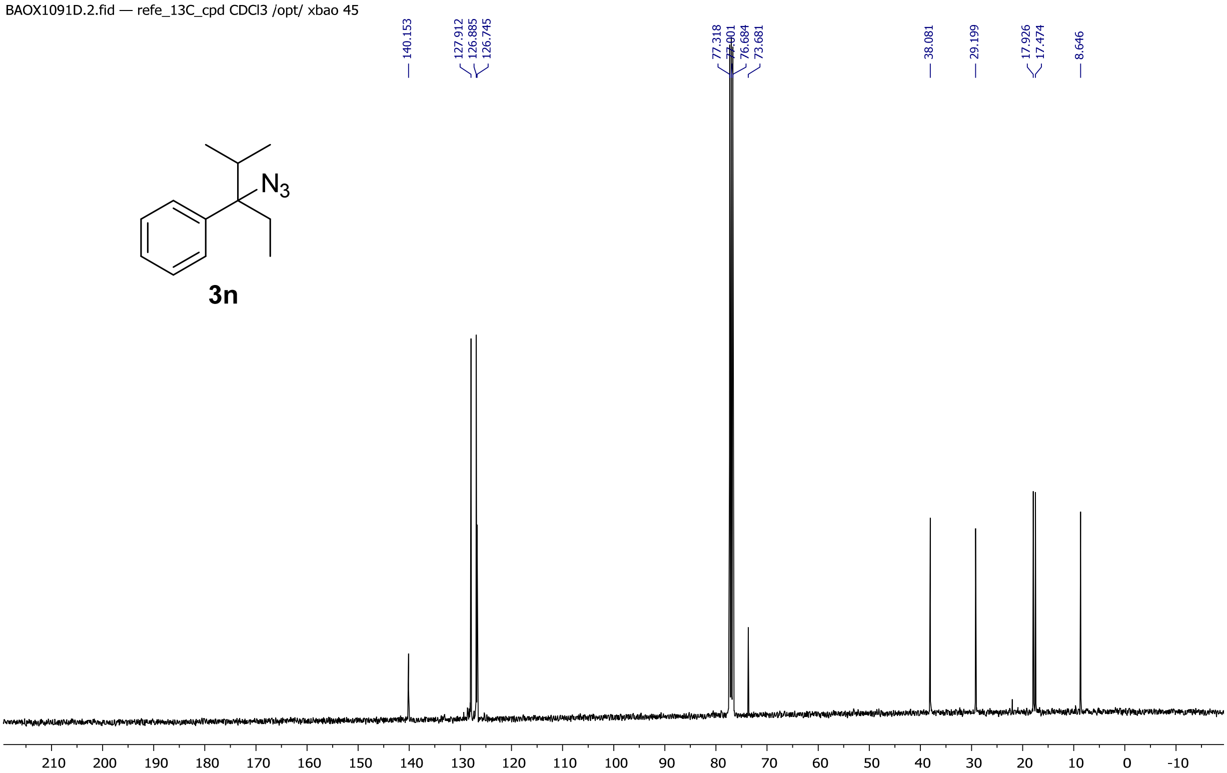

Supplementary Figure 52. ^{1}H and ^{13}C NMR spectra of 3n

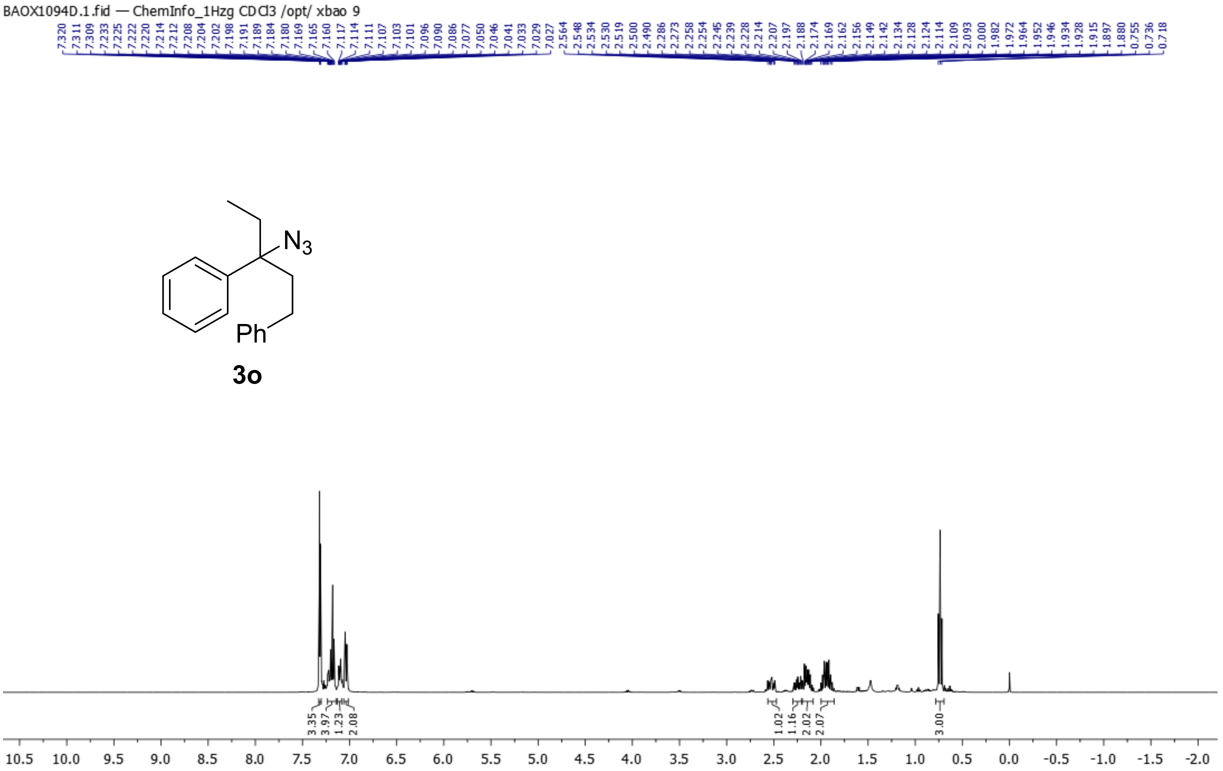

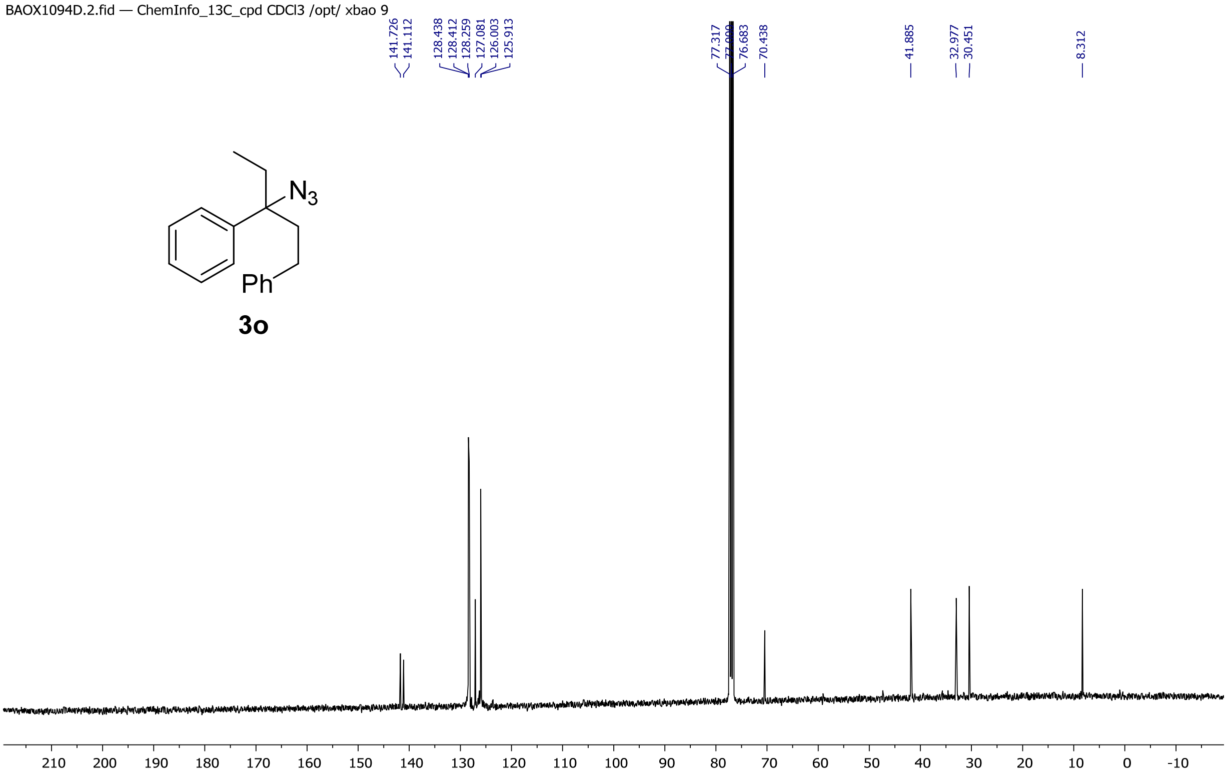

Supplementary Figure 53. ^{1}**H and** ^{13}**C NMR spectra of 3o**

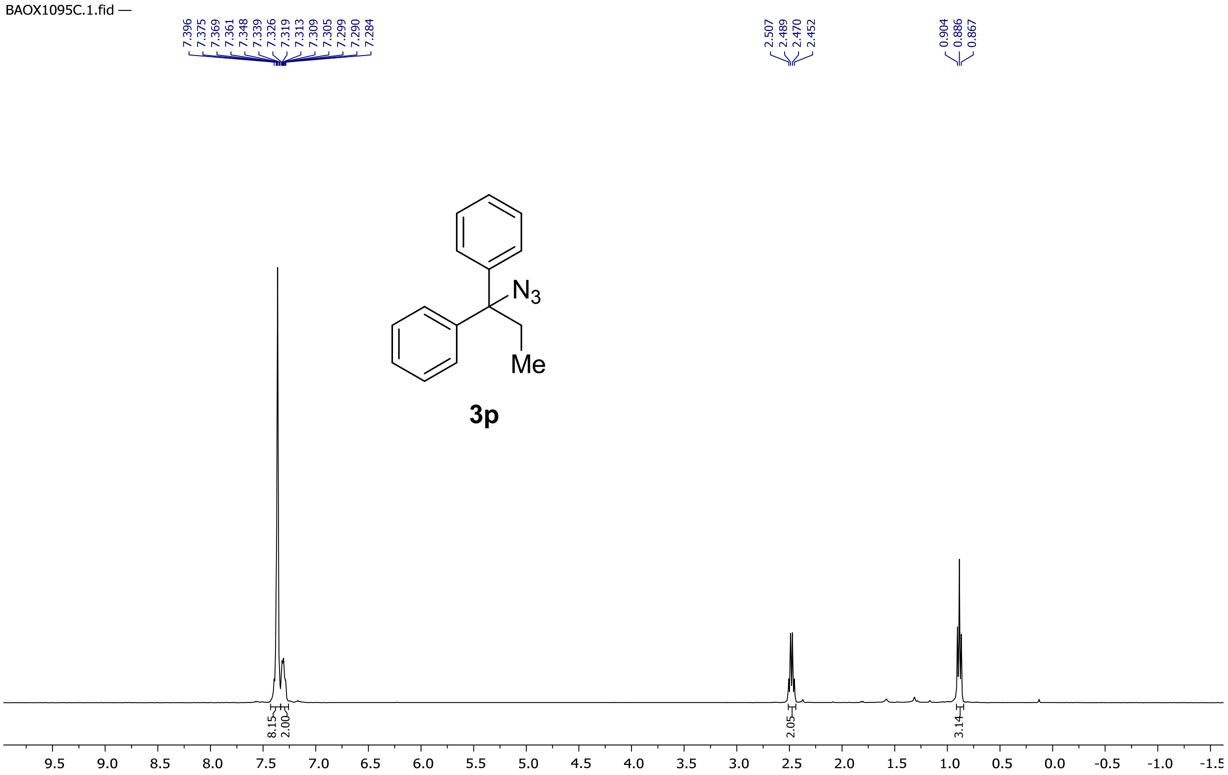

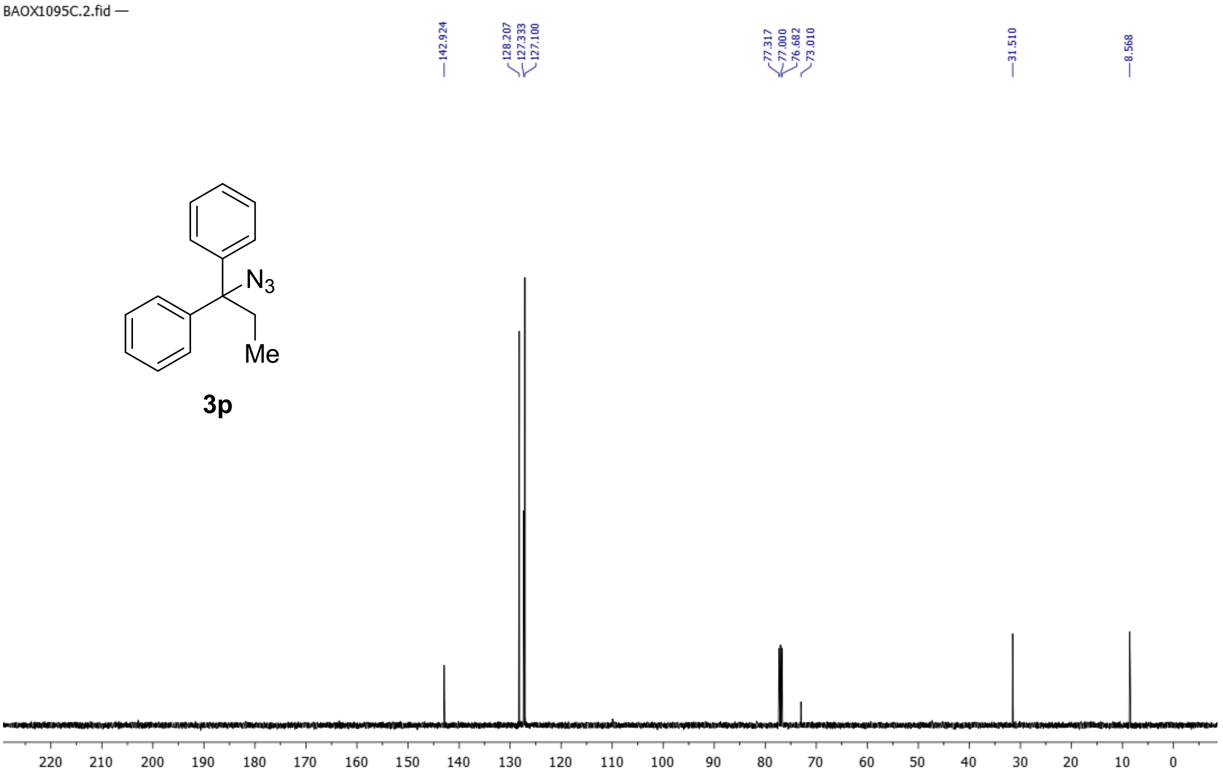

Supplementary Figure 54. ^{1}H and ^{13}C NMR spectra of 3p

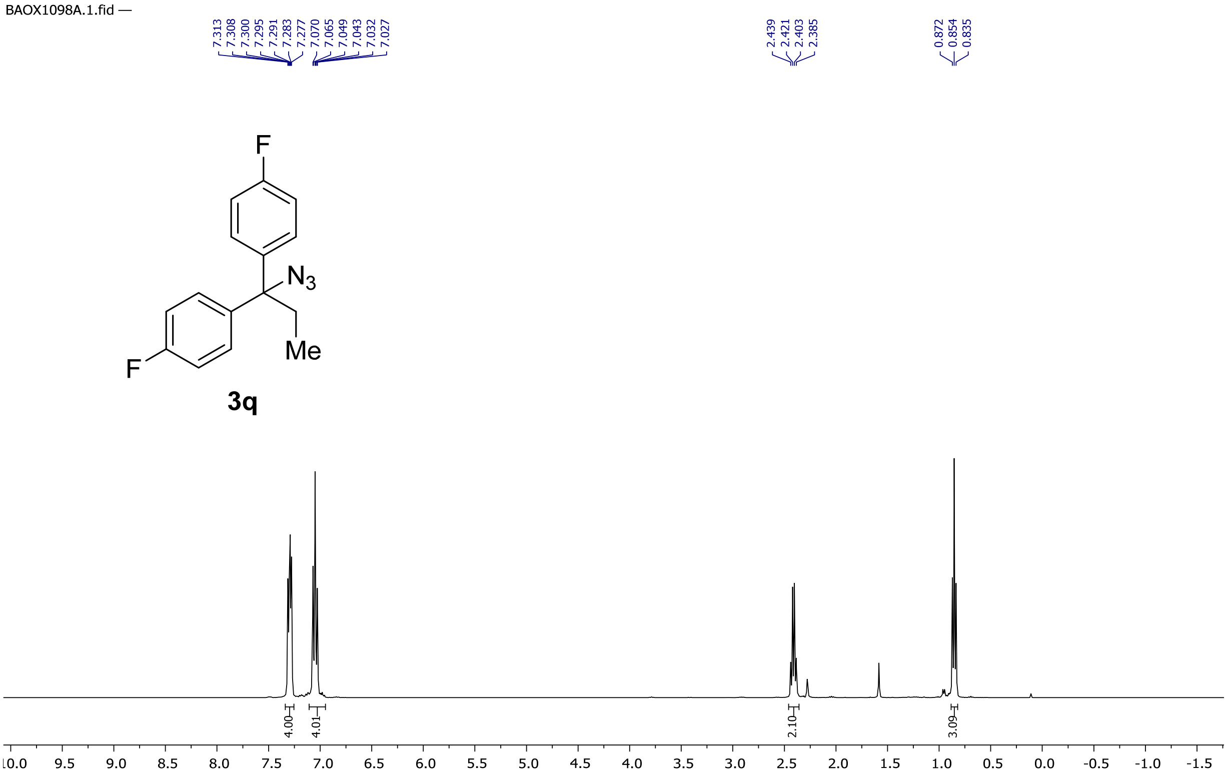

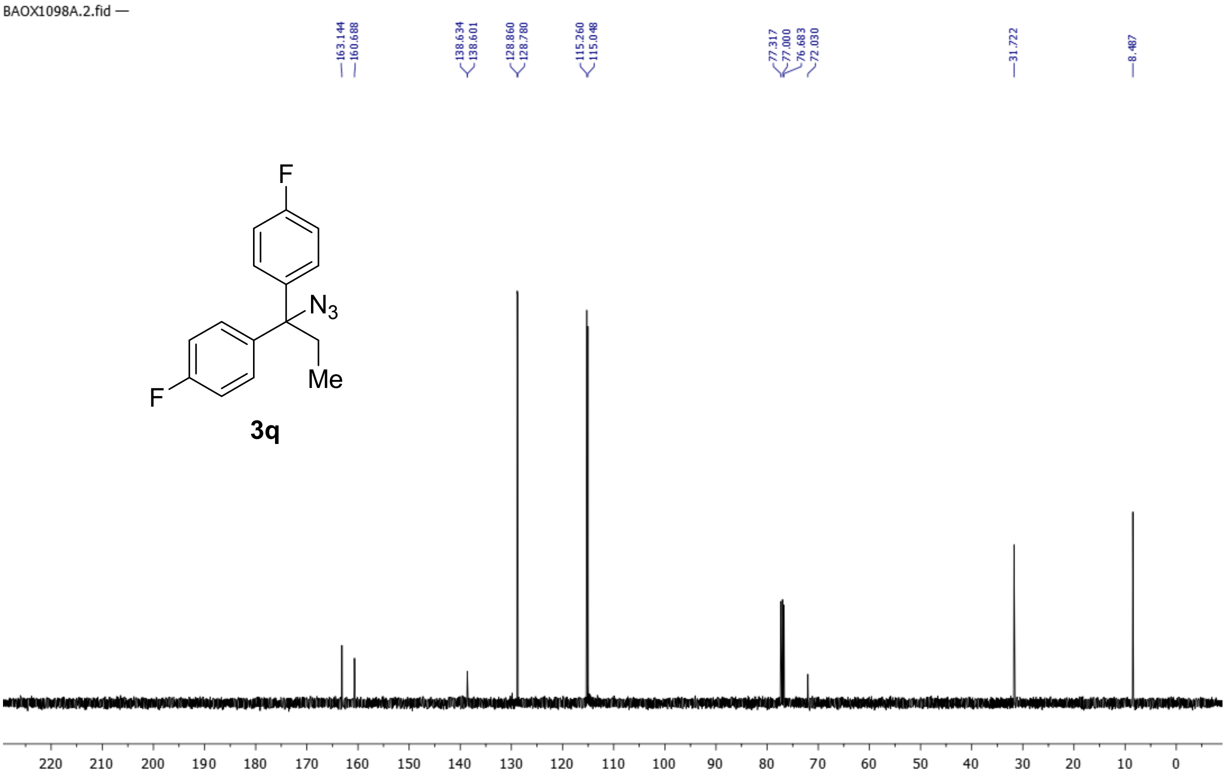

Supplementary Figure 55. ^{1}H and ^{13}C NMR spectra of 3q

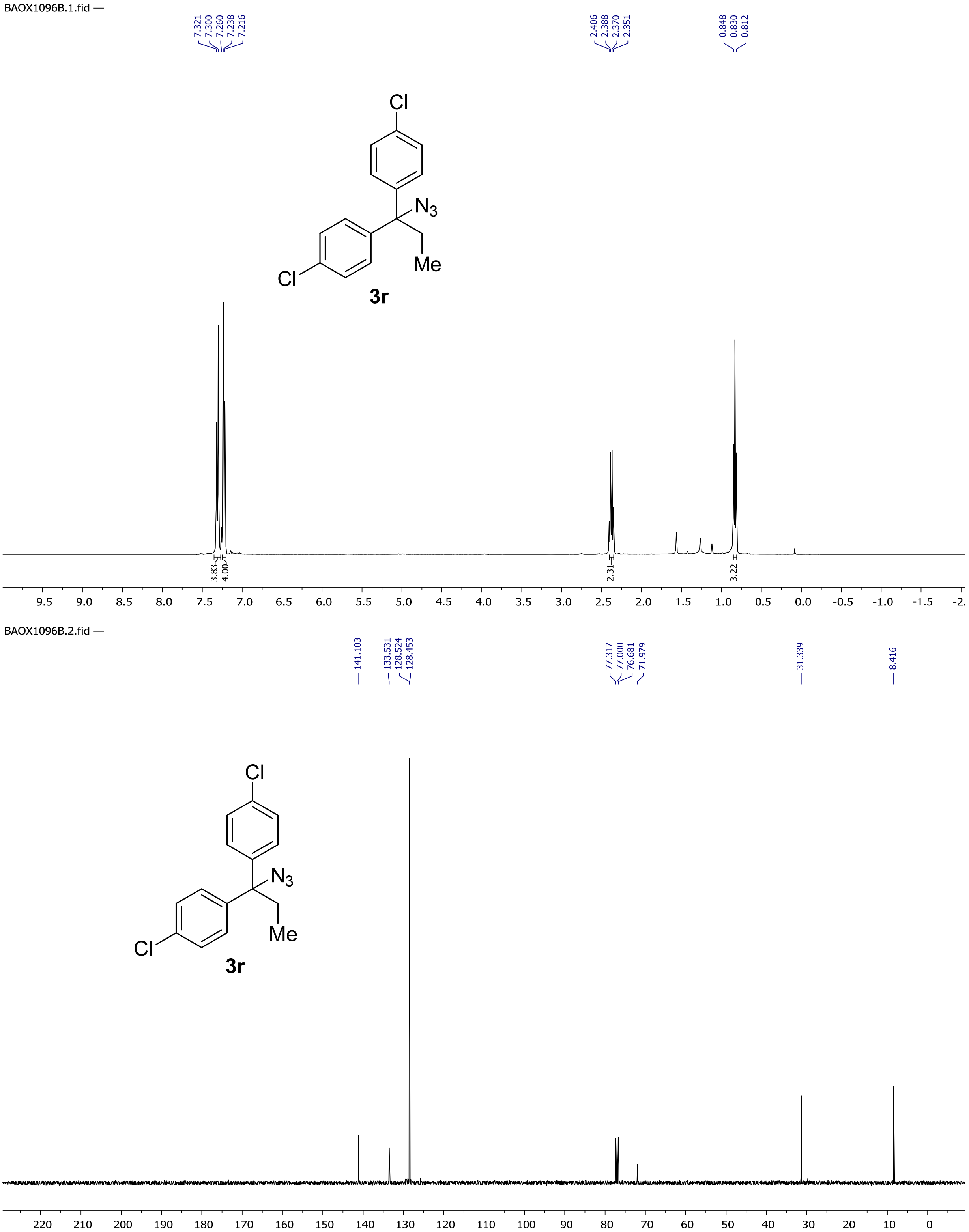

Supplementary Figure 56. ^{1}H and ^{13}C NMR spectra of 3r

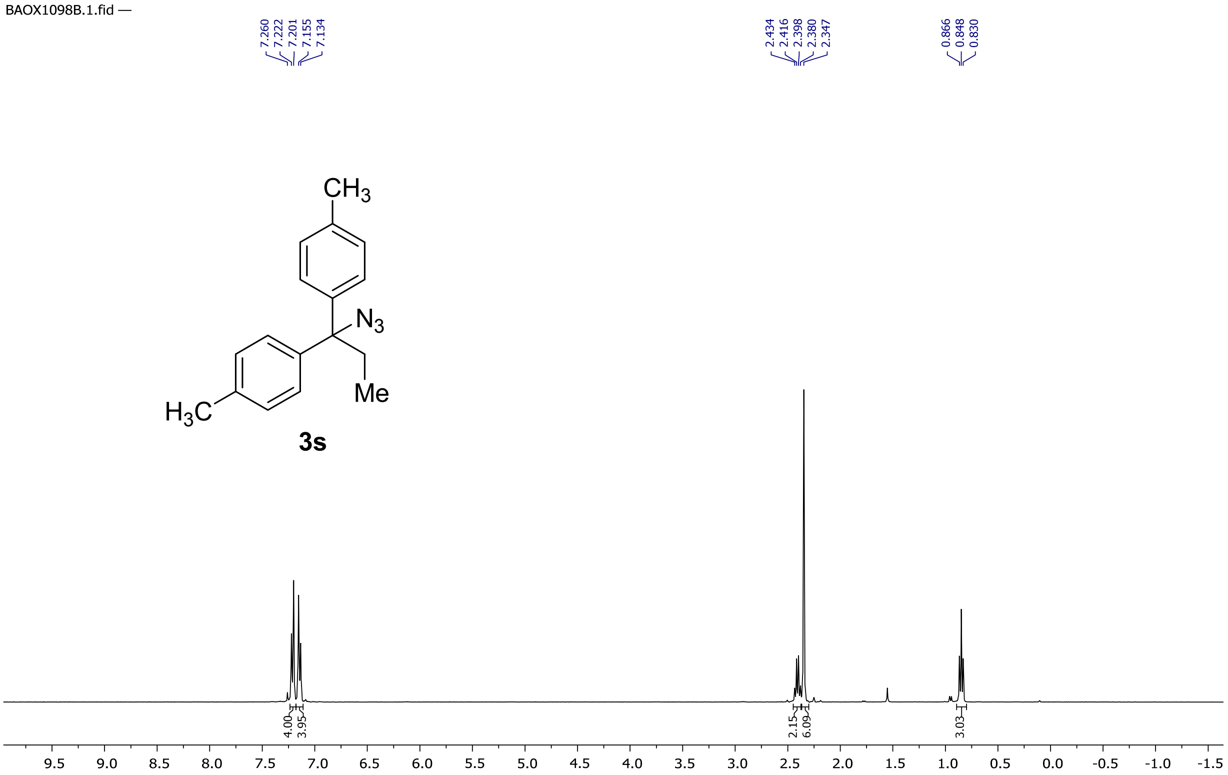

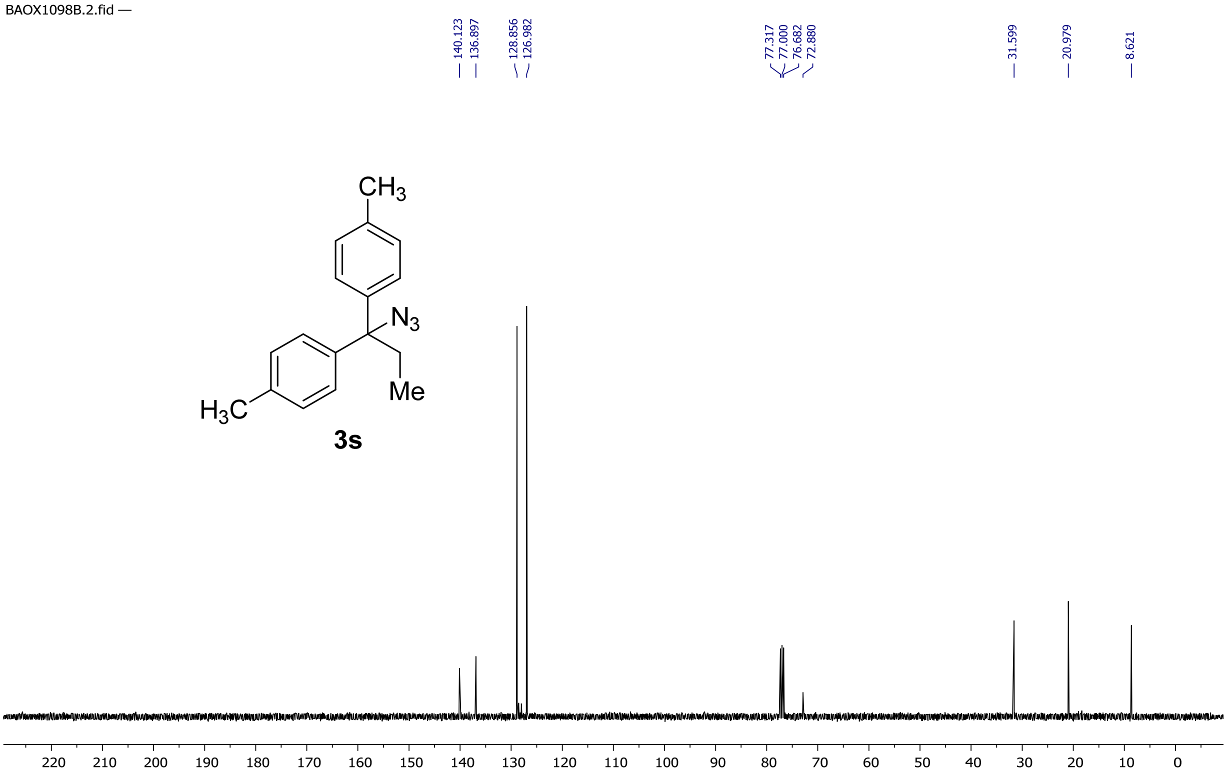

Supplementary Figure 57. ^{1}H and ^{13}C NMR spectra of 3s

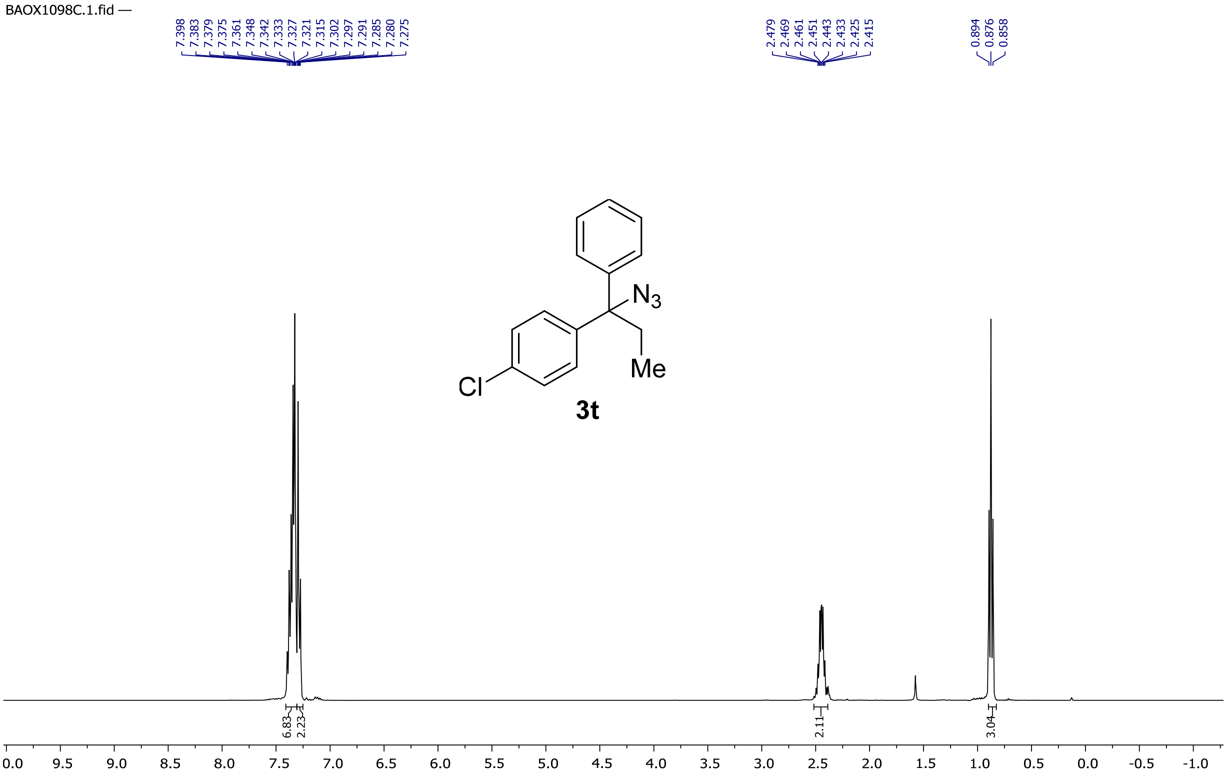

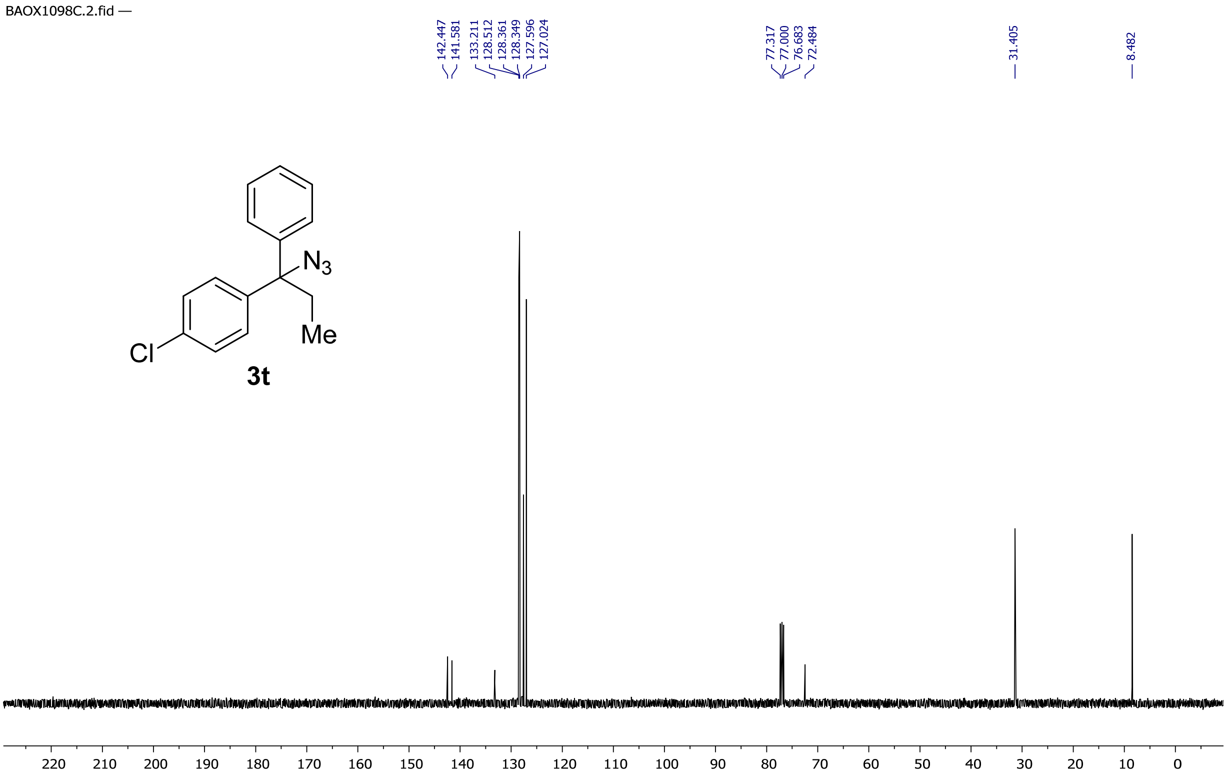

Supplementary Figure 58. ¹H and ¹³C NMR spectra of 3t

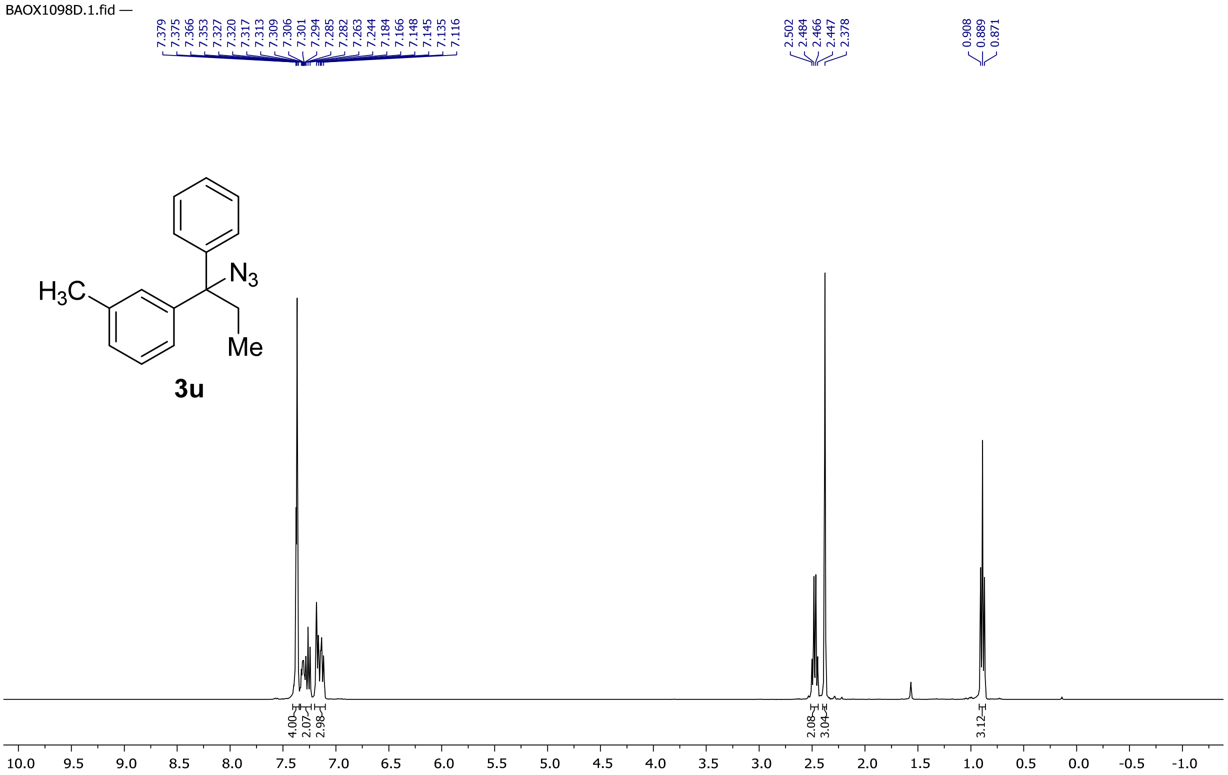

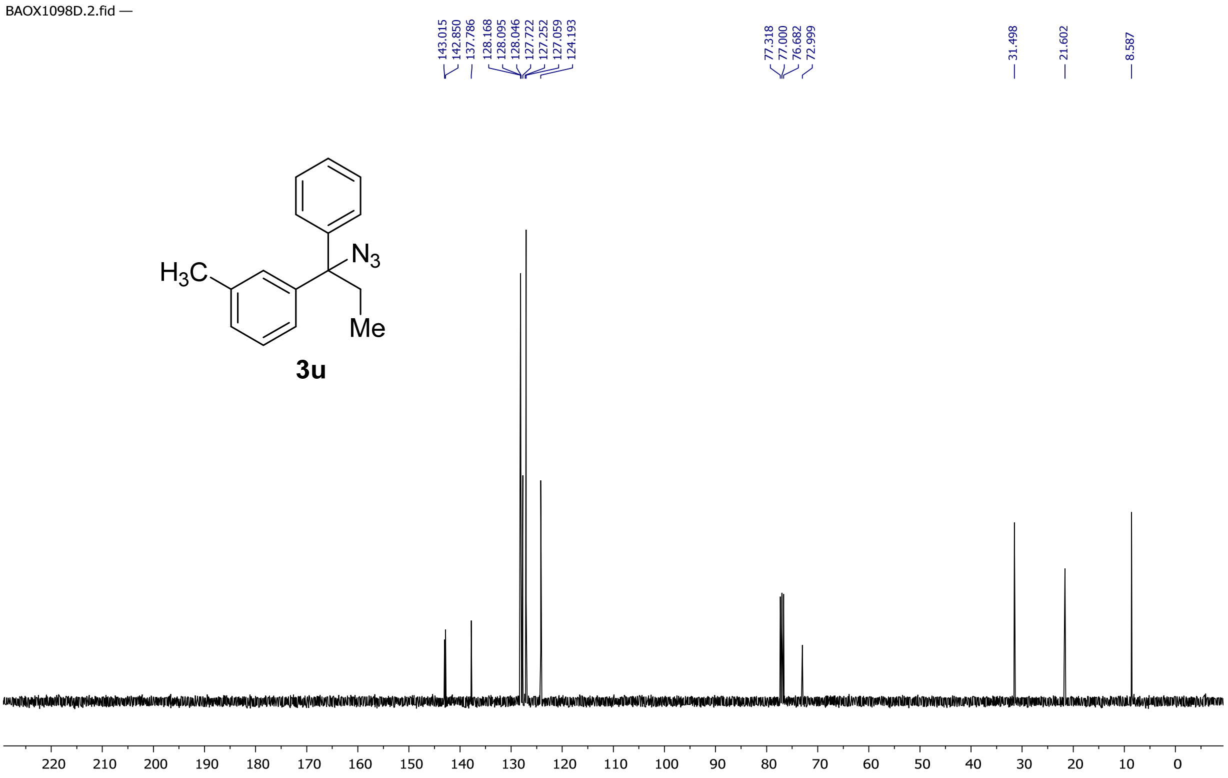

Supplementary Figure 59. ^{1}H and ^{13}C NMR spectra of 3u

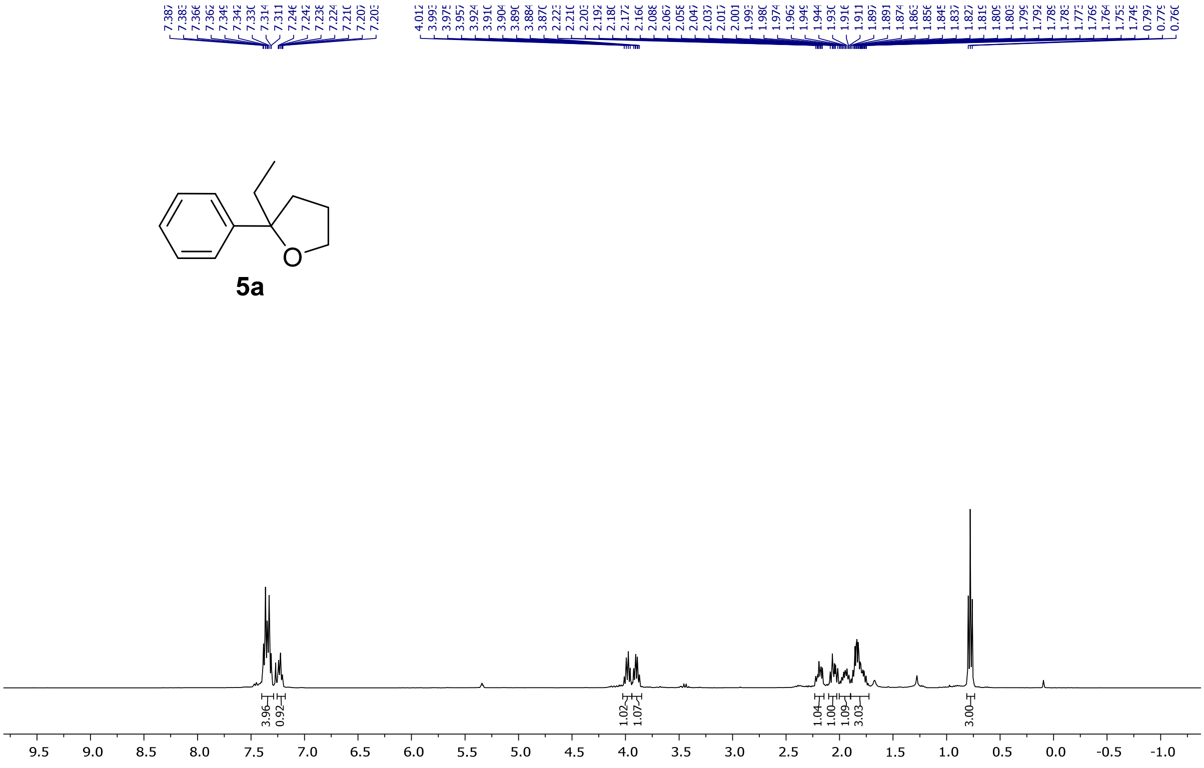

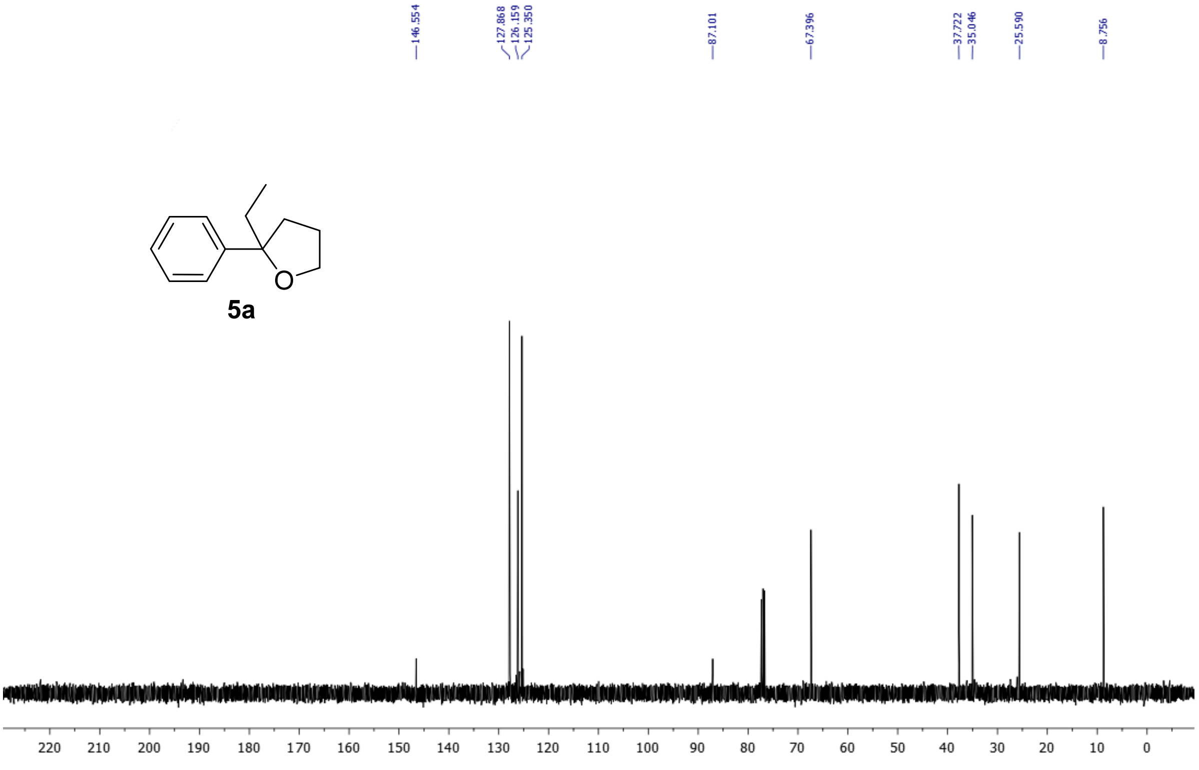

Supplementary Figure 60. ^{1}H and ^{13}C NMR spectra of 5a

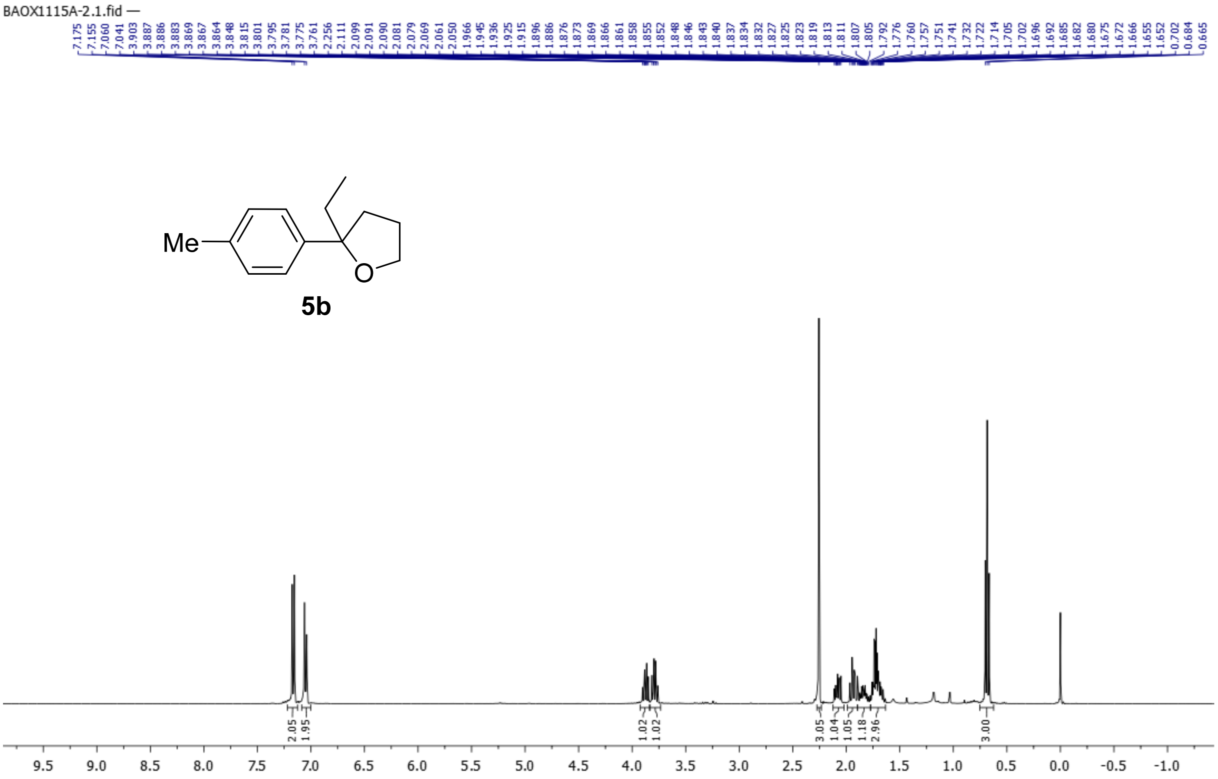

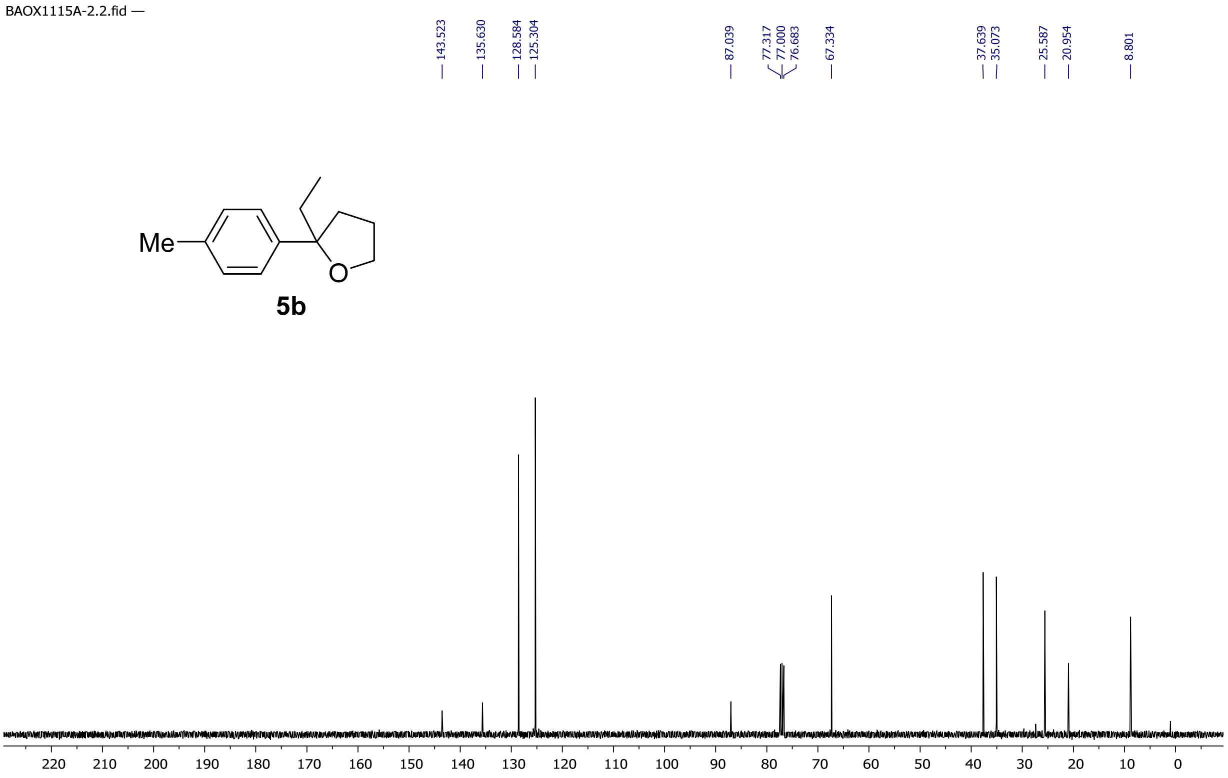

Supplementary Figure 61. ^{1}H and ^{13}C NMR spectra of 5b

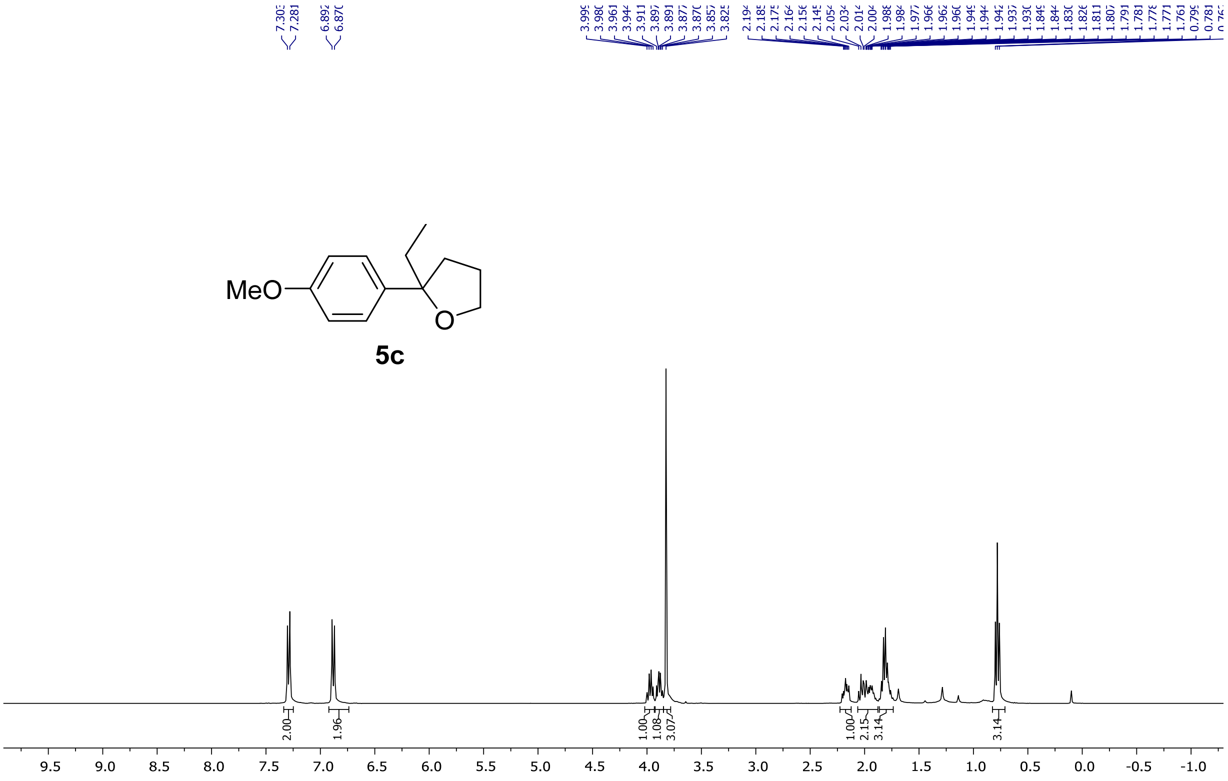

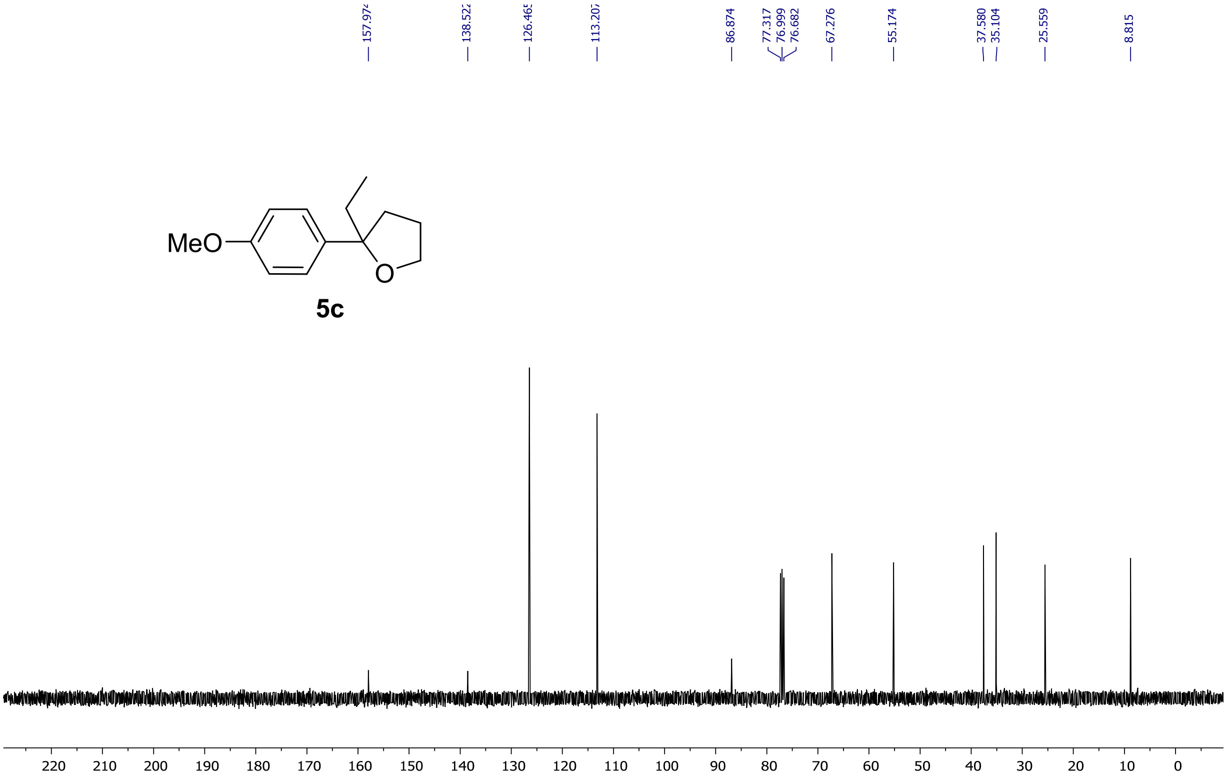

Supplementary Figure 62. ^{1}H and ^{13}C NMR spectra of 5c

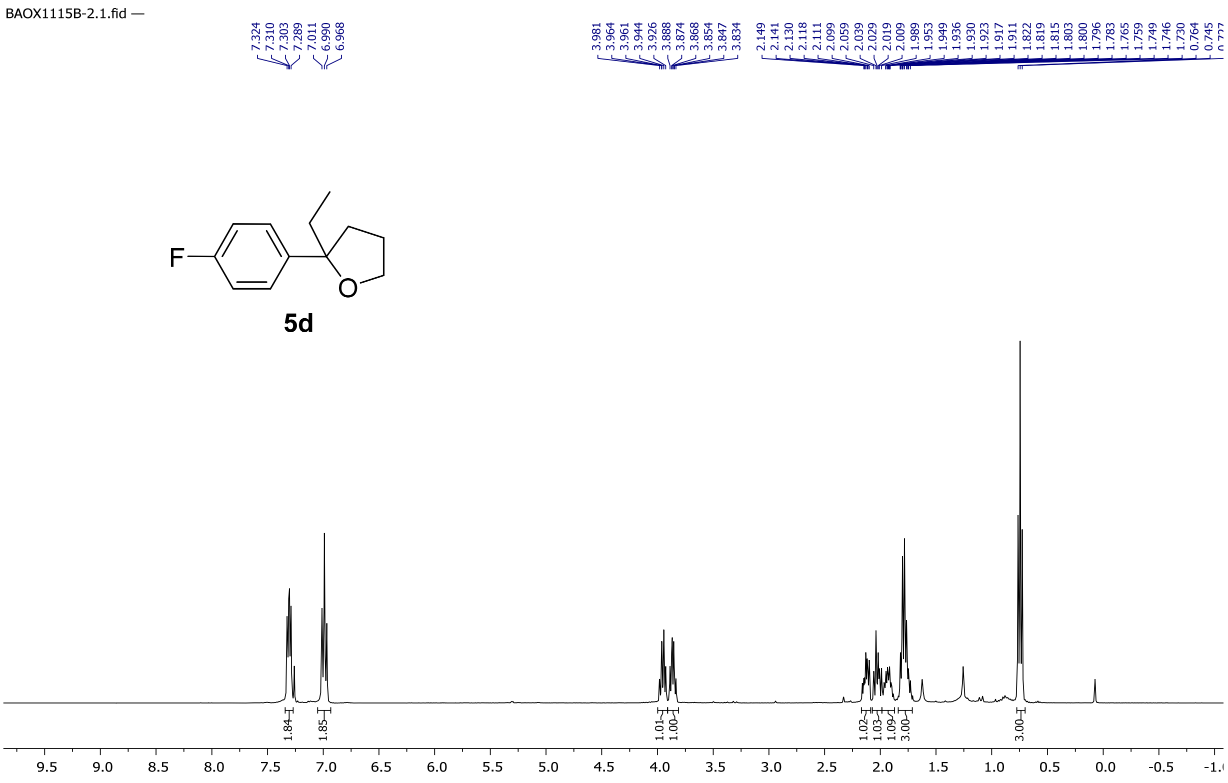

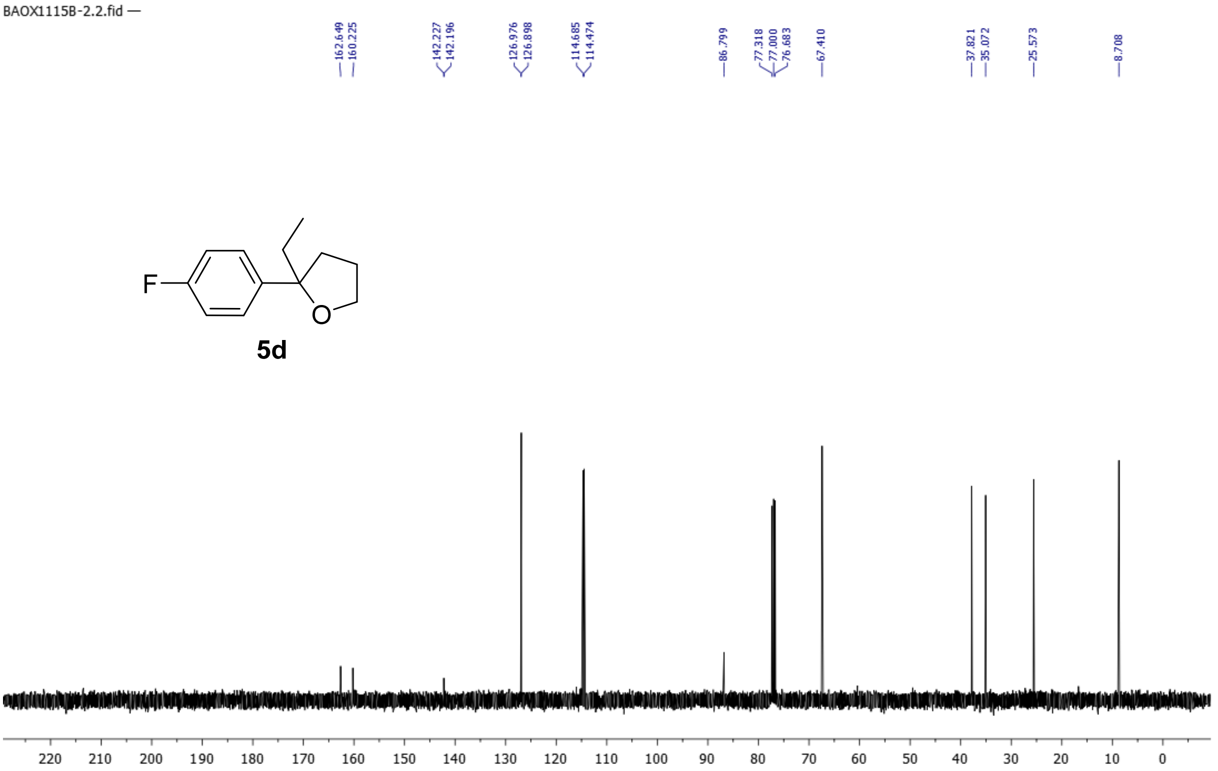

Supplementary Figure 63. ^{1}H and ^{13}C NMR spectra of 5d

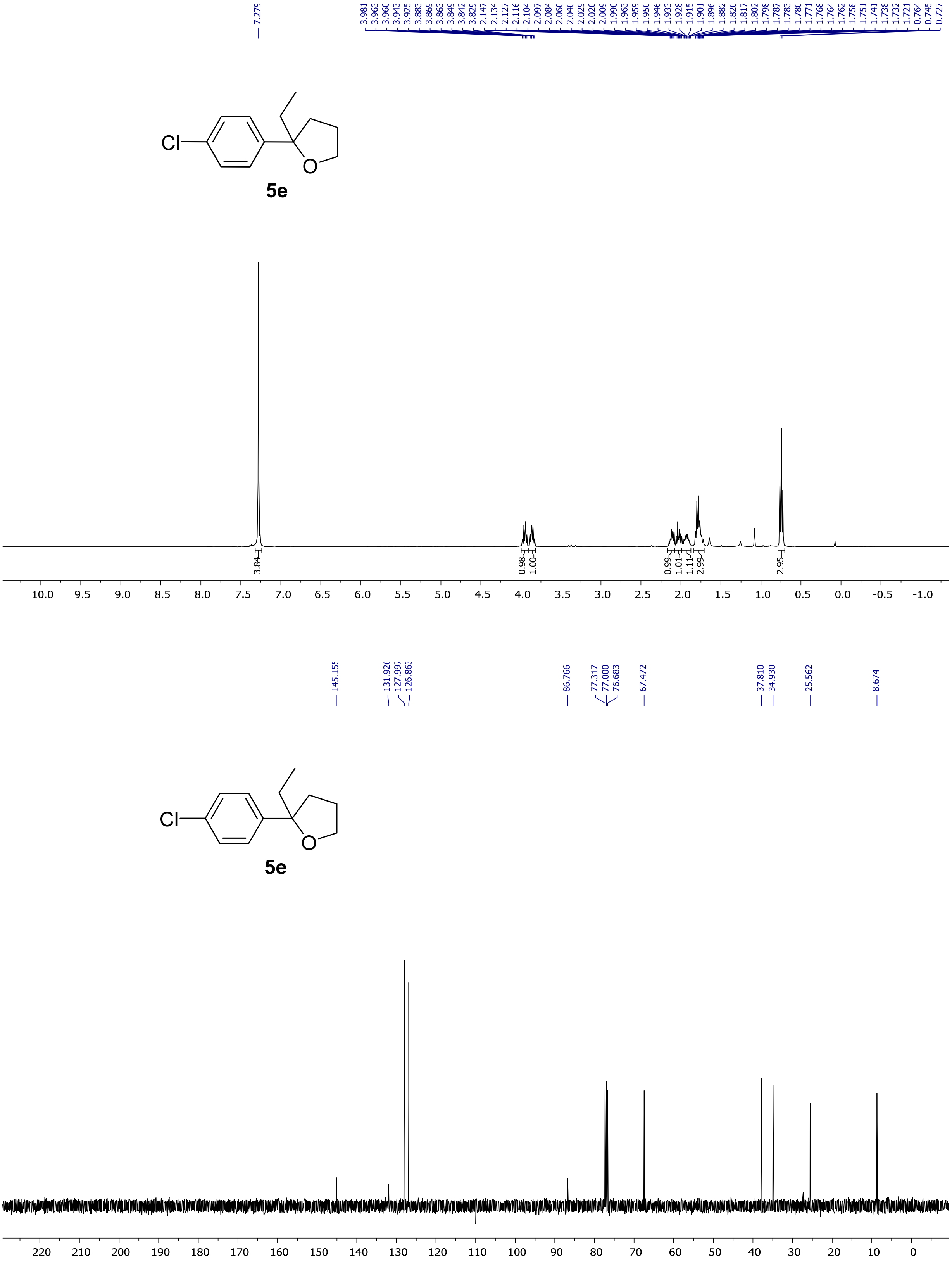

Supplementary Figure 64. ^{1}H and ^{13}C NMR spectra of 5e

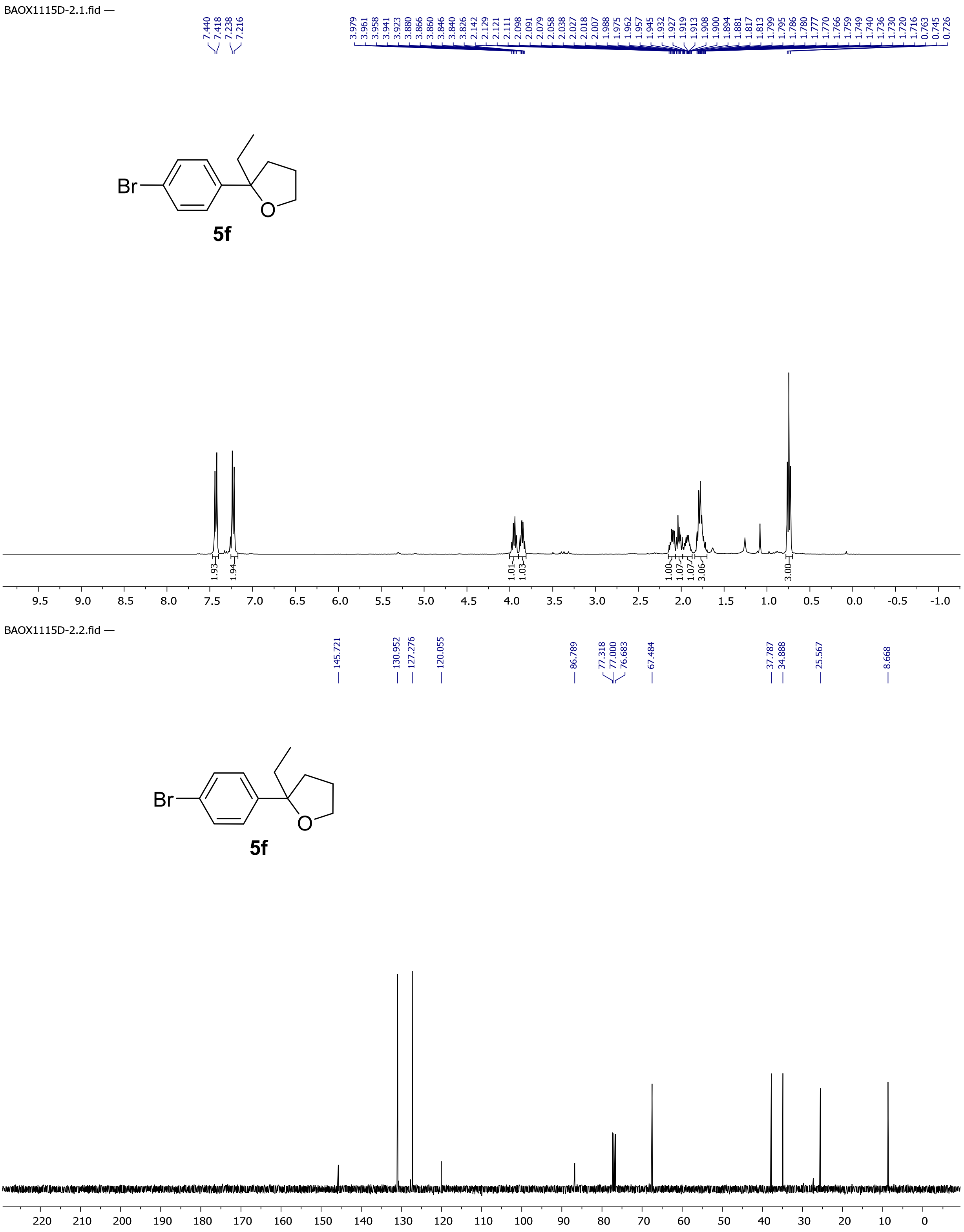

Supplementary Figure 65. ^{1}H **and** ^{13}C **NMR spectra of 5f**

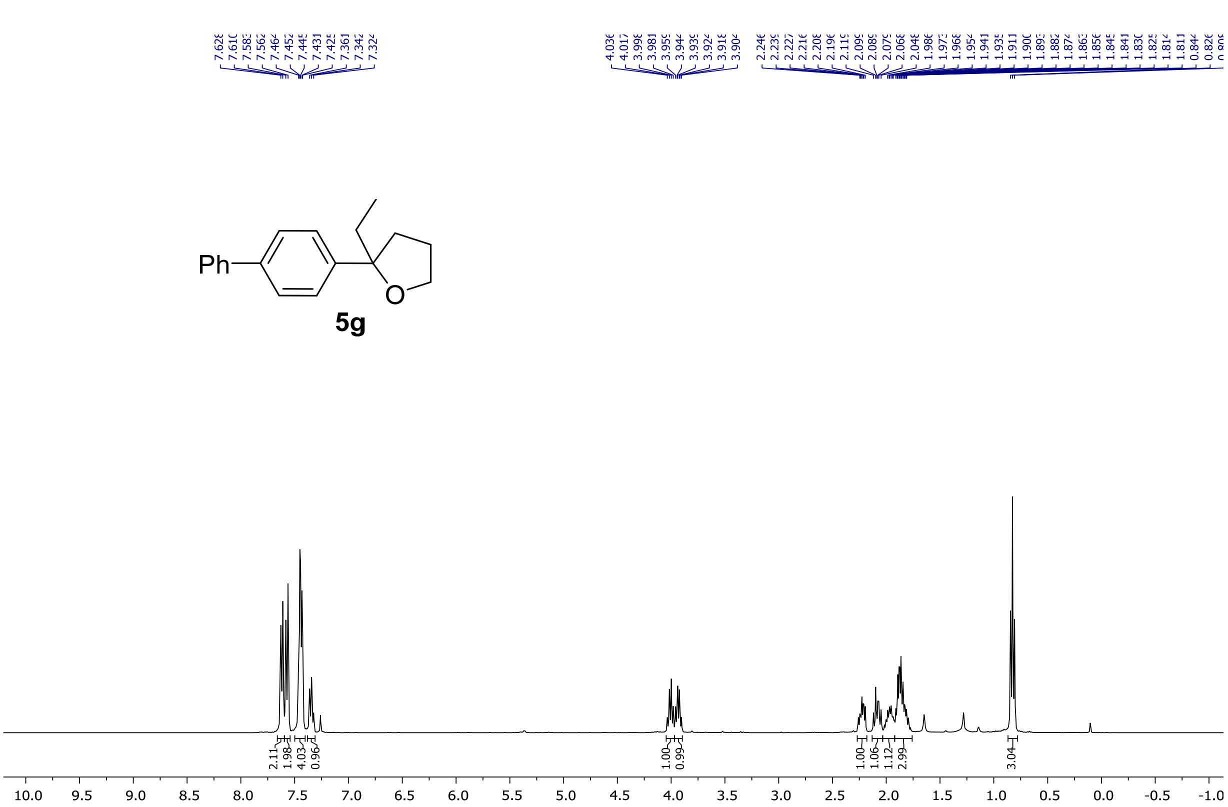

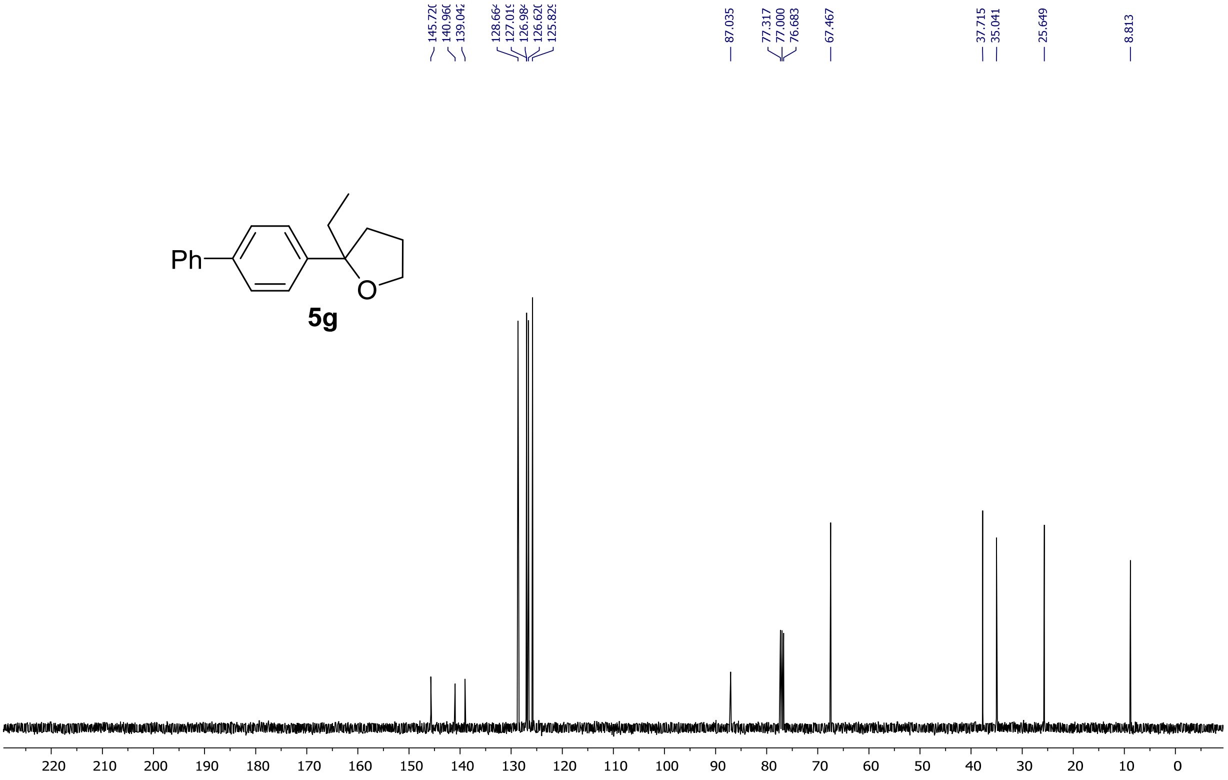

Supplementary Figure 66. ^{1}H and ^{13}C NMR spectra of 5g

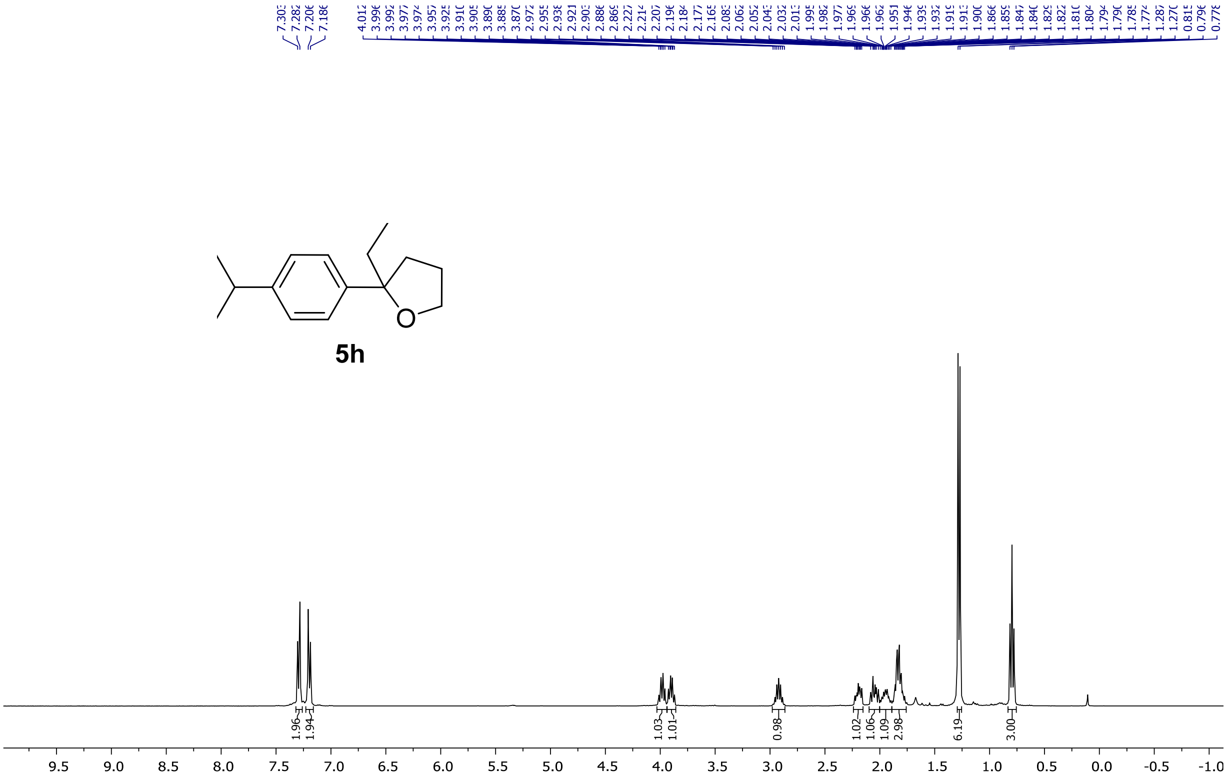

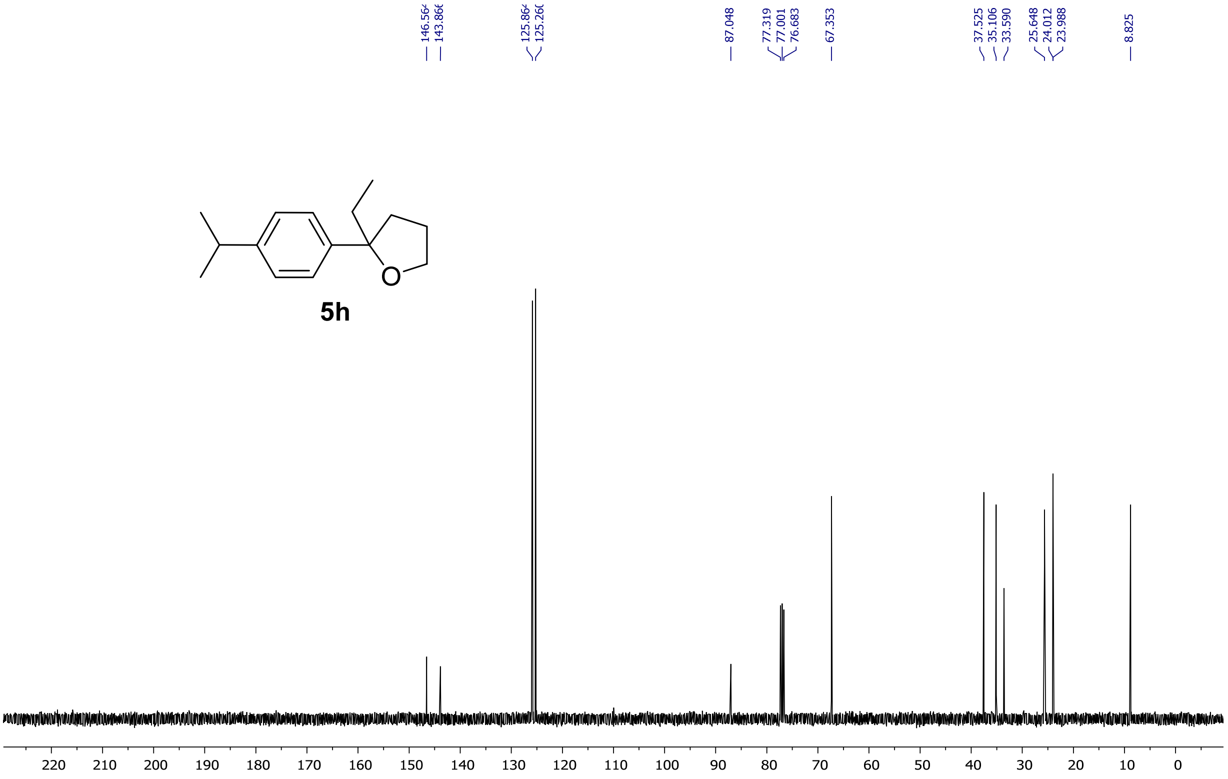

Supplementary Figure 67. ¹H and ¹³C NMR spectra of 5h

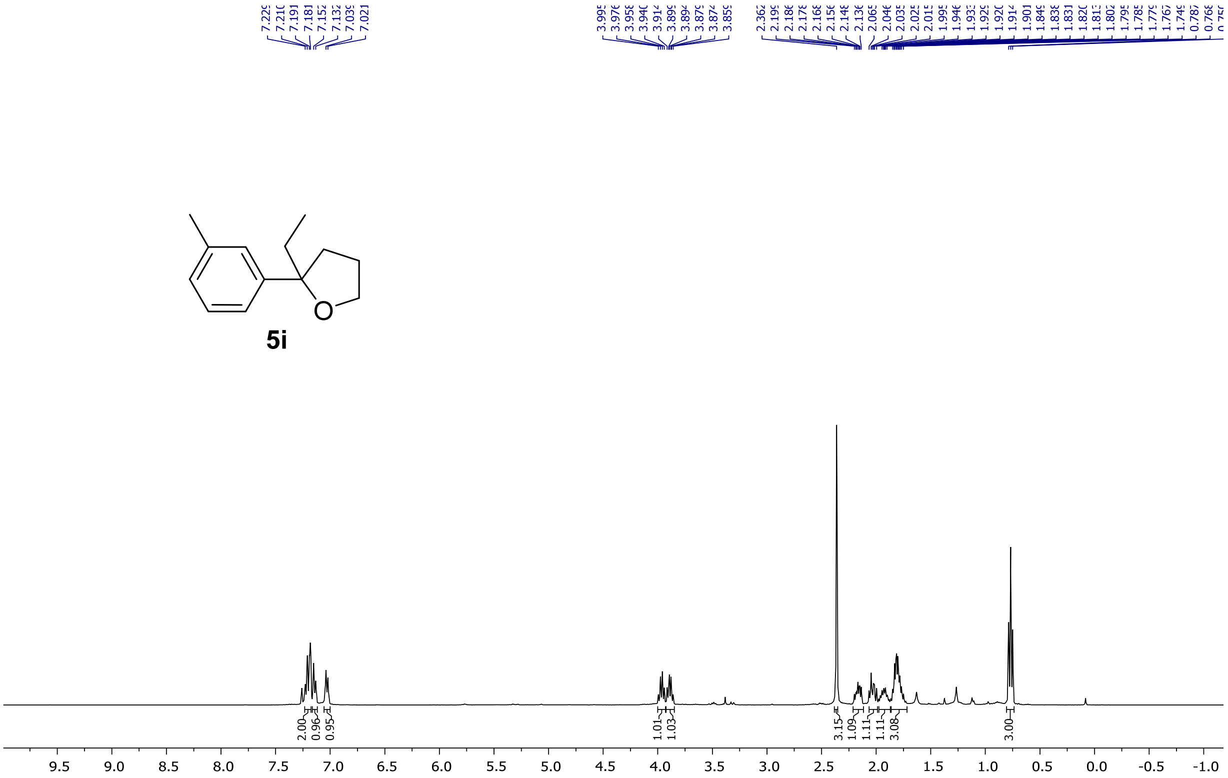

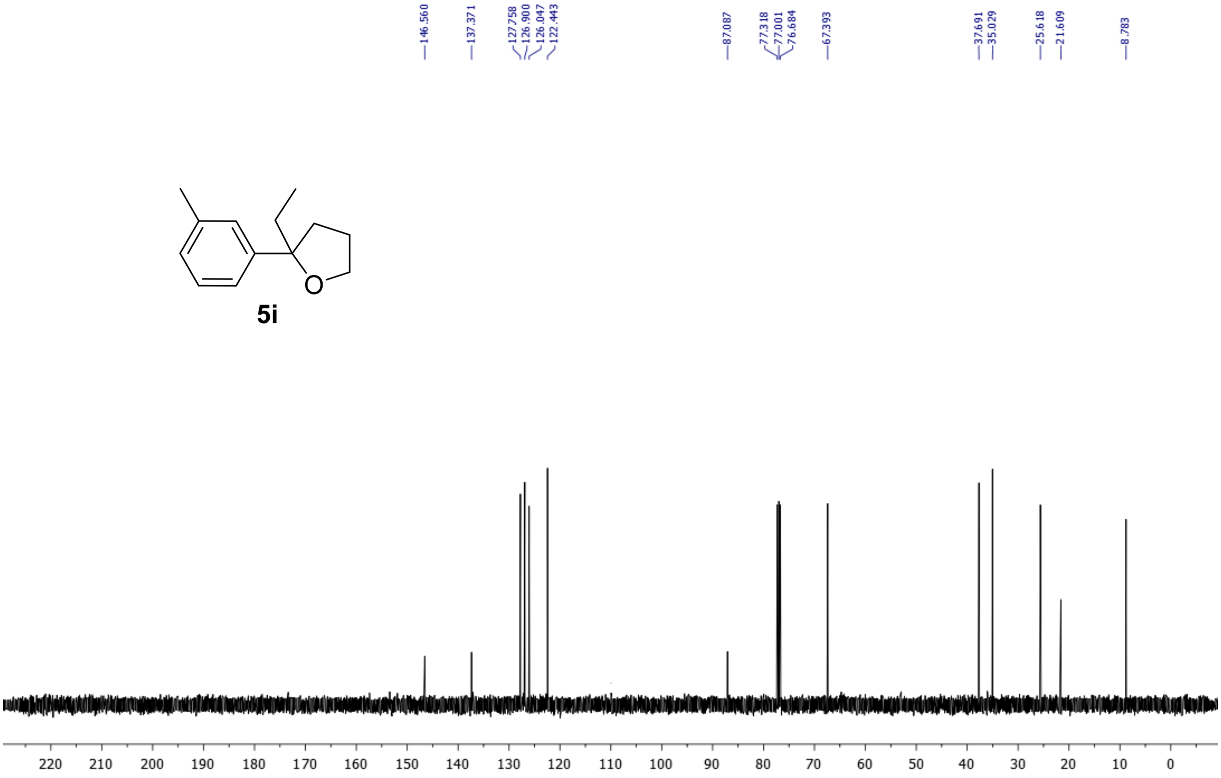

Supplementary Figure 68. ^{1}H and ^{13}C NMR spectra of 5i

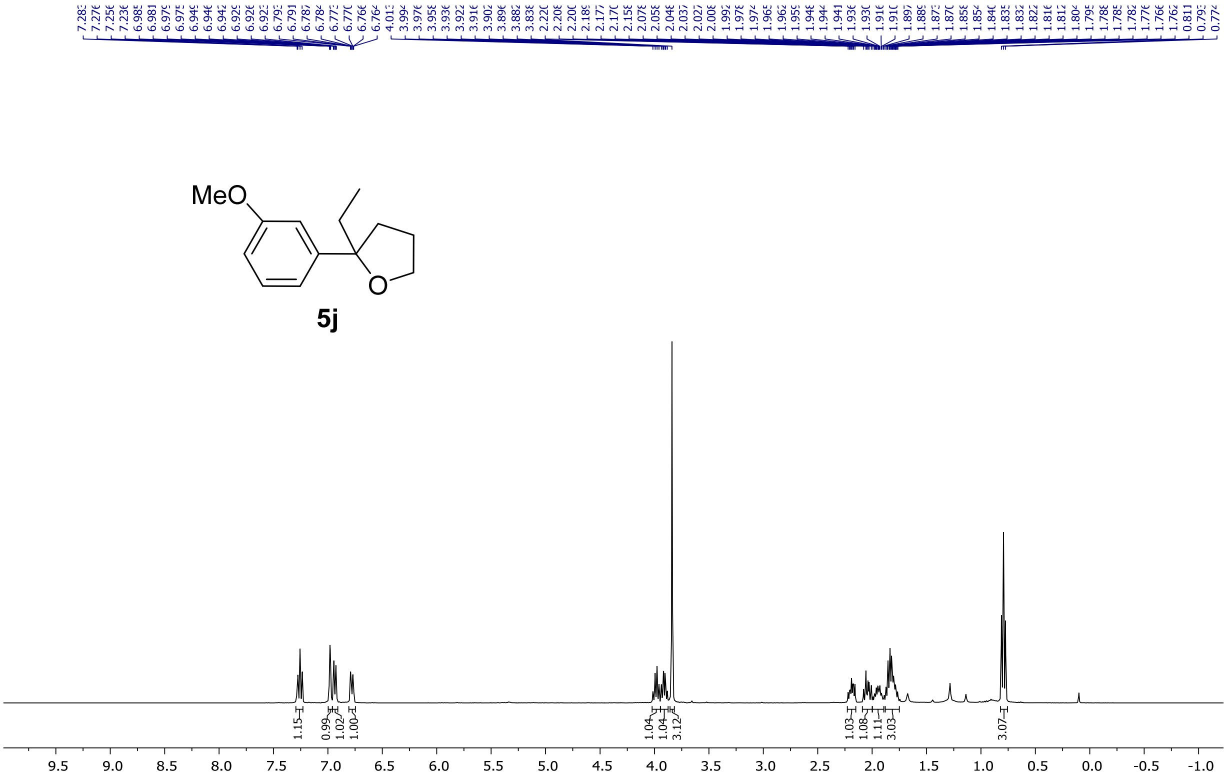

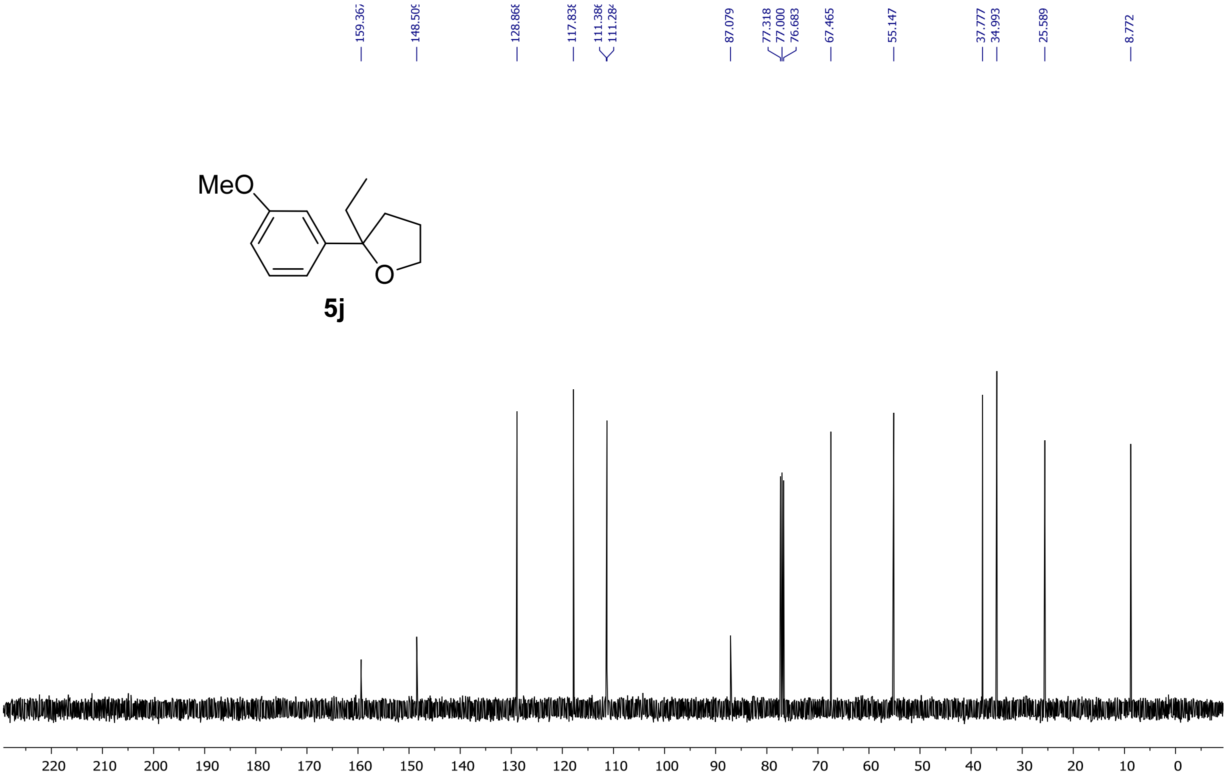

Supplementary Figure 69. ^1H and ^{13}C NMR spectra of 5j

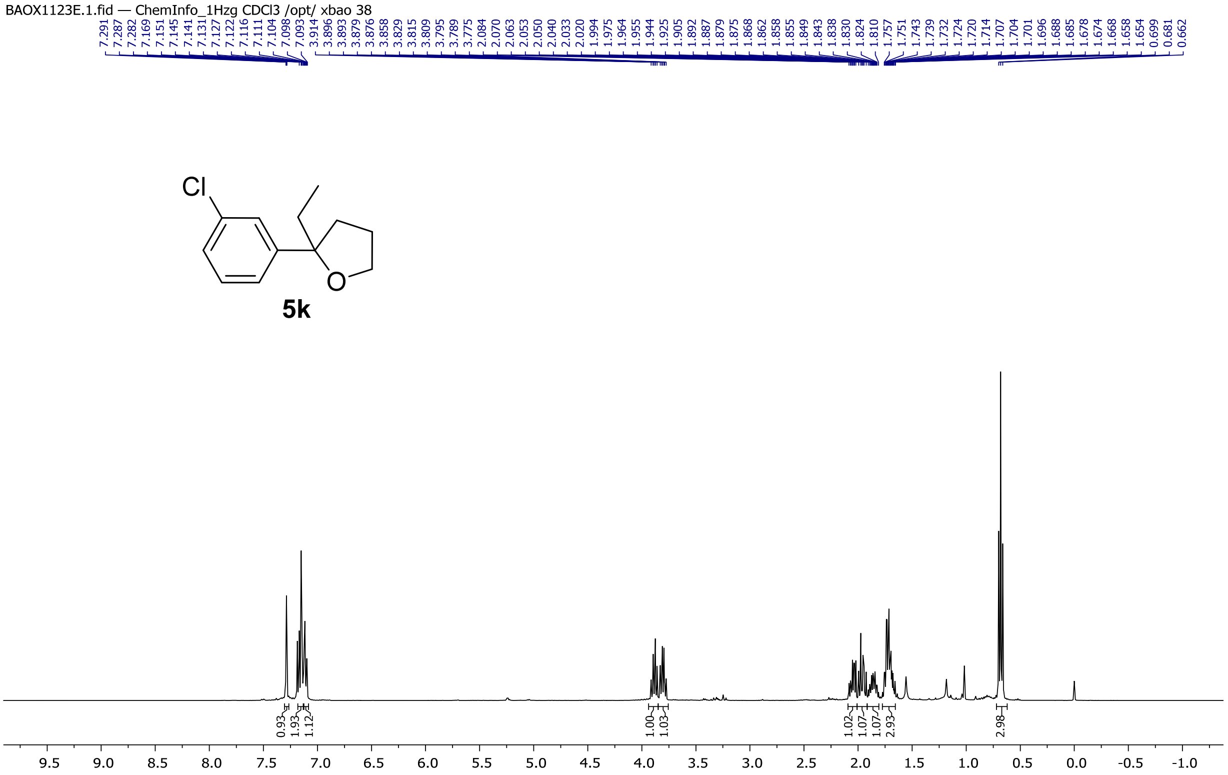

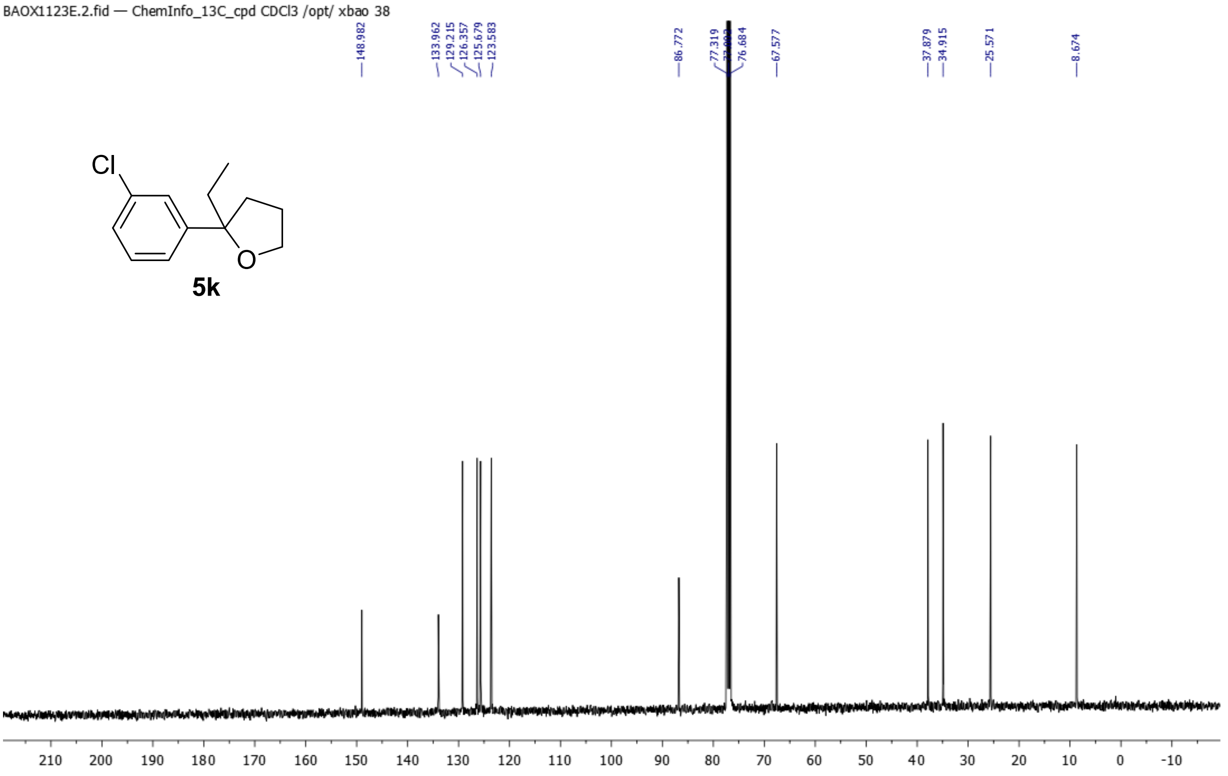

Supplementary Figure 70. ^{1}H and ^{13}C NMR spectra of 5k

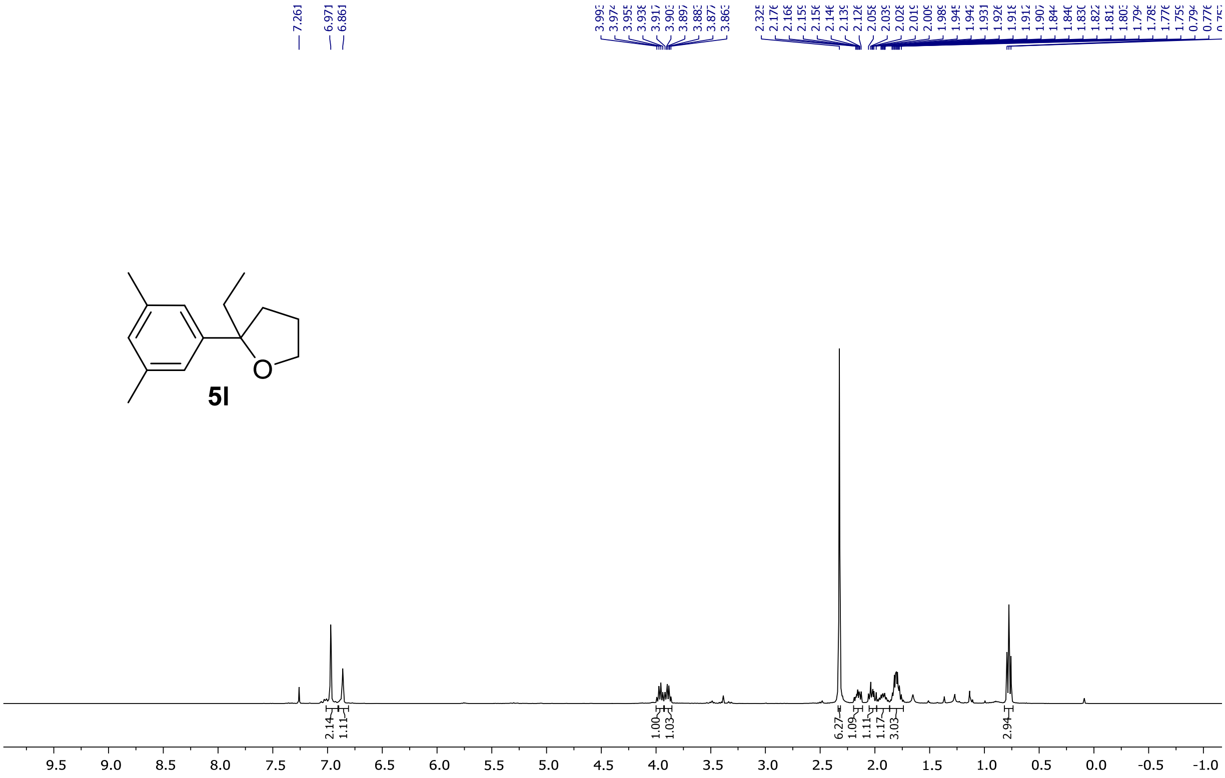

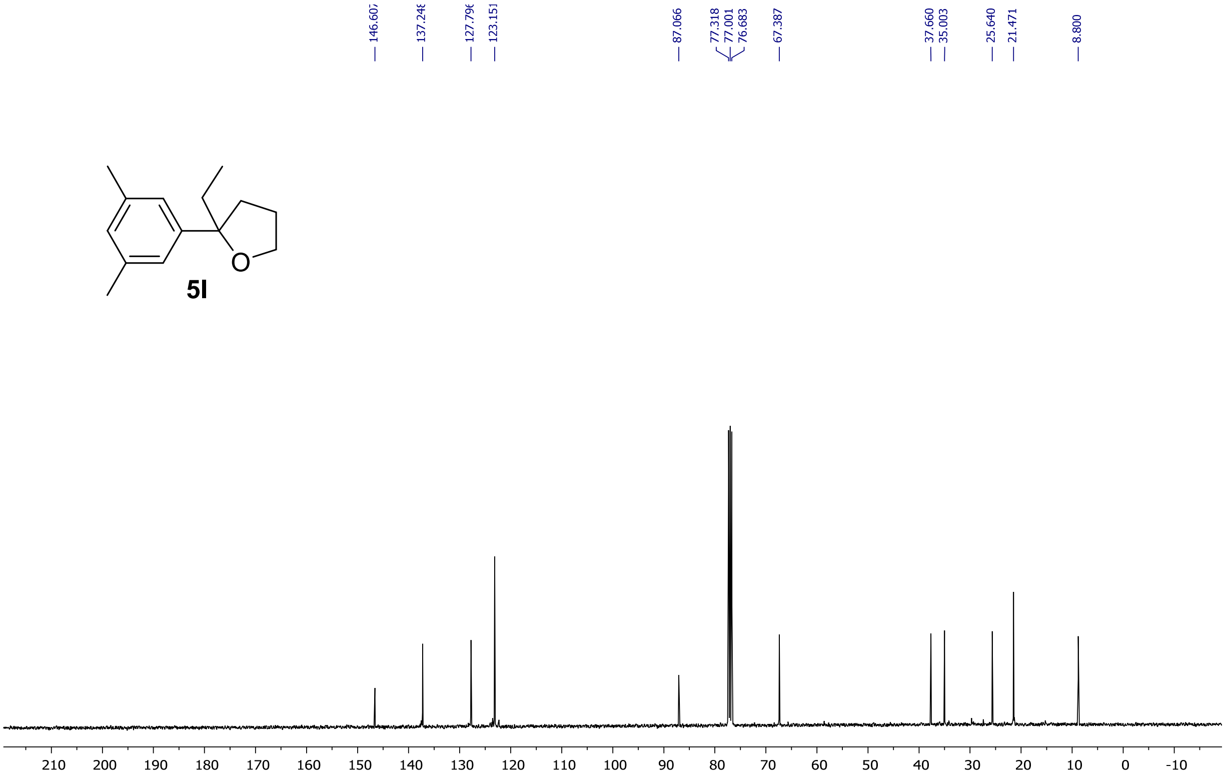

Supplementary Figure 71. ^{1}H and ^{13}C NMR spectra of 5l

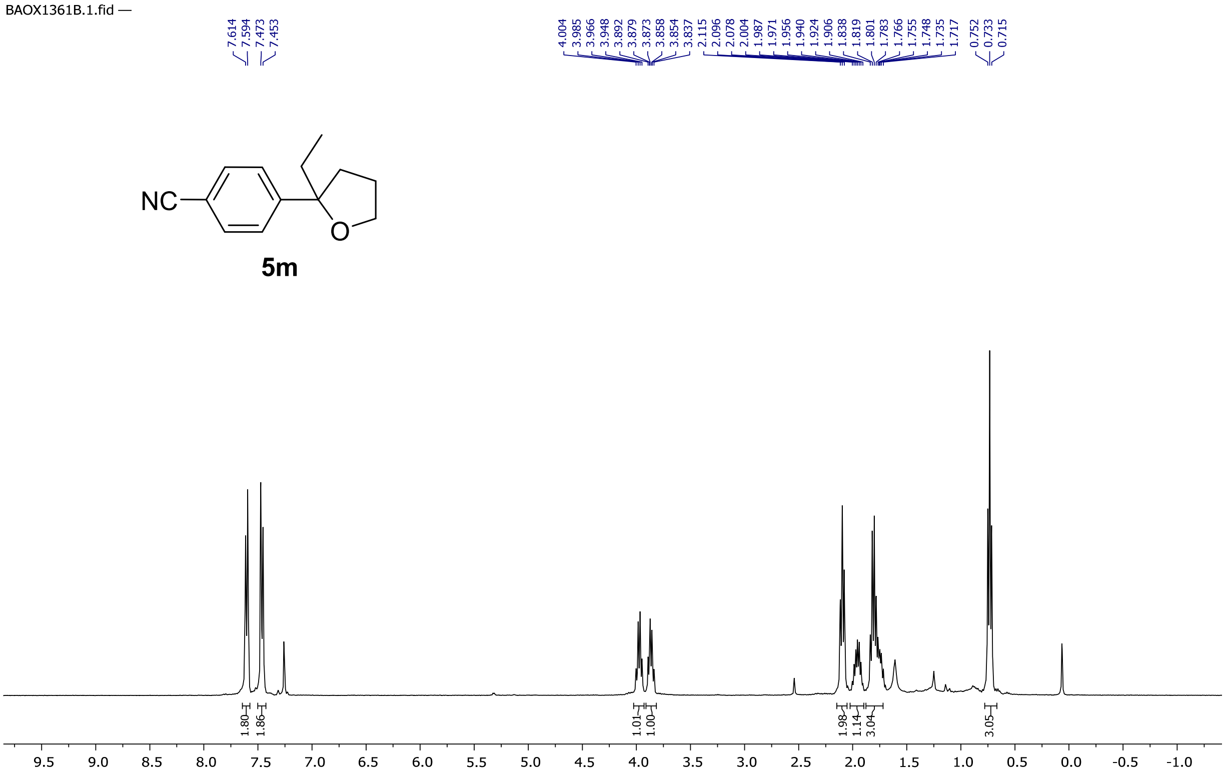

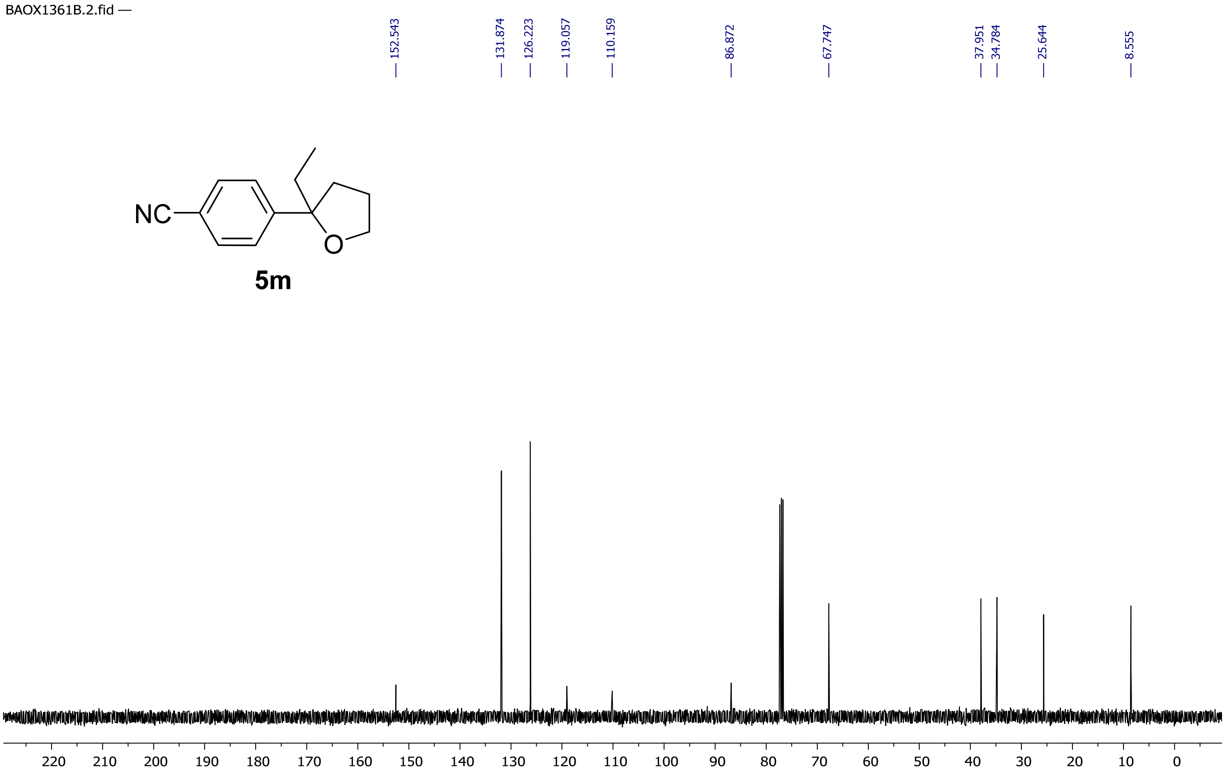

Supplementary Figure 72. ^{1}H and ^{13}C NMR spectra of 5m

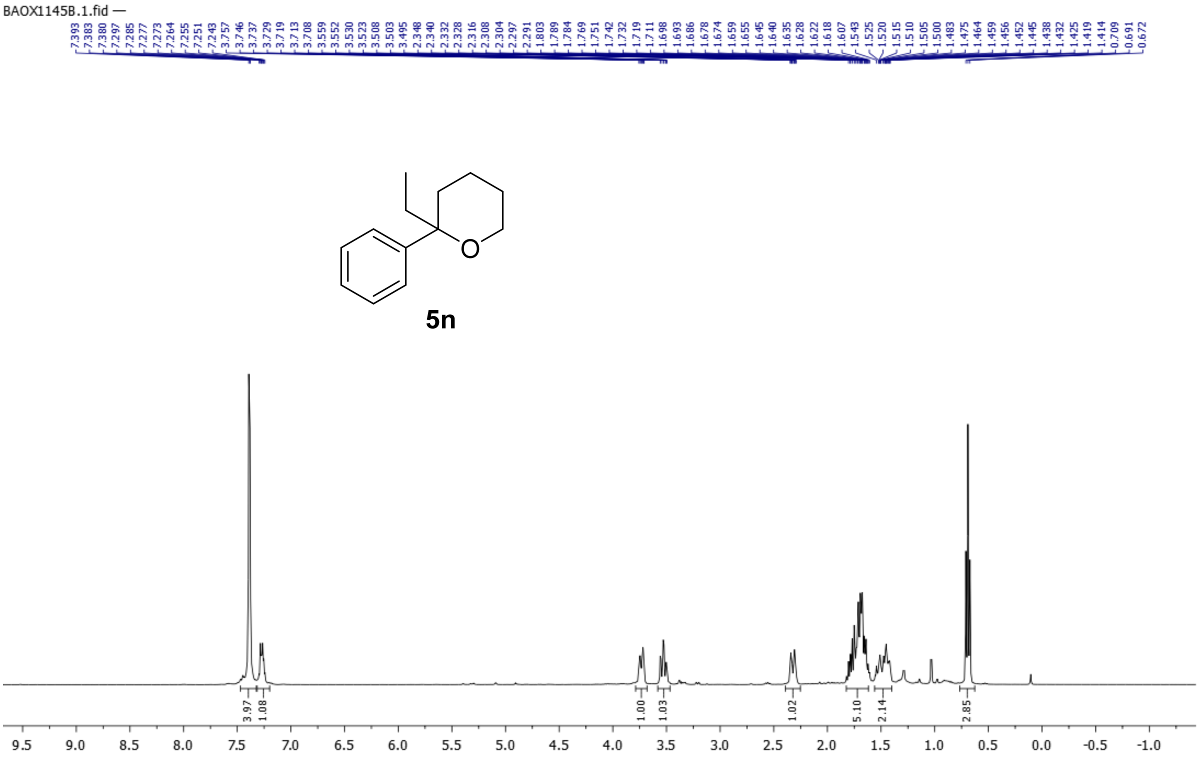

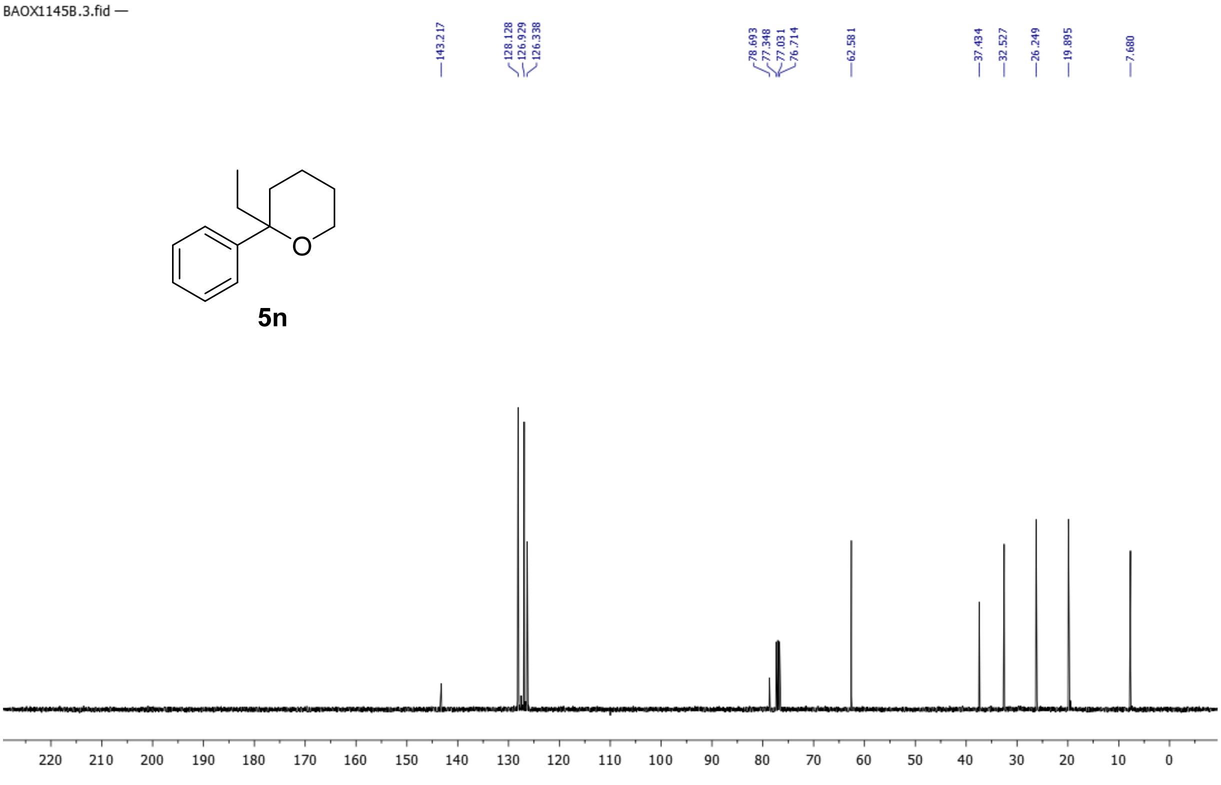

Supplementary Figure 73. ^{1}H and ^{13}C NMR spectra of 5n

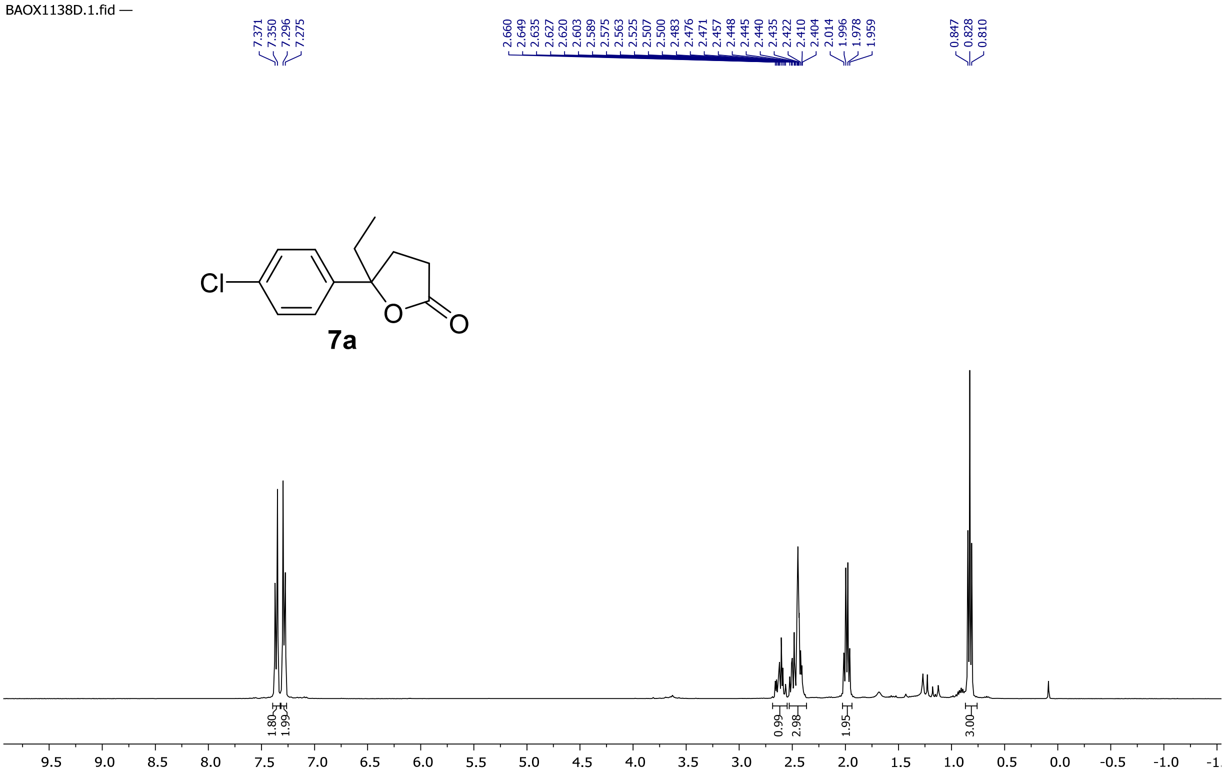

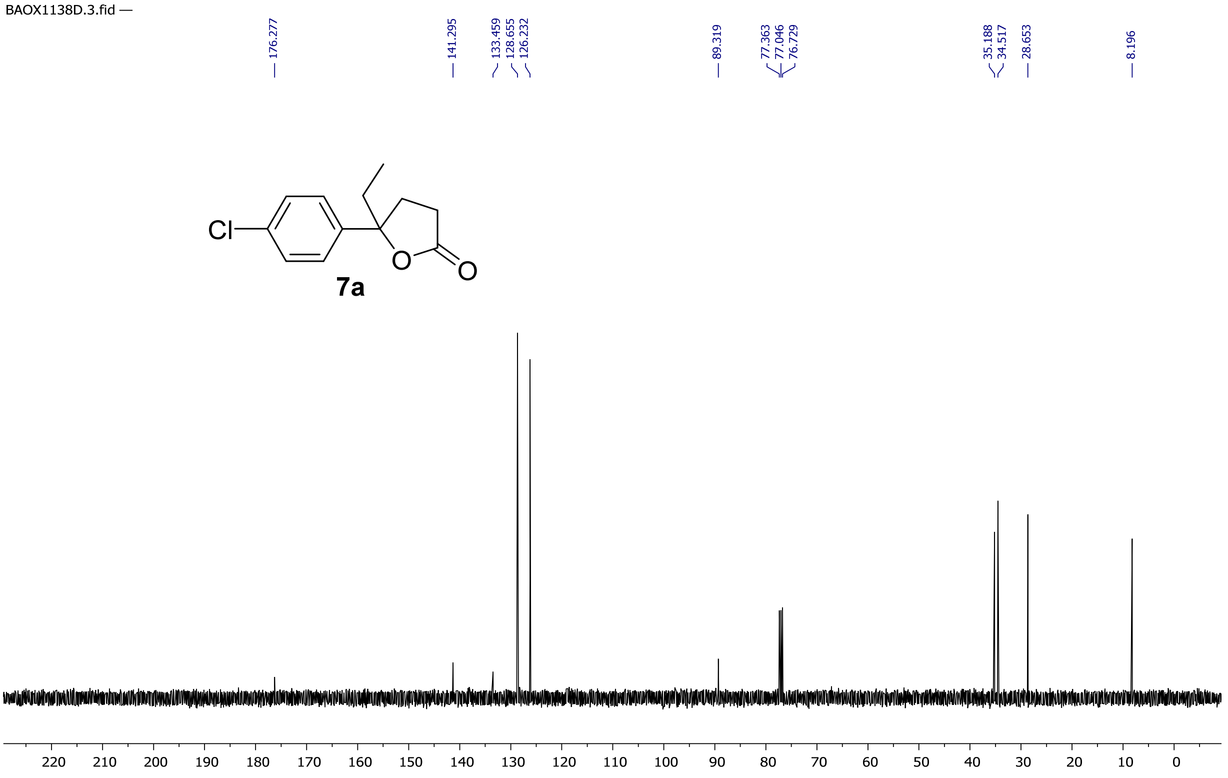

Supplementary Figure 74. ^{1}H and ^{13}C NMR spectra of 7a

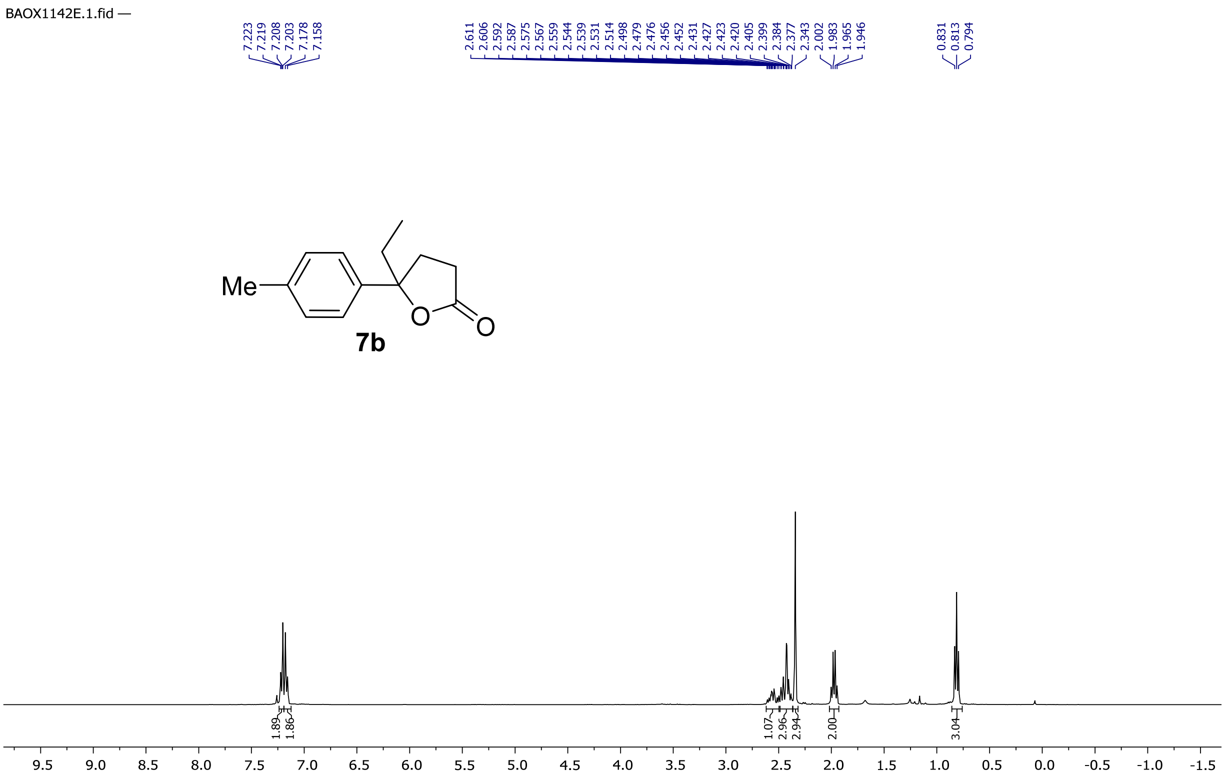

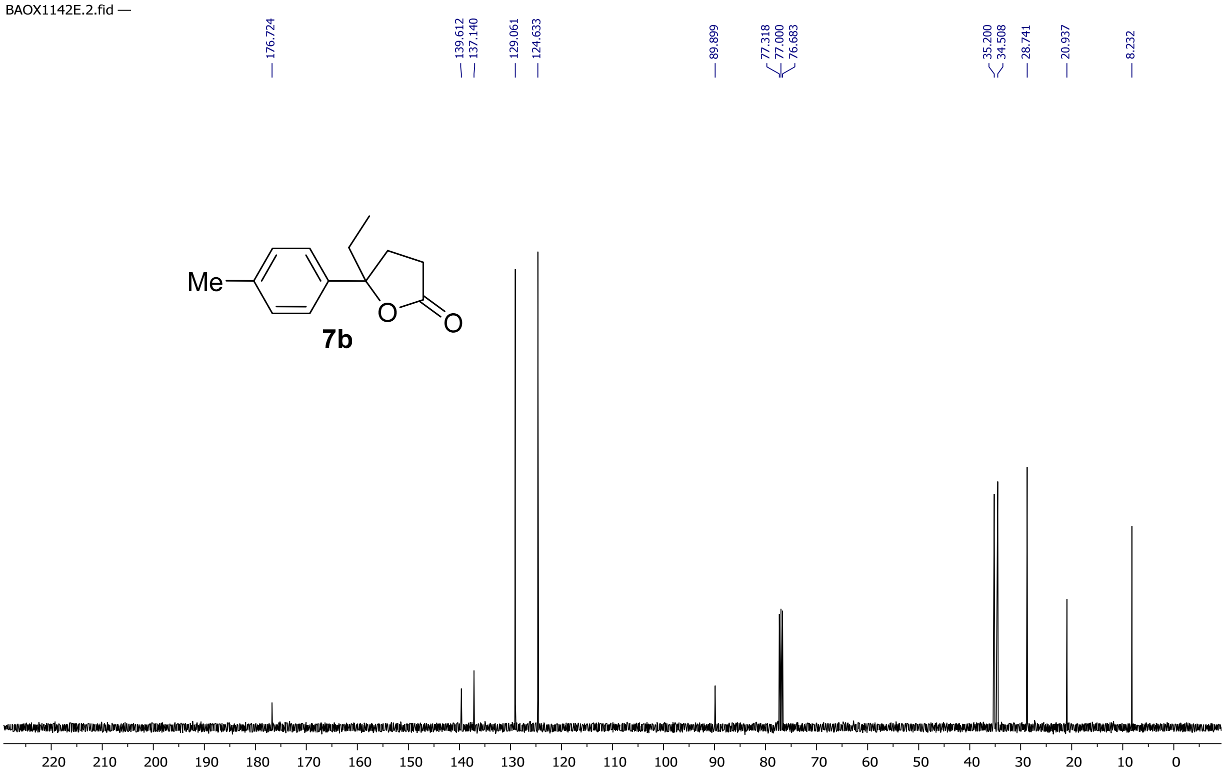

Supplementary Figure 75. ^{1}**H and** ^{13}**C NMR spectra of 7b**

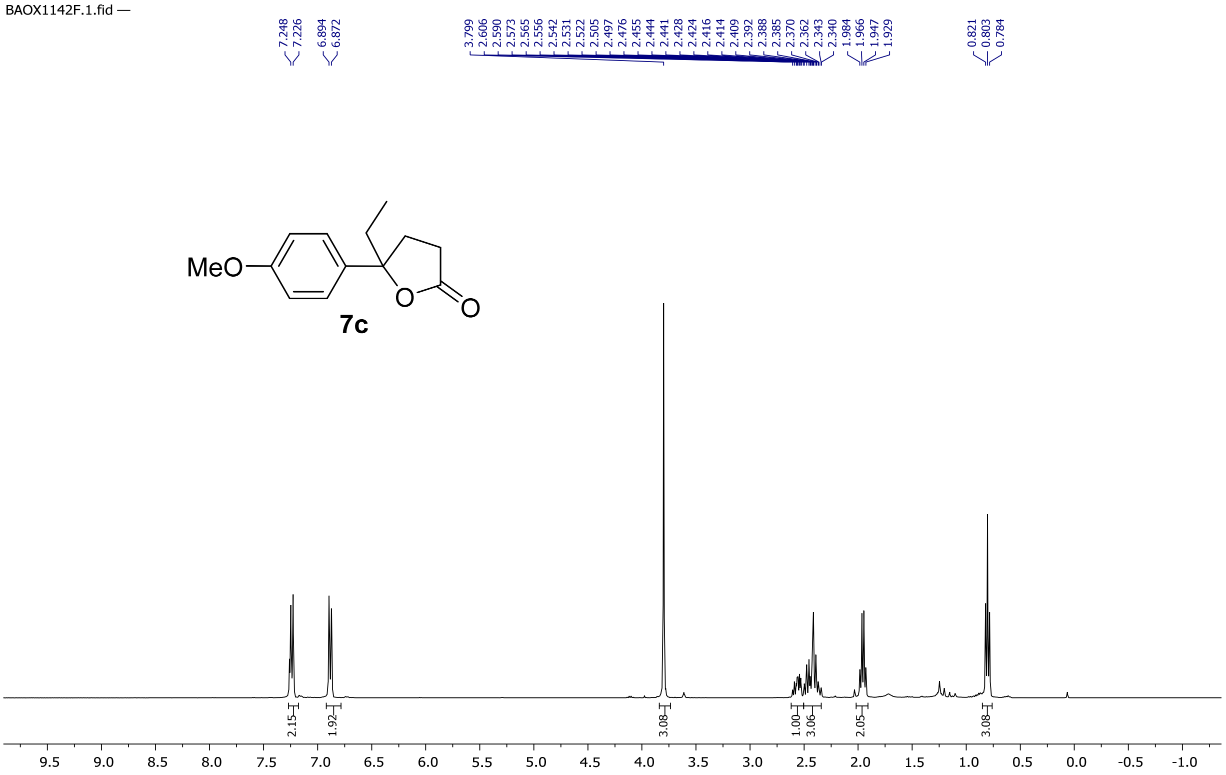

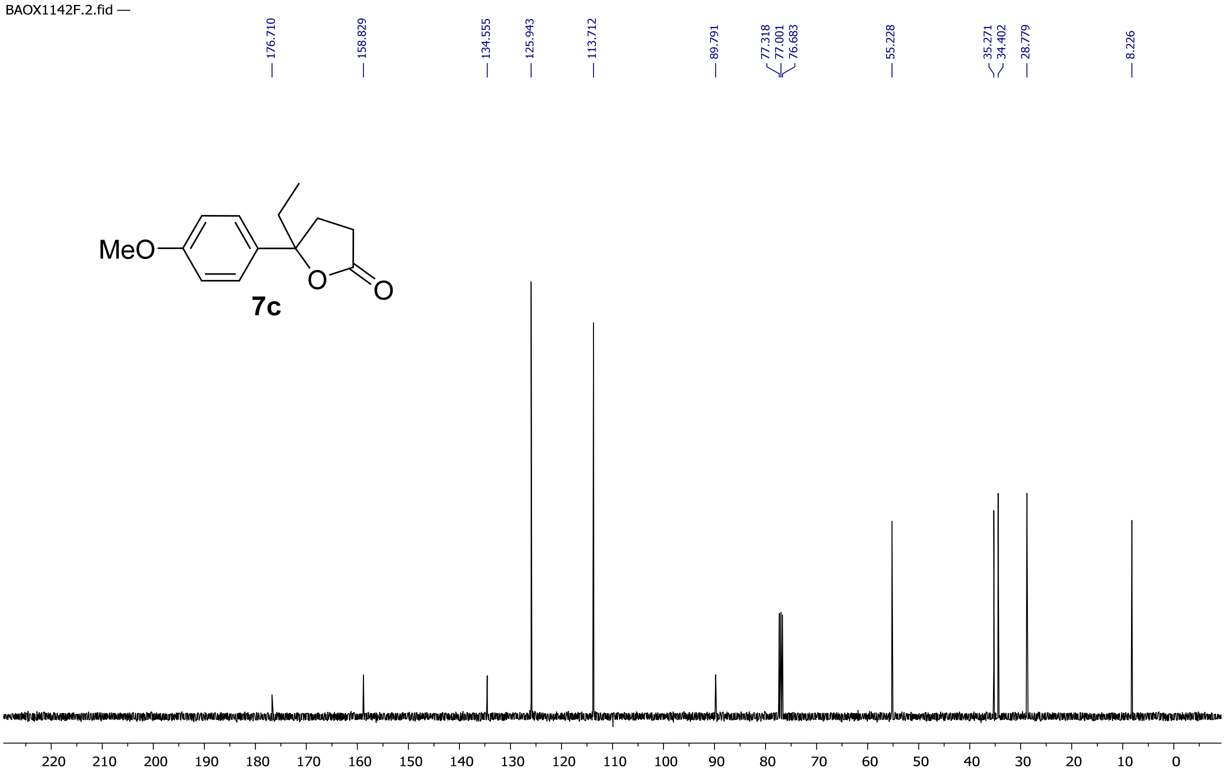

Supplementary Figure 76. ^{1}H and ^{13}C NMR spectra of 7c

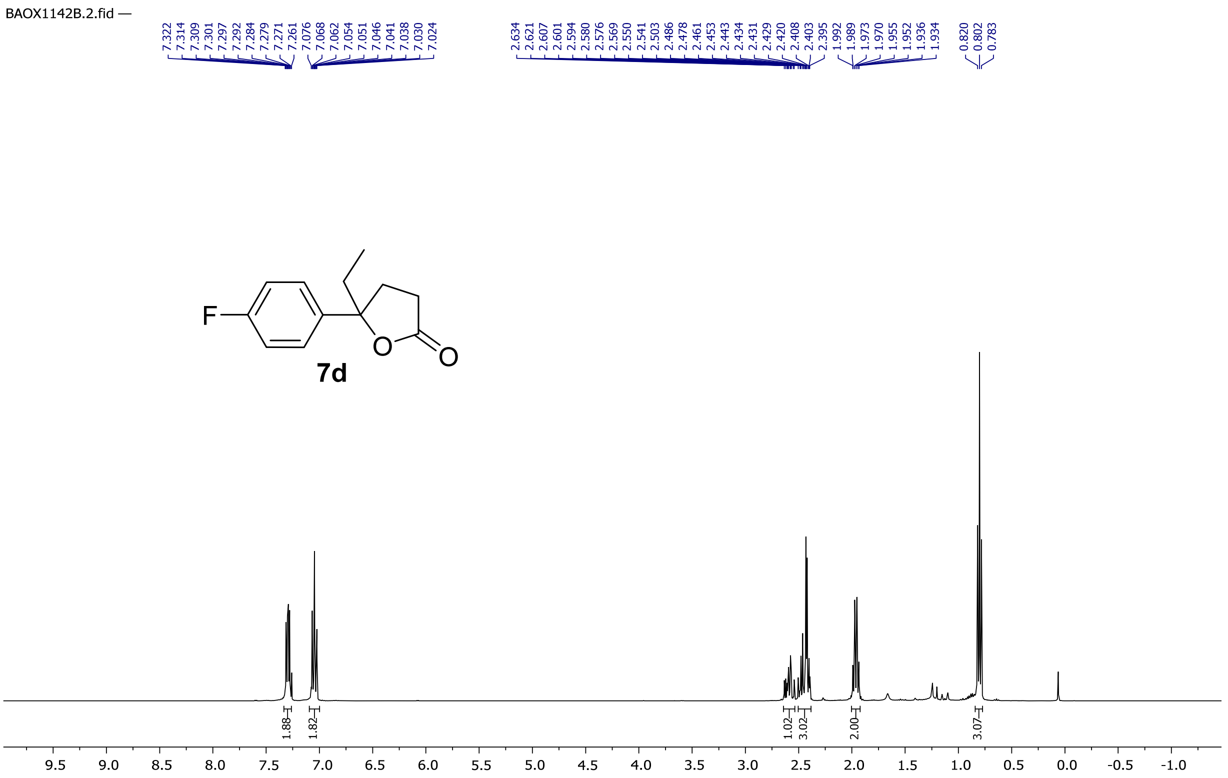

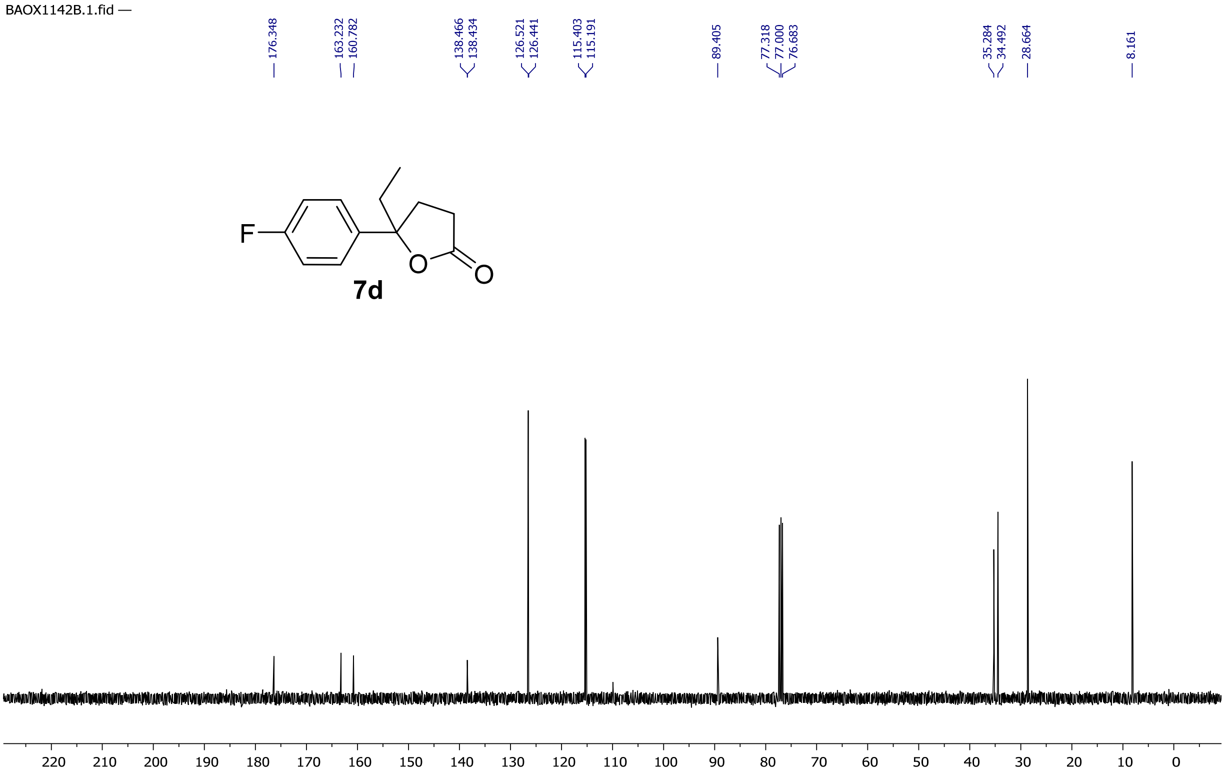

Supplementary Figure 77. ^{1}H and ^{13}C NMR spectra of 7d

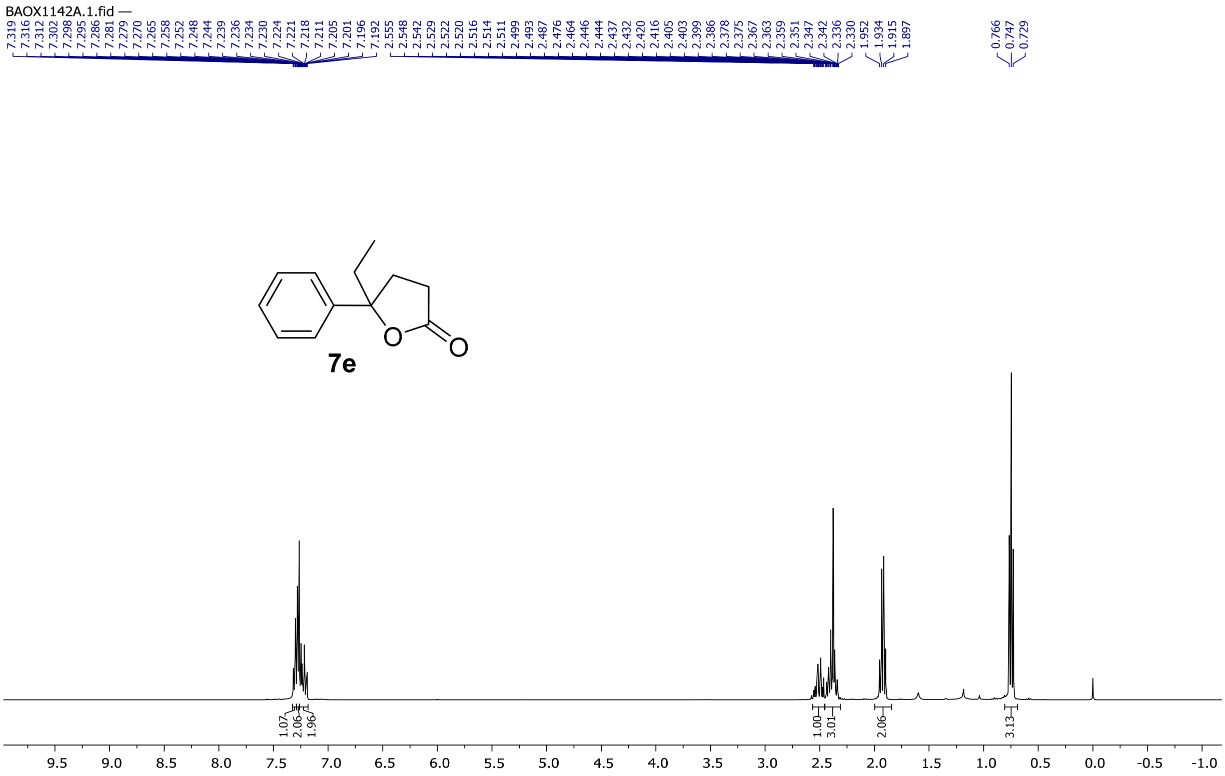

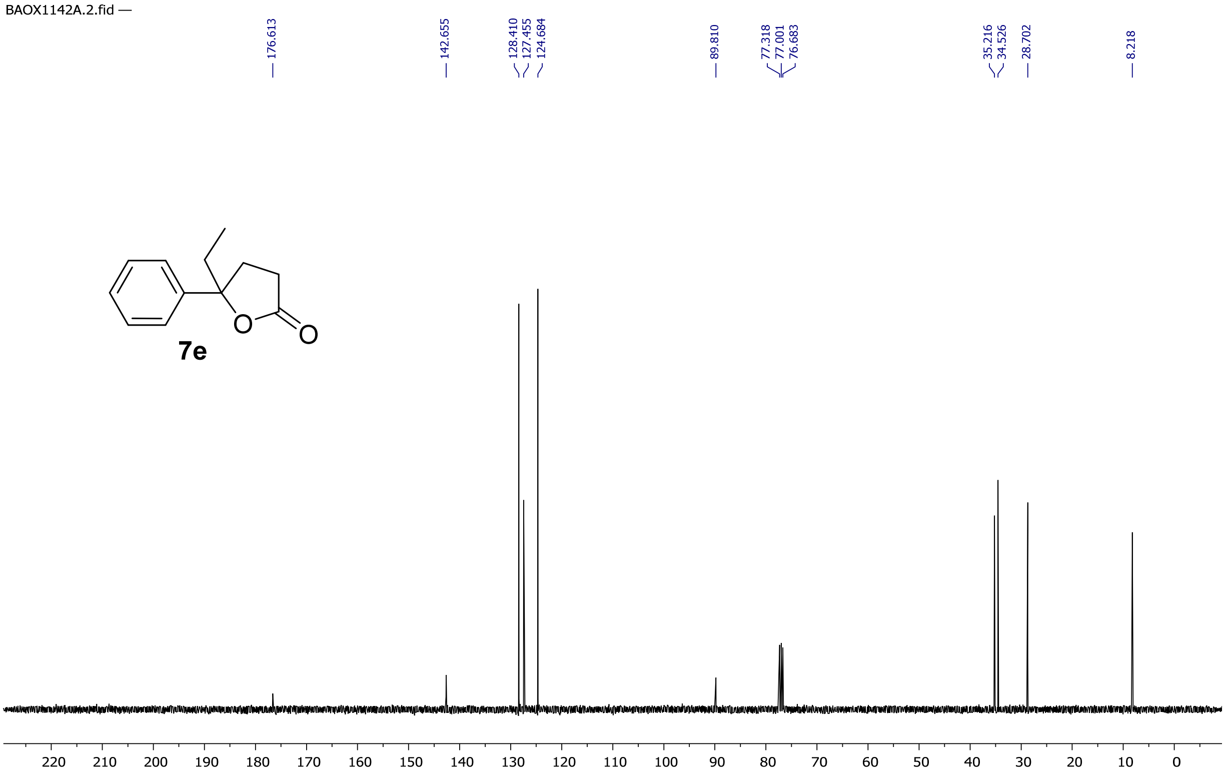

Supplementary Figure 78. ^{1}H and ^{13}C NMR spectra of 7e

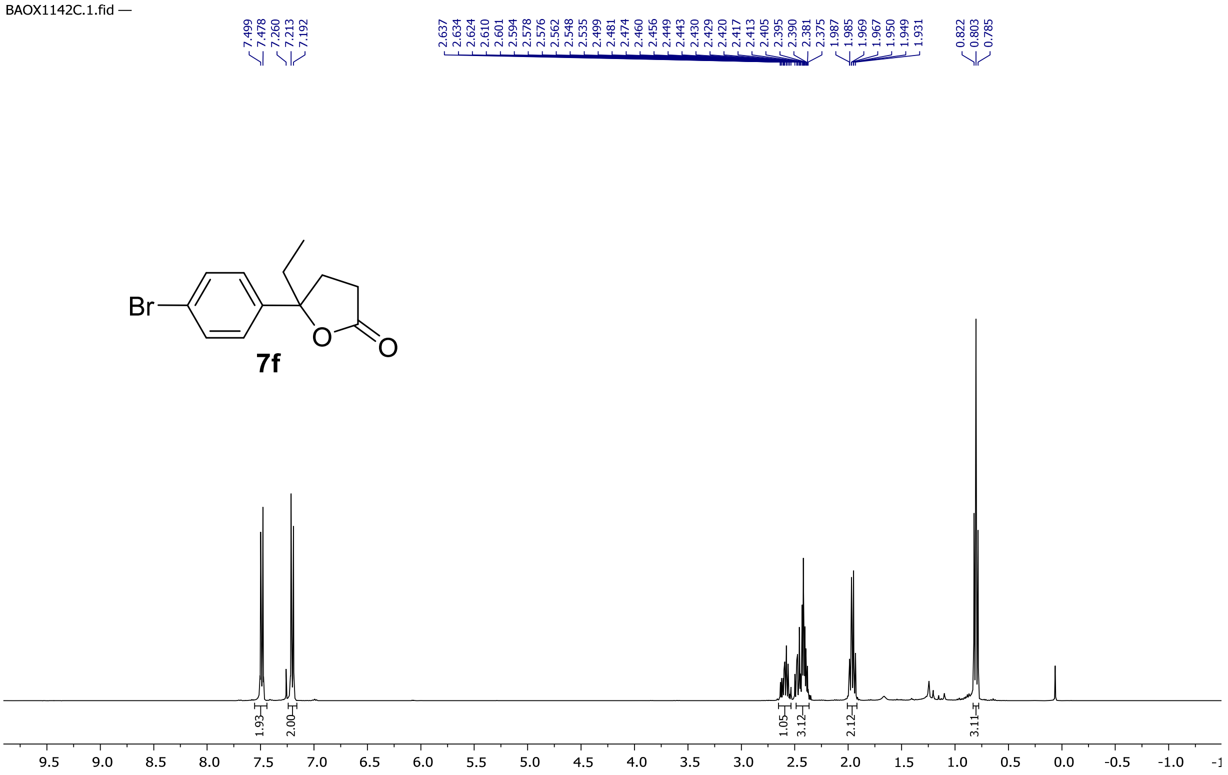

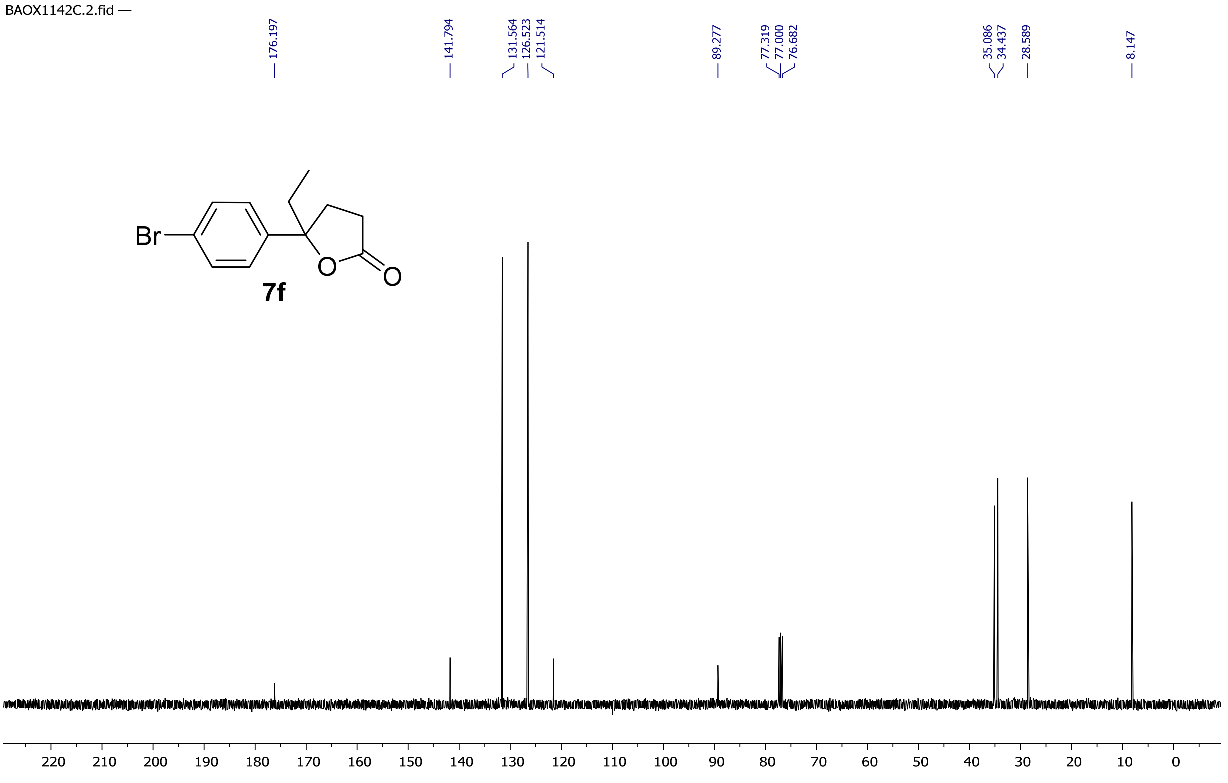

Supplementary Figure 79. ^{1}H and ^{13}C NMR spectra of 7f

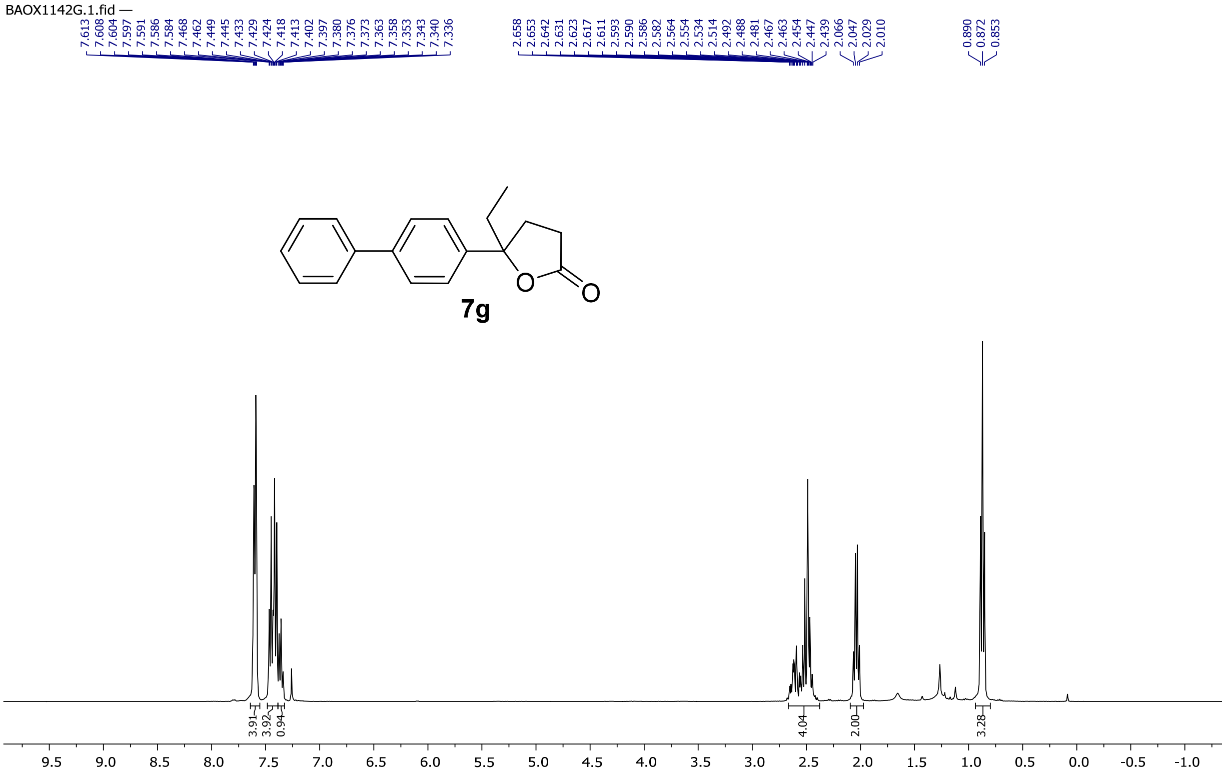

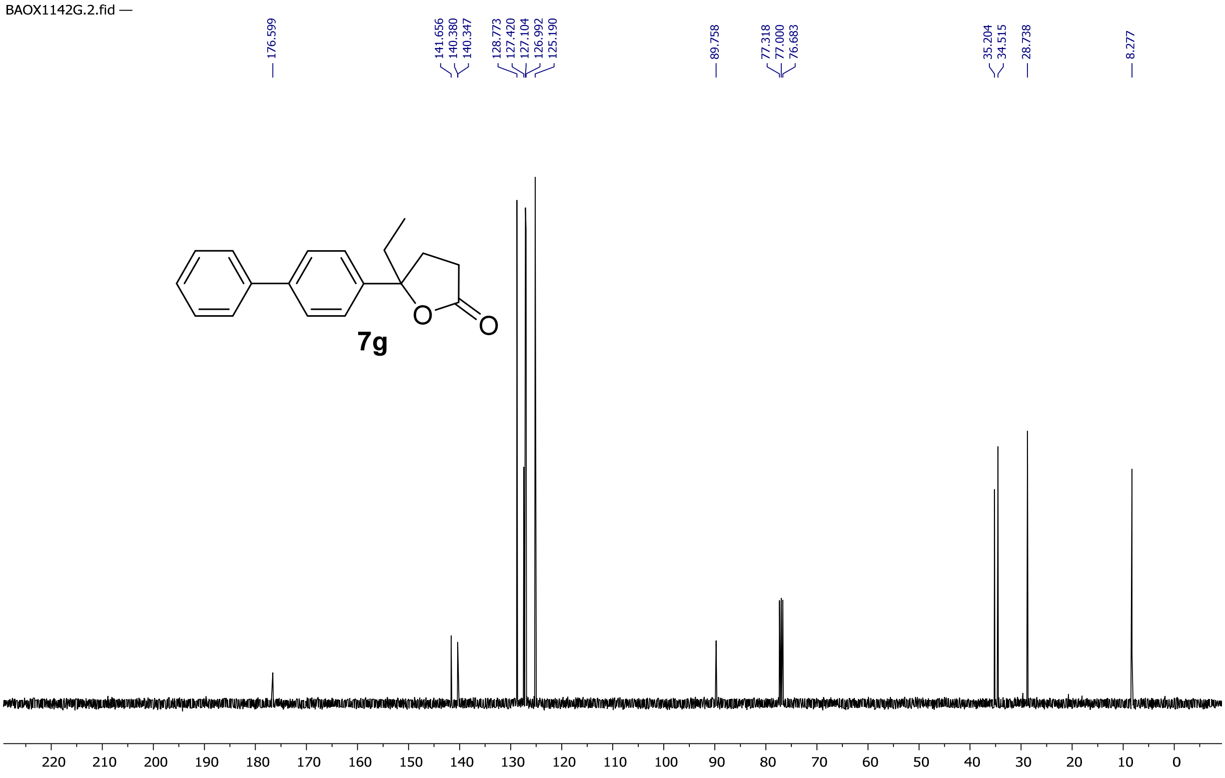

Supplementary Figure 80. ^{1}H and ^{13}C NMR spectra of 7g

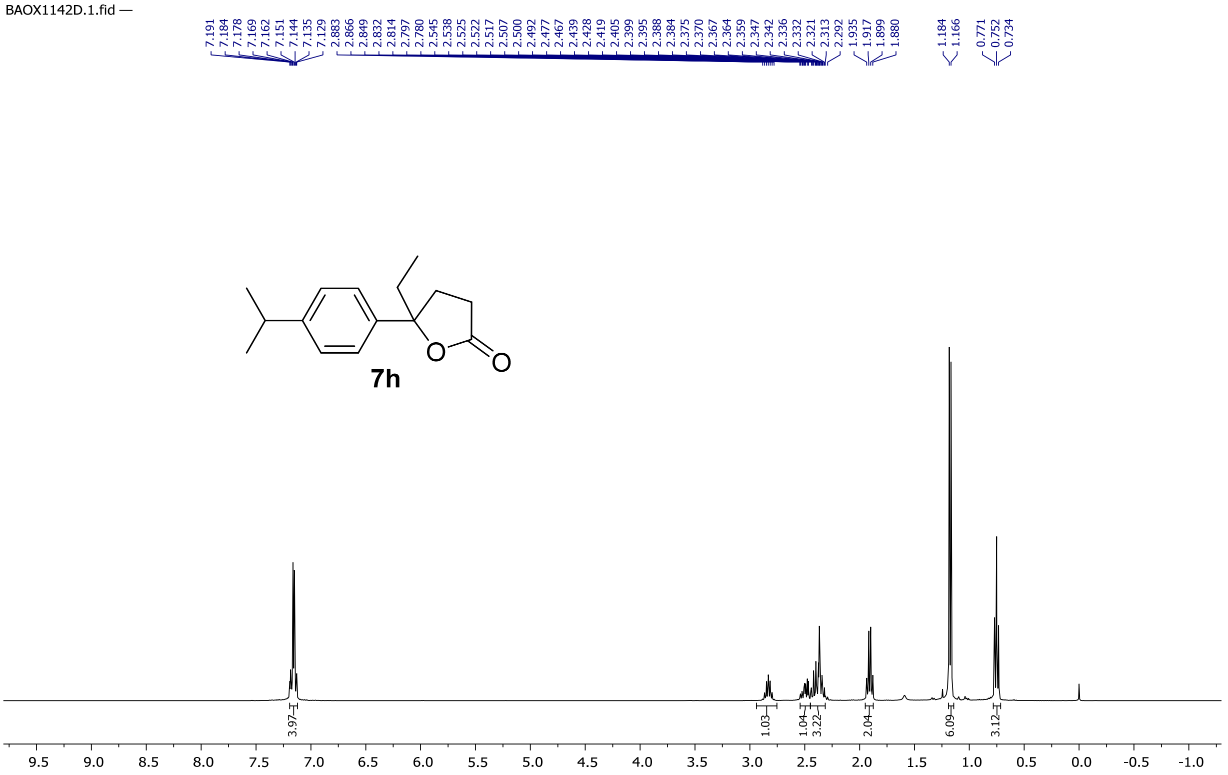

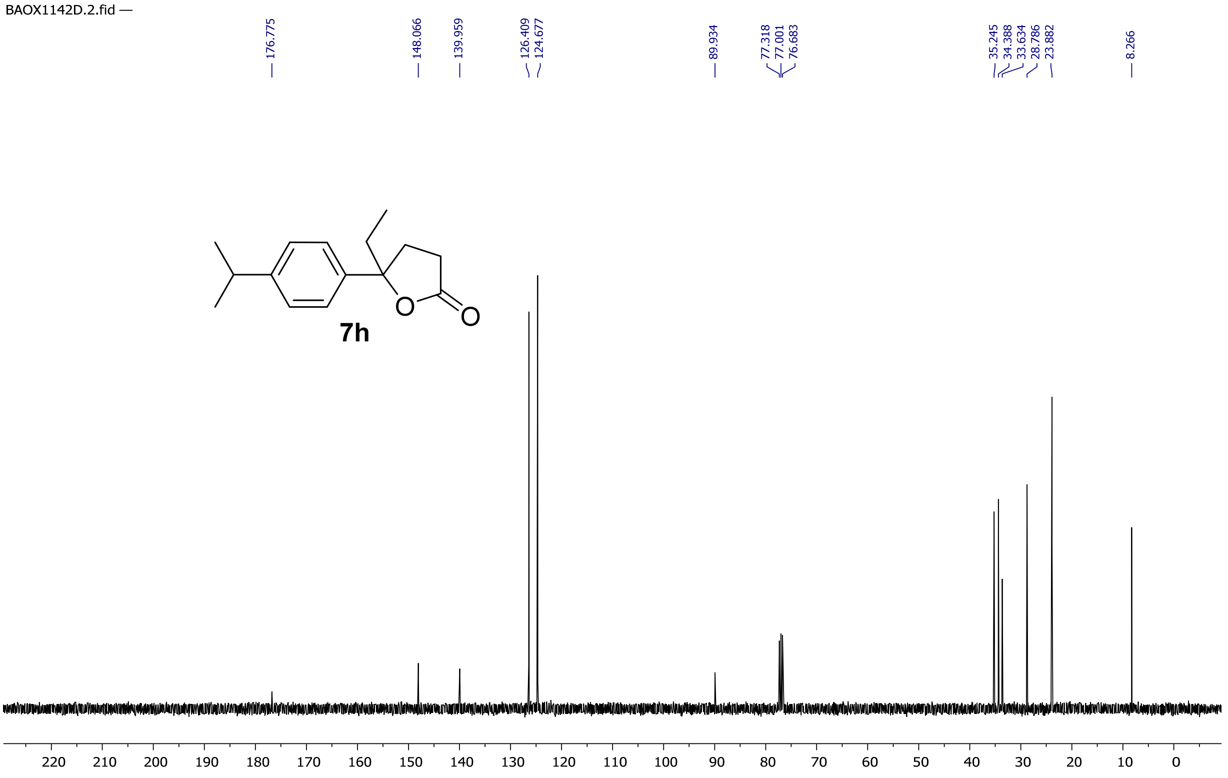

Supplementary Figure 81. ^{1}H **and** ^{13}C **NMR spectra of 7h**

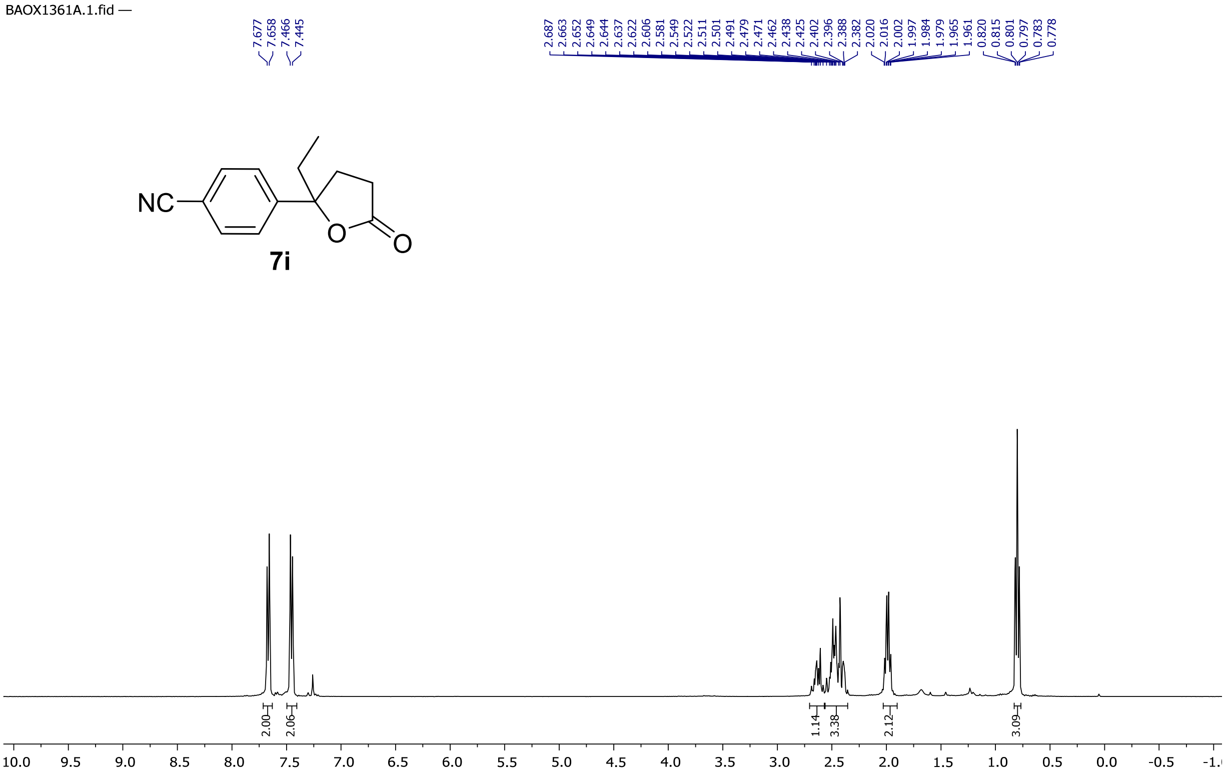

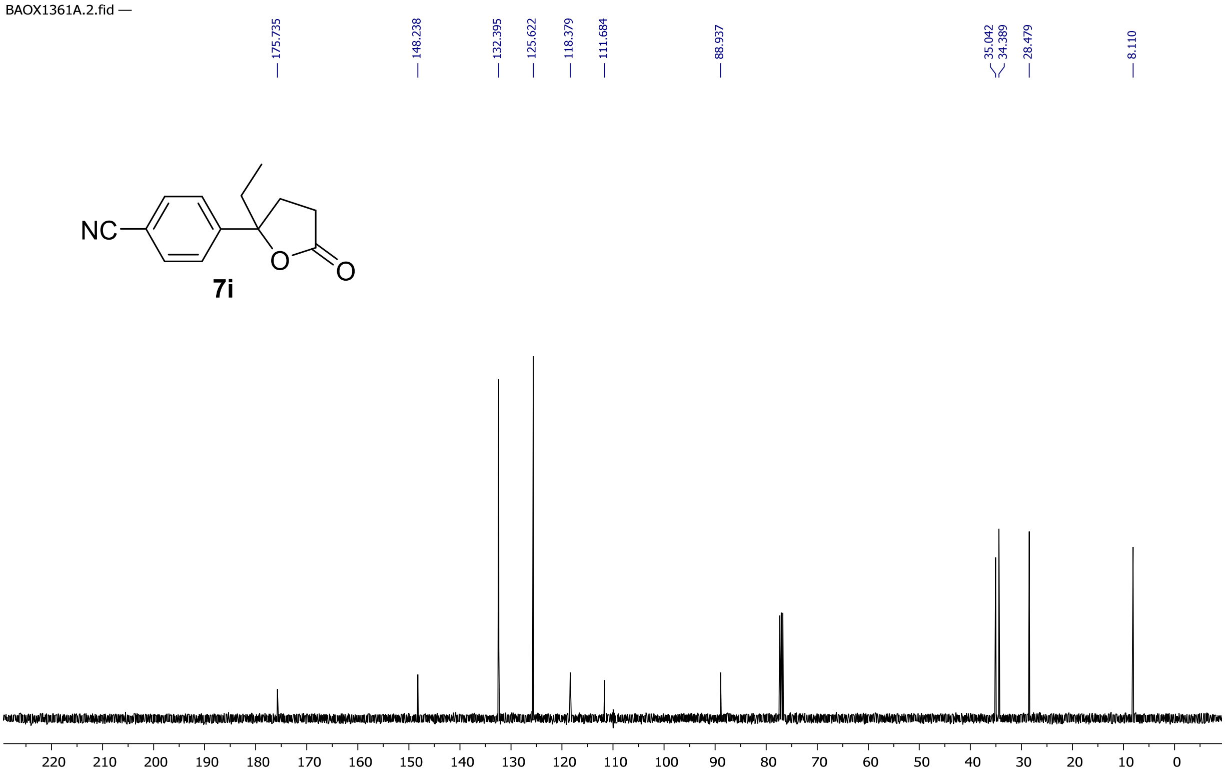

Supplementary Figure 82. ^{1}H and ^{13}C NMR spectra of 7i

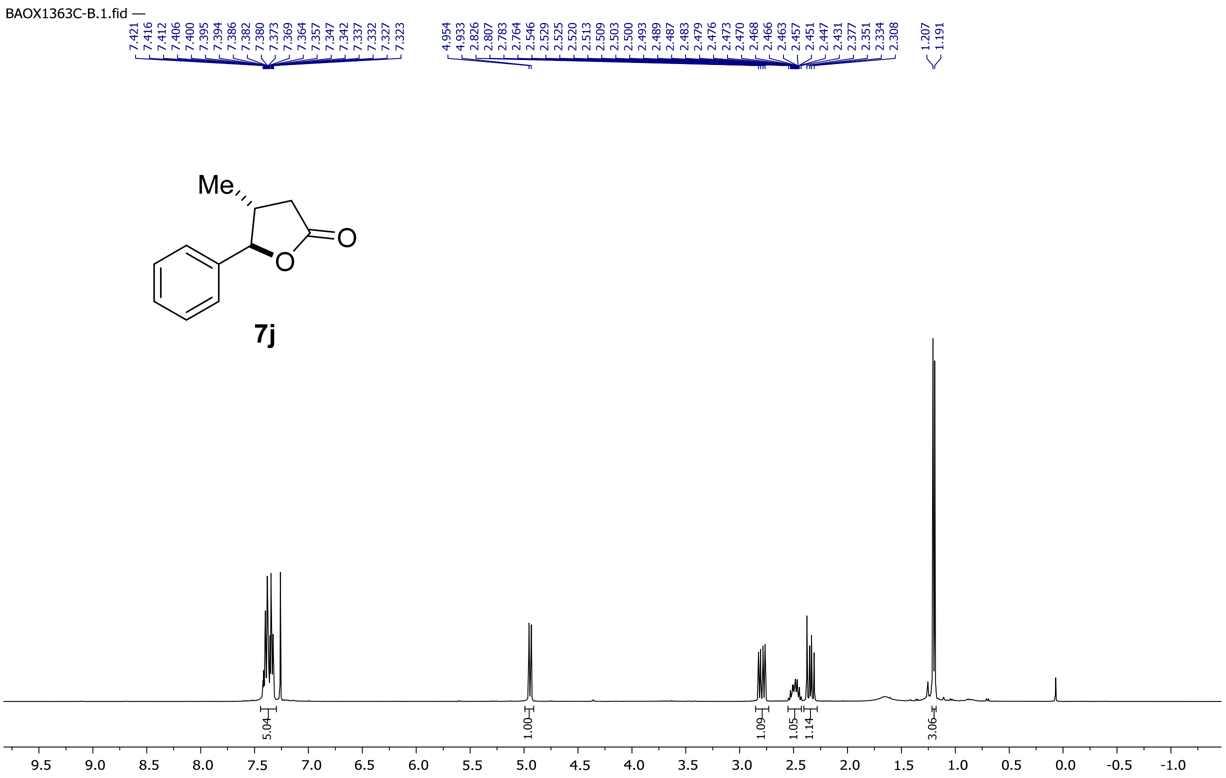

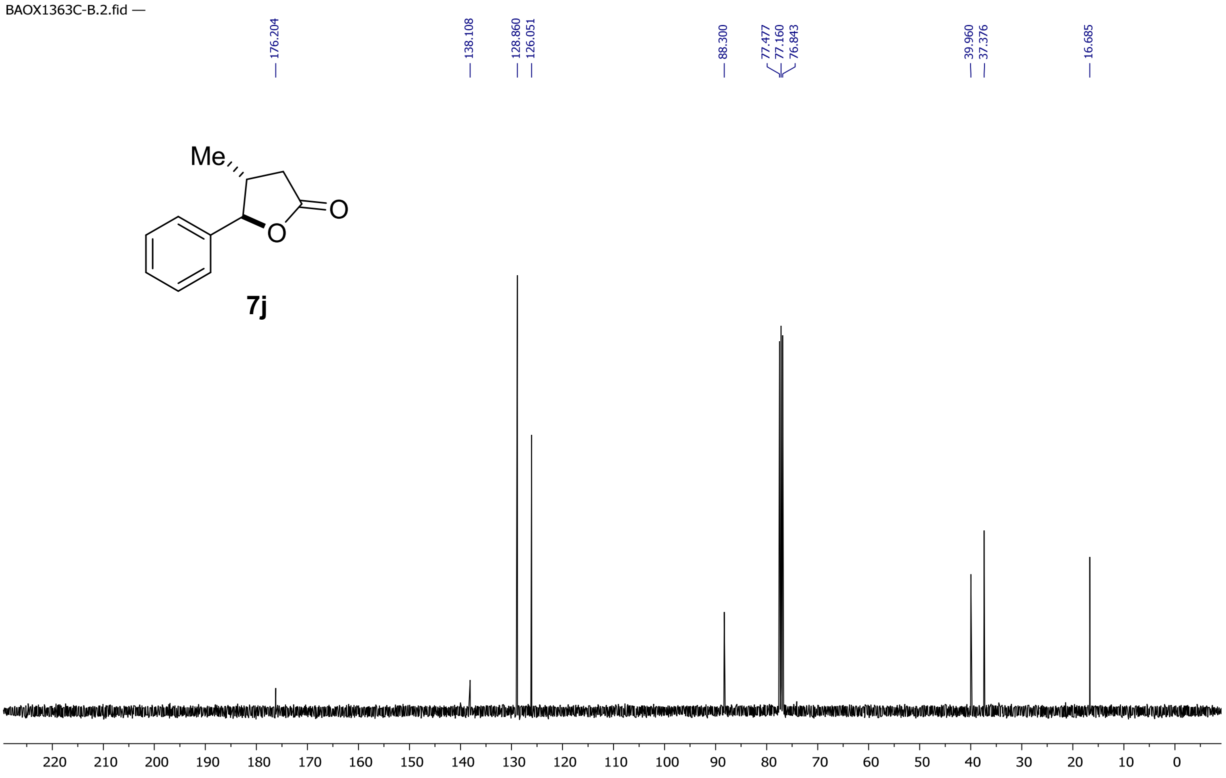

Supplementary Figure 83. ^{1}H and ^{13}C NMR spectra of 7j

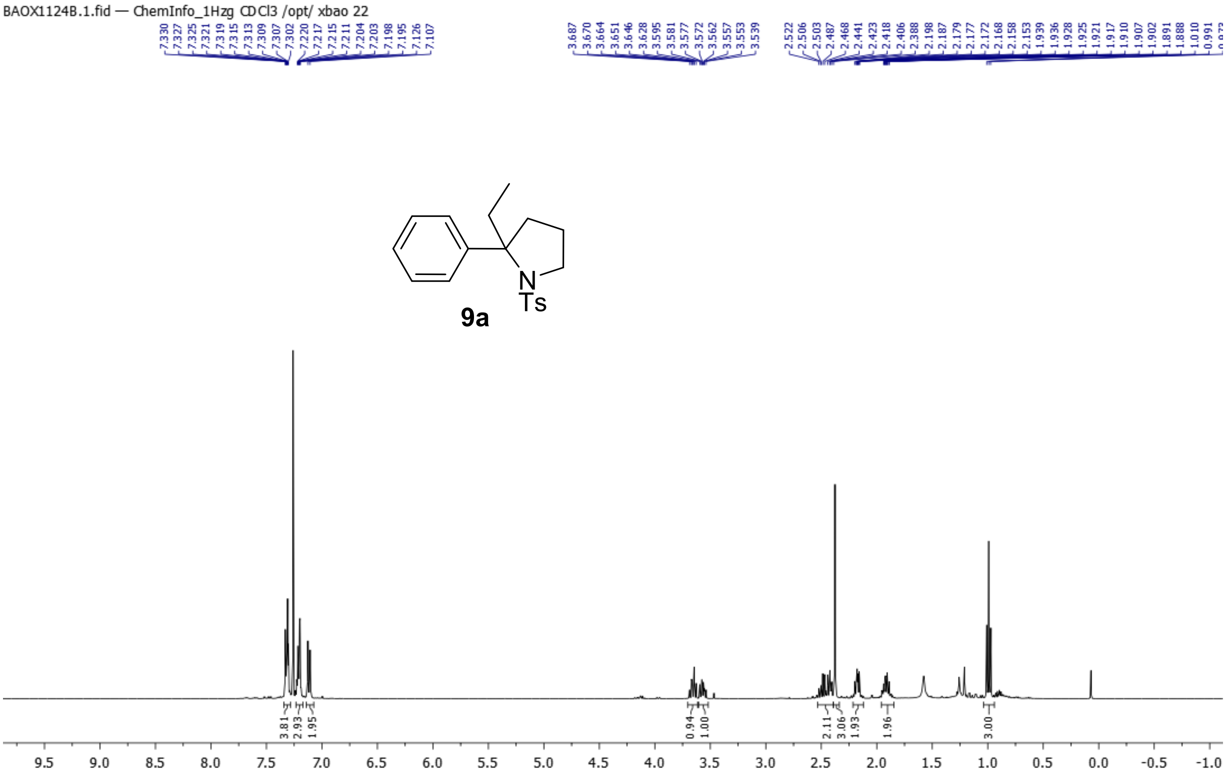

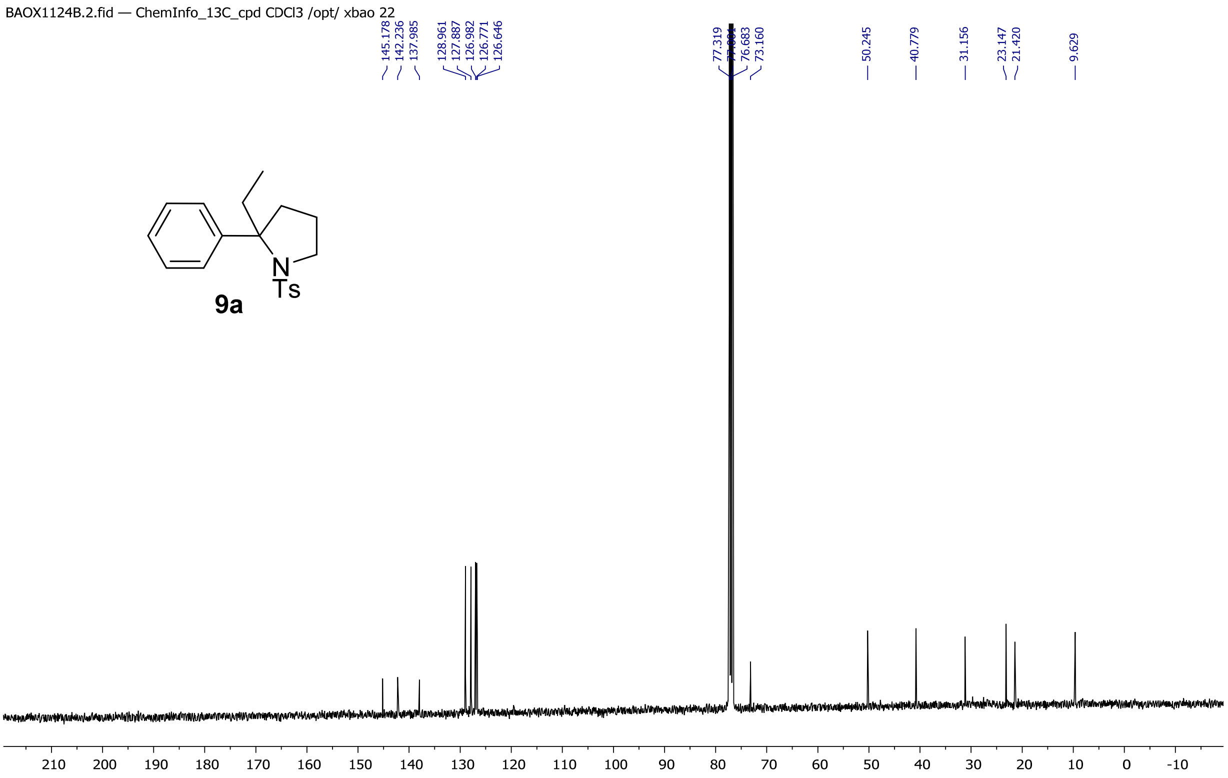

Supplementary Figure 84. ^{1}H and ^{13}C NMR spectra of 9a

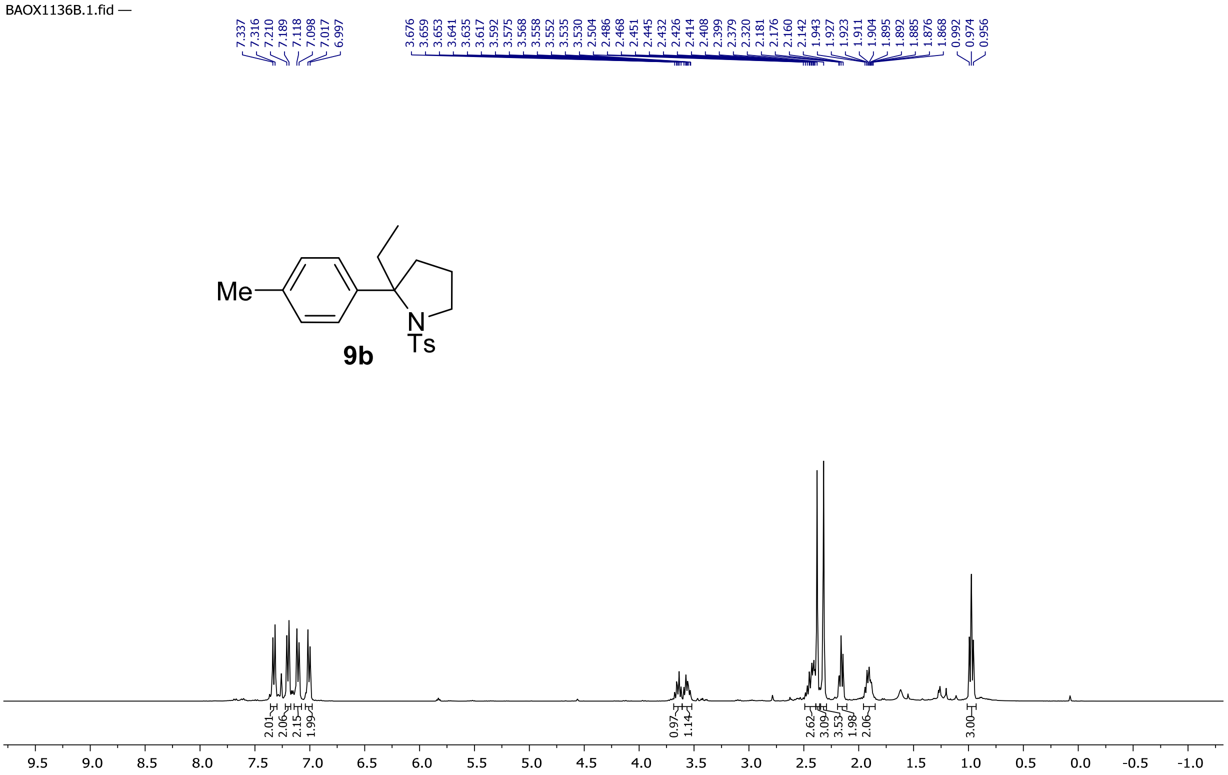

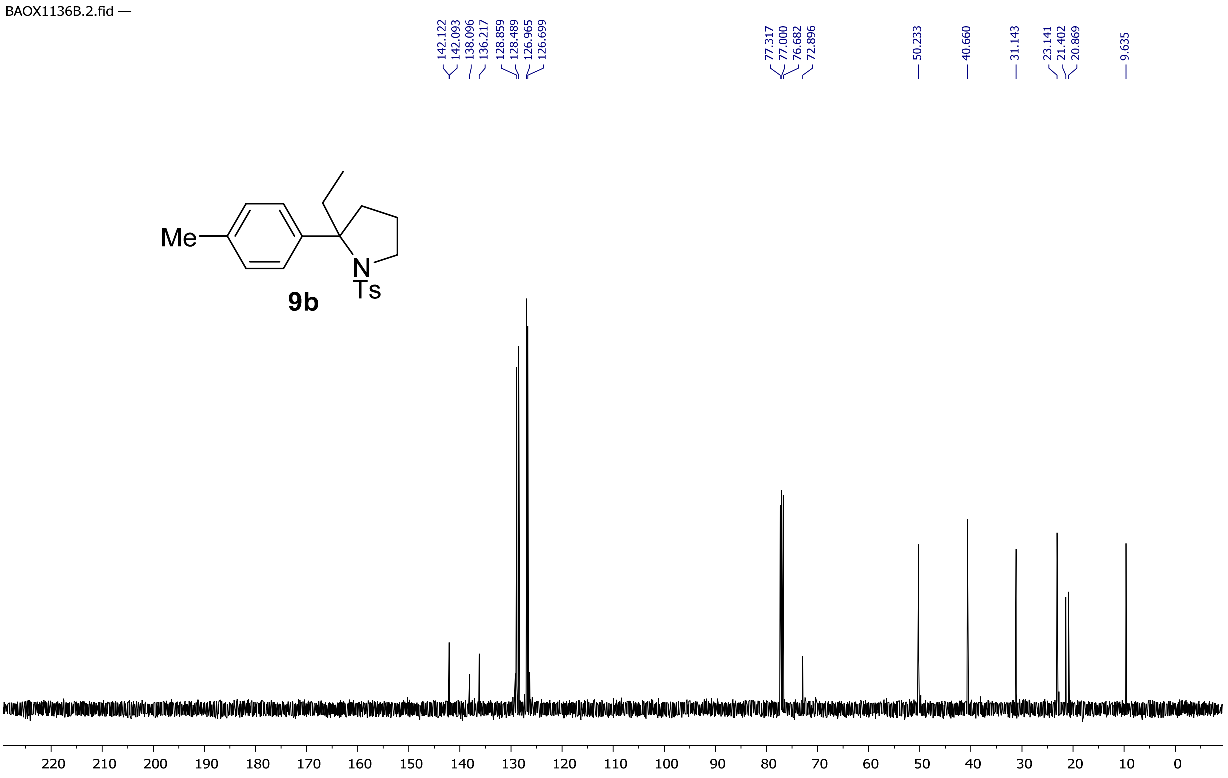

Supplementary Figure 85. ^{1}H and ^{13}C NMR spectra of 9b

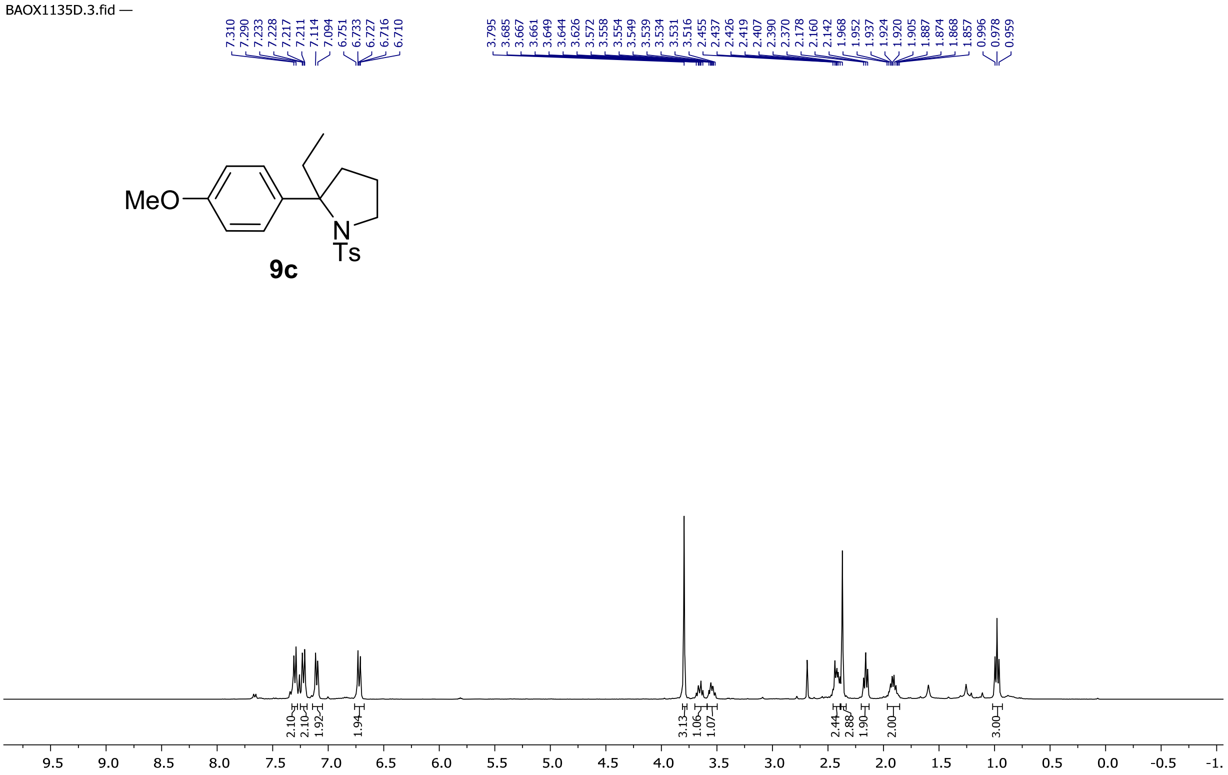

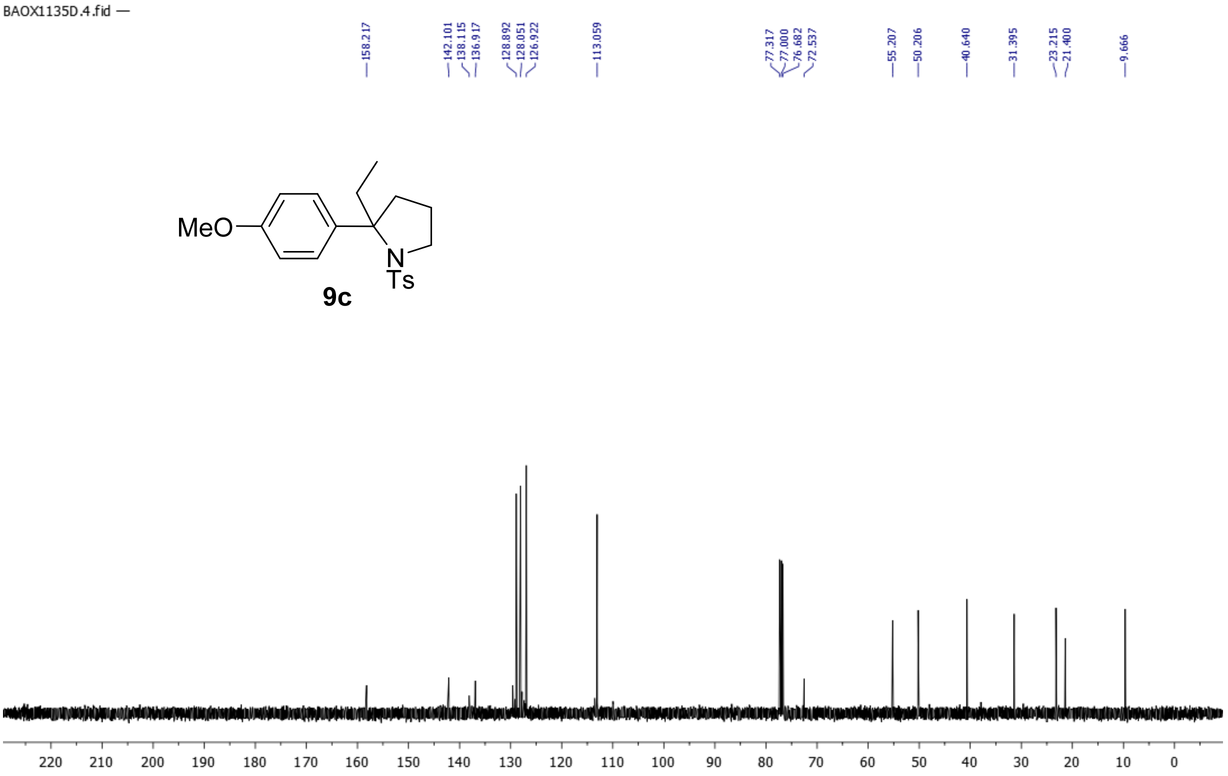

Supplementary Figure 86. ^{1}H and ^{13}C NMR spectra of 9c

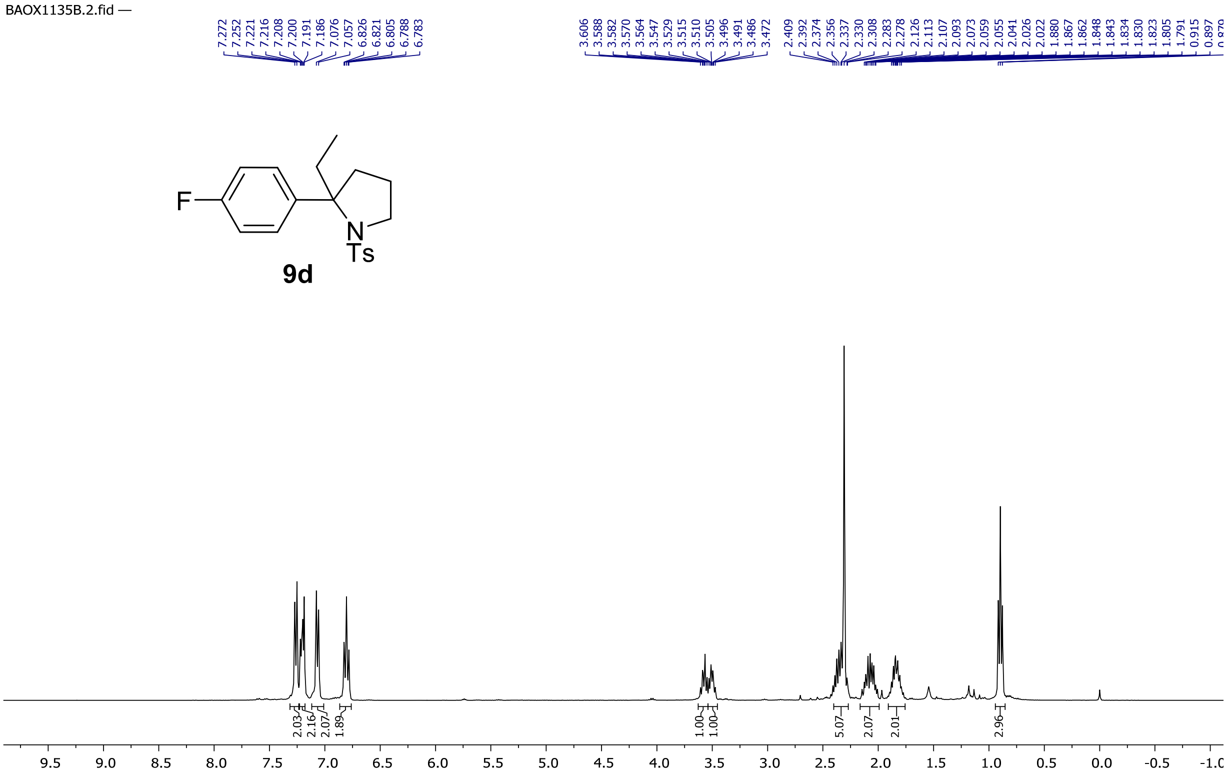

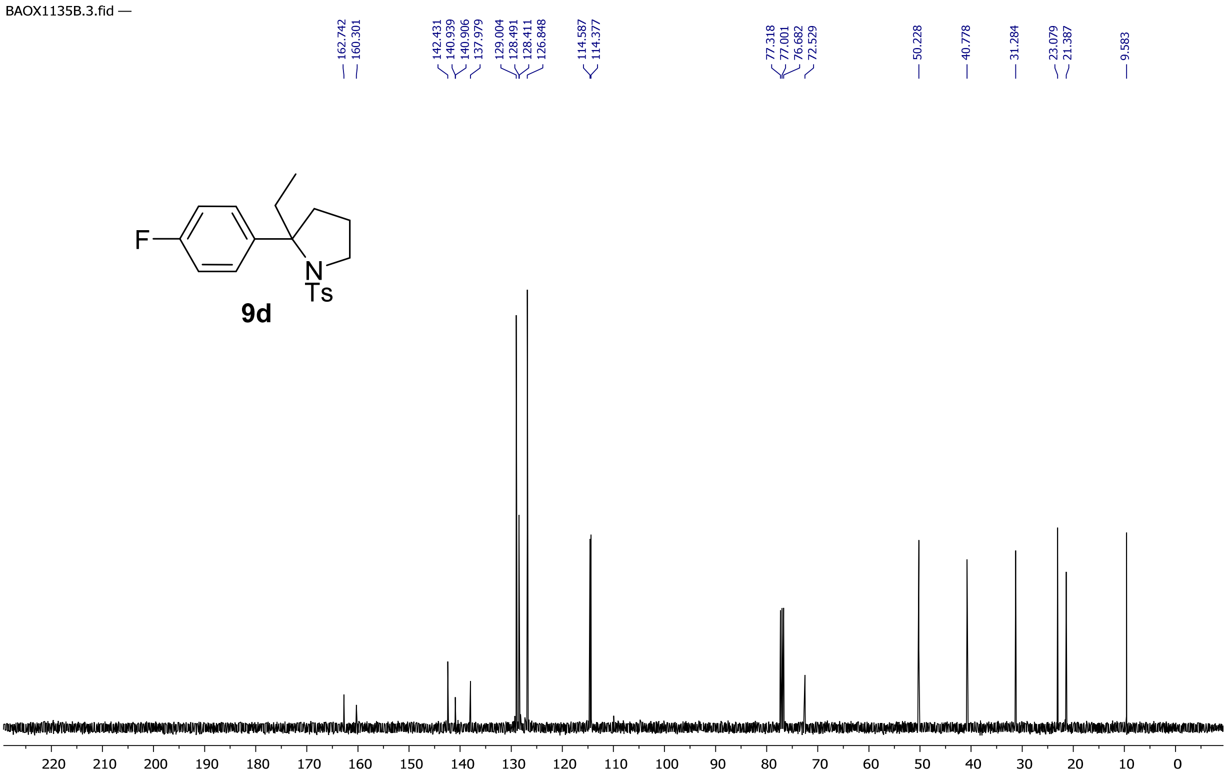

Supplementary Figure 87. ^{1}H and ^{13}C NMR spectra of 9d

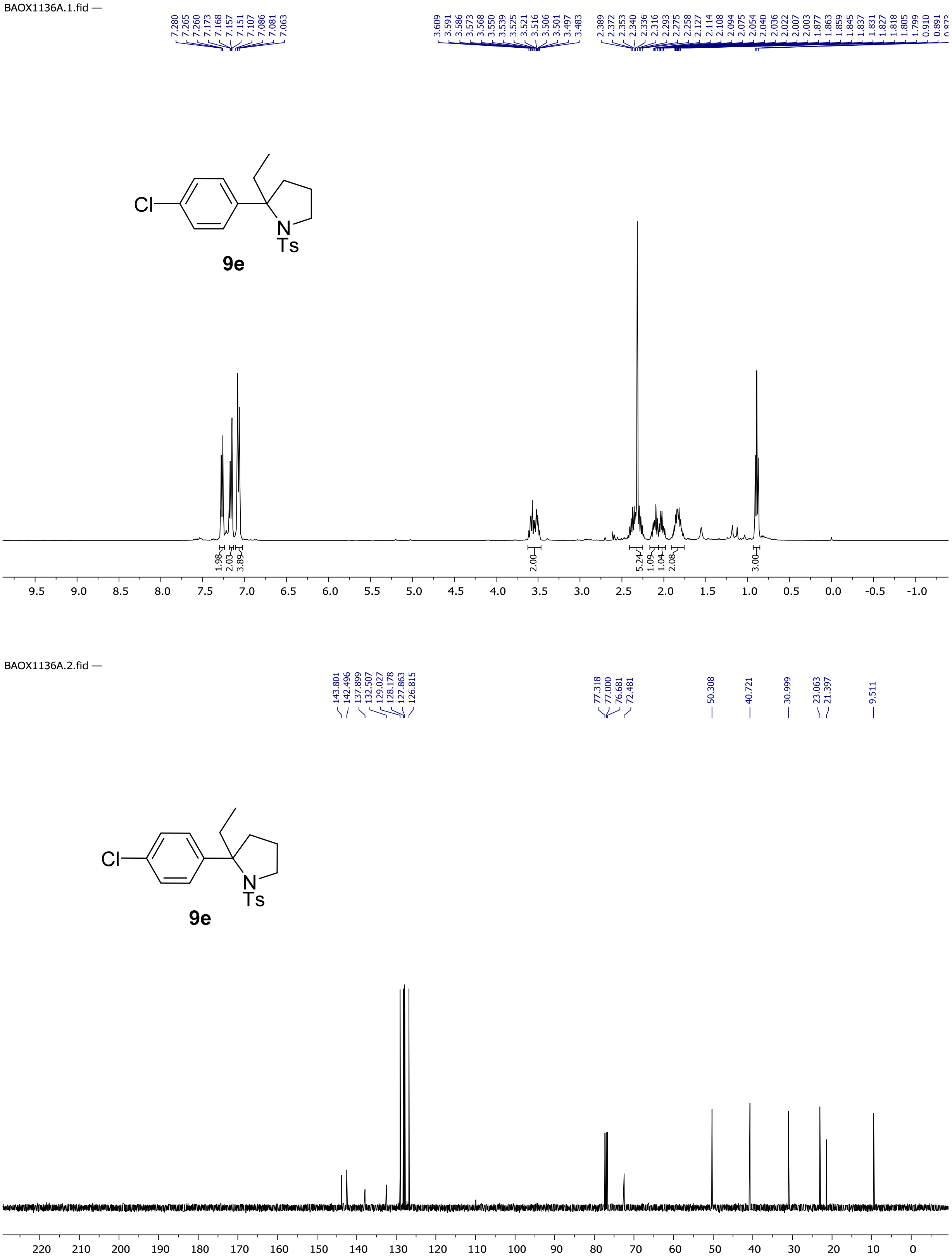

Supplementary Figure 88. ^{1}H and ^{13}C NMR spectra of 9e

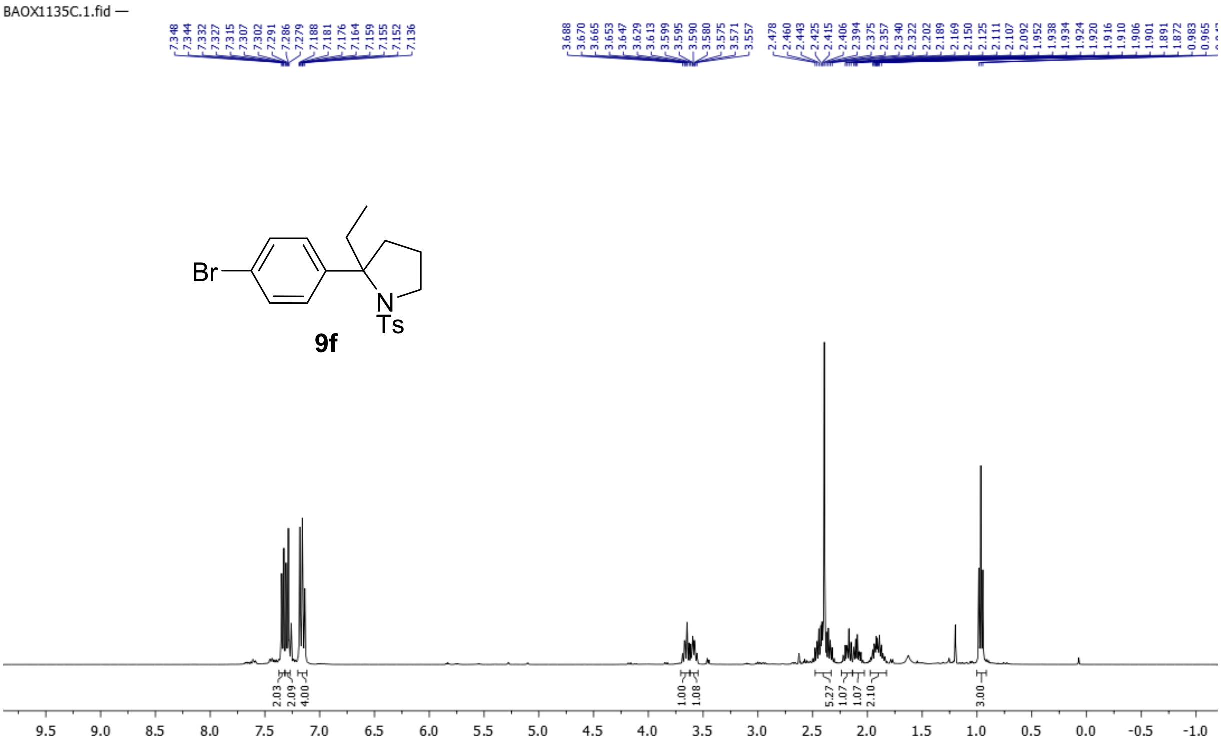

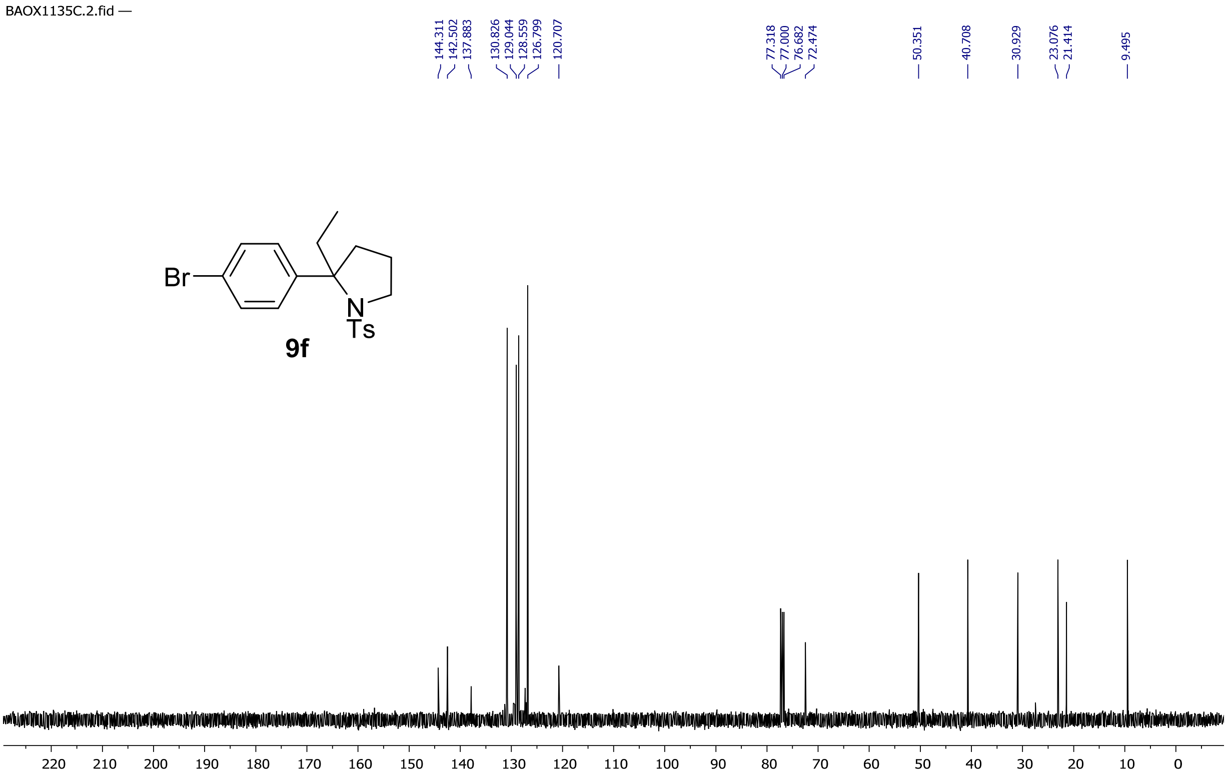

Supplementary Figure 89. ^{1}H and ^{13}C NMR spectra of 9f

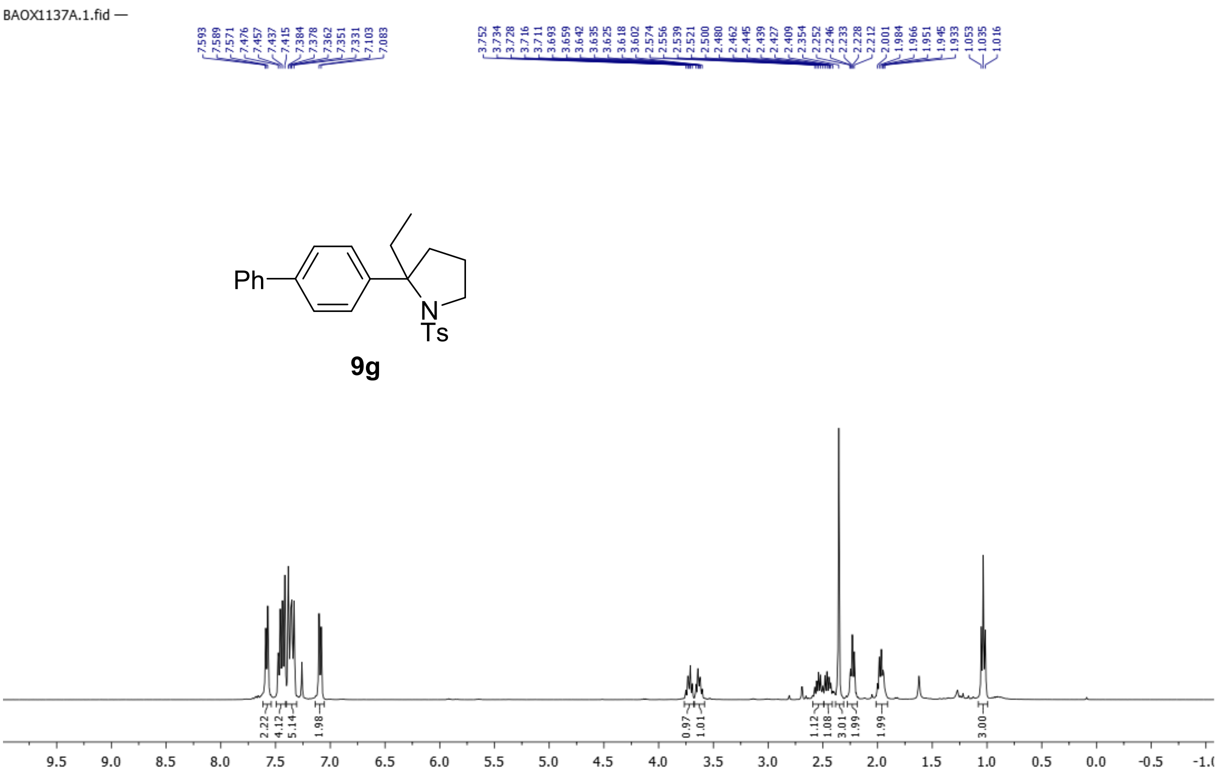

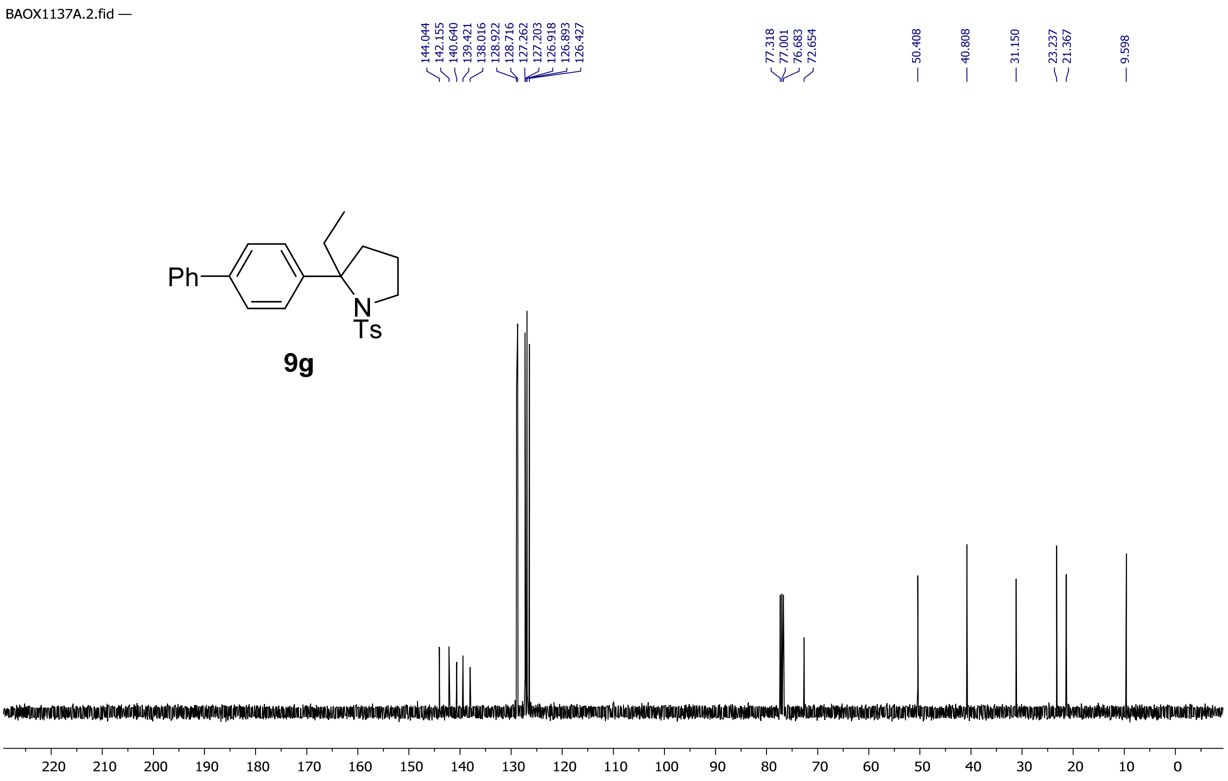

Supplementary Figure 90. ^{1}H and ^{13}C NMR spectra of 9g

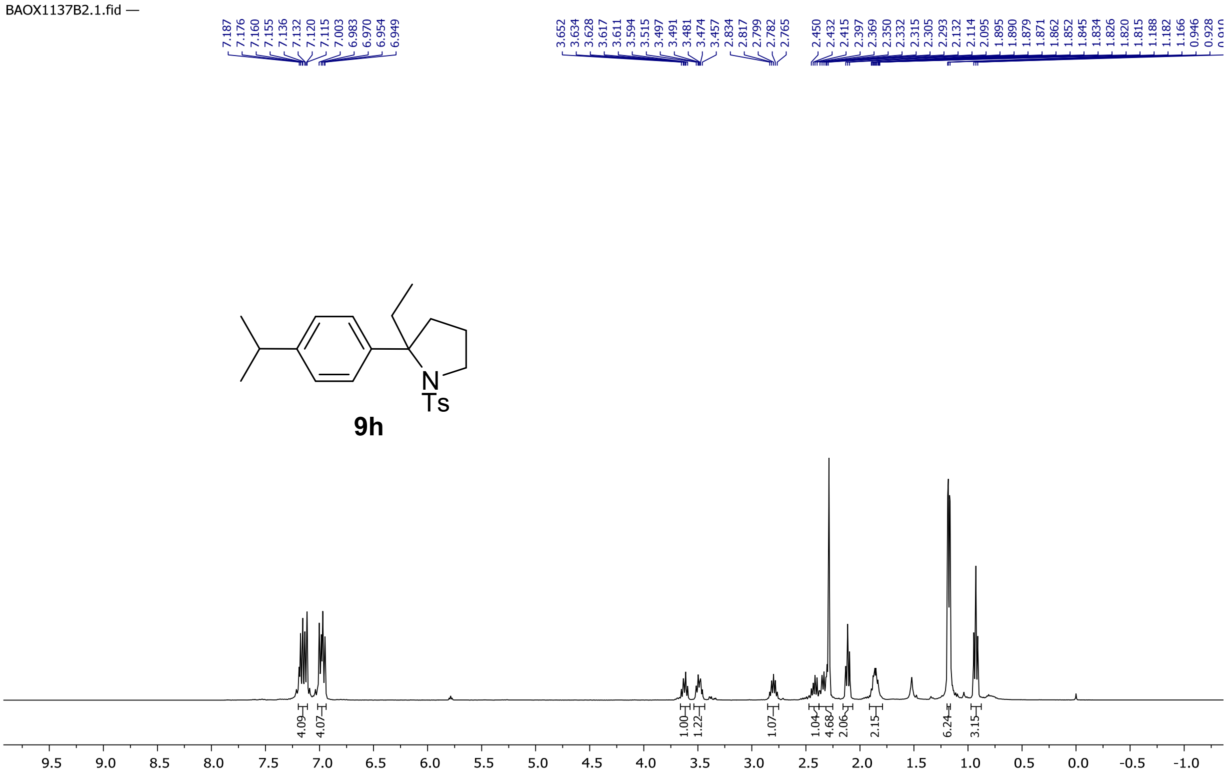

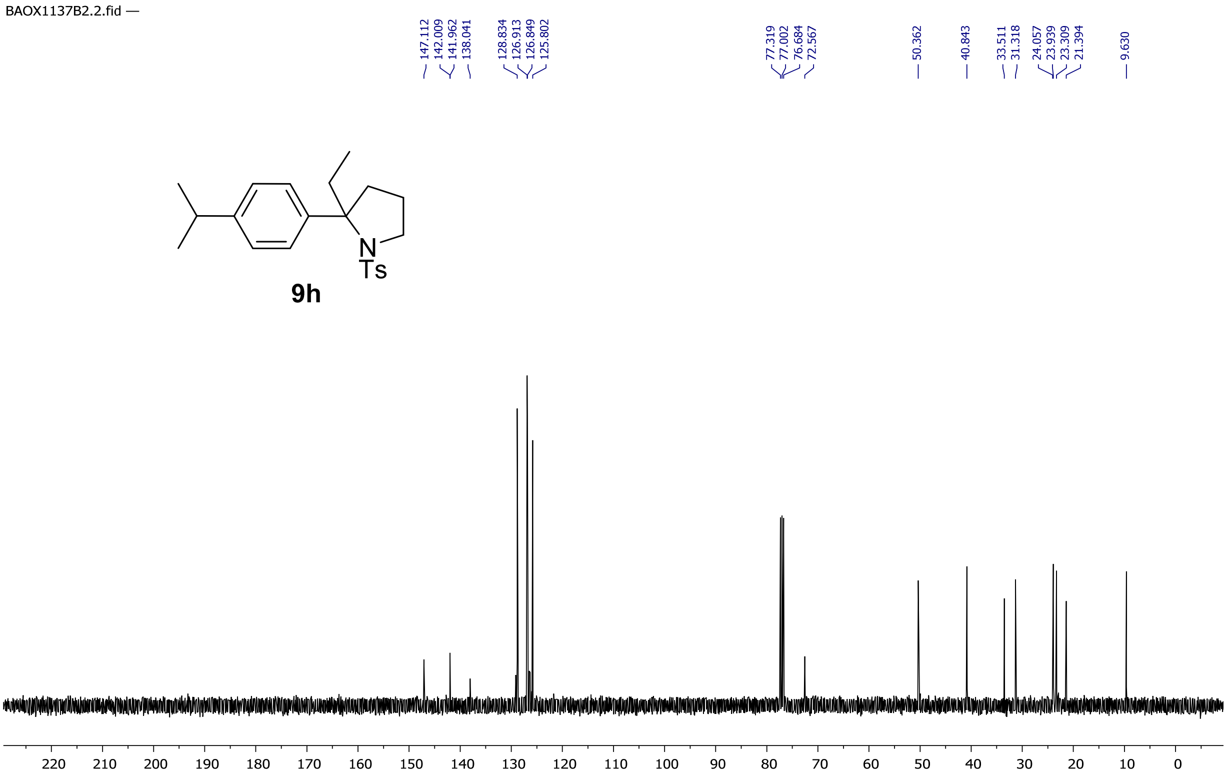

Supplementary Figure 91. ^{1}H and ^{13}C NMR spectra of 9h

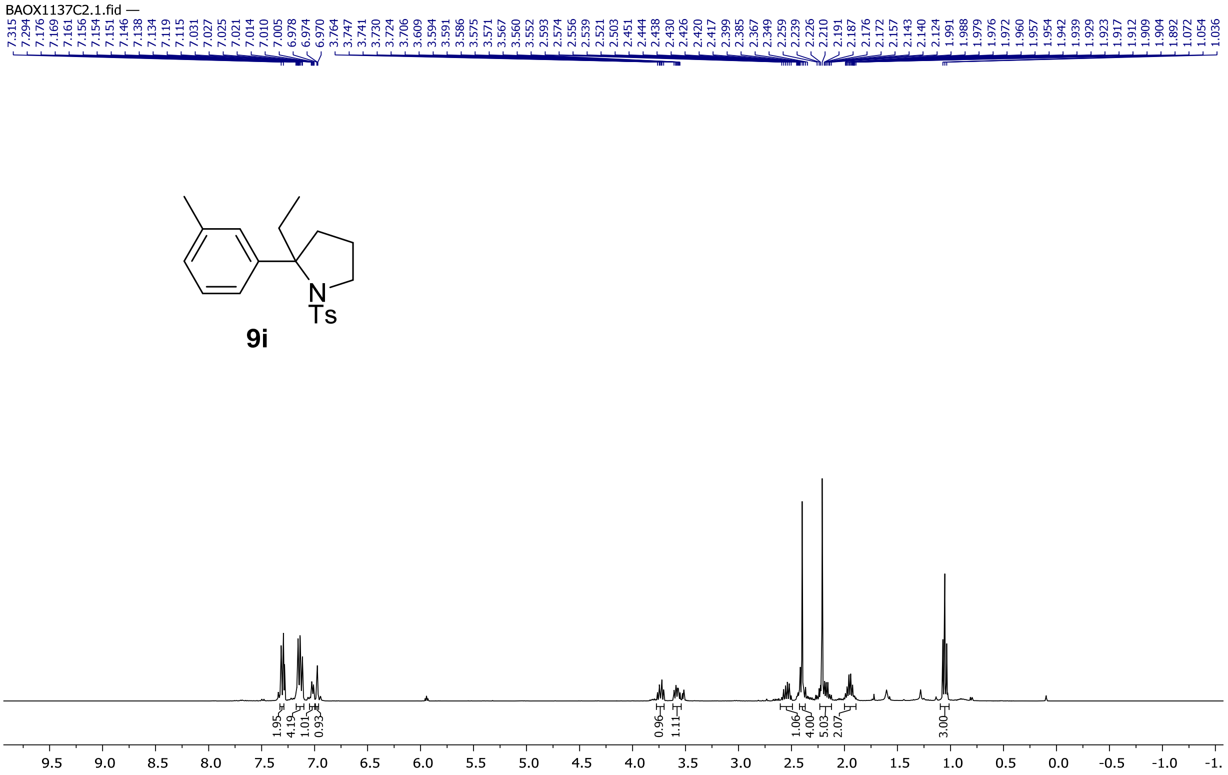

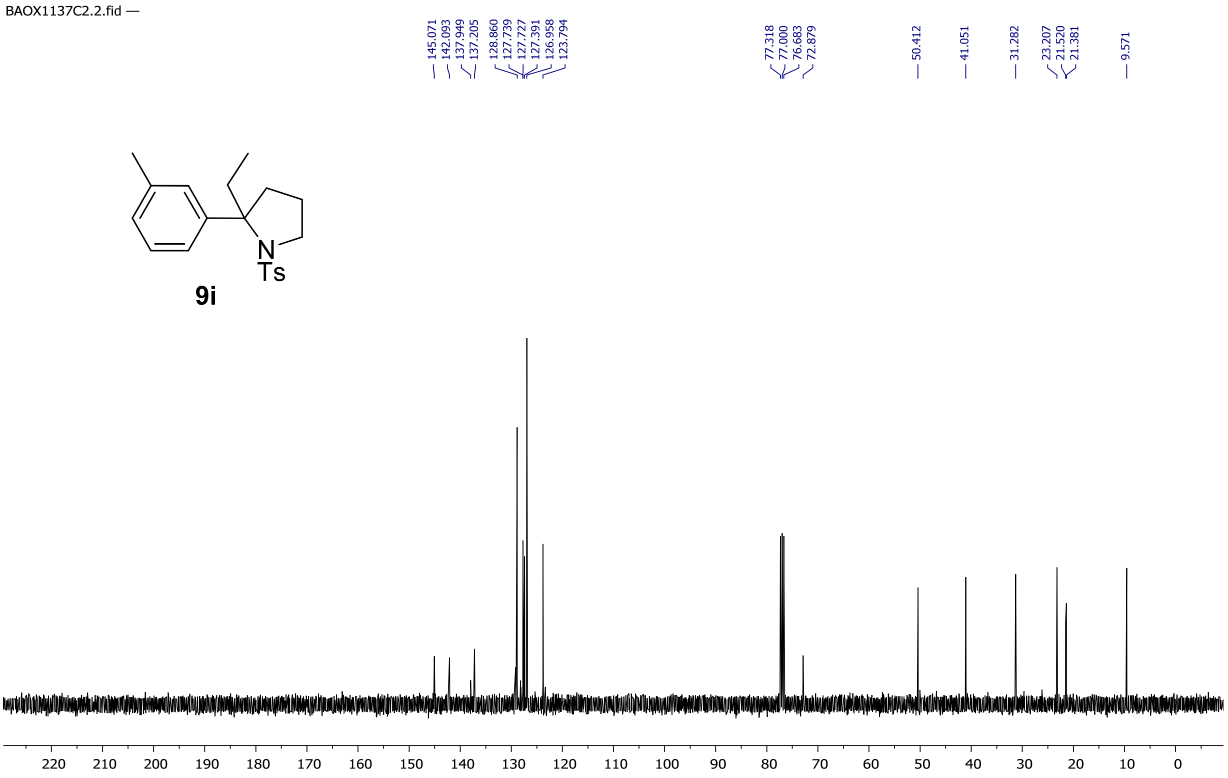

Supplementary Figure 92. ^{1}H and ^{13}C NMR spectra of 9i

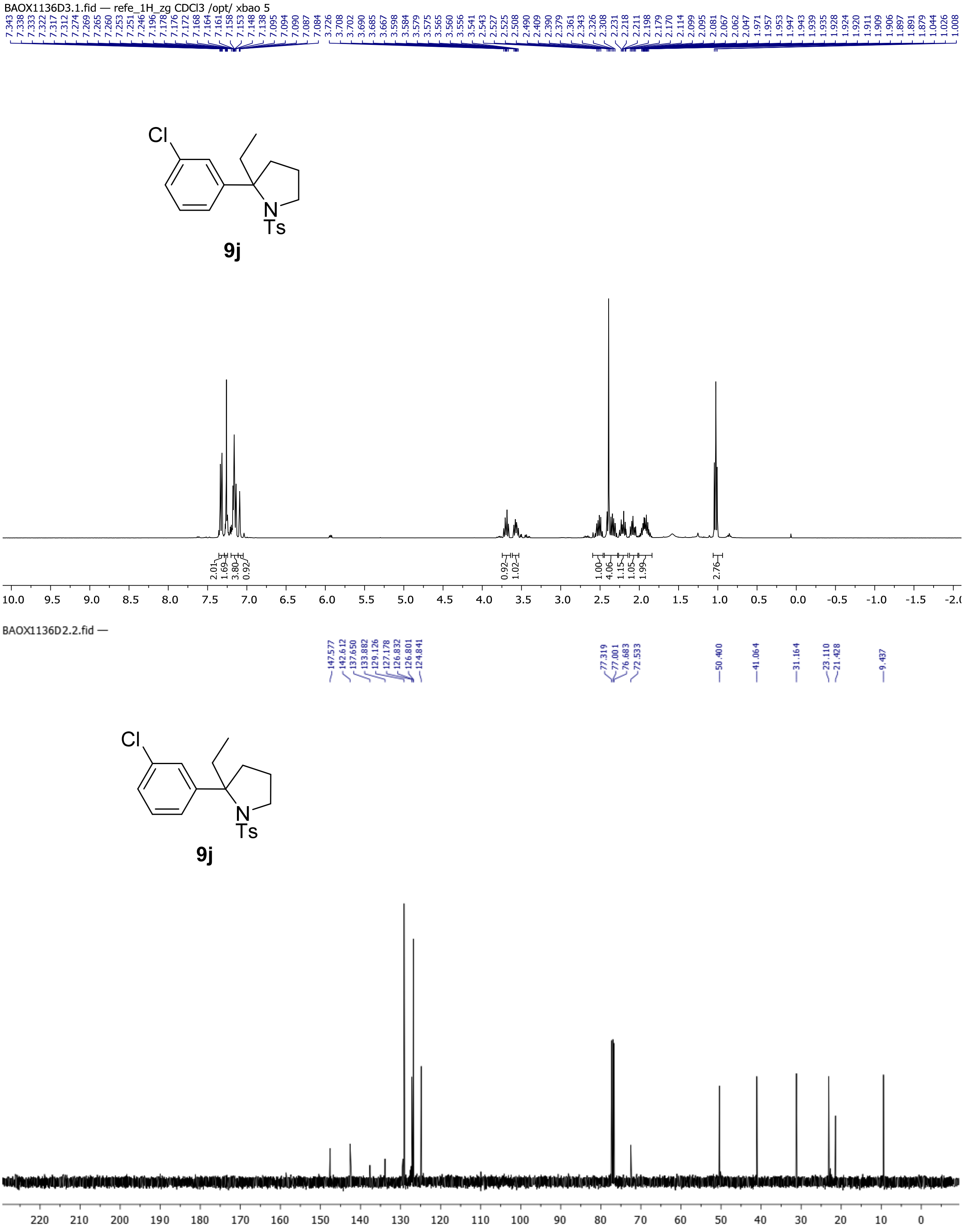

Supplementary Figure 93. ^{1}H and ^{13}C NMR spectra of 9j

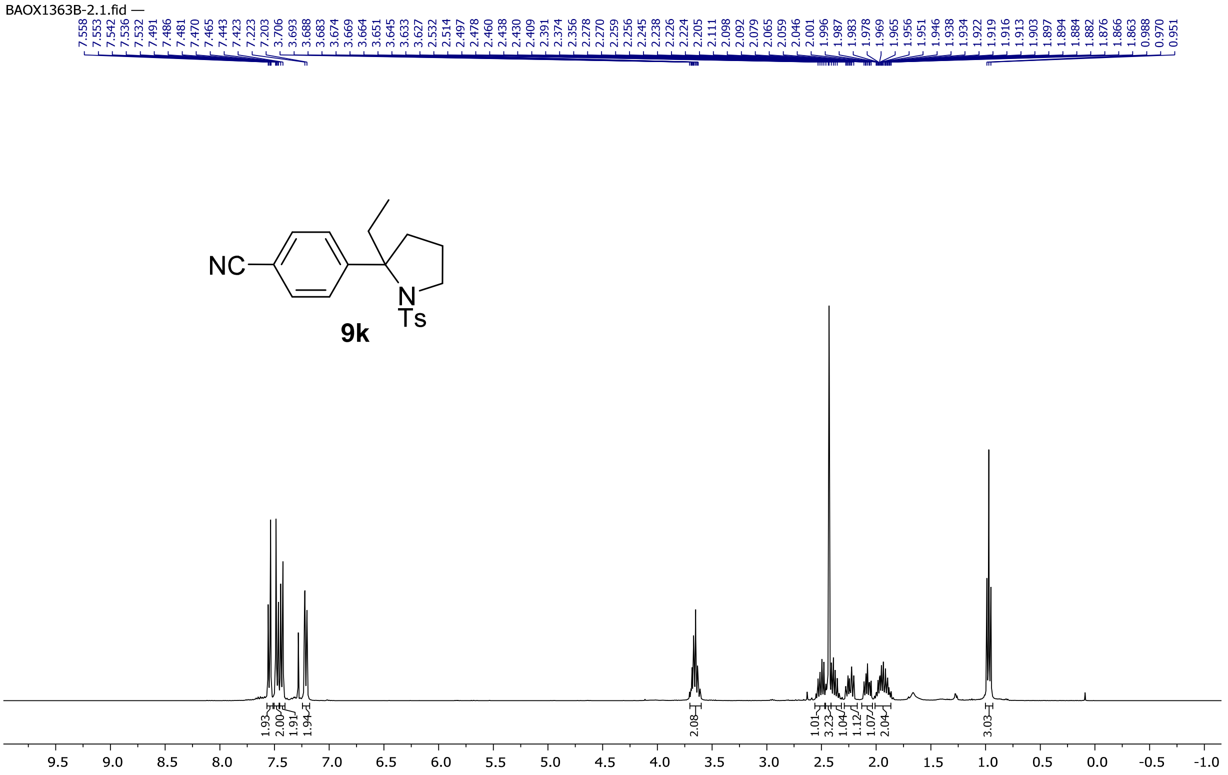

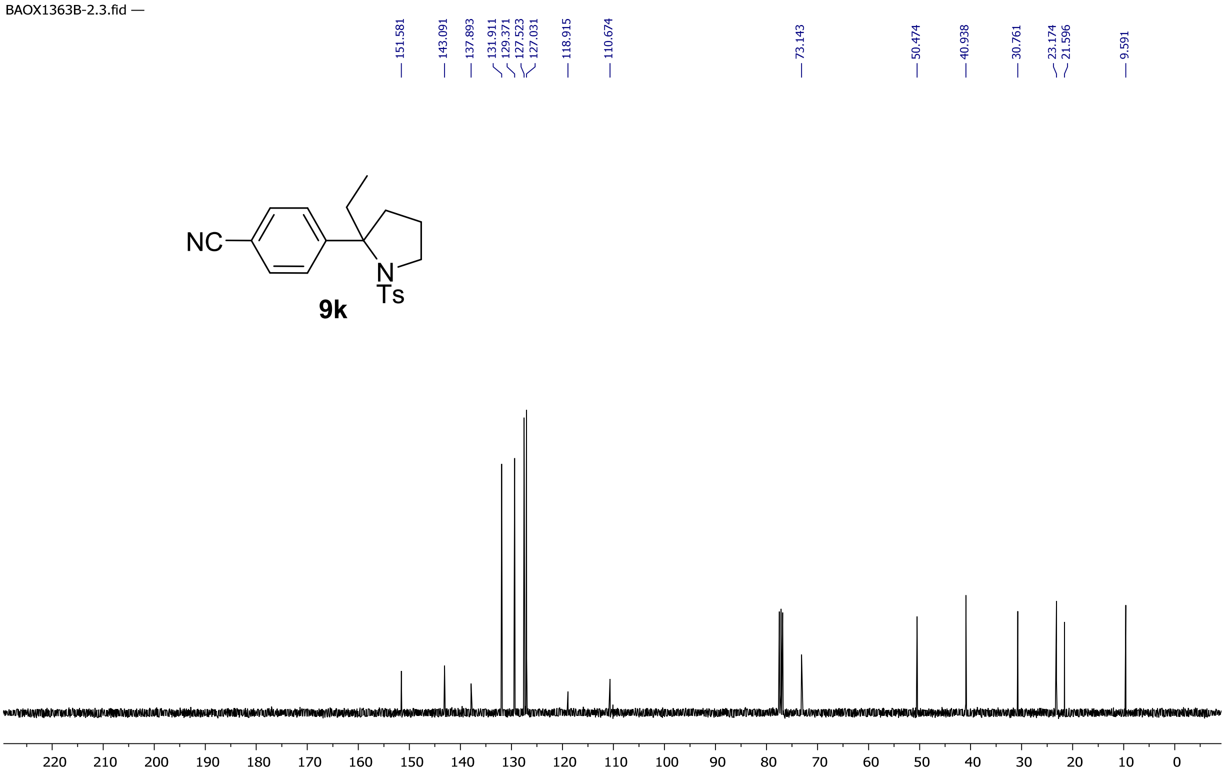

Supplementary Figure 94. ^{1}H and ^{13}C NMR spectra of 9k

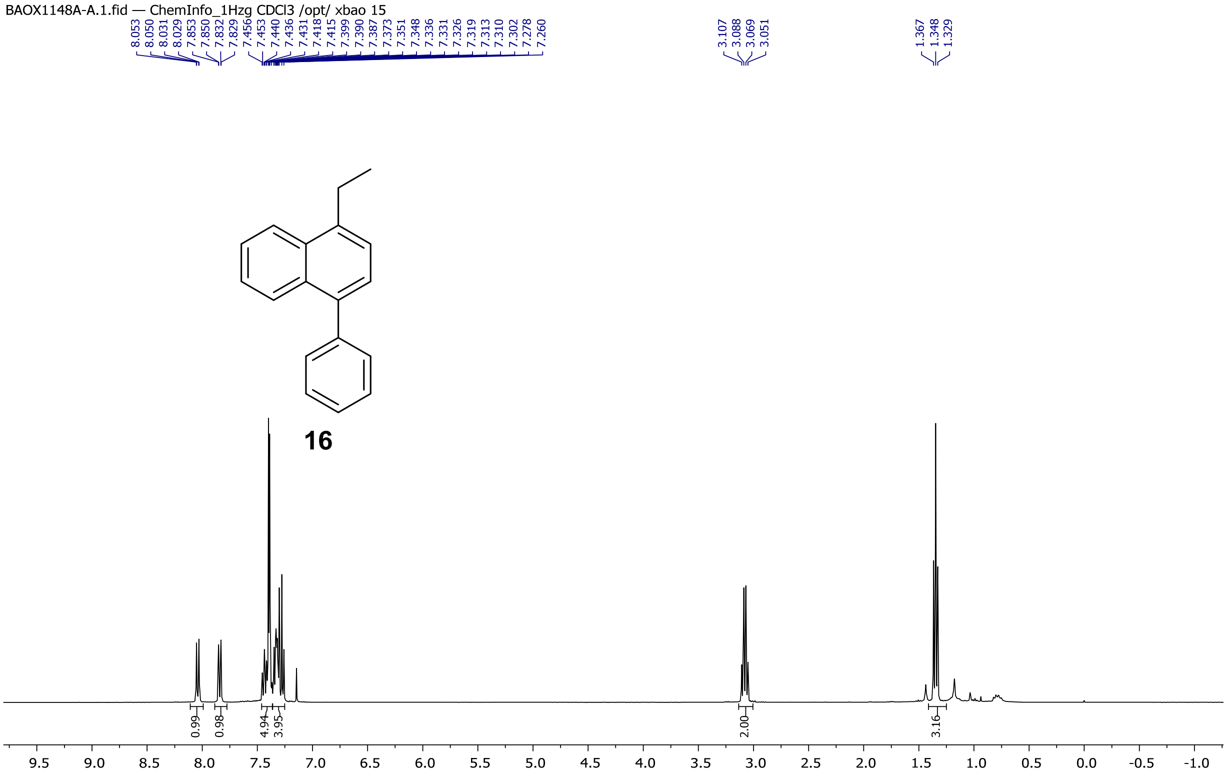

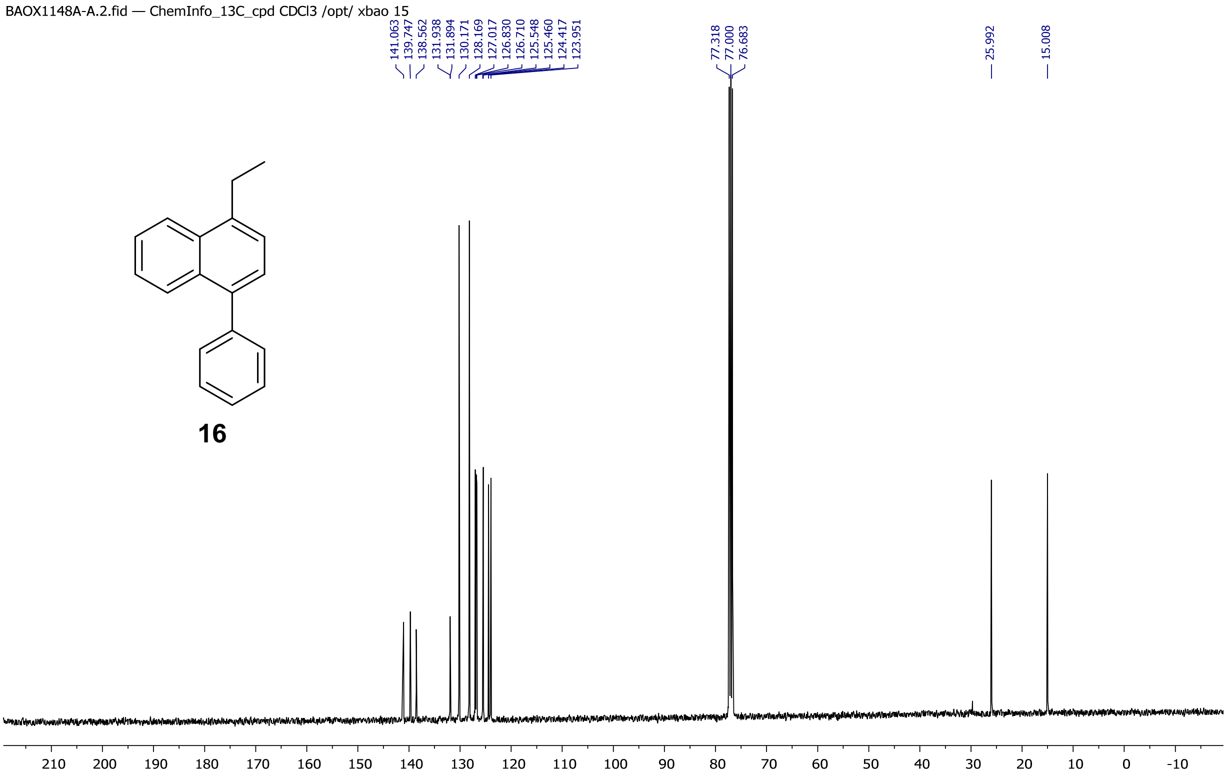

Supplementary Figure 95. ^{1}H and ^{13}C NMR spectra of 16

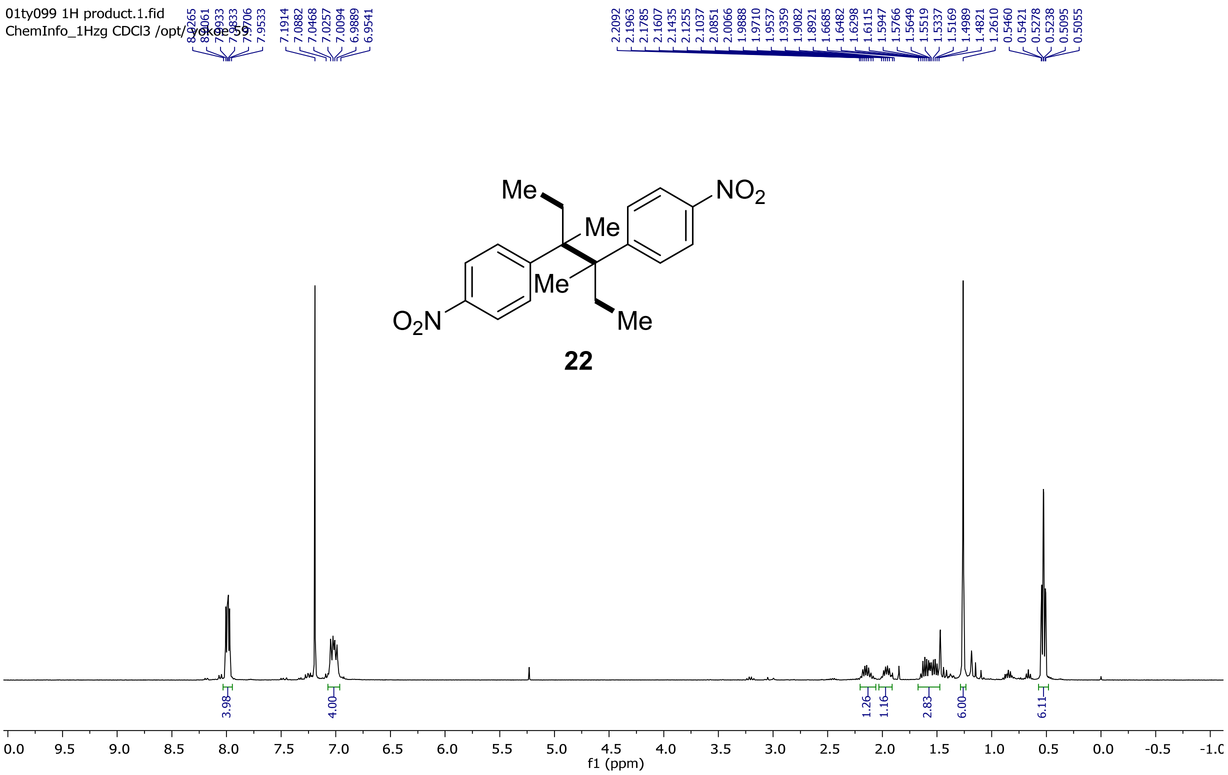

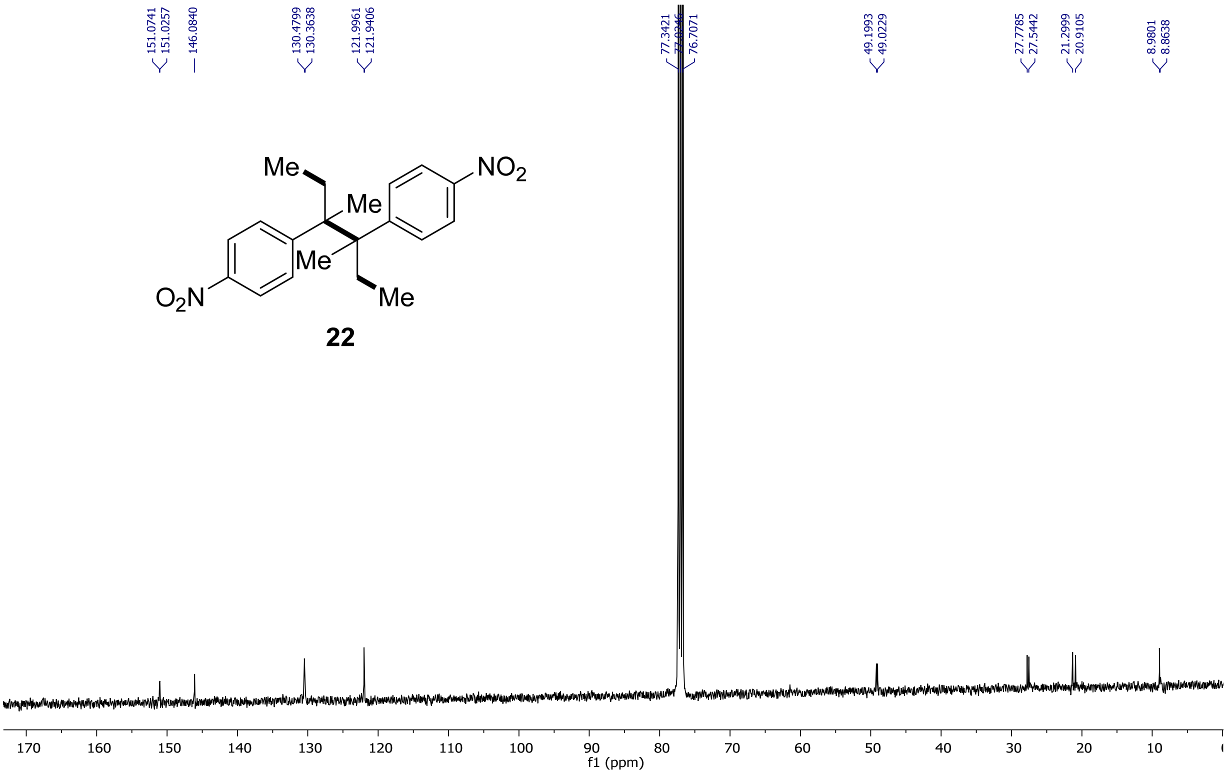

Supplementary Figure 96. ^{1}H and ^{13}C NMR spectra of 22

Supplementary Tables

Supplementary Table 1. 1,2-Methoxy methylation of alkenes: optimization of reaction conditions.[a]

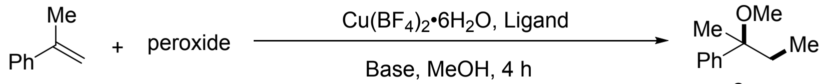

1a **2a**

Entry	Peroxide	Ligand	Base (equiv)	Yield (%)[f]
1	DTBP	**L4**	Na_3PO_4 (1.0)	23
2	DCP	**L4**	Na_3PO_4 (1.0)	52
3	PhCO-O*t*Bu	**L4**	Na_3PO_4 (1.0)	29
4	*t*BuO-OH	**L4**	Na_3PO_4 (1.0)	6
5	DCP	**L2**	Na_3PO_4 (1.0)	52
6	DCP	**L3**	Na_3PO_4 (1.0)	50
7	DCP	**L1**	Na_3PO_4 (1.0)	87
8	DCP	**L1**	Na_3PO_4 (1.0)	44
9[b]	DCP	**L1**	Na_3PO_4 (1.0)	52
10[b]	DCP	**L1**	Na_3PO_4 (0.5)	83
11[c]	DCP	**L1**	Na_3PO_4 (0.2)	80
12[d]	DCP	**L1**	Na_3PO_4 (0.1)	48
13[c]	DCP	**L1**	Na_2HPO_4 (0.2)	93
14[c]	DCP	**L1**	NaH_2PO_4 (0.2)	88
15[c]	DCP	**L1**	Na_3PO_4 (0.2)	61
16[c]	DCP	**L1**	K_2CO_3 (0.2)	19
17[c]	DCP	**L1**	Imidazole (0.2)	26
18[c]	DCP	**L1**	Et_3N (0.2)	34
19[e]	DCP	**L1**	Na_2HPO_4 (0.2)	97 (96)[g]

[a]The reaction was performed in a sealed tube. **1a** (0.2 mmol), DCP (0.8 mmol), $Cu(BF_4)_2$•$6H_2O$ (1.0 equiv), ligand (1.5 equiv), Na_3PO_4 (0.2 mmol), MeOH (4.0 mL, c 0.05 M), 140 °C, 4 h. [b]$Cu(BF_4)_2$•$6H_2O$ (0.5 equiv), **L1** (0.75 equiv). [c]Cu(BF4)2•6H2O (0.2 equiv), **L1** (0.3 equiv). [d]$Cu(BF_4)_2$•$6H_2O$ (0.1 equiv), **L1** (0.15 equiv). [e]$Cu(BF_4)_2$•$6H_2O$ (0.2 equiv), **L1** (0.3 equiv), c 0.1 M, 120 °C. [f]Determined by ^1H NMR spectroscopy using CH_2Br_2 as an internal standard. [g]Yield of isolated product in parenthesis. DCP = dicumyl peroxide; DTBP = di-tertbutyl peroxide.

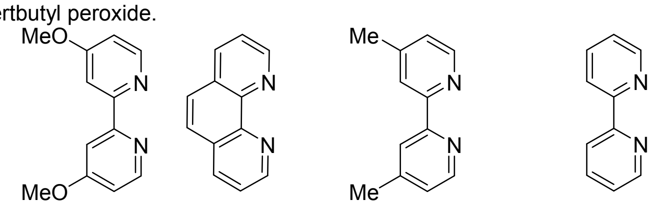

L1 **L2** **L3** **L4**

Supplementary Table 2 . 1,2-Azido methylation of alkenes: optimization of reaction conditions.[a]

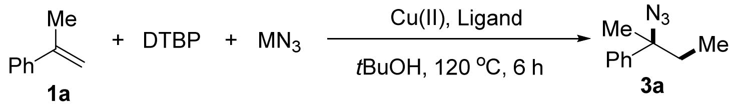

Entry	MN_3	Cu salt	Ligand	Yield (%)[f]
1	NaN_3	$Cu(BF_4)_2$•$6H_2O$	---	50
2	LiN_3	$Cu(BF_4)_2$•$6H_2O$	---	55
3	KN_3	$Cu(BF_4)_2$•$6H_2O$	---	22
4	LiN_3	$Cu(BF_4)_2$•$6H_2O$	**L1**	47
5	LiN_3	$Cu(BF_4)_2$•$6H_2O$	**L4**	37
6	LiN_3	$Cu(BF_4)_2$•$6H_2O$	**L3**	26
7	LiN_3	$Cu(BF_4)_2$•$6H_2O$	**L2**	59
8	LiN_3	$Cu(OAc)_2$	**L2**	58
9	LiN_3	$Cu(OTf)_2$	**L2**	54
10	LiN_3	CuF_2	**L2**	59
11	LiN_3	$CuSO_4$	**L2**	73
12[b]	LiN_3	$CuSO_4$	**L2**	72
13[c]	LiN_3	$CuSO_4$	**L2**	82
14[d]	LiN_3	$CuSO_4$	**L2**	84 (81)[g]
15[d,e]	LiN_3	$CuSO_4$	**L2**	82

[a]**1a** (0.2 mmol), MN_3 (1.0 mmol), Cu(II) (0.04 mmol), ligand (0.06 mmol), DTBP (0.8 mmol), *t*BuOH (2.0 mL). [b]$CuSO_4$ (0.02 mmol, 0.1 equiv), **L2** (0.03 mmol), 120 °C. [c]$CuSO_4$ (0.01 mmol, 0.05 equiv), **L2** (0.015 mmol). [d]$CuSO_4$ (0.002 mmol, 0,01 equiv), **L2** (0.006 mmol). eaqueous solution of LiN_3 (0.5 mmol, 2.5 equiv). [f]Yield was determined by ^1H NMR spectroscopy with DMAP as an internal standard. [g]Yield of isolated product in parenthesis.

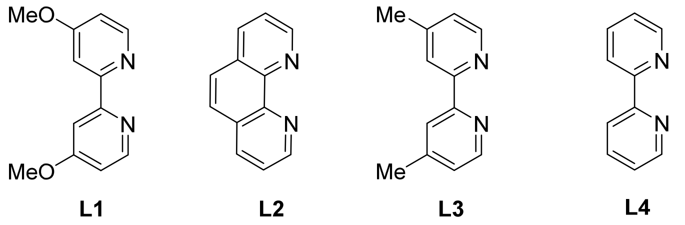

Supplementary Table 3 . Copper-catalyzed methylative alkoxylation of alkene 4a providing tetrahydrofuran 5a: optimization of reaction conditions.[a]

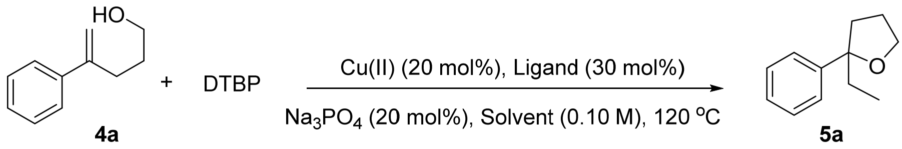

Entry	Cu(II)	Solvent	Ligand	Yield[b]
1	$Cu(BF_4)_2 \cdot 6H_2O$	^{t}BuOH	**L1**	57%
2	$Cu(BF_4)_2 \cdot 6H_2O$	^{t}BuCN	**L1**	30%
3	$Cu(BF_4)_2 \cdot 6H_2O$	CF_3CH_2OH	**L1**	81%
4	$Cu(OAc)_2$	CF_3CH_2OH	**L1**	52%
5	$CuSO_4$	CF_3CH_2OH	**L1**	59%
6	$Cu(OTf)_2$	CF_3CH_2OH	**L1**	84% (82%)[c]
7	CuF_2	CF_3CH_2OH	**L1**	54%
8	$Cu(OTf)_2$	CF_3CH_2OH	**L4**	62%
9	$Cu(OTf)_2$	CF_3CH_2OH	**L3**	63%
10	$Cu(OTf)_2$	CF_3CH_2OH	**L5**	63%
11	$Cu(OTf)_2$	CF_3CH_2OH	**L2**	32%

[a]**4a** (0.2 mmol), Cu(II) (0.04 mmol), ligand (0.06 mmol), DTBP (0.8 mmol), Na_3PO_4 (0.04 mmol), solvent (2.0 mL). [b]Yield was determined by ^1H NMR spectroscopy with DMAP as an internal standard. [c]Yield of the isolated product in parenthesis.

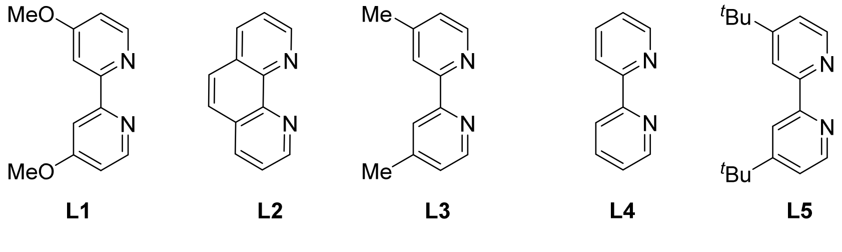

Supplementary Table 4 . Copper-catalyzed methylative lactonization of alkene 6a providing lactone 7a: optimization of reaction conditions.[a]

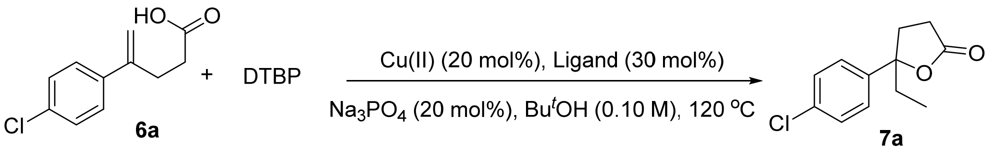

Entry	Cu(II)	Ligand	Yield[b]
1	$Cu(BF_4)_2$•$6H_2O$	**L1**	73%
2	$Cu(OAc)_2$	**L1**	69%
3	$CuSO_4$	**L1**	81% (76%)[c]
4	$Cu(OTf)_2$	**L1**	64%
5	$Cu(acac)_2$	**L1**	62%
6	$CuSO_4$	**L4**	76%
7	$CuSO_4$	**L3**	68%
8	$CuSO_4$	**L5**	69%
9	$CuSO_4$	**L2**	56%

[a]**6a** (0.2 mmol), Cu(II) (0.04 mmol), ligand (0.06 mmol), DTBP (0.8 mmol), Na_3PO_4 (0.04 mmol), ^{t}BuOH (2.0 mL). [b]Yield was determined by ^{1}H NMR spectroscopy with DMAP as an internal standard. [c]Yield of the isolated product in parenthesis.

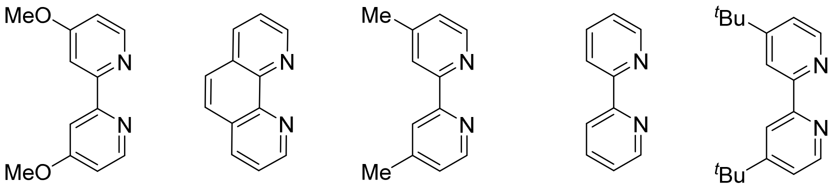

L1 **L2** **L3** **L4** **L5**

Supplementary Table 5. Copper-catalyzed methylative cycloamination of alkene 8a: optimization of reaction conditions.

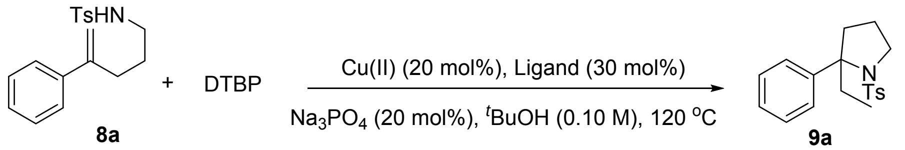

8a **9a**

Entry	Cu(II)	Ligand	Yield[b]
1	$Cu(BF_4)_2{\bullet}6H_2O$	**L1**	36%
2	$Cu(OAc)_2$	**L1**	65%
3	$CuSO_4$	**L1**	Trace
4	CuF_2	**L1**	Trace
5	$Cu(OTf)_2$	**L1**	27%
6	$Cu(OAc)_2$	**L5**	53%
7	$Cu(OAc)_2$	**L3**	44%
8	$Cu(OAc)_2$	**L6**	42%
9	$Cu(OAc)_2$	**L2**	75% (71%)[c]

[a]**8a** (0.2 mmol), Cu(II) (0.04 mmol), ligand (0.06 mmol), DTBP (0.8 mmol), ^{t}BuOH (2.0 mL). [b]Yield was determined by ^{1}H NMR spectroscopy with DMAP as an internal standard. [c]Yield of the isolated product in parenthesis.

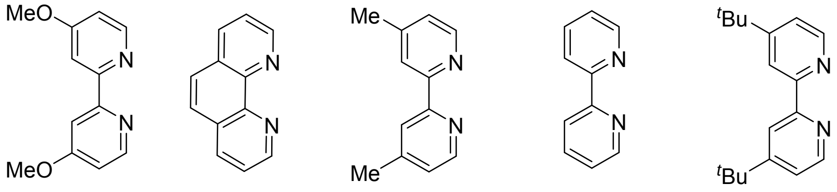

L1 **L2** **L3** **L4** **L5**

Supplementary Methods

General Information.

General Analytical Information.

NMR spectra were recorded on a Brüker AvanceIII-400, Brüker Avance-400 or Brüker DPX-400 spectrometer at room temperature, ^{1}H frequency is at 400.13 MHz, ^{13}C frequency is at 100.62 MHz. Chemical shifts (δ) were reported in parts per million (ppm) relative to residual solvent peaks rounded to the nearest 0.01 for proton and 0.1 for carbon (*ref: CHCl3 [^{1}H: 7.26, ^{13}C: 77.16]*. Coupling constants (*J*) were reported in Hz to the nearest 0.1 Hz. Peak multiplicity was indicated as follows s (singlet), d (doublet), t (triplet), q (quartet), m (multiplet) and br (broad). Attribution of peaks was done using the multiplicities and integrals of the peaks.

IR spectra were recorded in a Jasco FT/IR-4100 spectrometer outfitted with a PIKE technology MIRacleTM ATR accessory as neat films compressed onto a Zinc Selenide window. The spectra were reported in cm^{-1}.

The accurate masses were measured by the mass spectrometry service of the EPFL by ESI-TOF using a QTOF Ultima from Waters or APPI-FT-ICR using a linear ion trap Fourier transform ion cyclotron resonance mass spectrometer from Thermo Scientific.

Melting points were measured using a Stuart SMP30

Materials and Methods.

Unless otherwise stated, starting materials were purchased from Aldrich and/or Fluka. Solvents were purchased in HPLC quality, degassed by purging thoroughly with nitrogen and dried over activated molecular sieves of appropriate size. Alternatively, they were purged with argon and passed through alumina columns in a solvent purification system (Innovative Technology). Conversion was monitored by thin layer chromatography (TLC) using Merck TLC silica gel 60 F254. Compounds were visualized by UV light at 254 nm and by dipping the plates in an ethanolic vanillin/sulfuric acid solution or an aqueous potassium permanganate solution followed by heating. Flash column chromatography was performed over silica gel (230–400 mesh).

General procedure

Synthesis and characterization data of alcohols 4

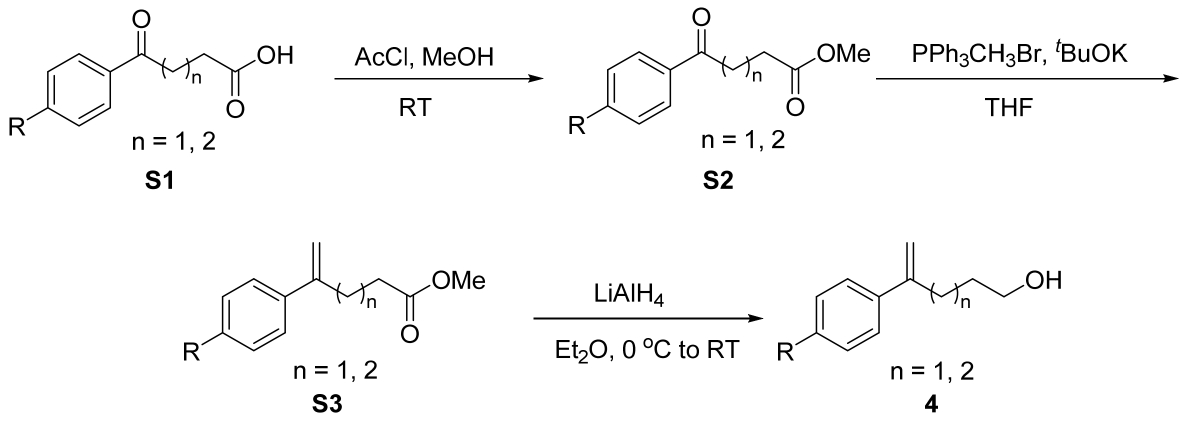

General procedure **A**:

To a solution of **S1** (10.0 mmol) in MeOH (15 mL) was added AcCl (cat.) at 0 °C, After being stirred at room temperature for 9 h, the solvent was evaporated under reduced pressure to give the crude product **S2** which was used for the next reaction without further purification.

To a suspension of PPh_3CH_3Br (3.93 g, 11.0 mmol, 1.1 equiv) in THF (30 mL) was added ^{t}BuOK (1.23 g, 11.0 mmol, 1.1 equiv) at 0 °C. The reaction mixture was stirred at room temperature for 0.5 h, then cooled down to 0 °C. A solution of **S2** obtained above in THF (5 mL) was added. After being stirred at room temperature for 10 h, the reaction was quenched by addition of water. The organic phase was separated and the aqueous phase was extracted with Et_2O. The combined organic extracts were washed with brine, dried over Na_2SO_4. The solvent was evaporated under reduced pressure. The residue was purified by flash chromatograph (SiO_2, eluent: PE/EtOAc = 10/1) to afford the desired product **S3**.

To a suspension of $LiAlH_4$ (0.38 g, 10.0 mmol, 2.0 equiv) in Et_2O (20 mL) was added a solution of **S3** (5.0 mmol, 1.0 equiv) in Et_2O (3 mL) at 0 ºC. The reaction mixture was stirred at at room temperature for 4 h, then cooled to 0 ºC, and water (0.4 mL), 15% NaOH (0.4 mL), water (1.2 mL) were added dropwise sequentially. The reaction mixture was stirred for 15 min., then filtered through Celite, and the filtrate was evaporated under reduced pressure. The residue was purified by flash chromatograph (SiO_2, eluent: PE/EtOAc = 4/1) to afford the desired product **4**.

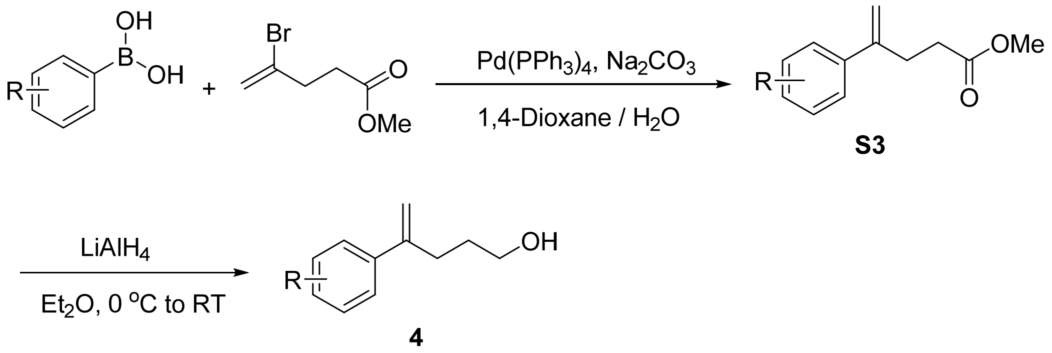

General procedure **B**:

To a solution of methyl 4-bromopent-4-enoate (0.96 g, 5.0 mmol, 1.0 equiv) and aryl boronic acid (6.0 mmol, 1.2 equiv) in 1,4-dioxane/H_2O (70 mL / 10 mL) was added $Pd(PPh_3)_4$ (0.58 g) and Na_2CO_3 (1.17 g, 11.0 mmol, 2.2 equiv) under N_2 atmosphere. After being stirred at 110 °C for 10 h, the reaction mixture was quenched by addition of aqueous water. The organic phase was separated and the aqueous phase was extracted with Et_2O. The combined organic extracts were washed with brine, dried over Na_2SO_4. The solvent was evaporated under reduced pressure. The residue was purified by flash chromatograph (SiO_2, eluent: PE/EtOAc = 10/1) to afford the desired product S**3**.

To a suspension of $LiAlH_4$ (0.30 g, 8.0 mmol, 2.0 equiv) in Et_2O (16 mL) was added a solution of **S3** (4.0 mmol, 1.0 equiv) in Et_2O (2 mL) at 0 °C. The reaction mixture was stirred at room temperature for 4 h, then cooled to 0 °C, and water (0.3 mL), 15% NaOH (0.3 mL), water (0.9 mL) were added dropwise sequentially. The reaction mixture was stirred for 15 min., then filtered through Celite, and tand the filtrate was evaporated under reduced pressure. The residue was purified by flash chromatograph (SiO_2, eluent: PE/EtOAc = 4/1) to afford the desired product **4**.

Synthesis of 4-phenylpent-4-en-1-ol (4a)

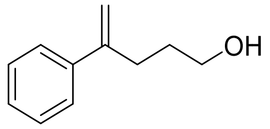

4a

Prepared according to general procedure **A.** Colorless oil. The physical and spectroscopic data were in accordance with those reported in the literature.[1]

^{1}H NMR (400 MHz, $CHCl_3$) δ 7.34 (d, J = 7.6 Hz, 2H), 7.28–7.23 (m, 2H), 7.21–7.18 (m, 1H), 5.22 (s, 1H), 5.02 (s, 1H), 3.59 (t, J = 6.0 Hz, 2H), 2.53 (t, J = 7.7 Hz, 2H), 1.71–1.60 (m, 2H).

Synthesis of 4-(p-tolyl)pent-4-en-1-ol (4b)

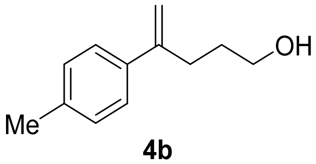

4b

Prepared according to general procedure **A**. Colorless oil. The physical and spectroscopic data were in accordance with those reported in the literature.[1]

^{1}H NMR (400 MHz, $CHCl_3$) δ 7.42–7.26 (m, 2H), 7.15 (d, J = 7.9 Hz, 2H), 5.29 (s, 1H), 5.06 (s, 1H), 3.66 (t, J = 6.5 Hz, 2H), 2.71–2.55 (m, 2H), 2.36 (s, 3H), 1.85 –1.60 (m, 2H).

Synthesis of 4-(4-methoxyphenyl)pent-4-en-1-ol (4c)

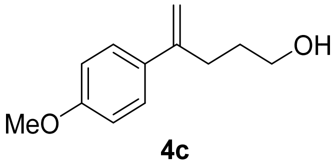

4c

Prepared according to general procedure **A**. Colorless oil. The physical and spectroscopic data were in accordance with those reported in the literature.[1]

^{1}H NMR (400 MHz, $CHCl_3$) δ 7.36 (d, J = 7.9 Hz, 2H), 6.86 (d, J = 7.9 Hz, 2H), 5.23 (s, 1H), 5.01 (s, 1H), 3.81 (s, 3H), 3.72–3.57 (m, 2H), 2.58 (t, J = 7.5 Hz, 2H), 1.76–1.69 (m, 2H).

Synthesis of 4-(4-fluorophenyl)pent-4-en-1-ol (4d)

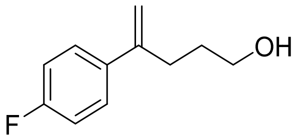

4d

Prepared according to general procedure **A**. Colorless oil. The physical and spectroscopic data were in accordance with those reported in the literature.[71]

^{1}H NMR (400 MHz, $CHCl_3$) δ 7.49–7.31 (m, 2H), 7.03–6.98 (m, 2H), 5.24 (s, 1H), 5.08 (s, 1H), 3.66 (t, J = 6.5 Hz, 2H), 2.58 (t, J = 7.7 Hz, 2H), 1.82–1.57 (m, 2H).

Synthesis of 4-(4-chlorophenyl)pent-4-en-1-ol (4e)

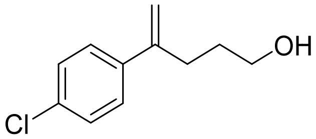

4e

Prepared according to general procedure **A**. Colorless oil. The physical and spectroscopic data were in accordance with those reported in the literature.[1]

¹H NMR (400 MHz, $CHCl_3$) δ 7.34 (d, J = 8.6 Hz, 2H), 7.29 (d, J = 8.6 Hz, 2H), 5.28 (s, 1H), 5.11 (d, J = 1.5 Hz, 1H), 3.66 (t, J = 6.4 Hz, 2H), 2.57 (t, J = 7.6 Hz, 2H), 1.87–1.65 (m, 2H).

Synthesis of 4-(4-bromophenyl)pent-4-en-1-ol (4f)

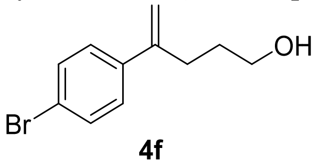

4f

Prepared according to general procedure **A**. Colorless oil. The physical and spectroscopic data were in accordance with those reported in the literature.[1]

¹H NMR (400 MHz, $CHCl_3$) δ 7.56–7.42 (m, 2H), 7.35–7.24 (m, 2H), 5.32 (s, 1H), 5.14 (s, 1H), 3.69 (t, J = 6.5 Hz, 2H), 2.60 (t, J = 7.6 Hz, 2H), 1.90–1.68 (m, 2H).

Synthesis of 4-([1,1'-biphenyl]-4-yl)pent-4-en-1-ol (4g)

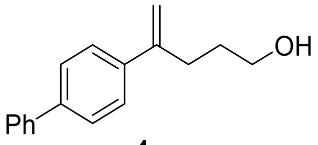

4g

Prepared according to general procedure **A**. White solid.

MP: 89–90 °C

¹H NMR (400 MHz, $CHCl_3$) δ 7.60 (d, J = 7.3 Hz, 2H), 7.57 (d, J = 8.3 Hz, 2H), 7.50 (d, J = 8.3 Hz, 2H), 7.44 (t, J = 7.5 Hz, 2H), 7.37–7.32 (m, 1H), 5.37 (s, 1H), 5.13 (s, 1H), 3.70 (t, J = 6.4 Hz, 2H), 2.65 (t, J = 7.6 Hz, 2H), 1.98–1.71 (m, 2H), 1.41 (s, 2H).

¹³C NMR (101 MHz, $CDCl_3$) δ 147.4, 140.7, 140.3, 139.8, 128.8, 127.3, 127.03, 126.97, 126.5, 112.6, 62.4, 31.5, 31.2.

IR (neat) cm^{-1} υ: 3304 (m), 1622 (w), 1486 (w), 1447 (w), 1405 (w), 1061 (m), 1052 (m), 1025 (m), 896 (w), 843 (s), 770 (m), 735 (s), 690 (s).

HRMS (APPI) m/z: $[M + H]^+$ Calcd for $C_{17}H_{19}O^+$ 239.1430; Found 239.1436.

Synthesis of 4-(4-isopropylphenyl)pent-4-en-1-ol (4h)

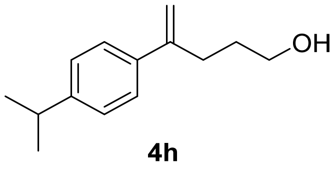

4h

Prepared according to general procedure **A**. Colorless oil.

^{1}H NMR (400 MHz, $CHCl_3$) δ 7.35 (d, J = 8.3 Hz, 2H), 7.19 (d, J = 8.0 Hz, 2H), 5.29 (d, J = 1.5 Hz, 1H), 5.06 (d, J = 1.7 Hz, 1H), 3.67 (t, J = 6.5 Hz, 2H), 2.91 (hept, J = 7.0 Hz, 1H), 2.60 (t, J = 7.6 Hz, 2H), 1.83 – 1.68 (m, 2H), 1.41 (brs, 1H), 1.26 (d, J = 6.9 Hz, 6H).

^{13}C NMR (101 MHz, $CDCl_3$) δ 148.2, 147.7, 138.3, 126.3, 126.0 111.8, 62.5, 33.7, 31.5, 31.2, 24.0

IR (neat) cm^{-1} υ: 3309 (m), 1625 (w), 1512 (w), 1460 (w), 1363 (w), 1056 (m), 1017 (w), 891 (m), 838 (s), 759 (w), 698 (w).

HRMS (APPI) m/z: $[M + H]^+$ Calcd for $C_{14}H_{21}O^+$ 205.1587; Found 205.1591.

Synthesis of 4-(m-tolyl)pent-4-en-1-ol (4i)

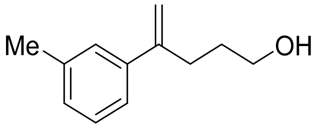

4i

Prepared according to general procedure **B**. Colorless oil. The physical and spectroscopic data were in accordance with those reported in the literature.[2]

^{1}H NMR (400 MHz, $CHCl_3$) δ 7.25–7.17 (m, 3H), 7.15–7.03 (m, 1H), 5.28 (d, J = 1.4 Hz, 1H), 5.08 (d, J = 1.4 Hz, 1H), 3.67 (t, J = 6.5 Hz, 2H), 2.74–2.55 (m, 2H), 2.36 (s, 3H), 1.83–1.63 (m, 2H).

Synthesis of 4-(3-methoxyphenyl)pent-4-en-1-ol (4j)

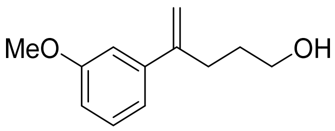

4j

Prepared according to general procedure **B**. Colorless oil.

^{1}H NMR (400 MHz, $CHCl_3$) δ 7.27 (t, J = 7.9 Hz, 1H), 7.06–7.01 (m, 1H), 6.98 (s, 1H), 6.85 (dd, J = 8.2, 2.5 Hz, 1H), 5.32 (s, 1H), 5.12 (s, 1H), 3.84 (s, 3H), 3.68 (t, J = 6.5 Hz, 2H), 2.72–2.57 (m, 2H), 1.84–1.68 (m, 2H), 1.67 (brs, 1H, OH).

^{13}C NMR (101 MHz, $CDCl_3$) δ 159.7, 148.0, 142.7, 129.4, 118.8, 112.9, 112.7, 112.3, 62.5, 55.3, 31.7, 31.3.

IR (neat) cm^{-1} υ: 3335 (m), 1598 (w), 1576 (m), 1488 (w), 1430 (w), 1326 (w), 1286 (m), 1230 (m), 1169 (w), 1041 (s), 882 (m), 857 (m), 786 (s), 729 (m).

HRMS (APPI) m/z: $[M]^+$ Calcd for $C_{12}H_{16}O_2^+$ 192.1145; Found 192.1148.

Synthesis of 4-(3-chlorophenyl)pent-4-en-1-ol (4k)

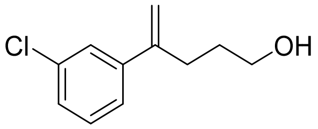

4k

Prepared according to general procedure **B**. Colorless oil.

^{1}H NMR (400 MHz, $CHCl_3$) δ 7.26–7.09 (m, 4H), 5.24 (s, 1H), 5.06 (s, 1H), 3.60 (t, J = 6.4 Hz, 2H), 2.51 (td, J = 7.5, 1.3 Hz, 2H), 1.68–1.61 (m, 2H), 1.27 (brs, 1H).

^{13}C NMR (101 MHz, $CDCl_3$) δ 146.8, 143.0, 134.3, 129.7, 127.5, 126.4, 124.4, 113.8, 62.3, 31.5, 31.1.

IR (neat) cm^{-1} υ: 3309 (m), 2945 (w), 1593 (w), 1562 (w), 1476 (w), 1412 (w), 1096 (w), 1057 (m), 901 (m), 884 (m), 790 (s), 726 (m).

HRMS (ESI) m/z: $[M{-}H_2O{+}H]^+$ Calcd for $C_{11}H_{12}Cl^+$ 179.0622; Found 179.0613.

Synthesis of 4-(3,5-dimethylphenyl)pent-4-en-1-ol (4l)

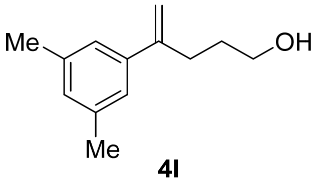

4l

Prepared according to general procedure **B**. Colorless oil.

^{1}H NMR (400 MHz, $CHCl_3$) δ 7.03 (s, 2H), 6.93 (s, 1H), 5.26 (d, J = 1.6 Hz, 1H), 5.06 (d, J = 1.5 Hz, 1H), 3.67 (t, J = 6.5 Hz, 2H), 2.73–2.55 (m, 2H), 2.33 (s, 6H), 2.13 (brs, 1H, OH), 1.81–1.65 (m, 2H).

^{13}C NMR (101 MHz, $CDCl_3$) δ 148.3, 141.2, 137.8, 129.2, 124.1, 112.3, 62.6, 31.8, 31.3, 21.5.

IR (neat) cm^{-1} υ: 2960 (w), 1624 (w), 1513 (w), 1461 (w), 1295 (w), 1055 (m), 1017 (w), 893 (m), 838 (s), 674 (m).

HRMS (APPI) m/z: $[M]^+$ Calcd for $C_{13}H_{18}O^+$ 190.1352; Found 190.1356.

Synthesis of 4-(5-hydroxypent-1-en-2-yl)benzonitrile (4m)

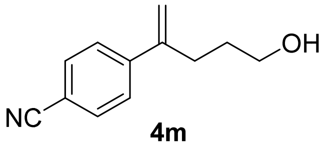

4m

Prepared according to general procedure B. Colorless oil.

^{1}H NMR (400 MHz, $CDCl_3$) δ 7.57 (d, J = 8.0 Hz, 2H), 7.48 (d, J = 8.0 Hz, 2H), 5.37 (s, 1H), 5.22 (s, 1H), 3.63 (t, J = 6.4 Hz, 2H), 2.57 (t, J = 7.7 Hz, 2H), 2.15 (brs, 1H), 1.68 (p, J = 6.9 Hz, 2H).

^{13}C NMR (101 MHz, $CDCl_3$) δ 146.5, 145.7, 132.2, 126.8, 118.9, 115.4, 110.8, 62.0, 31.1, 31.0.

IR (neat) cm^{-1} υ: 3344 (s), 2916 (s), 2203 (m), 1802 (w), 1585 (w), 1549 (m), 1484 (m), 1433 (m), 1148 (w), 1081 (w), 1010 (m), 970 (w), 970 (w), 886 (w), 825 (w), 737 (w).

HRMS (ESI) m/z: $[M + H]^+$ Calcd for $C_{12}H_{14}NO^+$ 188.1070; Found 188.1067

Synthesis of 5-phenylhex-5-en-1-ol (4n)

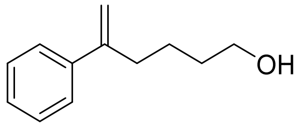

4n

Prepared according to general procedure **A**. Colorless oil. The physical and spectroscopic data were in accordance with those reported in the literature.[1]

^{1}H NMR (400 MHz, $CHCl_3$) δ 7.32–7.26 (m, 1H), 7.24–7.18 (m, 2H), 7.18–7.12 (m, 2H), 5.17 (s, 1H), 4.96 (s, 1H), 3.48 (t, J = 6.4 Hz, 2H), 2.59–2.36 (m, 2H), 1.83 (brs, 1H, OH), 1.67–1.31 (m, 4H).

Synthesis and characterization data of carboxylic acids 6

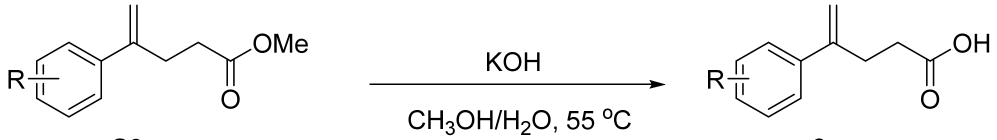

S3 **6**

General procedure **C:**

To a solution of ester **S3** (3.5 mmol, 1.0 equiv) in water (6 mL) and methanol (3 mL) was added KOH (0.39 g, 7.0 mmol, 2 equiv). The reaction mixture was stirred at 55 °C for 6 h, then cooled to room temperature. Brine (50 mL) was added and the mixture was acidified to pH = 3 with 1 N HCl. The mixture was extracted with Et_2O. The combined organic extracts were washed with brine, dried over Na_2SO_4. The solvent was evaporated under reduced pressure to give **6** as white solid, which can be used without further purification.

Synthesis of 4-(4-chlorophenyl)pent-4-enoic acid (6a)

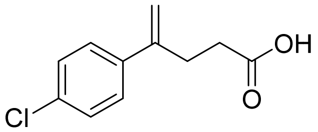

6a

Prepared according to general procedure **C**. White solid. The physical and spectroscopic data were in accordance with those reported in the literature.[3]

^{1}H NMR (400 MHz, $CHCl_3$) δ 8.74 (s, 1H), 7.35–7.27 (m, 4H), 5.30 (s, 1H), 5.10 (s, 1H), 2.79 (t, J = 8.0 Hz, 2H), 2.49 (t, J = 8.0 Hz, 2H).

Synthesis of 4-(p-tolyl)pent-4-enoic acid (6b)

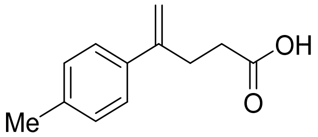

6b

Prepared according to general procedure **C**. White solid. The physical and spectroscopic data were in accordance with those reported in the literature.[3]

^{1}H NMR (400 MHz, $CHCl_3$) δ 7.30 (d, J = 8.2 Hz, 2H), 7.15 (d, J = 8.0 Hz, 2H), 5.30 (s, 1H), 5.06 (s, 1H), 2.89–2.79 (m, 2H), 2.64–2.48 (m, 2H), 2.35 (s, 3H).

Synthesis of 4-(4-methoxyphenyl)pent-4-enoic acid (6c)

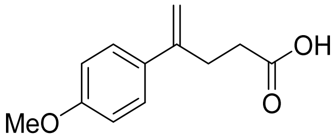

6c

Prepared according to general procedure **C**. White solid. The physical and spectroscopic data were in accordance with those reported in the literature.[3]

^{1}H NMR (400 MHz, $CHCl_3$) δ 11.4 (brs, 1H, COOH), 7.35 (d, J = 8.7 Hz, 2H), 6.87 (d, J = 8.8 Hz, 2H), 5.25 (s, 1H), 5.02 (s, 1H), 3.81 (s, 3H), 2.82 (t, J = 8.1 Hz, 2H), 2.53 (t, J = 8.1 Hz, 2H).

Synthesis of 4-(4-fluorophenyl)pent-4-enoic acid (6d)

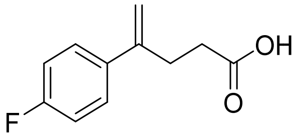

6d

Prepared according to general procedure **C**. White solid. The physical and spectroscopic data were in accordance with those reported in the literature.[3]

^{1}H NMR (400 MHz, $CHCl_3$) δ 11.40 (brs, 1H, COOH), 7.44–7.30 (m, 2H), 7.05–7.00 (m, 2H), 5.27 (s, 1H), 5.09 (s, 1H), 2.82 (t, J = 8.0 Hz, 2H), 2.52 (t, J = 8.0 Hz, 2H).

Synthesis of 4-phenylpent-4-enoic acid (6e)

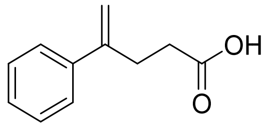

6e

Prepared according to general procedure **C**. White solid. The physical and spectroscopic data were in accordance with those reported in the literature.[3]

^{1}H NMR (400 MHz, $CHCl_3$) δ 7.44–7.38 (m, 2H), 7.36–7.32 (m, 2H), 7.31–7.27 (m, 1H), 5.33 (d, J = 0.9 Hz, 1H), 5.11 (d, J = 1.3 Hz, 1H), 2.91–2.77 (m, 2H), 2.69–2.37 (m, 2H).

Synthesis of 4-(4-bromophenyl)pent-4-enoic acid (6f)

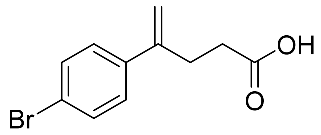

6f

Prepared according to general procedure **C**. White solid. The physical and spectroscopic data were in accordance with those reported in the literature.[4]

^{1}H NMR (400 MHz, $CHCl_3$) δ 7.46 (d, J = 8.4 Hz, 2H), 7.27 (d, J = 8.4 Hz, 2H), 5.32 (s, 1H), 5.13 (d, J = 1.2 Hz, 1H), 2.86–2.77 (m, 2H), 2.60–2.48 (m, 2H).

Synthesis of 4-([1,1'-biphenyl]-4-yl)pent-4-enoic acid (6g)

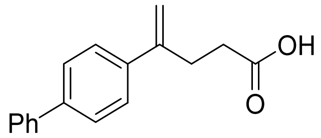

6g

Prepared according to general procedure **C**. White solid. The physical and spectroscopic data were in accordance with those reported in the literature.[3]

^{1}H NMR (400 MHz, $CHCl_3$) δ 7.67–7.55 (m, 4H), 7.54–7.42 (m, 4H), 7.40–7.31 (m, 1H), 5.40 (s, 1H), 5.14 (s, 1H), 2.89 (t, J = 7.6 Hz, 2H), 2.58 (t, J = 7.6 Hz, 2H).

Synthesis of 4-(4-isopropylphenyl)pent-4-enoic acid (6h)

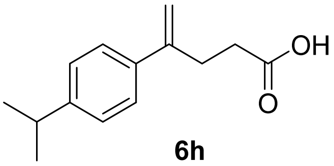

6h

Prepared according to general procedure **C**. White solid.

MP: 52−53 °C

^{1}H NMR (400 MHz, $CHCl_3$) δ 11.60 (brs, 1H, COOH), 7.35 (d, J = 8.2 Hz, 2H), 7.20 (d, J = 8.2 Hz, 2H), 5.32 (s, 1H), 5.07 (s, 1H), 2.96-2.86 (m, 1H), 2.84 (t, J = 7.8 Hz, 2H), 2.55 (t, J = 7.8 Hz, 2H), 1.26 (d, J = 6.9 Hz, 6H).

^{13}C NMR (101 MHz, $CDCl_3$) δ 179.9, 148.6, 146.4, 137.9, 126.6, 126.1, 112.3, 33.9, 33.2, 30.2, 24.1

IR (neat) cm^{-1} υ: 2959 (m), 1694 (s), 1625 (w), 1510 (w), 1441 (w), 1414 (w), 1333 (m), 1267 (w), 1222 (m), 1014 (w), 900 (m), 840 (s), 712 (w).

HRMS (ESI) m/z: [M + H]$^+$ Calcd for $C_{14}H_{19}O_2^+$ 219.1380; Found 219.1381.

Synthesis of 4-(4-cyanophenyl)pent-4-enoic acid (6i)

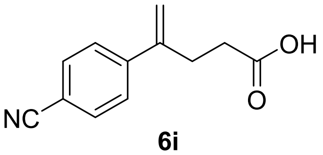

6i

Prepared according to general procedure **C**. White solid. The physical and spectroscopic data were in accordance with those reported in the literature.[5]

^{1}H NMR (400 MHz, $CDCl_3$) δ 10.63 (s, 1H), 7.62 (d, J = 8.2 Hz, 2H), 7.49 (d, J = 8.2 Hz, 2H), 5.42 (s, 1H), 5.25 (s, 1H), 2.83 (t, J = 7.7 Hz, 2H), 2.53 (t, J = 7.7 Hz, 2H).

Synthesis and characterization data of sulfonamides 8

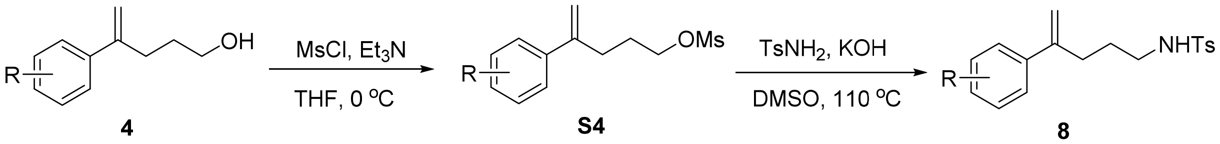

General procedure **D:**

To a solution of **4** (2.0 mmol, 1.0 euqiv) and Et_3N (0.43 mL, 3.0 mmol, 1.5 euqiv) in THF (30 mL) was added MsCl (0.15 mL, 2.0 mmol, 1.0 equiv) at 0 ºC. The reaction mixture was stirred at 0 ºC for 30 minutes, then filtered, and the filter cake was washed with THF three times. The filtrate was evaporated under reduced pressure. The residue **S4** was used for the next reaction without further purification.

To a solution of **S4** obtained above in DMSO (8 mL) was added $TsNH_2$ (0.51 g, 3.0 mmol, 1.5 equiv). The reaction mixture was stirred at 110 ºC for 4 h, then cooled to room temperature. Water was added. The mixture was extracted with Et_2O (3 × 15 mL). The combined organic extracts were washed with brine, dried over Na_2SO_4. The volatiles were evaporated under reduced pressure. The residue was purified by flash chromatography (SiO_2, eluent: PE/EtOAc = 5/1) to afford the desired **8**.

Synthesis of 4-methyl-N-(4-phenylpent-4-en-1-yl)benzenesulfonamide (8a)

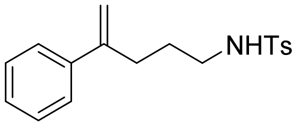

8a

Prepared according to general procedure **D**. Pale yellow oil. The physical and spectroscopic data were in accordance with those reported in the literature.[6]

^{1}H NMR (400 MHz, $CHCl_3$) δ 7.63 (d, J = 8.1 Hz, 2H), 7.23 (d, J = 1.2 Hz, 2H), 7.22–7.14 (m, 5H), 5.17 (d, J = 1.4 Hz, 1H), 4.93 (d, J = 1.4 Hz, 1H), 4.36 (brs, 1H) 2.88 (q, J = 6.6 Hz, 2H), 2.50–2.37 (m, 2H), 2.35 (s, 3H), 1.56–1.48 (m, 2H).

Synthesis of 4-methyl-N-(4-(p-tolyl)pent-4-en-1-yl)benzenesulfonamide (8b)

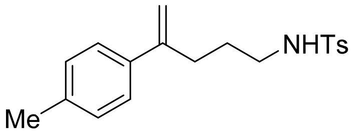

8b

Prepared according to general procedure **D**. Pale yellow solid.

MP: 69–70 ºC

^{1}H NMR (400 MHz, $CHCl_3$) δ 7.63 (d, J = 8.0 Hz, 2H), 7.18 (d, J = 8.0 Hz, 2H), 7.13 (d, J = 8.0 Hz, 2H), 7.02 (d, J = 8.0 Hz, 2H), 5.13 (s, 1H), 4.87 (s, 1H), 2.85 (q, J = 6.7 Hz, 2H), 2.39 (t, J = 7.5 Hz, 2H), 2.33 (s, 3H), 2.25 (s, 3H), 1.50 (p, J = 7.2 Hz, 2H).

^{13}C NMR (101 MHz, $CDCl_3$) δ 147.0, 143.4, 137.7, 137.4, 137.0, 129.8, 129.1, 127.2, 126.0, 112.4, 42.8, 32.3, 28.0, 21.6, 21.2.

IR (neat) cm^{-1} υ: 3255 (w), 1631 (w), 1512 (w), 1427 (w), 1323 (s), 1290 (w), 1158 (s), 1081 (m), 900 (m), 817 (s), 736 (w).

HRMS (ESI) m/z: $[M + H]^+$ Calcd for $C_{19}H_{24}NO_2S^+$ 330.1522; Found 330.1525.

Synthesis of N-(4-(4-methoxyphenyl)pent-4-en-1-yl)-4-methylbenzenesulfonamide (8c)

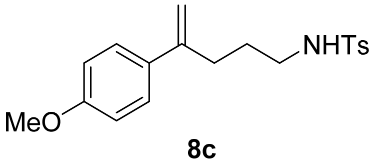

8c

Prepared according to general procedure **D**. Pale yellow oil.

^{1}H NMR (400 MHz, $CHCl_3$) δ 7.74 (d, J = 8.2 Hz, 2H), 7.30–7.27 (m, 4H), 6.85 (d, J = 9.0 Hz, 2H), 5.19 (s, 1H), 4.93 (s, 1H), 4.82 (t, J = 6.3 Hz, 1H), 3.82 (s, 3H), 2.96 (q, J = 6.8 Hz, 1H), 2.48 (t, J = 7.5 Hz, 1H), 1.66–1.58 (m, 2H).

^{13}C NMR (101 MHz, $CDCl_3$) δ 159.2, 146.4, 143.4, 137.0, 133.0, 129.8, 127.21, 127.15, 113.8, 111.6, 55.4, 42.8, 32.4, 28.0, 21.6.

IR (neat) cm^{-1} υ: 2921 (w), 1599 (w), 1492 (w), 1423 (w), 1323 (m), 1157 (s), 1094 (m), 899 (m), 814 (m), 794 (m), 727 (m).

HRMS (ESI) m/z: $[M + H]^+$ Calcd for $C_{19}H_{24}NO_3S^+$ 346.1471; Found 346.1474.

Synthesis of N-(4-(4-fluorophenyl)pent-4-en-1-yl)-4-methylbenzenesulfonamide (8d)

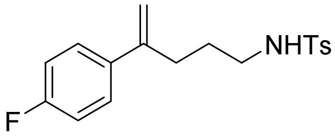

8d

Prepared according to general procedure **D**. Pale yellow solid.

MP: 59–60 °C

^{1}H NMR (400 MHz, $CHCl_3$) δ 7.74 (d, J = 8.3 Hz, 2H), 7.47–7.21 (m, 4H), 7.00 (t, J = 8.7 Hz, 2H), 5.21 (d, J = 1.2 Hz, 1H), 5.00 (d, J = 1.4 Hz, 1H), 4.72 (t, J = 6.3 Hz, 1H), 2.96 (q, J = 6.6 Hz, 2H), 2.53–2.46 (m, 2H), 2.44 (s, 3H), 1.74–1.50 (m, 2H).

^{13}C NMR (101 MHz, $CHCl_3$) δ 162.3 (d, J = 246.4 Hz), 146.1, 143.4, 136.9, 136.6 (d, J = 3.3 Hz), 129.7, 127.6 (d, J = 7.8 Hz), 127.0, 115.2 (d, J = 21.3 Hz), 113.0 (d, J = 1.2 Hz), 42.8, 32.5, 28.0, 21.6.

IR (neat) cm^{-1} υ: 3276 (m), 1599 (w), 1509 (s), 1431 (m), 1325 (s), 1230 (m), 1159 (s), 1062 (m), 909 (w), 897 (w), 845 (m), 845 (m), 816 (m), 677 (m).

HRMS (ESI) m/z: [M + H]$^+$ Calcd for $C_{18}H_{21}FNO_2S^+$ 334.1272; Found 334.1273.

Synthesis of N-(4-(4-chlorophenyl)pent-4-en-1-yl)-4-methylbenzenesulfonamide (8e)

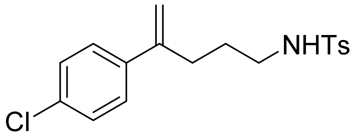

8e

Prepared according to general procedure **D**. Pale yellow solid.

MP: 73–74 ºC

^{1}H NMR (400 MHz, $CHCl_3$) δ 7.73 (d, J = 8.3 Hz, 2H), 7.37–7.24 (m, 6H), 5.25 (d, J = 1.1 Hz, 1H), 5.03 (d, J = 1.3 Hz, 1H), 2.96 (q, J = 6.6 Hz, 2H), 2.52–2.45 (m, 2H), 2.44 (s, 3H), 1.67–1.49 (m, 2H).

^{13}C NMR (101 MHz, $CDCl_3$) δ 146.1, 143.5, 139.1, 137.0, 133.4, 129.8, 128.6, 127.5, 127.2, 113.7, 42.8, 32.2, 28.0, 21.6.

IR (neat) cm^{-1} υ: 3266 (m), 2868 (w), 1739 (w), 1597 (w), 1491 (m), 1432 (w), 1318 (s), 1299 (m), 1155 (s), 1091 (m), 1060 (m), 1010 (m), 911 (m), 844 (m), 816 (m), 728 (s), 719 (s).

HRMS (ESI) m/z: [M + H]$^+$ Calcd for $C_{18}H_{21}ClNO_2S^+$ 350.0976; Found 350.0979.

Synthesis of N-(4-(4-bromophenyl)pent-4-en-1-yl)-4-methylbenzenesulfonamide (8f)

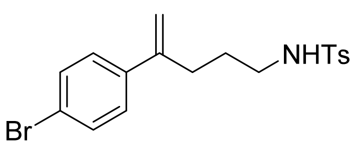

8f

Prepared according to general procedure **D**. Pale yellow solid.

MP: 76–77 ºC

^{1}H NMR (400 MHz, $CHCl_3$) δ 7.62 (d, J = 8.1 Hz, 2H), 7.35 (d, J = 8.1 Hz, 2H), 7.21 (d, J = 8.0 Hz, 2H), 7.11 (d, J = 8.1 Hz, 2H), 5.17 (s, 1H), 4.96 (s, 1H), 4.32 (t, J = 6.2 Hz, 1H), 2.88 (q, J = 6.8 Hz, 2H), 2.40 (t, J = 7.6 Hz, 2H), 2.36 (s, 3H), 1.50 (p, J = 7.2 Hz, 2H).

^{13}C NMR (101 MHz, $CDCl_3$) δ 146.1, 143.5, 139.6, 137.0, 131.6, 129.8, 127.8, 127.2, 121.6, 113.8, 42.7, 32.2, 28.0, 21.7.

IR (neat) cm^{-1} υ: 3250 (m), 2161 (w), 1597 (w), 1489 (w), 1435 (w), 1317 (s), 1154 (s), 1060 (m), 1005 (w), 906 (w), 839 (w), 816 (w), 719 (m).

HRMS (ESI) m/z: $[M + H]^+$ Calcd for $C_{18}H_{21}BrNO_2S^+$ 394.0471; Found 394.0474.

Synthesis of N-(4-([1,1'-biphenyl]-4-yl)pent-4-en-1-yl)-4-methylbenzenesulfonamide (8g)

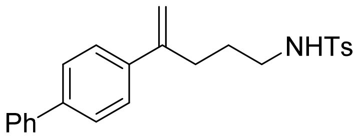

8g

Prepared according to general procedure **D**. Pale yellow solid.

MP: 94–96 ºC

^{1}H NMR (400 MHz, $CHCl_3$) δ 7.76 (d, J = 8.4 Hz, 2H), 7.63 (d, J = 7.2 Hz, 2H), 7.57 (d, J = 8.2 Hz, 2H), 7.51–7.35 (m, 5H), 7.32–7.27 (m, 2H), 5.35 (s, 1H), 5.07 (s, 1H), 4.75 (t, J = 6.5 Hz, 1H), 3.01 (q, J = 6.7 Hz, 2H), 2.57 (t, J = 7.5 Hz, 2H), 2.42 (s, 3H), 1.67 (p, J = 7.1 Hz, 2H).

^{13}C NMR (101 MHz, $CDCl_3$) δ 146.7, 143.4, 140.7, 140.4, 139.5, 137.1, 129.8, 128.9, 127.4, 127.18, 127.15, 127.1, 126.6, 113.2, 42.8, 32.3, 28.1, 21.6.

IR (neat) cm^{-1} υ: 3299 (m), 1597 (w), 1489 (w), 1422 (w), 1324 (m), 1156 (s), 1093 (m), 1075 (m), 896 (m), 842 (m), 817 (m), 772 (m), 737 (s).

HRMS (ESI) m/z: $[M + H]^+$ Calcd for $C_{24}H_{26}NO_2S^+$ 392.1679; Found 392.1684.

Synthesis of N-(4-(4-isopropylphenyl)pent-4-en-1-yl)-4-methylbenzenesulfonamide (8h)

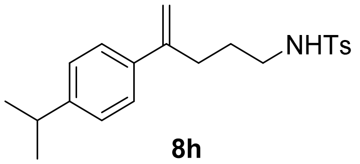

8h

Prepared according to general procedure **D**. Pale yellow oil.

^{1}H NMR (400 MHz, $CHCl_3$) δ 7.75 (d, J = 8.1 Hz, 2H), 7.36–7.25 (m, 4H), 7.19 (d, J = 8.1 Hz, 2H), 5.25 (d, J = 1.5 Hz, 1H), 4.98 (d, J = 1.6 Hz, 1H), 4.70 (t, J = 6.2 Hz, 1H), 3.02–2.88 (m, 3H), 2.51 (t, J = 7.5 Hz, 2H), 2.45 (s, 3H), 1.69–1.59 (m, 2H), 1.28 (d, J = 6.9 Hz, 6H);

^{13}C NMR (101 MHz, $CDCl_3$) δ 148.4, 146.9, 143.4, 138.0, 137.1, 129.8, 127.2, 126.5, 126.0, 112.4, 42.9, 33.9, 32.3, 28.1, 24.1, 21.6.

IR (neat) cm^{-1} υ: 3283 (m), 2162 (m), 1735 (w), 1459 (w), 1421 (w), 1325 (m), 1157 (s), 1094 (m), 901 (w), 840 (m), 813 (m), 719 (m), 662 (s).

HRMS (ESI) m/z: [M + H]$^+$ Calcd for $C_{21}H_{28}NO_2S^+$ 358.1835; Found 358.1837.

Synthesis of 4-methyl-N-(4-(m-tolyl)pent-4-en-1-yl)benzenesulfonamide (8i)

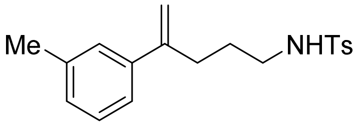

8i

Prepared according to general procedure **D**. Pale yellow oil.

^{1}H NMR (400 MHz, CHCl$_3$) δ 7.72 (d, J = 8.2 Hz, 1H), 7.33–7.26 (m, 3H), 7.26–7.07 (m, 4H), 5.25 (d, J = 1.4 Hz, 1H), 5.01 (d, J = 1.6 Hz, 1H), 4.30 (brs, 1H), 2.98 (q, J = 6.6 Hz, 2H), 2.51 (t, J = 7.5 Hz, 2H), 2.45 (s, 3H), 2.37 (s, 3H), 1.65–1.57 (m, 2H).

^{13}C NMR (101 MHz, CDCl$_3$) δ 147.2, 143.2, 140.6, 137.7, 136.9, 129.6, 128.2, 127.0, 126.7, 123.1, 112.7, 42.6, 32.1, 27.8, 21.4.

IR (neat) cm^{-1} υ: 3267 (w), 1741 (w), 1599 (w), 1448 (w), 1427 (w), 1322 (m), 1304 (m), 1151 (s), 1096 (m), 1073 (m), 813 (s), 799 (m), 694 (m).

HRMS (ESI) m/z: [M + H]$^+$ Calcd for $C_{19}H_{24}NO_2S^+$ 330.1522; Found 330.1524.

Synthesis of N-(4-(3-chlorophenyl)pent-4-en-1-yl)-4-methylbenzenesulfonamide (8j)

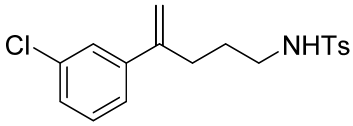

8j

Prepared according to general procedure **D**. Pale yellow oil.

^{1}H NMR (400 MHz, CHCl$_3$) δ 7.75 (d, J = 8.1 Hz, 2H), 7.31–7.29 (m, 3H), 7.26–7.17 (m, 3H), 5.26 (s, 1H), 5.12 (t, J = 6.3 Hz, 1H), 5.05 (s, 1H), 2.95 (q, J = 6.8 Hz, 2H), 2.47 (t, J = 7.6 Hz, 2H), 2.43 (s, 3H), 1.59 (p, J = 7.2 Hz, 2H).

^{13}C NMR (101 MHz, CDCl$_3$) δ 146.0, 143.4, 142.6, 136.9, 134.3, 129.8, 129.7, 127.5, 127.1, 126.2, 124.3, 114.2, 42.6, 32.0, 27.8, 21.6.

IR (neat) cm^{-1} υ: 3276 (m), 1594 (w), 1562 (w), 1476 (w), 1414 (w), 1322 (m), 1156 (s), 1094 (m), 901 (w), 815 (m), 729 (m), 662 (s).

HRMS (ESI) m/z: $[M + H]^+$ Calcd for $C_{18}H_{21}ClNO_2S^+$ 350.0976; Found 350.0980.

Synthesis of N-(4-(4-cyanophenyl)pent-4-en-1-yl)-4-methylbenzenesulfonamide (8k)

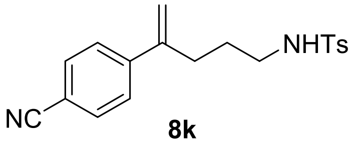

8k

Prepared according to general procedure **D**. Pale yellow oil.

^{1}H NMR (400 MHz, $CDCl_3$) δ 7.74 (d, *J* = 8.4 Hz, 2H), 7.58 (d, *J* = 8.3 Hz, 2H), 7.44 – 7.41 (m, 2H), 7.32 – 7.27 (m, 2H), 5.36 (s, 1H), 5.17 (s, 1H), 5.00 (t, *J* = 6.3 Hz, 1H), 2.96 (q, *J* = 6.7 Hz, 2H), 2.52 (t, *J* = 7.7 Hz, 2H), 2.44 (s, 3H), 1.61 (p, *J* = 7.0 Hz, 2H).

^{13}C NMR (101 MHz, $CDCl_3$) δ 145.8, 145.4, 143.6, 136.9, 132.3, 129.8, 127.1, 126.8, 118.9, 116.0, 111.1, 42.6, 31.8, 28.0, 21.6.

HRMS (ESI) m/z: $[M + Na]^+$ Calcd for $C_{19}H_{20}N_2NaO_2S^+$ 363.1138; Found 363.1135.

IR (neat) cm^{-1} υ: 3280 (m), 1732 (w), 1462 (w), 1420 (w), 1323 (m), 1158 (s), 1092 (m), 904 (w), 843 (m), 813 (m), 719 (m), 662 (s).

General procedure for the copper-catalyzed three-component 1,2-alkoxy methylation of alkenes

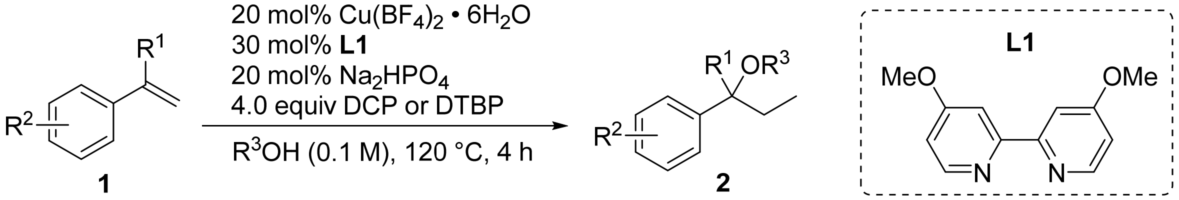

A screw cap tube was charged with $Cu(BF_4)_2•6H_2O$ (13.8 mg, 0.0400 mmol), 4,4'-dimethoxy-2,2'-bipyridyl **L1** (13.0 mg, 0.0601 mmol), Na_2HPO_4 (5.7 mg, 0.0402 mmol) and R^3OH (2.0 mL). The mixture was stirred at room temperature for 30 minutes, then substrate **1** (0.2 mmol, 1.0 equiv) and DCP (216.2 mg, 0.800 mmol) or DTBP (0.15 mL, 4.0 equiv) were added to the above mixture. After being stirred for 4 hours at 120 °C under N_2 atmosphere, the reaction mixture was quenched with water, extracted with EtOAc. The organic extracts were washed with brine, dried over Na_2SO_4. The solvent was removed under reduced pressure. The residue was purified by flash chromatography to give **2.**

Characterization of compounds 2

(2-methoxybutan-2-yl)benzene (2a)

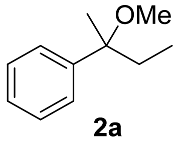

2a

According to the general procedure for the copper-catalyzed three-component 1,2-alkoxy methylation of alkenes. The crude product was purified by flash column chromatography on silica gel (PE/CH_2Cl_2 = 6/1, PE/EtOAc = 40/1, 8/1) to afford **2a** (31.0 mg, 96% yield) as a colorless oil.

^{1}H NMR (400 MHz, $CDCl_3$) δ 7.34–7.22 (m, 4H), 7.21–7.09 (m, 1H), 3.01 (s, 3H), 1.72 (q, J = 7.4 Hz, 2H), 1.44 (s, 3H), 0.70 (t, J = 7.4 Hz, 3H).

^{13}C NMR (101 MHz, $CDCl_3$) δ 145.2, 128.2, 126.8, 126.4, 79.5, 50.4, 35.2, 22.6, 8.5.

IR (neat) cm^{-1} υ: 3060 (w), 2973 (w), 2935 (w), 2880 (w), 2824 (w), 1447 (w), 1372 (w), 1169 (w), 1073 (s), 760 (m), 700 (s).

HRMS (ESI) calcd for $C_{11}H_{17}O^+$ $[M+H]^+$ 165.1274; found 165.1270.

1-(2-methoxybutan-2-yl)-4-methylbenzene (2b)

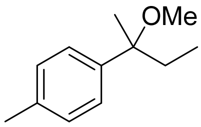

2b

According to the general procedure for the copper-catalyzed three-component 1,2-alkoxy methylation of alkenes. The crude product was purified by flash column chromatography on silica gel (PE/CH_2Cl_2 = 10/1, PE/EtOAc = 50/1) to afford **2b** (32.5 mg, 91% yield) as a colorless oil.

^{1}H NMR (400 MHz, $CDCl_3$) δ 7.18 (d, J = 7.6 Hz, 2H), 7.08 (d, J = 7.6 Hz, 2H), 2.99 (s, 3H), 2.27 (s, 3H), 1.71 (q, J = 7.4 Hz, 2H), 1.41 (s, 3H), 0.70 (t, J = 7.4 Hz, 3H).

^{13}C NMR (101 MHz, $CDCl_3$) δ 142.1, 136.3, 128.9, 126.4, 79.4, 50.4, 35.1, 22.6, 21.1, 8.5.

IR (neat) cm^{-1} υ: 2972 (w), 2933 (w), 2880 (w), 2824 (w), 1510 (w), 1462 (w), 1370 (w), 1169 (w), 1078 (s), 1020 (w), 857 (w), 814 (s).

HRMS (ESI) calcd for $C_{12}H_{19}O^+$ $[M+H]^+$ 179.1430; found 179.1423.

1-methoxy-4-(2-methoxybutan-2-yl)benzene (2c)

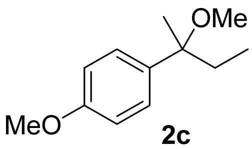

2c

According to the general procedure for the copper-catalyzed three-component 1,2-alkoxy methylation of alkenes. The crude product was purified by flash column chromatography on silica gel (PE/EtOAc = 50/1) and then by PTLC (PE/EtOAc = 20/1, developed twice) to afford **2c** (30.0 mg, 77% yield) as a colorless oil.

^{1}H NMR (400 MHz, $CDCl_3$) δ 7.24–7.17 (m, 2H), 6.84–6.77 (m, 2H), 3.74 (s, 3H), 2.98 (s, 3H), 1.70 (q, J = 7.5 Hz, 2H), 1.41 (s, 3H), 0.69 (t, J = 7.5 Hz, 3H).

^{13}C NMR (101 MHz, $CDCl_3$) δ158.4, 137.1, 127.6, 113.4, 79.2, 55.3, 50.3, 35.2, 22.5, 8.6.

IR (neat) cm^{-1} υ: 2971 (w), 2935 (w), 2834 (w), 1611 (w), 1509 (s), 1463 (w), 1371 (w), 1301 (m), 1249 (s), 1178 (s), 1077 (s), 1034 (s), 828 (s).

HRMS (ESI) calcd for $C_{12}H_{19}O_2^+$ $[M+H]^+$ 195.1380; found 195.1385.

1-fluoro-4-(2-methoxybutan-2-yl)benzene (2d)

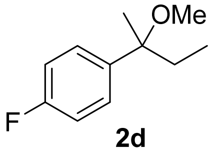

2d

According to the general procedure for the copper-catalyzed three-component 1,2-alkoxy methylation of alkenes. The crude product was purified by flash column chromatography on silica gel (PE/CH_2Cl_2 = 10/1, PE/EtOAc = 50/1) and then by PTLC (PE/EtOAc = 20/1, developed twice) to afford **2d** (29.8 mg, 80% yield) as a colorless oil.

^{1}H NMR (400 MHz, $CDCl_3$) δ 7.28–7.22 (m, 2H), 6.99–6.90 (m, 2H), 2.99 (s, 3H), 1.70 (q, J = 7.4 Hz, 2H), 1.42 (s, 3H), 0.68 (t, J = 7.4 Hz, 3H).

^{13}C NMR (100 MHz, $CDCl_3$) δ 161.8 (d, J = 244.7 Hz), 140.9 (d, J = 3.0 Hz), 128.1 (d, J = 7.9 Hz), 114.9 (d, J = 21.0 Hz), 79.2, 50.3, 35.2, 22.6, 8.4.

IR (neat) cm^{-1} υ: 2975 (w), 2937 (w), 2881 (w), 2826 (w), 1603 (w), 1506 (s), 1463 (w), 1372 (w), 1300 (w), 1225 (s), 1161 (m), 1077 (s), 832 (s), 814 (m).

HRMS (ESI) calcd for $C_{11}H_{16}FO^+$ $[M+H]^+$ 183.1180; found 183.1175.

4-(2-methoxybutan-2-yl)-1,1'-biphenyl (2e)

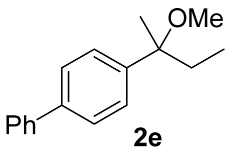

2e

According to the general procedure for the copper-catalyzed three-component 1,2-alkoxy methylation of alkenes. The crude product was purified by flash column chromatography on silica gel (PE/CH_2Cl_2 = 10/1, PE/EtOAc = 50/1) and then by PTLC (PE/DCM = 6/1) to afford **2e** (38.5 mg, 80% yield) as a colorless oil.

^{1}H NMR (400 MHz, $CDCl_3$) δ 7.66–7.53 (m, 4H), 7.48–7.41 (m, 4H), 7.38–7.31 (m, 1H), 3.13 (s, 3H), 1.84 (q, J = 7.4 Hz, 2H), 1.55 (s, 3H), 0.82 (t, J = 7.4 Hz, 3H).

^{13}C NMR (101 MHz, $CDCl_3$) δ 144.3, 141.0, 139.6, 128.9, 127.3, 127.2, 126.9, 79.4, 76.8, 50.5, 35.1, 29.9, 22.7, 8.5.

IR (neat) cm^{-1} υ: 2980 (w), 2931 (w), 1603 (w), 1506 (s), 1495 (w), 1440 (w), 1225 (s), 1152 (m), 1075 (s), 762 (m), 624 (s);

HRMS (ESI) calcd for $C_{17}H_{21}O^+$ $[M+H]^+$ 241.1587; found 241.1590.

1-(2-methoxybutan-2-yl)-3-methylbenzene (2e)

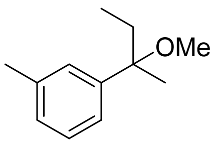

2f

According to the general procedure for the copper-catalyzed three-component 1,2-alkoxy methylation of alkenes. The crude product was purified by flash column chromatography on silica gel (PE/CH_2Cl_2 = 3/1) to afford **2e** (27.6 mg, 78% yield) as a colorless oil.

^{1}H NMR (400 MHz, $CDCl_3$) δ 7.31 – 7.23 (m, 1H), 7.23 – 7.15 (m, 2H), 7.09 (d, J = 7.4 Hz, 1H), 3.11 (s, 3H), 2.40 (s, 3H), 1.82 (q, J = 7.4 Hz, 2H), 1.53 (s, 3H), 0.81 (t, J = 7.4 Hz, 3H).

^{13}C NMR (101 MHz, $CDCl_3$) δ 145.2, 137.6, 128.0, 127.5, 127.1, 123.5, 79.4, 50.4, 35.1, 22.6, 21.8, 8.5.

IR (neat) cm^{-1} υ: 2974 (w), 2934 (w), 2024 (w), 1459 (w), 1373 (w), 1299 (w), 1168 (m), 1082 (s), 1039 (w), 872 (w), 820 (w), 785 (s), 708 (s).

HRMS (ESI) m/z: $[M–OCH_3]^+$ Calcd for $C_{11}H_{15}^+$ 147.1168; Found 147.1169.

1-chloro-3-(2-methoxybutan-2-yl)benzene (2g)

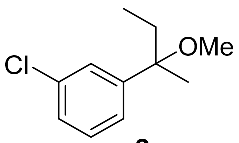

2g

According to the general procedure for the copper-catalyzed three-component 1,2-alkoxy methylation of alkenes. The crude product was purified by flash column chromatography on silica gel (PE/CH_2Cl_2 = 3/1) to afford **2g** (32.5 mg, 83% yield) as a colorless oil.

^{1}H NMR (400 MHz, $CDCl_3$) δ 7.38 (s, 1H), 7.32–7.21 (m, 3H), 3.11 (s, 3H), 1.79 (q, J = 7.4 Hz, 2H), 1.51 (s, 3H), 0.79 (t, J = 7.5 Hz, 3H).

^{13}C NMR (101 MHz, $CDCl_3$) δ 147.7, 134.3, 129.5, 127.0, 126.7, 124.6, 79.3, 50.5, 35.0, 22.6, 8.4.

IR (neat) cm^{-1} υ: 2936 (w), 1734 (w), 1570 (w), 1466 (w), 1410 (w), 1372 (w), 1296 (w), 1229 (w), 1170 (w), 1076 (s), 1034 (w), 869 (m), 786 (s), 752 (m).

HRMS (ESI) m/z: $[M–OCH_3]^+$ Calcd for $C_{10}H_{12}Cl^+$ 167.0622; Found 167.0625.

4-(2-methoxybutan-2-yl)-1,2-dimethylbenzene (2h)

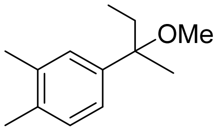

2h

According to the general procedure for the copper-catalyzed three-component 1,2-alkoxy methylation of alkenes. The crude product was purified by flash column chromatography on silica gel (PE/CH_2Cl_2 = 3/1) to afford **2h** (32.3 mg, 85% yield) as a colorless oil.

^{1}H NMR (400 MHz, $CDCl_3$) δ 7.12–7.08 (m, 3H), 3.09 (s, 3H), 2.29 (s, 3H), 2.27 (s, 3H), 1.80 (q, J = 7.4 Hz, 2H), 1.50 (s, 3H), 0.80 (t, J = 7.4 Hz, 3H).

^{13}C NMR (101 MHz, $CDCl_3$) δ 142.6, 136.2, 134.9, 129.4, 127.7, 123.9, 79.3, 50.4, 35.0, 22.6, 20.2, 19.5, 8.5.

IR (neat) cm^{-1} υ: 2970 (w), 1743 (w), 1504 (w), 1452 (w), 1371 (w), 1168 (w), 1126 (w), 1080 (s), 997 (w), 821 (m), 723 (w).

HRMS (ESI) m/z: $[M + Na]^+$ Calcd for $C_{13}H_{20}NaO^+$ 215.1406; Found 215.1407.

1-(2-methoxybutan-2-yl)-2-methylbenzene (2i)

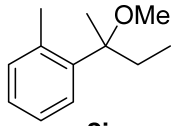

2i

According to the general procedure for the copper-catalyzed three-component 1,2-alkoxy methylation of alkenes. The crude product was purified by flash column chromatography on silica gel (PE/CH_2Cl_2 = 3/1) to afford **2i** (24.5 mg, 83% yield) as a colorless oil.

^{1}H NMR (400 MHz, $CDCl_3$) δ 7.24–7.18 (m, 1H), 7.17–7.12 (m, 3H), 3.04 (s, 3H), 2.54 (s, 3H), 2.02 – 1.82 (m, J = 7.2 Hz, 2H), 1.58 (s, 3H), 0.79 (t, J = 7.4 Hz, 3H).

^{13}C NMR (101 MHz, $CDCl_3$) δ 141.8, 137.1, 132.6, 128.2, 127.1, 125.5, 81.4, 50.2, 32.8, 23.2, 21.6, 8.8.

IR (neat) cm^{-1} υ: 2972 (m), 1738 (w), 1457 (w), 1371 (w), 1221 (w), 1147 (w), 1112 (w), 1083 (m), 865 (w), 758 (s), 728 (s), 668 (w).

HRMS (ESI) m/z: $[M-OCH_3]^+$ Calcd for $C_{11}H_{15}^+$ 147.1174; Found 147.1168.

(3-methoxypentan-3-yl)benzene (2j)

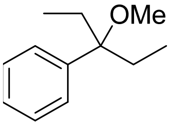

2j

According to the general procedure for the copper-catalyzed three-component 1,2-alkoxy methylation of alkenes. The crude product was purified by flash column chromatography on silica gel (PE/CH_2Cl_2 = 8/1, PE/EtOAc = 50/1) to afford **2j** (30.1 mg, 84% yield) as a colorless oil.

^{1}H NMR (400 MHz, $CDCl_3$) δ 7.33–7.21 (m, 4H), 7.20–7.11 (m, 1H), 2.99 (s, 3H), 1.85–1.70 (m, 4H), 0.64 (t, J = 7.4 Hz, 6H).

^{13}C NMR (101 MHz, $CDCl_3$) δ 144.4, 128.0, 126.8, 126.6, 81.6, 49.6, 28.3, 7.6.

IR (neat) cm^{-1} υ: 3060 (w), 3024 (w), 2969 (w), 2936 (w), 2879 (w), 2825 (w), 1447 (w), 1075 (m), 941 (w), 892 (w), 757 (s), 700 (s).

HRMS (ESI) calcd for $C_{12}H_{19}O^+$ $[M+H]^+$ 179.1430; found 179,1433.

(3-methoxy-2-methylpentan-3-yl)benzene (2k)

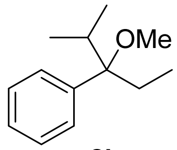

2k

According to the general procedure for the copper-catalyzed three-component 1,2-alkoxy methylation of alkenes. The crude product was purified by flash column chromatography on silica gel (PE/CH_2Cl_2 = 10/1, PE/EtOAc = 50/1) to afford **2k** (26.5 mg, 69% yield) as a colorless oil.

^{1}H NMR (400 MHz, $CDCl_3$) δ 7.28–7.07 (m, 5H), 3.07 (s, 3H), 2.16–1.98 (m, 2H), 1.94–1.80 (m, 1H), 0.83 (t, J = 7.0 Hz, 3H), 0.72–0.57 (m, 6H).

^{13}C NMR (101 MHz, $CDCl_3$) δ 140.7, 128.0 (2C), 127.2 (2C), 126.3, 83.7, 49.7, 33.3, 24.3, 18.0, 16.9, 7.1.

IR (neat) cm^{-1} υ: 2965 (w), 2938 (w), 2879 (w), 2826 (w), 1446 (w), 1383 (w), 1348 (w), 1144 (w), 1102 (m), 1074 (m), 1059 (m), 941 (w), 895 (w), 758 (s), 704 (s).

HRMS (ESI) calcd for $C_{13}H_{21}O^+$ $[M+H]^+$ 193.1587; found 193.1598.

(3-methoxypentane-1,3-diyl)dibenzene (2l)

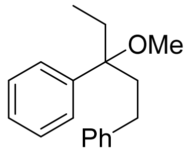

2l

According to the general procedure for the copper-catalyzed three-component 1,2-alkoxy methylation of alkenes. The crude product was purified by flash column chromatography on silica gel (PE/CH_2Cl_2 = 3/1) to afford **2l** (43.4 mg, 85% yield) as a colorless oil.

^{1}H NMR (400 MHz, $CDCl_3$) δ 7.49 (d, J = 7.8 Hz, 2H), 7.42 (t, J = 7.9 Hz, 2H), 7.32 (t, J = 7.1 Hz, 3H), 7.25–7.15 (m, 3H), 3.23 (s, 3H), 2.59–2.41 (m, 2H), 2.31–2.23 (m, 1H), 2.18–2.10 (m, 1H), 2.06–1.89 (m, 2H), 0.80 (t, J = 7.2 Hz, 3H).

^{13}C NMR (101 MHz, $CDCl_3$) δ 144.1, 142.8, 128.5, 128.4, 128.1, 126.7, 126.6, 125.8, 81.2, 49.5, 37.8, 29.7, 29.4, 7.7.

IR (neat) cm^{-1} υ: 2938 (w), 1602 (w), 1494 (w), 1452 (w), 1334 (w), 1190 (w), 1074 (m), 1032 (w), 910 (w), 887 (w), 758 (m), 700 (s).

HRMS (ESI) m/z: $[M + Na]^+$ Calcd for $C_{18}H_{22}NaO^+$ 277.1563; Found 277.1565.

(1-methoxypropane-1,1-diyl)dibenzene (2m)

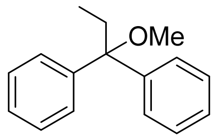

2m

According to the general procedure for the copper-catalyzed three-component 1,2-alkoxy methylation of alkenes. The crude product was purified by flash column chromatography on silica gel (PE/CH_2Cl_2 = 4/1) to afford **2m** (41.7 mg, 92% yield) as a white solid (DTBP was used as methyl source).

MP: 61–62 ºC

^{1}H NMR (400 MHz, $CDCl_3$) δ 7.42 (d, J = 8.0 Hz, 4H), 7.37–7.30 (m, 4H), 7.29–7.16 (m, 2H), 3.11 (s, 3H), 2.40 (q, J = 7.2 Hz, 2H), 0.79 (t, J = 7.2 Hz, 3H).

^{13}C NMR (101 MHz, $CDCl_3$) δ 145.6, 127.9, 127.2, 126.6, 82.7, 50.0, 27.7, 7.4.

IR (neat) cm^{-1} υ: 2942 (w), 1488 (w), 1447 (w), 1194 (w), 1175 (w), 1128 (w), 1068 (m), 1056 (w), 935 (w), 905 (w), 751 (w), 746 (m), 694 (s).

HRMS (ESI) m/z: [M-OCH3]$^+$ Calcd for $C_{15}H_{15}^+$ 195.1168; Found 195.1169.

4,4'-(1-methoxypropane-1,1-diyl)bis(methylbenzene) (2n)

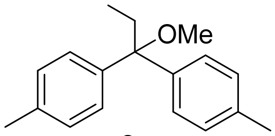

2n

According to the general procedure for the copper-catalyzed three-component 1,2-alkoxy methylation of alkenes. The crude product was purified by flash column chromatography on silica gel (PE/CH_2Cl_2 = 4/1) to afford **2n** (44.1 mg, 87% yield) as a colorless oil (DTBP was used as methyl source).

^{1}H NMR (400 MHz, $CDCl_3$) δ 7.27 (d, J = 7.8 Hz, 4H), 7.12 (d, J = 7.8 Hz, 4H), 3.08 (s, 3H), 2.41-2.26 (q, J = 7.2 Hz, 2H) 2.35 (s, 6H), 0.76 (t, J = 7.2 Hz, 3H).

^{13}C NMR (101 MHz, $CDCl_3$) δ 142.8, 136.0, 128.6, 127.1, 82.6, 49.9, 27.8, 21.1, 7.4.

IR (neat) cm^{-1} υ: 2936 (w), 1511 (w), 1450 (w), 1182 (w), 1107 (m), 1068 (s), 1022 (w), 924 (w), 812 (s), 729 (m).

HRMS (ESI) m/z: [M-OCH_3]$^+$ Calcd for $C_{17}H_{19}^+$ 223.1492; Found 223.1487.

1-(1-methoxy-1-phenylpropyl)-3-methylbenzene (2o)

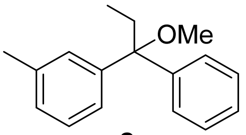

2o

According to the general procedure for the copper-catalyzed three-component 1,2-alkoxy methylation of alkenes. The crude product was purified by flash column chromatography on silica gel (PE/CH_2Cl_2 = 4/1) to afford **2o** (39.8 mg, 83% yield) as a colorless oil (DTBP was used as methyl source).

^{1}H NMR (400 MHz, $CDCl_3$) δ 7.42 (d, J = 7.8 Hz, 2H), 7.38–7.31 (m, 2H), 7.30–7.16 (m, 4H), 7.07 (d, J = 6.8 Hz, 2H), 3.11 (s, 3H), 2.41–2.35 (q, J = 7.4 Hz, 2H), 2.38 (s, 3H), 0.78 (t, J = 7.2 Hz, 3H).

^{13}C NMR (101 MHz, $CDCl_3$) δ 145.7, 145.5, 137.4, 127.9, 127.8, 127.4, 127.1, 126.6, 124.3, 82.7, 50.0, 27.7, 21.79, 7.4.

IR (neat) cm^{-1} υ: 2972 (w), 1604 (w), 1448 (w), 1288 (w), 1172 (w), 1122 (w), 1068 (m), 910 (w), 789 (m), 756 (m), 702 (s).

HRMS (APPI) m/z: $[M-OCH_3]^+$ Calcd for $C_{16}H_{17}^+$ 209.1325; Found 209.1334.

1-chloro-4-(1-methoxy-1-phenylpropyl)benzene (2p)

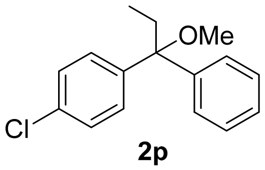

2p

According to the general procedure for the copper-catalyzed three-component 1,2-alkoxy methylation of alkenes. The crude product was purified by flash column chromatography on silica gel (PE/CH_2Cl_2 = 4/1) to afford **2p** (47.5 mg, 91% yield) as a colorless oil (DTBP was used as methyl source).

^{1}H NMR (400 MHz, $CDCl_3$) δ 7.97–6.87 (m, 9H), 3.06 (s, 3H), 2.47–2.21 (m, 2H), 0.74 (t, J = 7.2 Hz, 3H).
^{13}C NMR (101 MHz, $CDCl_3$) δ 145.0, 144.4, 132.4, 128.5, 128.08, 128.06, 127.1, 126.9, 82.4, 50.0, 27.6, 7.2.

IR (neat) cm^{-1} υ: 2940 (w), 1741 (w), 1598 (w), 1490 (m), 1448 (m), 1377 (w), 1197 (m), 1107 (m), 1068 (s), 1014 (m), 927 (w), 823 (s), 762 (s).

HRMS (ESI) m/z: $[M-OCH_3]^+$ Calcd for $C_{15}H_{14}Cl^+$ 229.0786; Found 229.0784.

4,4'-(1-methoxypropane-1,1-diyl)bis(fluorobenzene) (2q)

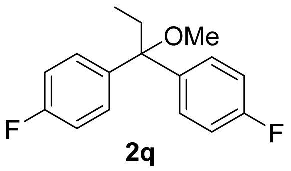

2q

According to the general procedure for the copper-catalyzed three-component 1,2-alkoxy methylation of alkenes. The crude product was purified by flash column chromatography on silica gel (PE/CH_2Cl_2 = 4/1) to afford **2q** (42.3 mg, 81% yield) as a colorless oil (DTBP was used as methyl source).

^{1}H NMR (400 MHz, $CDCl_3$) δ 7.65–7.21 (m, 4H), 7.10–6.87 (m, 4H), 3.05 (s, 3H), 2.31 (q, J = 7.2 Hz, 2H), 0.74 (t, J = 7.2 Hz, 3H).

^{13}C NMR (101 MHz, $CDCl_3$) δ 161.7 (d, J = 245.4 Hz), 141.3 (d, J = 3.2 Hz), 128.8 (d, J = 7.8 Hz), 114.8 (d, J = 21.2 Hz), 82.0, 49.9, 27.8, 7.2.

IR (neat) cm^{-1} υ: 2940 (w), 1603 (m), 1505 (s), 1226 (s), 1158 (m), 1067 (m), 1015 (m), 829 (s), 732 (w).

HRMS (ESI) m/z: $[M–OCH_3]^+$ Calcd for $C_{15}H_{13}F_2^+$ 231.0980; Found 231.098.

2-(2-methoxybutan-2-yl)naphthalene (2r)

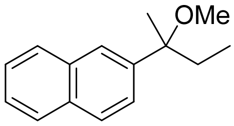

2r

According to the general procedure for the copper-catalyzed three-component 1,2-alkoxy methylation of alkenes. The crude product was purified by flash column chromatography on silica gel (PE/CH_2Cl_2 = 10/1, PE/EtOAc = 50/1) and then by PTLC (PE/DCM = 8/1) to afford **2r** (32,1 mg, 75% yield) as a colorless oil.

^{1}H NMR (400 MHz, $CDCl_3$) δ 7.91–7.72 (m, 4H), 7.61–7.44 (m, 3H), 3.11 (s, 3H), 1.90 (q, J = 7.4 Hz, 2H), 1.62 (s, 3H), 0.80 (t, J = 7.4 Hz, 3H).

^{13}C NMR (100 MHz, $CDCl_3$) δ 142.8, 133.3, 132.8, 128.2, 127.9, 127.6; 126.0, 125.8, 125.3, 124.9, 79.7, 50.6, 34.9, 22.5, 8.5.

IR (neat) cm^{-1} υ: 2971 (w), 2926 (w), 2881 (w), 2826 (w), 1600 (w), 1506 (w), 1463 (w), 1374 (w), 1298 (w), 1168 (m), 1082 (s), 822 (m), 746 (s).

HRMS (ESI) calcd for $C_{15}H_{19}O^+$ $[M+H]^+$ 215.1430; found 215.1431.

1-ethyl-1-methoxy-1,2,3,4-tetrahydronaphthalene (2s)

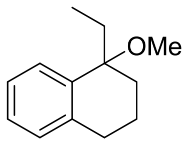

2s

According to the general procedure for the copper-catalyzed three-component 1,2-alkoxy methylation of alkenes. The crude product was purified by flash column chromatography on silica gel (PE/CH_2Cl_2 = 3/1) to afford **2s** (27.8 mg, 73% yield) as a colorless oil.

^{1}H NMR (400 MHz, $CDCl_3$) δ 7.31 (dd, J = 7.5, 1.7 Hz, 1H), 7.11–7.04 (m, 2H), 7.01–6.94 (m, 1H), 2.96 (s, 3H), 2.74–2.54 (m, 2H), 2.01–1.87 (m, 1H), 1.80–1.74 (m, 3H), 1.71 (q, J = 7.4 Hz, 2H), 0.77 (t, J = 7.4 Hz, 3H).

^{13}C NMR (101 MHz, $CDCl_3$) δ 139.7, 138.7, 128.8, 127.0, 126.9, 125.9, 77.9, 50.4, 35.2, 30.0, 29.5, 20.8, 8.4.

IR (neat) cm^{-1} υ: 2936 (w), 1486 (w), 1454 (m), 1186 (w), 1084 (s), 949 (w), 922 (w), 856 (w), 756 (s), 731 (w).

HRMS (ESI) m/z: $[M + Na]^+$ Calcd for $C_{13}H_{18}NaO^+$ 213.1250; Found 213.1251.

(2-ethoxybutan-2-yl)benzene (2t)

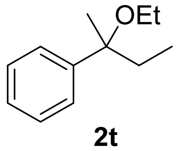

2t

According to the general procedure for the copper-catalyzed three-component 1,2-alkoxy methylation of alkenes. The crude was purified by flash column chromatography on silica gel (PE/CH_2Cl_2 = 10/1, PE/EtOAc = 50/1) and then PTLC (PE/EtOAc = 50/1) to afford **2t** (28.1 mg, 79% yield) as a colorless oil.

^{1}H NMR (400 MHz, $CDCl_3$) δ 7.43–7.22 (m, 5H), 3.32 (dq, J = 8.8, 7.0 Hz, 1H), 3.16 (dq, J = 8.8, 7.0 Hz, 1H), 1.82 (q, J = 7.4 Hz, 2H), 1.53 (s, 3H), 1.19 (t, J = 7.0 Hz, 3H), 0.80 (t, J = 7.4 Hz, 3H).

^{13}C NMR (100 MHz, CDCl3) δ 146.0, 128.1, 126.7, 126.3, 79.2, 57.8, 35.4, 23.3, 16.0, 8.5.

IR (neat) cm^{-1} υ: 2973 (w), 2931 (w), 2878 (w), 1446 (w), 1230 (w), 1163 (w), 1107 (w), 1072 (s), 1029 (w), 759 (m), 700 (s).

HRMS (ESI) calcd for $C_{12}H_{19}O^+$ $[M+H]^+$ 179.1430; found 179.1435.

(2-isopropoxybutan-2-yl)benzene (2u)

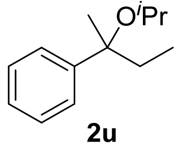

2u

According to the general procedure for the copper-catalyzed three-component 1,2-alkoxy methylation of alkenes. The crude product was purified by flash column chromatography on silica gel (PE/CH_2Cl_2 = 10/1, PE/EtOAc = 50/1) and then by PTLC (PE/EtOAc = 50/1) to afford **2u** (16.3 mg, 42% yield) as a colorless oil.

^{1}H NMR (400 MHz, $CDCl_3$) δ 7.46 (d, J = 7.5 Hz, 2H), 7.34 (dd, J = 7.5, 7.5 Hz, 2H), 7.31–7.22 (m, 1H), 3.59–3.50 (m, 1H), 1.82 (q, J = 7.4 Hz, 2H), 1.58 (s, 3H), 1.14 (d, J = 8.0 Hz, 3H), 1.02 (d, J = 8.1 Hz, 3H), 0.73 (t, J = 7.4 Hz, 3H).

^{13}C NMR (101 MHz, $CDCl_3$) δ 146.0, 127.8, 126.9, 126.8, 79.8, 65.2, 36.3, 25.2, 24.7, 23.2, 8.9.

IR (neat) cm^{-1} υ: 2934 (w), 2934 (w), 2880 (w), 1447 (w), 1377 (w), 1165 (w), 1117 (m), 1049 (w), 1011 (w), 966 (w), 760 (m), 700 (s).

HRMS (ESI) calcd for $C_{13}H_{21}O^+$ $[M+H]^+$ 193.1587; found 193.1592.

General procedure for the copper-catalyzed three-component 1,2-azido methylation of alkenes

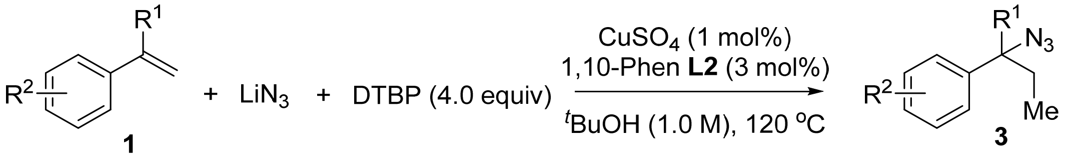

A screw cap tube was charged with $CuSO_4$ (0.32 mg, 0.002 mmol, 0.01 equiv), 1,10-Phen **L2** (1.08 mg, 0.003 mmol, 0.03 equiv) and ^{t}BuOH (2.0 mL). The mixture was stirred at 40 °C for 30 minutes, then cooled to room temperature. Substrate **1** (0.2 mmol, 1.0 equiv), LiN_3 (20% w/w, 0.12 mL, 2.5 equiv) and DTBP (0.15 mL, 4.0 equiv) were added to the above mixture, and the reaction mixture was stirred at 120 °C for 8 hours under N_2 atmosphere. The reaction was quenched with water, and the mixture was extracted with EtOAc. The organic extracts were washed with brine, dried over Na_2SO_4. The solvent was removed under reduced pressure. The residue was purified by flash chromatography to give **3.**

Characterization of compounds 3

(2-azidobutan-2-yl)benzene (3a)

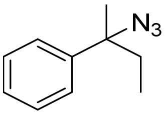

3a

According to the general procedure for the copper-catalyzed three-component 1,2-azido methylation of alkenes. The crude product was purified by flash column chromatography on silica gel (PE) to afford **3a** (28.2 mg, 81% yield) as a colorless oil.

^{1}H NMR (400 MHz, $CDCl_3$) δ 7.44–7.35 (m, 4H), 7.33–7.27 (m, 1H), 1.99–1.82 (m, 2H), 1.69 (s, 3H), 0.83 (t, J = 7.4 Hz, 3H).

^{13}C NMR (101 MHz, $CDCl_3$) δ 143.4, 128.6, 127.3, 125.8, 67.5, 35.2, 25.2, 8.8.

IR (neat) cm^{-1} υ: 2893 (w), 2338 (w), 2176 (m), 2117 (m), 1492 (s), 1411 (s), 1130 (s), 1069 (s), 925 (s), 786 (s), 703(s).

HRMS (ESI) calcd for $C_{10}H_{14}N^+$ $[M-N_2+H]^+$ 148.1126; found 148.1129.

1-(2-azidobutan-2-yl)-4-methylbenzene (3b)

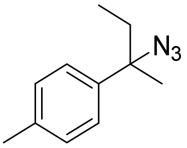

3b

According to the general procedure for the copper-catalyzed three-component 1,2-azido methylation of alkenes. The crude product was purified by flash column chromatography on silica gel (PE) to afford **3b** (27.4 mg, 72% yield) as a colorless oil.

^{1}H NMR (400 MHz, $CDCl_3$) δ 7.19 (d, J = 8.3 Hz, 2H), 7.09 (d, J = 8.0 Hz, 2H), 2.27 (s, 3H), 1.83–1.70 (m, 2H), 1.56 (s, 3H), 0.72 (t, J = 7.4 Hz, 3H).

^{13}C NMR (101 MHz, $CDCl_3$) δ 140.4, 137.0, 129.2, 125.7, 67.4, 35.1, 25.2, 21.1, 8.9.

IR (neat) cm^{-1} υ: 2093 (s), 1513 (w), 1460 (w), 1380 (w), 1294 (w), 1258 (m), 1191 (w), 1149 (w), 1020 (w), 854 (w), 814 (m), 705 (w), 647 (w).

HRMS (ESI) calcd for $C_{11}H_{16}N^+$ $[M-N_2+H]^+$ 162.1283; found 162.1282.

1-(2-azidobutan-2-yl)-4-(trifluoromethoxy)benzene (3c)

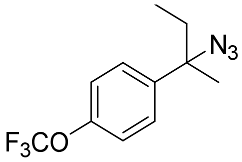

3c

According to the general procedure for the copper-catalyzed three-component 1,2-azido methylation of alkenes. The crude product was purified by flash column chromatography on silica gel (PE) to afford **3c** (32.2 mg, 62% yield) as a colorless oil.

^{1}H NMR (400 MHz, $CDCl_3$) δ 7.45–7.36 (m, 2H), 7.24–7.13 (m, 2H), 1.96–1.78 (m, 2H), 1.66 (s, 3H), 0.80 (t, J = 7.4 Hz, 3H).

^{13}C NMR (101 MHz, $CDCl_3$) δ 148.4, 142.2, 127.3, 120.9, 120.6 (q, J = 258.6 Hz), 66.9, 35.2, 25.4, 8.8.

IR (neat) cm^{-1} υ: 2098 (m), 1509 (w), 1461 (w), 1384 (w), 1253 (s), 1212 (s), 1163 (s), 1019 (w), 847 (w), 833 (w), 677 (w).

HRMS (ESI) calcd for $C_{11}H_{13F3}NO^+$ $[M-N_2+H]^+$ 232.0949; found 232.0945.

1-(2-azidobutan-2-yl)-4-chlorobenzene (3d)

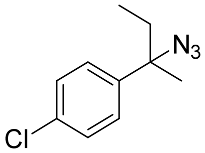

3d

According to the general procedure for the copper-catalyzed three-component 1,2-azido methylation of alkenes. The crude product was purified by flash column chromatography on silica gel (PE) to afford **3d** (36.0 mg, 86% yield) as a colorless oil.

^{1}H NMR (400 MHz, $CDCl_3$) δ 7.36–7.30 (m, 4H), 1.95–1.76 (m, 2H), 1.65 (s, 3H), 0.79 (t, J = 7.4 Hz, 3H).

^{13}C NMR (101 MHz, $CDCl_3$) δ 142.0, 133.2, 128.7, 127.3, 67.0, 35.1, 25.3, 8.8.

HRMS (ESI) calcd for $C_{10}H_{13Cl}N^+$ $[M-N_2+H]^+$ 182.0737; found 182.0737.

IR (neat) cm^{-1} υ: 2098 (m), 1509 (w), 1253 (s), 1212 (s), 1160 (s), 1019 (w), 923 (w), 847 (w), 807 (w), 677 (w), 618 (m).

1-(2-azidobutan-2-yl)-4-bromobenzene (3e)

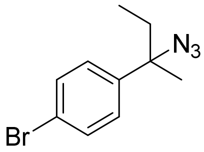

3e

According to the general procedure for the copper-catalyzed three-component 1,2-azido methylation of alkenes. The crude product was purified by flash column chromatography on silica gel (PE) to afford **3e** (32.5 mg, 64% yield) as a colorless oil.

^{1}**H NMR** (400 MHz, $CDCl_3$) δ 7.51(d, J = 8.4 Hz, 2H), 7.28 (d, J = 8.4 Hz, 2H), 1.95–1.80 (m, 2H), 1.67 (s, 3H), 0.81 (t, J = 7.4 Hz, 3H).

^{13}**C NMR** (101 MHz, $CDCl_3$) δ 142.6, 131.6, 127.6, 121.3, 67.0, 35.0, 25.3, 8.8.

IR (neat) cm^{-1} υ: 2095 (s), 1489 (w), 1487 (w), 1397 (w), 1294 (w), 1255 (m), 1102 (w), 1008 (s), 853 (w), 820 (s), 717 (w).

HRMS (ESI) calcd for $C_{10}H_{13}NBr^{+}$ $[M-N_2+H]^{+}$ 226.0231; found 226.0233.

1-(2-azidobutan-2-yl)-4-nitrobenzene (3f)

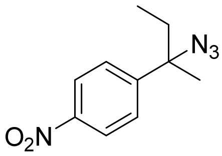

3f

According to the general procedure for the copper-catalyzed three-component 1,2-azido methylation of alkenes. The crude product was purified by flash column chromatography on silica gel (PE) to afford **3f** (34.5 mg, 78% yield) as a pale yellow oil.

^{1}**H NMR** (400 MHz, $CDCl_3$) δ 8.11 (d, J = 9.0 Hz, 2H), 7.46 (d, J = 8.9 Hz, 2H), 1.90–1.73 (m, 2H), 1.60 (s, 3H), 0.69 (t, J = 7.4 Hz, 3H).

^{13}**C NMR** (101 MHz, $CDCl_3$) δ 150.9, 130.2, 126.8, 123.8, 67.0, 35.0, 25.6, 8.7.

IR (neat) cm^{-1} υ: 2095 (s), 1948 (w), 1599(w), 1463 (w), 1411 (w), 1286(w), 1258(m), 1093 (w), 1089 (w), 999 (w), 872 (w), 796 (m), 699 (s).

HRMS (APPI) m/z: $[M–N_2+H]^{+}$ Calcd for $C_{10}H_{13}N_2O_2^{+}$ 193.0972; Found 193.0976.

4-(2-azidobutan-2-yl)benzonitrile (3g)

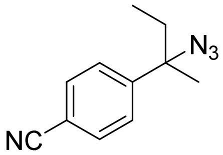

3g

According to the general procedure for the copper-catalyzed three-component 1,2-azido methylation of alkenes. The crude product was purified by flash column chromatography on silica gel (PE) to afford **3g** (32.7 mg, 82% yield) as a pale yellow oil.

^{1}H NMR (400 MHz, $CDCl_3$) δ 7.56 (d, J = 8.6 Hz, 2H), 7.40 (d, J = 8.5 Hz, 2H), 1.87–1.69 (m, 2H), 1.57 (s, 3H), 0.68 (t, J = 7.4 Hz, 3H).

^{13}C NMR (101 MHz, $CDCl_3$) δ 148.9, 132.4, 126.6, 118.7, 111.3, 67.0, 34.9, 25.4, 8.7.

IR (neat) cm^{-1} υ: 2099 (s), 1998(w), 1457 (w), 1385 (w), 1291 (w), 1256(m), 1026 (w), 908 (w), 799 (m), 754 (w).

HRMS (APPI) m/z: $[M–N_2+H]^+$ Calcd for $C_{11}H_{13}N_2^+$ 173.1073; Found 173.1077.

1-(2-azidobutan-2-yl)-3-methoxybenzene (3h)

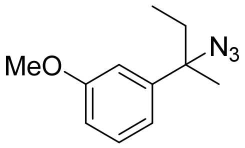

3h

According to the general procedure for the copper-catalyzed three-component 1,2-azido methylation of alkenes. The crude product was purified by flash column chromatography on silica gel (PE) to afford **3h** (2.4 mg, 64% yield) as a colorless oil.

^{1}H NMR (400 MHz, $CDCl_3$) δ 7.25–7.19 (m, 1H), 6.95–6.89 (m, 2H), 6.79–6.74 (m, 1H), 3.78 (s, 3H), 1.88–1.75 (m, 2H), 1.61 (s, 3H), 0.76 (t, J = 7.4 Hz, 3H).

^{13}C NMR (101 MHz, $CDCl_3$) δ 159.8, 145.2, 129.5, 118.1, 112.2, 67.4, 55.4, 35.1, 25.3, 8.8.

IR (neat) cm^{-1} υ: 2093 (s), 1612 (w), 1513 (m), 1462 (w), 1301 (w), 1248 (s), 1181 (m), 1034 (m), 828 (m), 687 (w), 651 (w).

HRMS (ESI) calcd for $C_{11}H_{16}NO^+$ $[M–N_2+H]^+$ 178.1232; found 178.1231.

1-(2-azidobutan-2-yl)-3-methylbenzene (3i)

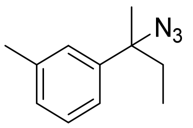

3i

According to the general procedure for the copper-catalyzed three-component 1,2-azido methylation of alkenes. The crude product was purified by flash column chromatography on silica gel (PE) to afford **3i** (27.9 mg, 74% yield) as a colorless oil.

^{1}H NMR (400 MHz, $CDCl_3$) δ 7.28 (t, J = 7.6 Hz, 1H), 7.25–7.18 (m, 2H), 7.12 (d, J = 7.5 Hz, 1H), 2.41 (s, 3H), 1.95–1.85 (m, 2H), 1.68 (s, 3H), 0.84 (t, J = 7.4 Hz, 3H).

^{13}C NMR (101 MHz, $CDCl_3$) δ 143.4, 138.1, 128.4, 128.0, 126.5, 122.8, 67.4, 35.1, 25.3, 21.8, 8.9.

IR 2098 (s), 1605 (w), 1463 (w), 1380 (w), 1268 (w), 1248 (m), 1149 (w), 1046 (w), 782 (m), 704 (s), 670 (w).

HRMS (ESI) calcd for $C_{11}H_{16}N^+$ $[M–N_2+H]^+$ 162.1283; found 162.1280.

1-(2-azidobutan-2-yl)-3-chlorobenzene (3j)

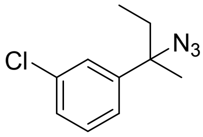

3j

According to the general procedure for the copper-catalyzed three-component 1,2-azido methylation of alkenes. The crude product was purified by flash column chromatography on silica gel (PE) to afford **3j** (32.8 mg, 78% yield) as a colorless oil.

^{1}H NMR (400 MHz, $CDCl_3$) δ 7.40 (s, 1H), 7.35–7.22 (m, 3H), 1.94–1.83 (m, 2H), 1.68 (s, 3H), 0.82 (t, J = 7.4 Hz, 3H).

^{13}C NMR (101 MHz, $CDCl_3$) δ 140.2, 136.4, 133.0, 127.7, 127.2, 125.8, 68.3, 32.8, 24.6, 21.9, 9.1.

IR (neat) cm^{-1} υ: 2097 (s), 1945 (w), 1596 (w), 1569 (w), 1465 (w), 1415 (w), 1411 (w), 1292 (w), 1255 (m), 1099 (w), 1084 (w), 999 (w), 872 (w), 791 (m), 700 (s).

HRMS (ESI) calcd for $C_{10}H_{13Cl}N^+$ $[M–N_2+H]^+$ 182.0737; found 182.0737.

1-(2-azidobutan-2-yl)-2-methylbenzene (3k)

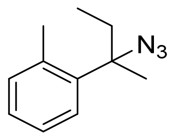

3k

According to the general procedure for the copper-catalyzed three-component 1,2-azido methylation of alkenes. The crude product was purified by flash column chromatography on silica gel (PE) to afford **3k** (23.7 mg, 63% yield) as a colorless oil.

^{1}H NMR (400 MHz, $CDCl_3$) δ 7.36–7.29 (m, 1H), 7.23–7.12 (m, 3H), 2.58 (s, 3H), 2.00–1.91 (m, 2H), 1.73 (s, 3H), 0.83 (t, J = 7.4 Hz, 3H).

^{13}C NMR (101 MHz, $CDCl_3$) δ 140.1, 136.2, 132.9, 127.5, 127.1, 125.6, 68.1, 32.6, 24.5, 21.8, 9.0.

IR (neat) cm^{-1} υ: 2949 (w), 2891 (w), 2338 (w), 2173 (m), 2117 (m), 1490 (s), 1411 (s), 1319 (s), 1130 (s), 1066 (s), 927 (s), 779 (s), 704 (s).

HRMS (ESI) calcd for $C_{11}H_{16}N^+$ $[M-N_2+H]^+$ 162.1283; found 162.1280.

4-(2-azidobutan-2-yl)-1,2-dimethylbenzene (3l)

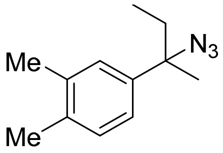

3l

According to the general procedure for the copper-catalyzed three-component 1,2-azido methylation of alkenes. The crude product was purified by flash column chromatography on silica gel (PE) to afford **3l** (26.3 mg, 65% yield) as a colorless oil.

^{1}H NMR (400 MHz, $CDCl_3$) δ 7.17–7.14 (m, 1H), 7.14–7.08 (m, 2H), 2.29 (s, 3H), 2.26 (s, 3H), 1.92–1.81 (m, 2H), 1.64 (s, 3H), 0.81 (t, J = 7.4 Hz, 3H).

^{13}C NMR (101 MHz, $CDCl_3$) δ 140.9, 136.7, 135.6, 129.8, 127.1, 123.2, 67.4, 35.1, 25.2, 20.2, 19.5, 8.9.

IR (neat) cm^{-1} υ: 2123 (m), 1489 (w), 1431 (m), 1357 (w), 1279 (s), 1110 (s), 974 (m), 852 (w), 791 (s).

HRMS (ESI) calcd for $C_{12}H_{18}N^+$ $[M-N_2+H]^+$ 176.1439; found 176.1438.

(3-azidopentan-3-yl)benzene (3m)

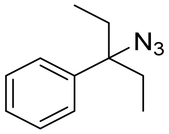

3m

According to the general procedure for the copper-catalyzed three-component 1,2-azido methylation of alkenes. The crude product was purified by flash column chromatography on silica gel (PE) to afford **3m** (28.8 mg, 76% yield) as a colorless oil.

^{1}H NMR (400 MHz, $CDCl_3$) δ 7.31–7.22 (m, 4H), 7.21–7.12 (m, 1H), 1.97–1.81 (m, 4H), 0.71 (t, J = 7.4 Hz, 6H).

^{13}C NMR (101 MHz, $CDCl_3$) δ 141.4, 128.4, 127.0, 126.3, 71.2, 32.5, 8.5.

IR (neat) cm^{-1} υ: 2049 (w), 1800 (w), 1699 (w), 1653 (w), 1542 (m), 1490 (s), 1458 (m), 1255 (s), 1096 (m), 1014 (m), 891 (m), 826 (s), 763 (s), 759 (s).

HRMS (ESI) calcd for $C_{11}H_{16}N^+$ $[M-N_2+H]^+$ 162.1283; found 162.1284.

(3-azido-2-methylpentan-3-yl)benzene (3n)

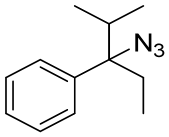

3n

According to the general procedure for the copper-catalyzed three-component 1,2-azido methylation of alkenes. The crude product was purified by flash column chromatography on silica gel (PE) to afford **3n** (29.1 mg, 72% yield) as a colorless oil.

^{1}H NMR (400 MHz, $CDCl_3$) δ 7.30–7.22 (m, 4H), 7.20–7.15 (m, 1H), 2.17–1.93 (m, 3H), 0.88 (d, J = 6.8 Hz, 3H), 0.74 (t, J = 7.3 Hz, 3H), 0.70 (d, J = 6.8 Hz, 3H).

^{13}C NMR (101 MHz, $CDCl_3$) δ 140.3, 128.1, 127.0, 126.9, 73.8, 38.2, 29.4, 18.1, 17.6, 8.8.

IR (neat) cm^{-1} υ: 2096 (s), 1494 (w), 1447 (w), 1265 (w), 936 (w), 887 (w), 760 (m), 702 (s), 632 (m).

HRMS (ESI) calcd for $C_{12}H_{18}N^+$ $[M-N_2+H]^+$ 176.1439; found 176.1440.

(3-azidopentane-1,3-diyl)dibenzene (3o)

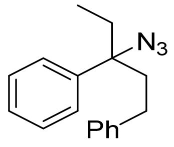

3o

According to the general procedure for the copper-catalyzed three-component 1,2-azido methylation of alkenes. The crude product was purified by flash column chromatography on silica gel (PE) to afford **3o** (33.6 mg, 63% yield) as a colorless oil.

^{1}H NMR (400 MHz, $CDCl_3$) δ 7.36–7.28 (m, 3H), 7.25–7.15 (m, 4H), 7.13–7.07 (m, 1H), 7.06–7.00 (m, 2H), 2.59–2.47 (m, 1H), 2.31–2.20 (m, 1H), 2.20–2.06 (m, 2H), 2.03–1.82 (m, 2H), 0.74 (t, J = 7.4 Hz, 3H).

^{13}C NMR (101 MHz, $CDCl_3$) δ 141.9, 141.3, 128.6, 128.6, 128.4, 127.2, 126.2, 126.1, 70.6, 42.0, 33.1, 30.6, 8.5.

IR (neat) cm^{-1} υ: 2098 (s), 1542 (w), 1490 (w), 1255 (w), 1096 (w), 1014 (w), 891 (w), 826 (w), 763 (m), 759 (w), 699 (s).

HRMS (ESI) calcd for $C_{17}H_{20}N^+$ $[M–N_2+H]^+$ 238.1590; found 238.1596.

(1-azidopropane-1,1-diyl)dibenzene (3p)

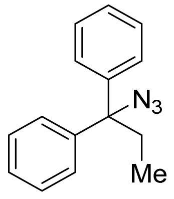

3p

According to the general procedure for the copper-catalyzed three-component 1,2-azido methylation of alkenes. The crude product was purified by flash column chromatography on silica gel (PE) to afford **3p** (37.1 mg, 78% yield) as a colorless oil.

^{1}H NMR (400 MHz, $CDCl_3$) δ 7.40–7.34 (m, 8H), 7.33–7.28 (m, 2H), 2.48 (q, J = 7.3 Hz, 2H), 0.89 (t, J = 7.3 Hz, 3H).

^{13}C NMR (101 MHz, $CDCl_3$) δ 143.1, 128.4, 127.5, 127.3, 73.2, 31.7, 8.7.

IR (neat) cm^{-1} υ: 2096 (s), 1492 (w), 1447 (w), 1252 (m), 1217 (w), 1033 (w), 888 (w), 754 (m), 696 (s).

HRMS (ESI) calcd for $C_{15}H_{16}N^+$ $[M–N_2+H]^+$ 210.1277; found 210.1279.

4,4'-(1-azidopropane-1,1-diyl)bis(fluorobenzene) (3q)

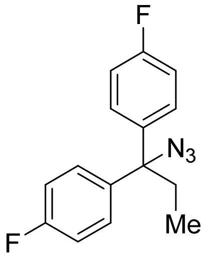

3q

According to the general procedure for the copper-catalyzed three-component 1,2-azido methylation of alkenes. The crude product was purified by flash column chromatography on silica gel (PE) to afford **3q** (29.4 mg, 54% yield) as a colorless oil.

^{1}H NMR (400 MHz, $CDCl_3$) δ 7.40–7.23 (m, 4H), 7.08–7.02 (m, 4H), 2.41 (q, J = 7.3 Hz, 2H), 0.85 (t, J = 7.3 Hz, 3H).

^{13}C NMR (101 MHz, $CDCl_3$) δ 162.1 (d, J = 247.1 Hz), 138.8 (d, J = 3.3 Hz), 129.0 (d, J = 8.1 Hz), 115.3 (d, J = 21.3 Hz), 72.2, 31.9, 8.6.

IR (neat) cm^{-1} υ: 2098 (s), 1510 (w), 1377 (w), 1257 (m), 1099 (w), 901 (w), 759 (w), 700 (m).

HRMS (APPI) m/z: $[M–N_2+H]^+$ Calcd for $C_{15}H_{14}F_2N^+$ 246.1089; Found 246.1093.

4,4'-(1-azidopropane-1,1-diyl)bis(chlorobenzene) (3r)

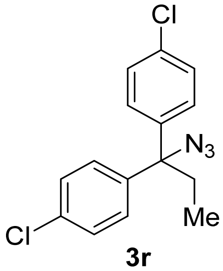

3r

According to the general procedure for the copper-catalyzed three-component 1,2-azido methylation of alkenes. The crude product was purified by flash column chromatography on silica gel (PE) to afford **3r** (50.3 mg, 85% yield) as a colorless oil.

^{1}H NMR (400 MHz, $CDCl_3$) δ 7.31 (d, J = 8.7 Hz, 4H), 7.23 (d, J = 8.7 Hz, 4H), 2.38 (q, J = 7.3 Hz, 2H), 0.83 (t, J = 7.2 Hz, 3H).

^{13}C NMR (101 MHz, $CDCl_3$) δ 141.3, 133.7, 128.7, 128.6, 72.1, 31.5, 8.6.

IR (neat) cm^{-1} υ: 2096 (s), 1494 (w), 1463 (w), 1448 (w), 1312 (w), 1260 (m), 1140 (w), 1079 (w), 1030 (w), 884 (w), 806 (w), 758 (m), 699 (s).

HRMS (ESI) calcd for $C_{15}H_{14}Cl2N^+$ $[M–N_2+H]^+$ 278.0498; found 278.0504.

4,4'-(1-azidopropane-1,1-diyl)bis(methylbenzene) (3s)

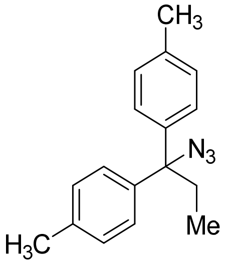

3s

According to the general procedure for the copper-catalyzed three-component 1,2-azido methylation of alkenes. The crude product was purified by flash column chromatography on silica gel (PE) to afford **3s** (32.9 mg, 62% yield) as a colorless oil.

^{1}H NMR (400 MHz, $CDCl_3$) δ 7.21 (d, J = 8.3 Hz, 4H), 7.14 (d, J = 8.1 Hz, 4H), 2.41 (q, J = 7.3 Hz, 2H), 2.35 (s, 6H), 0.85 (t, J = 7.3 Hz, 3H).

^{13}C NMR (101 MHz, $CDCl_3$) δ 140.3, 137.1, 129.0, 127.1, 73.0, 31.8, 21.1, 8.8.

IR (neat) cm^{-1} υ: 2098 (s), 1993 (w), 1510 (w), 1455 (w), 1380 (w), 1285 (w), 1257 (m), 1022 (w), 901 (w), 810 (m), 759 (w).

HRMS (ESI) calcd for $C_{17}H_{20}N^+$ $[M–N_2+H]^+$ 238.1590; found 238.1601.

1-(1-azido-1-phenylpropyl)-4-chlorobenzene (3t)

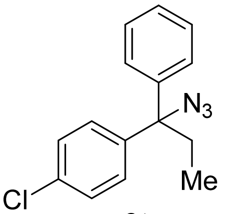

3t

According to the general procedure for the copper-catalyzed three-component 1,2-azido methylation of alkenes. The crude product was purified by flash column chromatography on silica gel (PE) to afford **3t** (47.6 mg, 88% yield) as a colorless oil.

^{1}H NMR (400 MHz, $CDCl_3$) δ 7.41–7.31 (m, 7H), 7.31–7.26 (m, 2H), 2.48–2.40 (m, 2H), 0.88 (t, J = 7.3 Hz, 3H).

^{13}C NMR (101 MHz, $CDCl_3$) δ 142.6, 141.7, 133.4, 128.7, 128.5, 128.5, 127.8, 127.2, 72.6, 31.6, 8.6

IR (neat) cm^{-1} υ: 2097 (s), 1598 (w), 1492 (w), 1448 (w), 1257 (m), 1103 (w), 1014 (w), 825 (m), 764 (m).

HRMS (ESI) calcd for $C_{15}H_{15Cl}N^+$ $[M–N_2+H]^+$ 244.0888; found 244.0886.

1-(1-azido-1-phenylpropyl)-3-methylbenzene (3u)

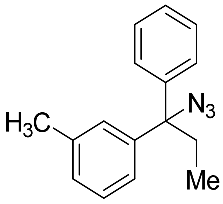

3u

According to the general procedure for the copper-catalyzed three-component 1,2-azido methylation of alkenes. The crude product was purified by flash column chromatography on silica gel (PE) to afford **3u** (35.7 mg, 71% yield) as a colorless oil.

^{1}H NMR (400 MHz, $CDCl_3$) δ 7.40–7.35 (m, 4H), 7.34–7.23 (m, 2H), 7.20–7.10 (m, 3H), 2.47 (q, J = 7.3 Hz, 2H), 2.38 (s, 3H), 0.89 (t, J = 7.3 Hz, 3H).

^{13}C NMR (101 MHz, $CDCl_3$) δ 143.2, 143.0, 138.0, 128.3, 128.2, 128.2, 127.9, 127.4, 127.2, 124.4, 73.2, 31.7, 21.8, 8.8.

IR (neat) cm^{-1} υ: 2161 (w), 1984 (w), 1598 (w), 1448 (w), 1234 (m), 1190 (w), 1014 (m), 891 (m), 764 (s), 704 (s).

HRMS (ESI) m/z: $[M–N_2+H]^+$ Calcd for $C_{16}H_{18}N^+$ 224.1434; Found 224.1432.

General procedure for the copper-catalyzed methylative cycloetherification of alkenes

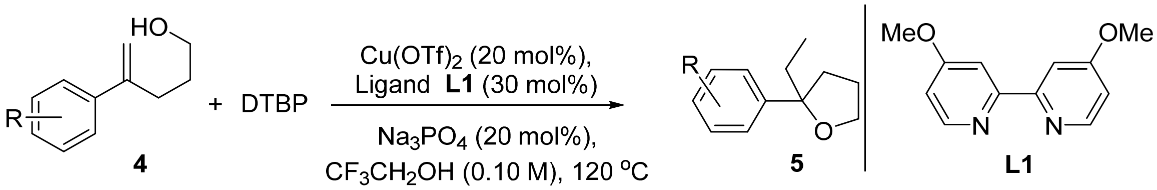

A screw cap tube was charged with $Cu(OTf)_2$ (14.5 mg, 0.04 mmol, 0.2 equiv), **L1** (13.0 mg, 0.06 mmol, 0.03 equiv) and CF_3CH_2OH (2.0 mL). The mixture was stirred at room temperature for 30 minutes. Substrate **4** (0.2 mmol, 1.0 equiv), Na_3PO_4 (6.5 mg, 0.04 mmol, 0.2 equiv) and DTBP (0.15 mL, 4.0 equiv) were added to the above mixture, and the reaction mixture was stirred at 120 °C for 6 hours under N_2 atmosphere. The reaction was quenched with water, and the mixture was extracted with EtOAc. The organic extracts were washed with brine, dried over Na_2SO_4. The solvent was removed under reduced pressure. The residue was purified by flash chromatography to give **5.**

Characterization of compounds 5

2-ethyl-2-phenyltetrahydrofuran (5a)

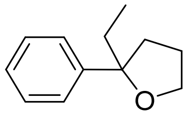

5a

According to the general procedure for the copper-catalyzed methylative cycloetherification of alkenes. The crude product was purified by flash column chromatography on silica gel (PE/EtOAc = 15/1) to afford **5a** (28.8 mg, 82% yield) as a colorless oil.

^{1}H NMR (400 MHz, $CDCl_3$) δ 7.40–7.29 (m, 4H), 7.25–7.18 (m, 1H), 4.00–3.95 (m, 1H), , 1H), 3.90 (dt, J = 5.7, 8.0, Hz, 1H), 2.22–2.15 (m, 1H), 2.09–2.01 (m, 1H), 2.00–1.89 (m, 1H), 1.81 (m, 3H), 0.78 (t, J = 7.4 Hz, 3H).

^{13}C NMR (101 MHz, $CDCl_3$) δ 146.7, 128.0, 126.3, 125.5, 87.3, 67.6, 37.9, 35.2, 25.8, 8.9.

HRMS (APPI) m/z: $[M–C_2H_5]^+$ Calcd for $C_{10}H_{11}O^+$ 147.0804; Found 147.0804.

IR (neat) cm^{-1} υ: 2024 (w), 1494 (w), 1450 (w), 1280 (w), 1261 (w), 1161 (m), 1057 (s), 1032 (s), 802 (m), 760 (s), 702 (s).

2-ethyl-2-(p-tolyl)tetrahydrofuran (5b)

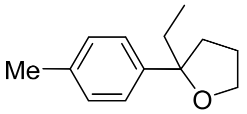

5b

According to the general procedure for the copper-catalyzed methylative cycloetherification of alkenes. The crude product was purified by flash column chromatography on silica gel (PE/EtOAc = 15/1) to afford **5b** (30.9 mg, 81% yield) as a colorless oil.

^{1}H NMR (400 MHz, $CDCl_3$) δ 7.16 (d, J = 8.2 Hz, 2H), 7.05 (d, J = 7.7 Hz, 2H), 3.90–3.86 (m, 1H), 3.79 (td, J = 8.0, 5.6 Hz, 1H), 2.26 (s, 3H), 2.08 (ddd, J = 11.8, 7.9, 4.8 Hz, 1H), 1.96–1.90 (m, 1H), 1.89–1.78 (m, 1H), 1.78–1.63 (m, 3H), 0.68 (t, J = 7.4 Hz, 3H).

^{13}C NMR (101 MHz, $CDCl_3$) δ 143.7, 135.8, 128.7, 125.5, 87.2, 67.5, 37.8, 35.2, 25.8, 21.1, 8.9.

HRMS (APPI) m/z: $[M–C_2H_5]^+$ Calcd for $C_{11}H_{13}O^+$ 161.0961; Found 161.0963.

IR (neat) cm^{-1} υ: 1511 (w), 1456 (w), 1262 (w), 1182 (w), 1101 (w), 1057 (m), 1038 (s), 909 (w), 816 (s), 721 (m).

2-ethyl-2-(4-methoxyphenyl)tetrahydrofuran (5c)

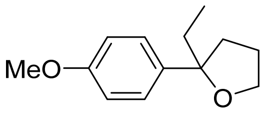

5c

According to the general procedure for the copper-catalyzed methylative cycloetherification of alkenes. The crude product was purified by flash column chromatography on silica gel (PE/EtOAc = 15/1) to afford **5c** (26.8 mg, 65% yield) as a colorless oil.

^{1}H NMR (400 MHz, $CDCl_3$) δ 7.29 (d, J = 8.8 Hz, 2H), 6.88 (d, J = 8.8 Hz, 2H), 3.97 (q, J = 7.3, 6.9 Hz, 1H), 3.91–3.86 (m, 1H), 3.82 (s, 3H), 2.17 (ddd, J = 11.9, 7.9, 4.5 Hz, 1H), 2.08–1.89 (m, 2H), 1.88–1.75 (m, 3H), 0.78 (t, J = 7.4 Hz, 3H).

^{13}C NMR (101 MHz, $CDCl_3$) δ 158.1, 138.7, 126.6, 113.4, 87.0, 67.4, 55.3, 37.7, 35.3, 25.7, 9.0.

HRMS (APPI) m/z: $[M + H]^+$ Calcd for $C_{13}H_{19}O_2^+$ 207.1380; Found 207.1380.

IR (neat) cm^{-1} υ: 1611 (w), 1605 (w), 1510 (s), 1463 (w), 1297 (w), 1245 (s), 1175 (m), 1105 (w), 1036 (s), 913 (w), 830 (s), 721 (w).

2-ethyl-2-(4-fluorophenyl)tetrahydrofuran (5d)

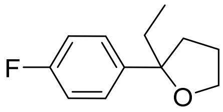

5d

According to the general procedure for the copper-catalyzed methylative cycloetherification of alkenes. The crude product was purified by flash column chromatography on silica gel (PE/EtOAc = 15/1) to afford **5d** (25.2 mg, 68% yield) as a colorless oil.

^{1}H NMR (400 MHz, $CDCl_3$) δ 7.32–7.29 (m, 2H), 6.99 (t, J = 8.8 Hz, 2H), 4.00–3.90 (m, 1H), 3.89–3.83 (m, 1H), 2.13 (ddd, J = 12.5, 8.0, 4.9 Hz, 1H), 2.06–1.98 (m, 1H), 1.98–1.88 (m, 1H), 1.84–1.72 (m, 3H), 0.75 (t, J = 7.4 Hz, 3H).

^{13}C NMR (101 MHz, $CDCl_3$) δ 161.6 (d, J = 243.9 Hz), 142.4 (d, J = 3.1 Hz), 127.1 (d, J = 7.9 Hz), 114.7 (d, J = 21.2 Hz), 87.0, 67.6, 38.0, 35.2, 25.7, 8.9.

HRMS (APPI) m/z: $[M–C_2H_5]^+$ Calcd for $C_{10}H_{10}FO^+$ 165.0710; Found 165.0710.

IR (neat) cm^{-1} υ: 1603 (w), 1507 (s), 1460 (w), 1296 (w), 1222 (m), 1158 (m), 1092 (w), 1057 (m), 1038 (m), 910 (w), 835 (s), 722 (m).

2-(4-chlorophenyl)-2-ethyltetrahydrofuran (5e)

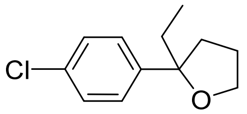

5e

According to the general procedure for the copper-catalyzed methylative cycloetherification of alkenes. The crude product was purified by flash column chromatography on silica gel (PE/EtOAc = 15/1) to afford **5e** (28.8 mg, 67% yield) as a colorless oil.

^{1}H NMR (400 MHz, $CDCl_3$) δ 7.28 (s, 4H), 4.00–3.91 (m, 1H), 3.88–3.83 (m, 1H), 2.15–2.08 (m, 1H), 2.06–1.99 (m, 1H), 1.97–1.87 (m, 1H), 1.84–1.68 (m, 3H), 0.75 (t, J = 7.4 Hz, 3H).

^{13}C NMR (101 MHz, $CDCl_3$) δ 145.3, 132.1, 128.2, 127.0, 86.9, 67.6, 38.0, 35.1, 25.7, 8.8.

HRMS (APPI) m/z: $[M–C_2H_5]^+$ Calcd for $C_{10}H_{10}ClO^+$ 181.0415; Found 181.0415.

IR (neat) cm^{-1} υ: 1489 (m), 1459 (w), 1398 (w), 1292 (w), 1167 (w), 1091 (s), 1058 (s), 1013 (s), 910 (m), 823 (s), 744 (w).

2-(4-bromophenyl)-2-ethyltetrahydrofuran (5f)

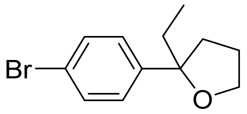

5f

According to the general procedure for the copper-catalyzed methylative cycloetherification of alkenes. The crude product was purified by flash column chromatography on silica gel (PE/EtOAc = 15/1) to afford **5f** (36.2 mg, 71% yield) as a colorless oil.

^{1}H NMR (400 MHz, $CDCl_3$) δ 7.43 (d, J = 8.5 Hz, 2H), 7.23 (d, J = 8.5 Hz, 2H), 4.03–3.91 (m, 1H), 3.89–3.82 (m, 1H), 2.11 (ddd, J = 12.7, 8.0, 5.0 Hz, 1H), 2.06–1.99 (m, 1H), 1.96–1.88 (m, 1H), 1.83–1.67 (m, 3H), 0.74 (t, J = 7.4 Hz, 3H).

^{13}C NMR (101 MHz, $CDCl_3$) δ 145.9, 131.1, 127.4, 120.2, 87.0, 67.6, 38.0, 35.0, 25.7, 8.8.

HRMS (APPI) m/z: $[M–C_2H_5]^+$ Calcd for $C_{10}H_{10}BrO^+$ 224.9910; Found 224.9919.

IR (neat) cm^{-1} υ: 2163 (w), 1590 (w), 1484 (m), 1461 (w), 1392 (w), 1286 (w), 1166 (w), 1095 (m), 1057 (s), 1010 (s), 910 (m), 823 (s), 727 (m).

2-([1,1'-biphenyl]-4-yl)-2-ethyltetrahydrofuran (5g)

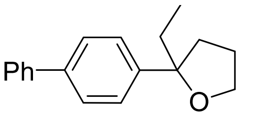

5g

According to the general procedure for the copper-catalyzed methylative cycloetherification of alkenes. The crude product was purified by flash column chromatography on silica gel (PE/EtOAc = 15/1) to afford **5g** (38.5 mg, 76% yield) as a white solid.

MP: 37–39 ºC

^{1}H NMR (400 MHz, $CDCl_3$) δ 7.62 (d, J = 7.1 Hz, 2H), 7.57 (d, J = 8.2 Hz, 2H), 7.49–7.40 (m, 4H), 7.34 (t, J = 7.4 Hz, 1H), 4.01 (q, J = 7.4 Hz, 1H), 3.93 (td, J = 8.0, 5.8 Hz, 1H), 2.23 (ddd, J = 12.6, 8.0, 5.0 Hz, 1H), 2.12– 2.05 (m, 1H), 2.01–1.93 (m, 1H), 1.94–1.77 (m, 3H), 0.83 (t, J = 7.4 Hz, 3H).

^{13}C NMR (101 MHz, $CDCl_3$) δ 145.9, 141.1, 139.2, 128.8, 127.2, 127.1, 126.8, 126.0, 87.2, 67.6, 37.9, 35.2, 25.8, 9.0.

HRMS (APPI) m/z: $[M–C_2H_5]^+$ Calcd for $C_{16}H_{15}O^+$ 223.1117; Found 223.1117.

IR (neat) cm^{-1} υ: 1497 (s), 1425 (s), 1419 (s), 1304 (s), 1219 (s), 1143 (s), 1072 (m), 1024 (s), 953 (m), 889 (m), 815 (w), 742 (w).

2-ethyl-2-(4-isopropylphenyl)tetrahydrofuran (5h)

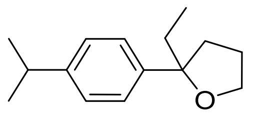

5h

According to the general procedure for the copper-catalyzed methylative cycloetherification of alkenes. The crude product product was purified by flash column chromatography on silica gel (PE/EtOAc = 15/1) to afford **5h** (31.8 mg, 73% yield) as a colorless oil.

^{1}H NMR (400 MHz, $CDCl_3$) δ 7.29 (d, J = 8.2 Hz, 2H), 7.20 (d, J = 8.1 Hz, 2H), 4.09–3.94 (m, 1H), 3.92–3.87 (m, 1H), 2.92 (hept, J = 6.9 Hz, 1H), 2.20 (ddd, J = 12.6, 8.0, 5.0 Hz, 1H), 2.09–2.01 (m, 1H), 2.00–1.89 (m, 1H), 1.91–1.73 (m, 3H), 1.28 (d, J = 7.0 Hz, 6H), 0.80 (t, J = 7.4 Hz, 3H).

^{13}C NMR (101 MHz, $CDCl_3$) δ 146.7, 144.0, 126.0, 125.4, 87.2, 67.5, 37.7, 35.3, 33.8, 25.8, 24.17, 24.15, 9.0.

HRMS (APPI) m/z: $[M–C_2H_5]^+$ Calcd for $C_{13}H_{17}O^+$ 189.1274; Found 189.1273.

IR (neat) cm^{-1} υ: 2023 (w), 1509 (w), 1461 (w), 1411 (w), 1363 (w), 1282 (w), 1160 (m), 1107 (m), 1055 (s), 1052 (s), 938 (w), 829 (s), 782 (w), 714 (w), 692 (w).

2-ethyl-2-(m-tolyl)tetrahydrofuran (5i)

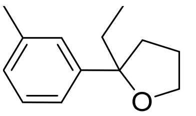

5i

According to the general procedure for the copper-catalyzed methylative cycloetherification of alkenes. The crude product was purified by flash column chromatography on silica gel (PE/EtOAc = 15/1) to afford **5i** (30.2 mg, 79% yield) as a colorless oil.

^{1}H NMR (400 MHz, $CDCl_3$) δ 7.21 (t, J = 7.6 Hz, 1H), 7.18 (s, 1H), 7.14 (d, J = 7.8 Hz, 1H), 7.03 (d, J = 7.4 Hz, 1H), 3.97 (q, J = 7.3 Hz, 1H), 3.92–3.86 (m, 1H), 2.36 (s, 3H), 2.17 (ddd, J = 12.6, 8.1, 5.2 Hz, 1H), 2.07–2.00 (m, 1H), 1.98 –1.88 (m, 1H), 1.85–1.74 (m, 3H), 0.77 (t, J = 7.4 Hz, 3H).

^{13}C NMR (101 MHz, $CDCl_3$) δ 146.7, 137.5, 127.9, 127.1, 126.2, 122.6, 87.2, 67.6, 37.8, 35.2, 25.8, 21.8, 8.9.

HRMS (APPI) m/z: $[M\text{-}C_2H_5]^+$ Calcd for $C_{11}H_{13}O^+$ 161.0961; Found 161.0961.

IR (neat) cm^{-1} υ: 2023 (w), 1607 (w), 1487 (w), 1459 (w), 1377 (w), 1281 (w), 1165 (m), 1121 (m), 1056 (s), 1040 (s), 910 (m), 784 (s), 722 (s).

2-ethyl-2-(3-methoxyphenyl)tetrahydrofuran (5j)

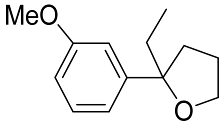

5j

According to the general procedure for the copper-catalyzed methylative cycloetherification of alkenes. The crude product was purified by flash column chromatography on silica gel (PE/EtOAc = 15/1) to afford **5j** (25.3 mg, 61% yield) as a colorless oil.

^{1}H NMR (400 MHz, $CDCl_3$) δ 7.26 (t, J = 7.9 Hz, 1H), 6.98 (brs, 1H), 6.94 (dt, J = 7.8, 1.3 Hz, 1H), 6.78 (ddd, J = 8.2, 2.6, 1.1 Hz, 1H), 3.99 (q, J = 7.4 Hz, 1H), 3.91 (td, J = 8.0, 5.7 Hz, 1H), 3.84 (s, 3H), 2.19 (ddd, J = 12.5, 8.0, 4.9 Hz, 1H), 2.08–2.01 (m, 1H), 1.99–1.88 (m, 1H), 1.87–1.76 (m, 3H), 0.79 (t, J = 7.4 Hz, 3H).

^{13}C NMR (101 MHz, $CDCl_3$) δ 159.5, 148.7, 129.0, 118.0, 111.6, 111.4, 87.2, 67.6, 55.3, 37.9, 35.2, 25.8, 8.9.

HRMS (APPI) m/z: $[M–C_2H_5]^+$ Calcd for $C_{11}H_{13}O_2^+$ 177.0910; Found 177.0909.

IR (neat) cm^{-1} υ: 1600 (w), 1582 (w), 1485 (w), 1463 (w), 1434 (w), 1315 (w), 1285 (m), 1255 (m), 1166 (w), 1118 (w), 1048 (s), 928 (w), 876 (w), 821 (w), 779 (m), 734 (m), 723 (m).

2-(3-chlorophenyl)-2-ethyltetrahydrofuran (5k)

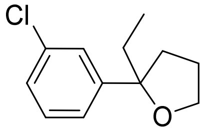

5k

According to the general procedure for the copper-catalyzed methylative cycloetherification of alkenes. The crude product was purified by flash column chromatography on silica gel (PE/EtOAc = 15/1) to afford **5k** (28.7 mg, 68% yield) as a colorless oil.

^{1}H NMR (400 MHz, $CDCl_3$) δ 7.29 (t, J = 1.8 Hz, 1H), 7.18–7.13 (m, 2H), 7.13–7.08 (m, 1H), 3.92–3.85 (m, 1H), 3.80 (td, J = 8.0, 5.6 Hz, 1H), 2.00–1.93 (m, 1H), 1.91–1.81 (m, 1H), 1.92–1.80 (m, 1H), 1.77–1.64 (m, 3H), 0.68 (t, J = 7.4 Hz, 3H).

^{13}C NMR (101 MHz, $CDCl_3$) δ 149.1, 134.1, 129.4, 126.5, 125.8, 123.7, 86.9, 67.7, 38.0, 35.1, 25.7, 8.8.

HRMS (APPI) m/z: $[M–C_2H_5]^+$ Calcd for $C_{10}H_{10}ClO^+$ 181.0415; Found 181.0415.

IR (neat) cm^{-1} υ: 1596 (w), 1571 (w), 1465 (w), 1421 (w), 1282 (w), 1223 (w), 1164 (w), 1117 (m), 1062 (m), 1037 (s), 924 (w), 781 (s), 700 (s).

2-(3,5-dimethylphenyl)-2-ethyltetrahydrofuran (5l)

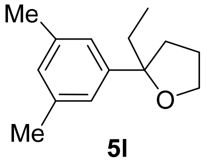

5l

According to the general procedure for the copper-catalyzed methylative cycloetherification of alkenes. The crude product was purified by flash column chromatography on silica gel (PE/EtOAc = 15/1) to afford **5l** (26.3 mg, 64% yield) as a colorless oil.

^{1}H NMR (400 MHz, $CDCl_3$) δ 6.97 (s, 2H), 6.86 (s, 1H), 3.96 (q, J = 7.3 Hz, 1H), 3.89 (td, J = 8.0, 5.8 Hz, 1H), 2.32 (s, 6H), 2.16 (ddd, J = 11.9, 8.2, 5.2 Hz, 1H), 2.06-1.97 (m, 1H), 1.96-1.88 (m, 1H), 1.84-1.76 (m, 3H), 0.78 (t, J = 7.4 Hz, 3H).

^{13}C NMR (101 MHz, $CDCl_3$) δ 146.8, 137.4, 128.0, 123.3, 87.2, 67.6, 37.8, 35.2, 25.8, 21.3, 9.0.

HRMS (APPI) m/z: $[M–C_2H_5]^+$ Calcd for $C_{12}H_{15}O^+$ 175.1117; Found 175.1117.

IR (neat) cm^{-1} υ: 2357 (w), 2341 (w), 2274 (w), 2160 (m), 2022 (w), 1604 (w), 1459 (m), 1378 (w), 1162 (m), 1057 (s), 1038 (s), 848 (s), 761 (m), 721 (s).

4-(2-ethyltetrahydrofuran-2-yl)benzonitrile (5m)

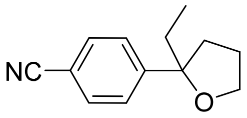

5m

According to the general procedure for the copper-catalyzed methylative cycloetherification of alkenes. The crude product was purified by flash column chromatography on silica gel (PE/EtOAc = 15/1) to afford **5m** (30.2 mg, 75% yield) as a colorless oil.

^{1}H NMR (400 MHz, $CDCl_3$) δ 7.60 (d, *J* = 8.0 Hz, 2H), 7.46 (d, *J* = 8.1 Hz, 2H), 3.98 (q, *J* = 7.5 Hz, 1H), 3.90-3.83 (m, 1H), 2.10 (t, *J* = 7.3 Hz, 2H), 2.01-1.90 (m, 1H), 1.81 (q, *J* = 7.2 Hz, 2H), 1.78–1.70 (m, 3H), 0.73 (t, *J* = 7.4 Hz, 3H).

^{13}C NMR (101 MHz, $CDCl_3$) δ 152.5, 131.9, 126.2, 119.1, 110.2, 86.9, 67.8, 38.0, 34.8, 25.6, 8.6.

HRMS (APCI) m/z: $[M + H]^+$ Calcd for $C_{13}H_{16}NO^+$ 202.1226; Found 202.1223.

IR (neat) cm^{-1} υ: 2952 (s), 2250 (w), 2152 (w), 2015 (w), 1783 (w), 1590 (w), 1479 (m), 1420 (m), 1351 (m), 1143 (m), 1113 (s), 989 (s), 920 (s), 817 (s).

2-ethyl-2-phenyltetrahydro-2H-pyran (5n)

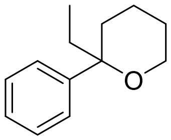

5n

According to the general procedure for the copper-catalyzed methylative cycloetherification of alkenes. The crude product was purified by flash column chromatography on silica gel (PE/EtOAc = 15/1) to afford **5**n (28.9 mg, 76% yield) as a colorless oil.

^{1}H NMR (400 MHz, $CDCl_3$) *δ* 7.47–7.31 (m, 4H), 7.30–7.24 (m, 1H), 3.82–3.64 (m, 1H), 3.56–3.48 (m, 1H), 2.36–2.29 (m, 1H), 1.85–1.59 (m, 5H), 1.58–1.34 (m, 2H), 0.69 (t, *J* = 7.5 Hz, 3H).

^{13}C NMR (101 MHz, $CDCl_3$) *δ* 143.4, 128.3, 127.1, 126.5, 78.8, 62.7, 37.6, 32.7, 26.4, 20.0, 7.8.

HRMS (APPI) m/z: $[M–C_2H_5]^+$ Calcd for $C_{11}H_{13}O^+$ 161.0961; Found 161.0967.

IR (neat) cm^{-1} υ: 2936 (w), 1450 (w), 1284 (w), 1178 (w), 1084 (s), 1049 (m), 997 (w), 889 (w), 758 (s), 702 (s).

General procedure for the copper-catalyzed methylative lactonization of alkenes

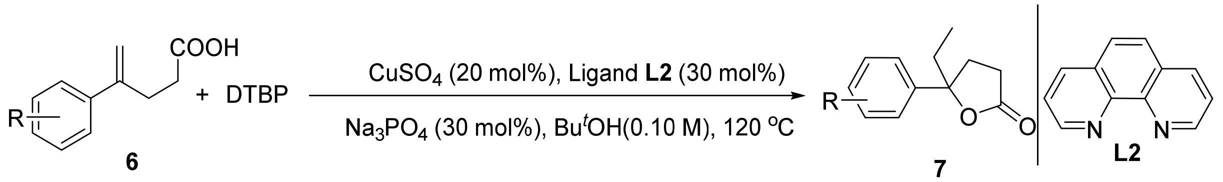

A screw cap tube was charged with $CuSO_4$ (6.4 mg, 0.04 mmol, 0.2 equiv), 1,10-Phen **L2** (10.8 mg, 0.06 mmol, 0.03 equiv) and CF_3CH_2OH (2.0 mL). The mixture was stirred at room temperature for 30 minutes. Substrate **6** (0.2 mmol, 1.0 equiv), Na_3PO_4 (9.8 mg, 0.06 mmol, 0.3 equiv) and DTBP (0.15 mL, 4.0 equiv) were added to the above mixture, and the reaction mixture was stirred at 120°C for 6 hours under N_2 atmosphere. The reaction was quenched with water, and extracted with EtOAc. The organic extracts were washed with brine, dried over Na_2SO_4. The solvent was removed under reduced pressure. The residue was purified by flash chromatography to give **7.**

Characterization of compounds 7

5-(4-chlorophenyl)-5-ethyldihydrofuran-2(3H)-one (7a)

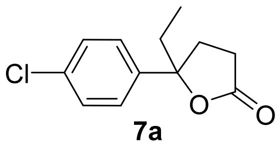

7a

According to the general procedure for the copper-catalyzed methylative lactonization of alkenes. The crude product was purified by flash column chromatography on silica gel (PE/EtOAc = 4/1) to afford **7a** (34.2 mg, 76% yield) as a colorless oil.

^{1}H NMR (400 MHz, $CDCl_3$) δ 7.36 (d, J = 8.6 Hz, 2H), 7.29 (d, J = 8.7 Hz, 2H), 2.68–2.54 (m, 1H), 2.55–2.38 (m, 3H), 1.99 (q, J = 7.4 Hz, 2H), 0.83 (t, J = 7.4 Hz, 3H).

^{13}C NMR (101 MHz, $CDCl_3$) δ 176.4, 141.4, 133.6, 128.8, 126.4, 89.4, 35.3, 34.6, 28.8, 8.3.

HRMS (ESI) m/z: $[M + Na]^+$ Calcd for $C_{12}H_{13}ClNaO_2^+$ 247.0496; Found 247.0498.

IR (neat) cm^{-1} υ: 1771 (s), 1492 (w), 1463 (w), 1292 (w), 1229 (w), 1194 (m), 1118 (w), 1091 (m), 1013 (m), 962 (s), 914 (m), 829 (s), 723 (w).

5-ethyl-5-(p-tolyl)dihydrofuran-2(3H)-one (7b)

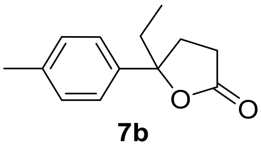

7b

According to the general procedure for the copper-catalyzed methylative lactonization of alkenes. The crude product was purified by flash column chromatography on silica gel (PE/EtOAc = 8/1) to afford **7b** (26.8 mg, 66% yield) as a colorless oil.

^{1}H NMR (400 MHz, $CDCl_3$) δ 7.21 (d, J = 8.4 Hz, 2H), 7.17 (d, J = 8.2 Hz, 2H), 2.62–2.51 (m, 1H), 2.50–2.37 (m, 3H), 2.34 (s, 3H), 1.97 (q, J = 7.4 Hz, 2H), 0.81 (t, J = 7.4 Hz, 3H).

^{13}C NMR (101 MHz, $CDCl_3$) δ 176.9, 139.8, 137.3, 129.2, 124.8, 90.1, 35.4, 34.7, 28.9, 21.1, 8.4.

HRMS (ESI) m/z: $[M + H]^+$ Calcd for $C_{12}H_{14}BrO_2^+$ 269.0172; Found 269.0168.

IR (neat) cm^{-1} υ: 2159 (w), 1758 (w), 1612 (w), 1513 (s), 1465 (w), 1305 (w), 1251 (s), 1177 (s), 1033 (m), 833 (s), 677 (w).

5-ethyl-5-(4-methoxyphenyl)dihydrofuran-2(3H)-one (7c)

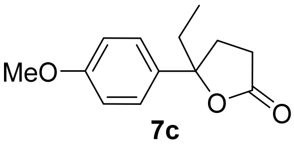

7c

According to the general procedure for the copper-catalyzed methylative lactonization of alkenes. The crude product was purified by flash column chromatography on silica gel (PE/EtOAc = 3/1) to afford **7c** (23.9 mg, 54% yield) as a colorless oil.

^{1}H NMR (400 MHz, $CDCl_3$) δ 7.24 (d, J = 8.9 Hz, 2H), 6.88 (d, J = 8.8 Hz, 2H), 3.80 (s, 4H), 2.63–2.50 (m, 1H), 2.53–2.29 (m, 3H), 1.96 (q, J = 7.4 Hz, 2H), 0.80 (t, J = 7.4 Hz, 3H).

^{13}C NMR (101 MHz, $CDCl_3$) δ 176.9, 159.0, 134.7, 126.1, 113.9, 90.0, 55.4, 35.4, 34.6, 28.9, 8.4.

HRMS (ESI) m/z: $[M + Na]^+$ Calcd for $C_{13}H_{16}NaO_3^+$ 243.0992; Found 243.0997.

IR (neat) cm^{-1} υ: 1770 (s), 1613 (w), 1514 (m), 1463 (w), 1304 (w), 1251 (s), 1179 (s), 1136 (w), 1103 (w), 1031 (m), 960 (m), 914 (m), 832 (s), 788 (w), 670 (w).

5-ethyl-5-(4-fluorophenyl)dihydrofuran-2(3H)-one (7d)

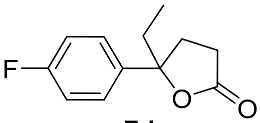

7d

According to the general procedure for the copper-catalyzed methylative lactonization of alkenes. The crude product was purified by flash column chromatography on silica gel (PE/EtOAc = 4/1) to afford **7d** (28.7 mg, 69% yield) as a colorless oil.

^{1}H NMR (400 MHz, $CDCl_3$) δ 7.33–7.26 (m, 2H), 7.09–7.00 (m, 2H), 2.64–2.53 (m, 1H), 2.51–2.38 (m, 3H), 1.96 (q, J = 7.4 Hz, 2H), 0.80 (t, J = 7.4 Hz, 3H).

^{13}C NMR (101 MHz, $CDCl_3$) δ 176.5, 162.2 (d, J = 246.5 Hz), 138.6 (d, J = 3.3 Hz), 126.6 (d, J = 8.1 Hz), 115.5 (d, J = 21.3 Hz), 89.6, 35.4, 34.6, 28.8, 8.3.

HRMS (ESI) m/z: $[M + Na]^+$ Calcd for $C_{12}H_{13}FNaO_2^+$ 231.0792; Found 231.0795.

IR (neat) cm^{-1} υ: 1773 (s), 1603 (w), 1510 (s), 1460 (w), 1302 (w), 1226 (s), 1192 (s), 1162 (m), 1114 (m), 1030 (w), 967 (m), 915 (m), 837 (s).

5-ethyl-5-phenyldihydrofuran-2(3H)-one (7e)

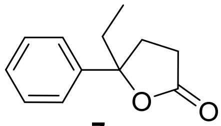

7e

According to the general procedure for the copper-catalyzed methylative lactonization of alkenes. The crude product was purified by flash column chromatography on silica gel (PE/EtOAc = 4/1) to afford **7e** (24.2 mg, 63% yield) as a colorless oil.

^{1}H NMR (400 MHz, $CDCl_3$) δ 7.32-7.29 (m, 1H), 7.29 – 7.26 (m, 2H), 7.26 – 7.18 (m, 2H), 2.56 – 2.45 (m, 1H), 2.45 – 2.32 (m, 3H), 1.92 (q, J = 7.4 Hz, 2H), 0.75 (t, J = 7.4 Hz, 3H).

^{13}C NMR (101 MHz, $CDCl_3$) δ 176.8, 142.8, 128.6, 127.6, 124.8, 90.0, 35.4, 34.7, 28.9, 8.4.

HRMS (ESI) m/z: $[M + Na]^+$ Calcd for $C_{12}H_{13}ClNaO_2^+$ 247.0496; Found 247.0498.

IR (neat) cm^{-1} υ: 1770 (s), 1448 (w), 1318 (w), 1248 (w), 1232 (w), 1194 (m), 1135 (w), 1110 (m), 1028 (w), 962 (m), 920 (m), 765 (s), 701 (s).

5-(4-bromophenyl)-5-ethyldihydrofuran-2(3H)-one (7f)

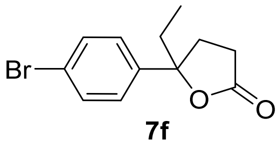

7f

According to the general procedure for the copper-catalyzed methylative lactonization of alkenes. The crude product was purified by flash column chromatography on silica gel (PE/EtOAc = 4/1) to afford **7f** (33.5 mg, 62% yield) as a colorless oil.

^{1}H NMR (400 MHz, $CDCl_3$) δ 7.49 (d, J = 8.6 Hz, 2H), 7.20 (d, J = 8.6 Hz, 2H), 2.69–2.52 (m, 1H), 2.51–2.32 (m, 3H), 1.96 (q, J = 7.4 Hz, 2H), 0.80 (t, J = 7.4 Hz, 3H).

^{13}C NMR (101 MHz, $CDCl_3$) δ 176.4, 142.0, 131.7, 126.7, 121.7, 89.4, 35.2, 34.6, 28.8, 8.3.

HRMS (ESI/QTOF) m/z: $[M + H]^+$ Calcd for $C_{12}H_{14}BrO_2^+$ 269.0172; Found 269.0168.

IR (neat) cm^{-1} υ: 1771 (s), 1488 (w), 1463 (w), 1396 (w), 1230 (w), 1195 (m), 1115 (w), 1100 (w), 1009 (m), 961 (m), 913 (m), 910 (m), 824 (m), 722 (w).

5-([1,1'-biphenyl]-4-yl)-5-ethyldihydrofuran-2(3H)-one (7g)

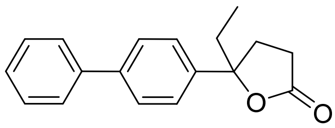

7g

According to the general procedure for the copper-catalyzed methylative lactonization of alkenes. The crude product was purified by flash column chromatography on silica gel (PE/EtOAc = 5/1) to afford **7g** (42.3 mg, 79% yield) as a white solid.

MP: 88–89 ºC.

^{1}H NMR (400 MHz, $CDCl_3$) δ 7.65–7.54 (m, 4H), 7.49–7.39 (m, 4H), 7.39–7.32 (m, 1H), 2.67–2.41 (m, 4H), 2.04 (q, J = 7.4 Hz, 2H), 0.87 (t, J = 7.4 Hz, 3H).

^{13}C NMR (101 MHz, $CDCl_3$) δ 176.8, 141.8, 140.5, 140.5, 128.9, 127.6, 127.3, 127.2, 125.4, 89.9, 35.4, 34.7, 28.9, 8.4.

HRMS (ESI) m/z: $[M + H]^+$ Calcd for $C_{18}H_{19}O_2^+$ 267.1380; Found 267.1382.

IR (neat) cm^{-1} υ: 1764 (s), 1489 (w), 1463 (w), 1401 (w), 1232 (w), 1193 (m), 1137 (w), 1104 (m), 1006 (w), 961 (m), 916 (m), 914 (m), 836 (m), 762 (s), 728 (s), 692 (s).

5-ethyl-5-(4-isopropylphenyl)dihydrofuran-2(3H)-one (7h)

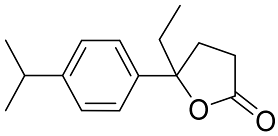

7h

According to the general procedure for the copper-catalyzed methylative lactonization of alkenes. The crude product was purified by flash column chromatography on silica gel (PE/EtOAc = 5/1) to afford **7h** (24.3 mg, 52% yield) as a colorless oil.

^{1}H NMR (400 MHz, $CDCl_3$) δ 7.46–7.00 (m, 4H), 2.83 (hept, J = 6.9 Hz, 1H), 2.55–2.44 (m, 1H), 2.44–2.26 (m, 3H), 1.91 (q, J = 7.4 Hz, 2H), 1.17 (d, J = 6.9 Hz, 6H), 0.75 (t, J = 7.4 Hz, 3H).

^{3}C NMR (101 MHz, $CDCl_3$) δ 176.9, 148.2, 140.1, 126.6, 124.8, 90.1, 35.4, 34.6, 33.8, 28.9, 24.0, 8.4

HRMS (ESI) m/z: $[M + H]^+$ Calcd for $C_{12}H_{14}BrO_2^+$ 269.0172; Found 269.0168.

IR (neat) cm^{-1} υ: 1770 (s), 1463 (w), 1230 (w), 1187 (m), 1135 (w), 1107 (w), 1027 (w), 962 (m), 916 (w), 910 (w), 834 (m), 791 (w), 668 (w).

4-(2-ethyl-5-oxotetrahydrofuran-2-yl)benzonitrile (7i)

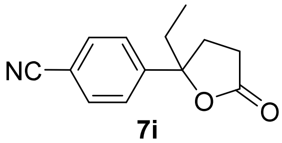

7i

According to the general procedure for the copper-catalyzed methylative lactonization of alkenes. The crude product was purified by flash column chromatography on silica gel (PE/EtOAc = 5/1) to afford 7i (35.1 mg, 82% yield) as a colorless oil.

^{1}H NMR (400 MHz, $CDCl_3$) δ 7.67 (d, J = 8.0 Hz, 2H), 7.46 (d, J = 8.0 Hz, 2H), 2.77 – 2.56 (m, 1H), 2.56 – 2.33 (m, 3H), 1.99 (q, J = 7.2 Hz, 2H), 0.80 (t, J = 7.4 Hz, 3H).

^{13}C NMR (101 MHz, $CDCl_3$) δ 175.7, 148.2, 132.4, 125.6, 118.4, 111.7, 88.9, 35.0, 34.4, 28.5, 8.1.

HRMS (APCI) m/z: $[M + H]^+$ Calcd for $C_{13}H_{14}NO_2^+$ 216.1019; Found 216.1019.

IR (neat) cm^{-1} υ: 2975 (w), 2229 (w), 2158 (w), 1771 (s), 1610 (w), 1506 (w), 1460 (w), 1406 (w), 1345 (w), 1229 (m), 1192 (m), 1104 (m), 1030 (m), 965 (s), 915 (m), 839 (s).

trans-4-methyl-5-phenyldihydrofuran-2(3H)-one (7j)

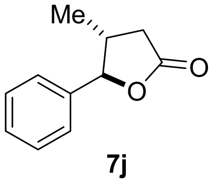

7j

According to the general procedure for the copper-catalyzed methylative lactonization of alkenes. The crude product was purified by flash column chromatography on silica gel (PE/EtOAc = 4/1) to afford **7j** (28.7 mg, 41% yield) as a colorless oil. The physical and spectroscopic data were in accordance with those reported in the literature.[7]

^{1}H NMR (400 MHz, $CDCl_3$) δ 7.57 – 7.30 (m, 5H), 4.94 (d, J = 8.3 Hz, 1H), 2.79 (dd, J = 16.9, 7.7 Hz, 1H), 2.56 – 2.41 (m, 1H), 2.34 (dd, J = 16.9, 10.4 Hz, 1H), 1.20 (d, J = 6.5 Hz, 3H).

^{13}C NMR (101 MHz, $CDCl_3$) δ 176.2, 138.1, 128.9, 126.0, 88.3, 40.0, 37.4, 16.7.

General procedure for the copper-catalyzed methylative cycloamination of alkenes

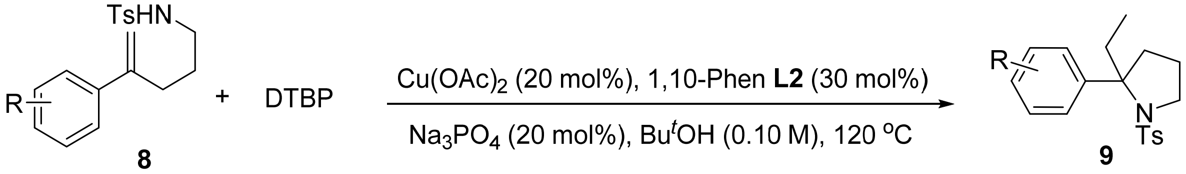

A screw cap tube was charged with $Cu(OAc)_2$ (7.3 mg, 0.04 mmol, 0.2 equiv), 1,10-Phen (10.8 mg, 0.06 mmol, 0.03 equiv) and ^{t}BuOH (2.0 mL). The mixture was stirred at room temperature for 30 minutes. Substrate **8** (0.2 mmol, 1.0 equiv), Na_3PO_4 (6.5 mg, 0.04 mmol, 0.2 equiv) and DTBP (0.15 mL, 4.0 equiv) were added to the above mixture, and the reaction mixture was stirred for 3 hours at 120 °C under N_2 atmosphere. The reaction was quenched with water, and the mixture was extracted with EtOAc. The organic extracts were washed with brine, dried over Na_2SO_4. The solvent was removed under reduced pressure. The residue was purified by flash chromatography to give **9.**

Characterization of compounds 9

2-ethyl-2-phenyl-1-tosylpyrrolidine (9a)

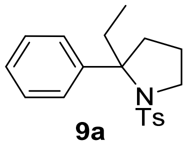

9a

According to the general procedure for the copper-catalyzed methylative cycloamination of alkenes. The crude product was purified by flash column chromatography on silica gel (PE/EtOAc = 6/1) to afford **9a** (46.7 mg, 71% yield) as a colorless oil.

^{1}H NMR (400 MHz, $CDCl_3$) δ 7.36–7.27 (m, 4H), 7.24–7.18 (m, 3H), 7.12 (d, J = 7.8 Hz, 2H), 3.69–3.63 (m, 1H), 3.57 (ddd, J = 9.4, 7.4, 5.8 Hz, 1H), 2.53–2.40 (m, 2H), 2.39 (s, 3H), 2.21–2.14 (m, 2H), 1.97–1.83 (m, 2H), 0.99 (t, J = 7.3 Hz, 3H).

^{13}C NMR (101 MHz, $CDCl_3$) δ 145.3, 142.4, 138.2, 129.1, 128.0, 127.1, 126.9, 126.8, 73.3, 50.4, 40.9, 31.3, 23.3, 21.6, 9.8.

HRMS (ESI) m/z: $[M + Na]^+$ Calcd for $C_{19}H_{23}NNaO_2S^+$ 352.1342; Found 352.1345.

IR (neat) cm^{-1} υ: 1733 (w), 1589 (w), 1474 (w), 1393 (w), 1333 (m), 1148 (s), 1086(s), 1044 (m), 1009(s), 927 (w), 807(s), 706(m).

2-ethyl-2-(p-tolyl)-1-tosylpyrrolidine (9b)

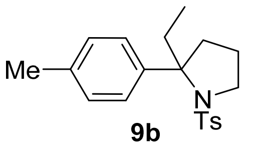

9b

According to the general procedure for the copper-catalyzed methylative cycloamination of alkenes. The crude product was purified by flash column chromatography on silica gel (PE/EtOAc = 6/1) to afford **9b** (44.9 mg, 65% yield) as a colorless oil.

^{1}H NMR (400 MHz, $CDCl_3$) δ 7.33 (d, J = 8.2 Hz, 2H), 7.20 (d, J = 8.1 Hz, 2H), 7.11 (d, J = 8.0 Hz, 2H), 7.01 (d, J = 7.9 Hz, 2H), 3.67–3.62 (m, 1H), 3.59–3.53 (m, 1H), 2.51–2.36 (m, 2H), 2.38 (s, 3H), 2.32 (s, 3H), 2.19–2.14 (m, 2H), 1.91−1.85 (m, 2H), 0.97 (t, J = 7.3 Hz, 3H).

^{13}C NMR (101 MHz, $CDCl_3$) δ 142.3, 142.2, 138.3, 136.4, 129.0, 128.6, 127.1, 126.9, 73.1, 50.4, 40.8, 31.3, 23.3, 21.6, 21.0, 9.8.

HRMS (ESI) m/z: $[M + Na]^+$ Calcd for $C_{20}H_{25}NNaO_2S^+$ 366.1498; Found 366.1500.

IR (neat) cm^{-1} υ: 1803 (w), 1434 (m), 1368 (m), 1296 (s), 1173 (s), 1126 (s), 1028 (s), 993 (s), 833 (s), 782 (s).

2-ethyl-2-(4-methoxyphenyl)-1-tosylpyrrolidine (9c)

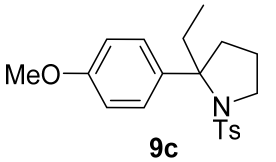

9c

According to the general procedure for the copper-catalyzed methylative cycloamination of alkenes. The crude product was purified by flash column chromatography on silica gel (PE/EtOAc = 6/1) to afford **9c** (42.4 mg, 56% yield) as a colorless oil.

^{1}H NMR (400 MHz, $CDCl_3$) δ 7.30 (d, J = 8.0 Hz, 2H), 7.22 (d, J = 8.8 Hz, 2H), 7.10 (d, J = 8.0 Hz, 2H), 6.72 (d, J = 8.8 Hz, 2H), 3.80 (s, 3H), 3.68–3.62 (m, 1H), 3.59–3.49 (m, 1H), 2.47–2.39 (m, 2H), 2.37 (s, 3H), 2.16 (t, J = 7.3 Hz, 2H), 1.95–1.86 (m, 2H), 0.98 (t, J = 7.3 Hz, 3H).

^{13}C NMR (101 MHz, $CDCl_3$) δ 158.4, 142.3, 138.3, 137.1, 129.0, 128.2, 127.1, 113.2, 72.7, 55.4, 50.4, 40.8, 31.6, 23.4, 21.6, 9.8.

HRMS (ESI) m/z: $[M + Na]^+$ Calcd for $C_{20}H_{25}NNaO_3S^+$ 382.1447; Found 382.1445.

IR (neat) cm^{-1} υ: 1610 (w), 1513 (m), 1463 (w), 1325 (m), 1253 (m), 1188 (m), 1154 (s), 1093 (s), 1029 (m), 1008 (m), 818 (m), 721 (m).

2-ethyl-2-(4-fluorophenyl)-1-tosylpyrrolidine (9d)

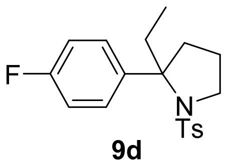

9d

According to the general procedure for the copper-catalyzed methylative cycloamination of alkenes. The crude product was purified by flash column chromatography on silica gel (PE/EtOAc = 6/1) to afford **9d** (45.8 mg, 66% yield) as a white solid.

MP: 63–64 °C

^{1}H NMR (400 MHz, $CDCl_3$) δ 7.26 (d, J = 7.9 Hz, 2H), 7.22–7.19 (m, 2H), 7.07 (d, J = 8.0 Hz, 2H), 6.80 (t, J = 8.7 Hz, 2H), 3.61–3.55 (m, 1H), 3.53–3.46 (m, 1H), 2.44–2.25 (m, 2H), 2.31 (s, 3H), 2.15–2.01 (m, 2H), 1.91–1.75 (m, 2H), 0.90 (t, J = 7.3 Hz, 3H).

^{13}C NMR (101 MHz, $CDCl_3$) δ 161.7 (d, J = 245.7 Hz), 142.6, 141.1 (d, J = 3.3 Hz), 138.1, 128.6 (d, J = 8.0 Hz), 127.0, 114.6 (d, J = 21.2 Hz), 72.7, 50.4, 40.9, 31.4, 23.2, 21.6, 9.7.

HRMS (ESI) m/z: $[M + H]^+$ Calcd for $C_{19}H_{23}FNO_2S^+$ 348.1428; Found 348.1429.

IR (neat) cm^{-1} υ: 1602 (w), 1510 (m), 1470 (w), 1331 (s), 1306 (w), 1224 (m), 1151 (s), 1093 (m), 1049 (w), 1008 (m), 873 (w), 823 (m), 807 (m), 707 (m).

2-(4-chlorophenyl)-2-ethyl-1-tosylpyrrolidine (9e)

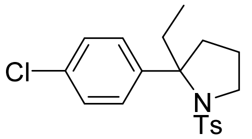

9e

According to the general procedure for the copper-catalyzed methylative cycloamination of alkenes. The crude product was purified by flash column chromatography on silica gel (PE/EtOAc = 6/1) to afford **7e** (53.2 mg, 73% yield) as a white solid.

MP: 102–103 ºC

^{1}H NMR (400 MHz, $CDCl_3$) δ 7.27 (d, J = 8.1 Hz, 2H), 7.16 (d, J = 8.8 Hz, 2H), 7.11–7.02 (m, 4H), 3.61–3.55 (m, 1H), 3.54–3.48 (m, 1H), 2.43–2.23 (m, 2H), 2.32 (s, 3H), 2.15–2.08 (m, 1H), 2.06–1.96 (m, 1H), 1.91–1.77 (m, 2H), 0.89 (t, J = 7.3 Hz, 3H).

^{13}C NMR (101 MHz, $CDCl_3$) δ 144.0, 142.7, 138.1, 132.7, 129.2, 128.3, 128.0, 127.0, 72.6, 50.5, 40.9, 31.2, 23.2, 21.6, 9.7.

HRMS (ESI) m/z: $[M + H]^+$ Calcd for $C_{19}H_{23}ClNO_2S^+$ 364.1133; Found 364.1128.

IR (neat) cm^{-1} υ: 1597 (w), 1492 (w), 1401 (w), 1330 (s), 1210 (w), 1152 (s), 1091 (s), 1046 (m), 1008 (s), 866 (w), 811 (s), 720 (m).

2-(4-bromophenyl)-2-ethyl-1-tosylpyrrolidine (9f)

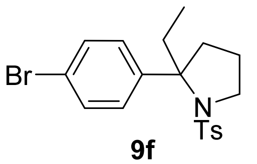

9f

According to the general procedure for the copper-catalyzed methylative cycloamination of alkenes. The crude product was purified by flash column chromatography on silica gel (PE/EtOAc = 6/1) to afford **7f** (51.6 mg, 63% yield) as a colorless oil.

^{1}H NMR (400 MHz, $CDCl_3$) δ 7.37–7.32 (m, 2H), 7.32–7.27 (m, 2H), 7.21–7.13 (m, 4H), 3.69–3.63 (m, 1H), 3.61–3.56 (m, 1H), 2.53–2.29 (m, 2H), 2.39 (s, 3H), 2.22–2.15 (m, 1H), 2.13–2.06 (m, 1H), 1.99–1.82 (m, 2H), 0.96 (t, J = 7.3 Hz, 3H).

^{13}C NMR (101 MHz, $CDCl_3$) δ 144.5, 142.7, 138.0, 131.0 129.2, 128.7, 127.0, 120.9, 72.6, 50.5, 40.9, 31.1, 23.2, 21.6, 9.7.

HRMS (ESI) m/z: $[M + H]^+$ Calcd for $C_{19}H_{23}BrNO_2S^+$ 408.0627; Found 408.0629.

IR (neat) cm^{-1} υ: 2160 (w), 1735 (w), 1597 (w), 1488 (w), 1329 (s), 1209 (w), 1151 (s), 1089 (s), 1005 (s), 865 (m), 810 (s), 709 (m).

2-([1,1'-biphenyl]-4-yl)-2-ethyl-1-tosylpyrrolidine (9g)

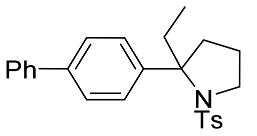

9g

According to the general procedure for the copper-catalyzed methylative cycloamination of alkenes. The crude product was purified by flash column chromatography on silica gel (PE/EtOAc = 8/1) to afford **7g** (51.6 mg, 64% yield) as a colorless oil.

^{1}H NMR (400 MHz, $CDCl_3$) δ 7.58 (d, J = 7.2 Hz, 2H), 7.48–7.41 (m, 4H), 7.39–7.31 (m, 5H), 7.09 (d, J = 8.0 Hz, 2H), 3.75–3.70 (m, 1H), 3.66–3.60 (m, 1H), 2.59–2.50 (m, 1H), 2.48–2.41 (m, 1H), 2.35 (s, 3H), 2.25–2.20 (m, 2H), 2.04–1.89 (m, 2H), 1.03 (t, J = 7.3 Hz, 3H).

^{13}C NMR (101 MHz, $CDCl_3$) δ 144.2, 142.3, 140.8, 139.6, 138.2, 129.1, 128.9, 127.42, 127.36, 127.1, 127.0, 126.6, 72.8, 50.6, 41.0, 31.3, 23.4, 21.5, 9.8.

HRMS (ESI) m/z: $[M + Na]^+$ Calcd for $C_{25}H_{27}NNaO_2S^+$ 428.1655; Found 428.1655.

IR (neat) cm^{-1} υ: 1730 (w), 1596 (w), 1486 (w), 1470 (w), 1397 (w), 1329 (m), 1151 (s), 1089 (s), 1046 (m), 1006 (s), 924 (w), 809 (s), 709 (m).

2-ethyl-2-(4-isopropylphenyl)-1-tosylpyrrolidine (9h)

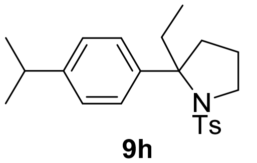

9h

According to the general procedure for the copper-catalyzed methylative cycloamination of alkenes. The crude product was purified by flash column chromatography on silica gel (PE/EtOAc = 8/1) to afford **9h** (40.3 mg, 62% yield) as a colorless oil.

^{1}H NMR (400 MHz, $CDCl_3$) δ 7.21 – 7.09 (m, 4H), 7.02–6.94 (m, 4H), 3.64–3.59 (m, 1H), 3.52–3.45 (m, 1H), 2.83–2.76 (m, 1H), 2.48–2.39 (m, 1H), 2.37–2.30 (m, 1H), 2.29 (s, 3H), 2.11 (t, J = 7.3 Hz, 2H), 1.89–1.80 (m, 2H), 1.18 (d, J = 7.0 Hz, 3H), 1.17 (d, J = 7.0 Hz, 3H), 0.93 (t, J = 7.3 Hz, 3H).

^{13}C NMR (101 MHz, $CDCl_3$) δ 147.3, 142.2, 142.1, 138.2, 129.0, 127.1, 127.0, 126.0, 72.7, 50.5, 41.0, 33.7, 31.5, 24.2, 24.1, 23.5, 21.6, 9.8.

HRMS (ESI) m/z: $[M + Na]^+$ Calcd for $C_{22}H_{29}NNaO_2S^+$ 394.1811; Found 394.1813.

IR (neat) cm^{-1} υ: 1599 (w), 1463 (w), 1335 (m), 1221 (w), 1154 (s), 1093 (s), 1053 (m), 1007 (m), 865 (w), 815 (m), 709 (w).

2-ethyl-2-(m-tolyl)-1-tosylpyrrolidine (9i)

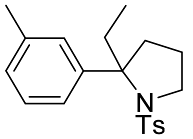

9i

According to the general procedure for the copper-catalyzed methylative cycloamination of alkenes. The crude product was purified by flash column chromatography on silica gel (PE/EtOAc = 6/1) to afford **9i** (41.8 mg, 61% yield) as a white solid.

MP: 86–88 ºC.

^{1}H NMR (400 MHz, $CDCl_3$) δ 7.30 (d, J = 8.3 Hz, 2H), 7.18–7.10 (m, 4H), 7.04–7.00 (m, 1H), 6.98 (d, J = 1.7 Hz, 1H), 3.74 (dt, J = 9.4, 7.0 Hz, 1H), 3.61–3.55 (m, 1H), 2.60–2.50 (m, 1H), 2.44–2.37 (m, 1H), 2.40 (s, 3H), 2.24–2.12 (m, 2H), 2.21 (s, 3H), 2.01–1.86 (m, 2H), 1.05 (t, J = 7.3 Hz, 3H).

^{13}C NMR (101 MHz, $CDCl_3$) δ 145.2, 142.2, 138.1, 137.4, 129.0, 127.9, 127.9, 127.6, 127.1, 124.0, 73.0, 50.6, 41.2, 31.4, 23.4, 21.7, 21.5, 9.7.

HRMS (ESI) m/z: $[M + Na]^+$ Calcd for $C_{20}H_{25}NNaO_2S^+$ 366.1498; Found 366.1499.

IR (neat) cm^{-1} υ: 1600 (w), 1468 (w), 1452 (w), 1330 (s), 1220 (w), 1153 (s), 1094 (s), 1048 (m), 1008 (m), 871 (w), 814 (m), 778 (m), 707 (s).

2-(3-chlorophenyl)-2-ethyl-1-tosylpyrrolidine (9j)

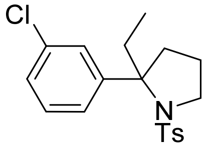

9j

According to the general procedure for the copper-catalyzed methylative cycloamination of alkenes. The crude product was purified by flash column chromatography on silica gel (PE/EtOAc = 6/1) to afford **7j** (45.6 mg, 63% yield) as a white solid.

MP: 106–108 ºC

^{1}H NMR (400 MHz, $CDCl_3$) δ 7.26 (d, J = 8.0 Hz, 2H), 7.20–7.17 (m, 1H), 7.14–7.05 (m, 4H), 7.02 (brs, 1H), 3.65–3.59 (m, 1H), 3.53–3.47 (m, 1H), 2.49–2.40 (m, 1H), 2.32 (s, 3H), 2.29–2.22 (m, 1H), 2.18–2.08 (m, 1H), 2.06–1.96 (m, 1H), 1.92–1.78 (m, 2H), 0.95 (t, J = 7.3 Hz, 3H).

^{13}**C NMR** (101 MHz, $CDCl_3$) δ 147.7, 142.8, 137.8, 134.0, 129.3, 127.3, 126.99, 126.96, 125.0, 72.7, 50.6, 41.2, 31.3, 23.3, 21.6, 9.6.

HRMS (ESI) m/z: $[M + H]^+$ Calcd for $C_{19}H_{23}ClNO_2S^+$ 364.1133; Found 364.1131.

IR (neat) cm^{-1} υ: 1595 (w), 1570 (w), 1468 (w), 1413 (w), 1329 (s), 1304 (w), 1217 (w), 1153 (s), 1092 (s), 1049 (m), 1008 (m), 1001 (w), 899 (w), 871 (w), 815 (m), 781 (m), 730 (m), 697 (m).

4-(2-ethyl-1-tosylpyrrolidin-2-yl)benzonitrile (9k)

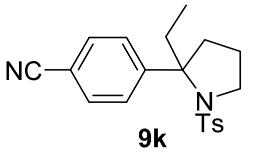

9k

According to the general procedure for the copper-catalyzed methylative cycloamination of alkenes. The crude product was purified by flash column chromatography on silica gel (PE/EtOAc = 6/1) to afford **9k** (48.0 mg, 68% yield) as a colorless oil.

^{1}**H NMR** (400 MHz, $CDCl_3$) δ 7.60 – 7.52 (m, 2H), 7.51 – 7.46 (m, 2H), 7.43 (d, J = 8.3 Hz, 2H), 7.21 (d, J = 8.0 Hz, 2H), 3.74 – 3.61 (m, 2H), 2.55–2.34 (m, 2H), 2.43 (s, 3H), 2.24 (ddd, J = 13.1, 8.5, 7.5 Hz, 1H), 2.08 (ddd, J = 13.0, 7.5, 5.3 Hz, 1H), 2.00 – 1.84 (m, 2H), 0.97 (t, J = 7.3 Hz, 3H).

^{13}**C NMR** (101 MHz, $CDCl_3$) δ 151.6, 143.1, 137.9, 131.9, 129.4, 127.5, 127.0, 118.9, 110.7, 73.1, 50.5, 40.9, 30.8, 23.2, 21.6, 9.6.

HRMS (ESI) m/z: $[M + Na]^+$ Calcd for $C_{20}H_{22}N_2NaO_2S^+$ 377.1294; Found 377.1298.

IR (neat) cm^{-1} υ: 2920 (w), 2227 (w), 1604 (w), 1505 (w), 1405 (w), 1323 (m), 1156 (s), 1093 (m), 909 (w), 848 (m), 814 (m), 755 (s), 750 (s).

Supplementary Notes

Scaled up reactions

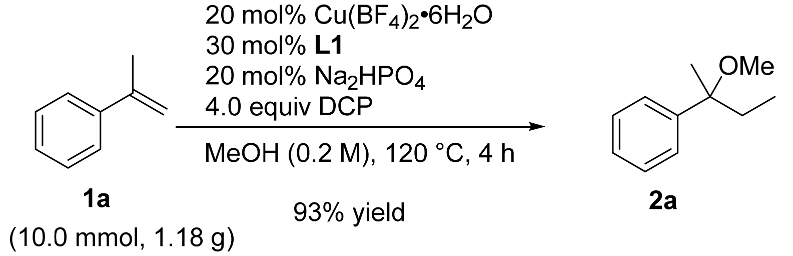

A screw cap flask was charged with $Cu(BF_4)_2$•$6H_2O$ (690.0 mg, 2.0 mmol), 4,4'-dimethoxy-2,2'-bipyridyl **L1** (650.0 mg, 3.0 mmol), Na_2HPO_4 (285.0 mg, 2.0 mmol) and MeOH (50.0 mL). The mixture was stirred at room temperature for 30 minutes, then substrate **1a** (10.0 mmol, 1.18 g, 1.0 equiv) and DCP (10.8 g, 40.0 mmol) were added to the above mixture. After being stirred for 4 hours at 120 °C under N_2 atmosphere, the reaction mixture was quenched with water, extracted with EtOAc. The organic extracts were washed with brine, dried over Na_2SO_4. The solvent was removed under reduced pressure. The residue was purified by flash chromatography to give **2a** (1.54 g, 93% yield).

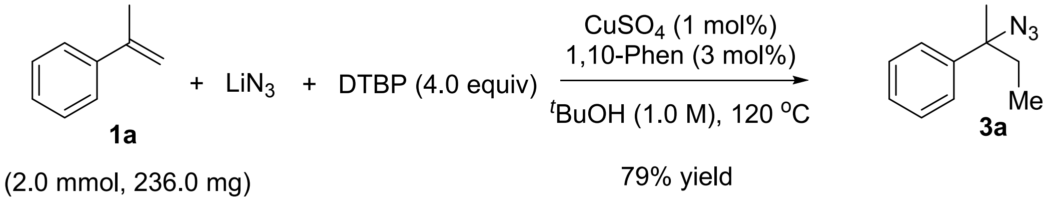

A screw cap flask was charged with $CuSO_4$ (3.2 mg, 0.02 mmol, 0.01 equiv), 1,10-Phen **L2** (10.8 mg, 0.03 mmol, 0.03 equiv) and ^{t}BuOH (20 mL). The mixture was stirred at 40 °C for 30 minutes, then cooled to room temperature. Substrate **1a** (2.0 mmol, 236.0 mg, 1.0 equiv), LiN_3 (20% w/w, 2.4 mL, 5.0 equiv) and DTBP (1.5 mL, 4.0 equiv) were added to the above mixture, and the reaction mixture was stirred at 120 °C for 8 hours under N_2 atmosphere. The reaction was quenched with water, and the mixture was extracted with EtOAc. The organic extracts were washed with brine, dried over Na_2SO_4. The solvent was removed under reduced pressure. The residue was purified by flash chromatography to give **3a** (275.6 mg, 79% yield).

Mechanism Study

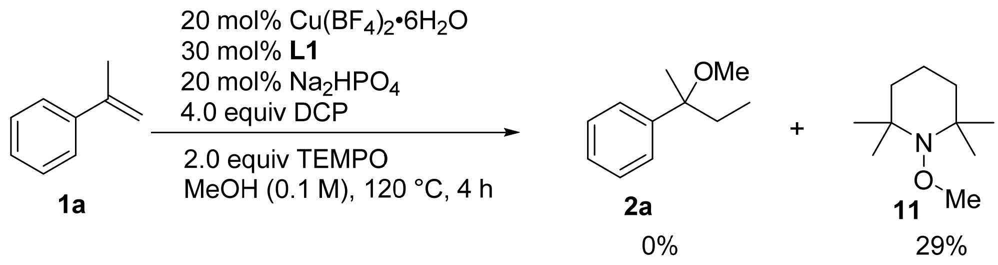

A screw cap tube was charged with $Cu(BF_4)_2$•$6H_2O$ (13.8 mg, 0.0400 mmol), 4,4'-dimethoxy-2,2'-bipyridyl **L1** (13.0 mg, 0.0601 mmol), Na_2HPO_4 (5.7 mg, 0.0402 mmol) and MeOH (2.0 mL). The mixture was stirred at room temperature for 30 minutes, then substrate **1a** (0.2 mmol, 1.0 equiv), DCP (216.2 mg, 0.800 mmol) and TEMPO (62.6 mg, 0.4 mmol, 2.0 equiv) were added to the above mixture. After being stirred for 4 hours at 120 °C under N_2 atmosphere, the reaction mixture was quenched with water, extracted with EtOAc. The organic extracts were washed with brine, dried over Na_2SO_4. The solvent was removed under reduced pressure. The residue was purified by flash chromatography to give **11** (19.4 mg, 29% yield). The physical and spectroscopic data were in accordance with those reported in the literature.[8]

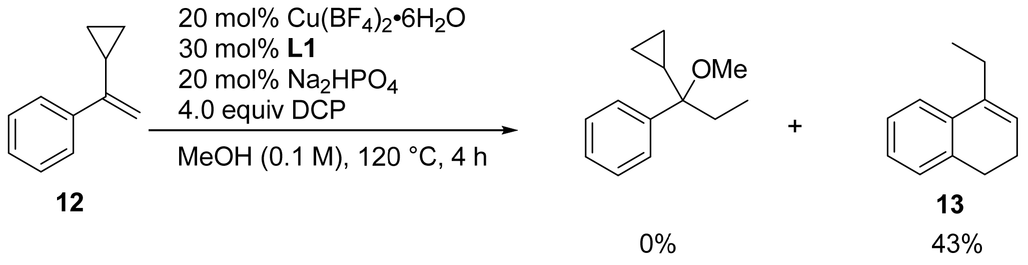

A screw cap tube was charged with $Cu(BF_4)_2$•$6H_2O$ (13.8 mg, 0.0400 mmol), 4,4'-dimethoxy-2,2'-bipyridyl **L1** (13.0 mg, 0.0601 mmol), Na_2HPO_4 (5.7 mg, 0.0402 mmol) and MeOH (2.0 mL). The mixture was stirred at room temperature for 30 minutes, then substrate **12** (0.2 mmol, 1.0 equiv) and DCP (216.2 mg, 0.800 mmol) were added to the above mixture. After being stirred for 4 hours at 120 °C under N_2 atmosphere, the reaction mixture was quenched with water, extracted with EtOAc. The organic extracts were washed with brine, dried over Na_2SO_4. The solvent was removed under reduced pressure. The residue was purified by flash chromatography to give **13** (15.4 mg, 43% yield). The physical and spectroscopic data were in accordance with those reported in the literature.[9]

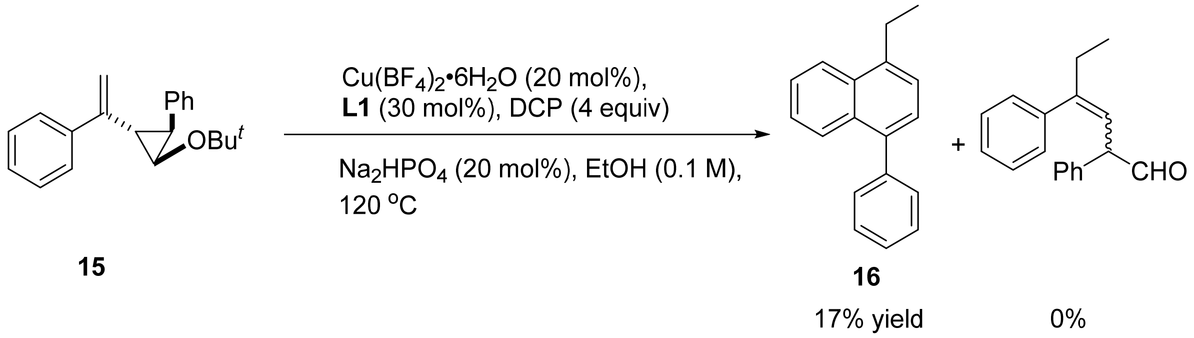

15

16

A screw cap tube was charged with $Cu(BF_4)_2 \cdot 6H_2O$ (13.8 mg, 0.0400 mmol), 4,4'-dimethoxy-2,2'-bipyridyl **L1** (13.0 mg, 0.0601 mmol), Na_2HPO_4 (5.7 mg, 0.0402 mmol) and EtOH (2.0 mL). The mixture was stirred at 40 °C for 30 minutes, then cooled to room temperature. Substrate **15**[10] (54.5mg, 0.2 mmol, 1.0 equiv) and DTBP (0.15 mL, 4.0 equiv) were added to the above mixture, and the reaction mixture was stirred at 120 °C for 4 hours under N_2 atmosphere. The reaction was quenched with water, and the mixture was extracted with EtOAc. The organic extracts were washed with brine, dried over Na_2SO_4. The solvent was removed under reduced pressure. The residue was purified by flash chromatography to give **16** (7.9 mg, 17% yield) as a white solid.

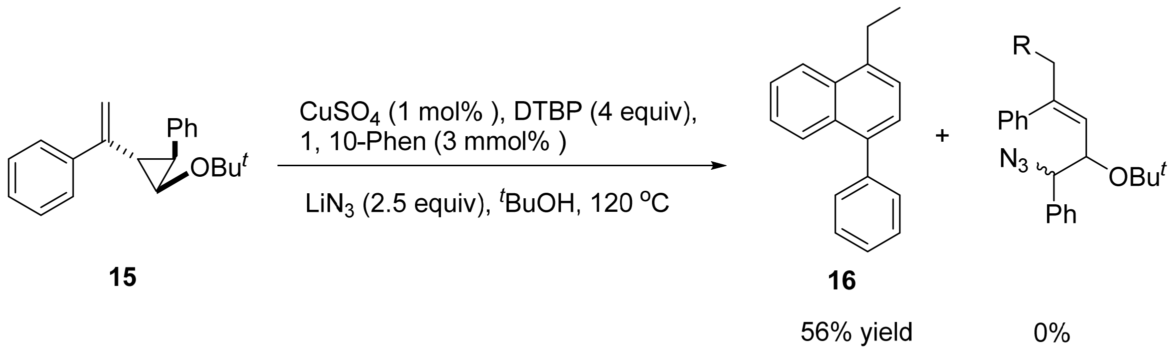

15

16

A screw cap tube was charged with $CuSO_4$ (0.32 mg, 0.002 mmol, 0.01 equiv), 1, 10-Phen (1.08 mg, 0.003 mmol, 0.03 equiv) and ^{t}BuOH (2.0 mL). The mixture was stirred at 40 °C for 30 minutes, then cooled to room temperature. Substrate **15** (54.5 mg, 0.2 mmol, 1.0 equiv), LiN_3 (20% w/w, 0.12 mL, 2.5 equiv), DTBP (0.15 mL, 4.0 equiv) were added to the above mixture, and the reaction mixture was stirred at 120 °C for 8 hours under N_2 atmosphere. The reaction was quenched with water, and the mixture was extracted with EtOAc. The organic extracts were washed with brine, dried over Na_2SO_4. The solvent was removed under reduced pressure. The residue was purified by flash chromatography to give **16** (26.2 mg, 56% yield) as a white solid.

Characterization of 1-ethyl-4-phenylnaphthalene (16)

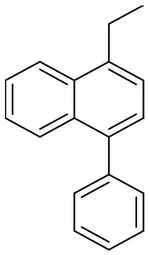

16

MP: 34–36 °C

^{1}H NMR (400 MHz, $CDCl_3$) δ 8.04 (dd, J = 8.7, 1.2 Hz, 1H), 7.84 (dd, J = 8.5, 1.2 Hz, 1H), 7.48–7.37 (m, 5H), 7.36–7.23 (m, 4H), 3.08 (q, J = 7.5 Hz, 2H), 1.35 (t, J = 7.5 Hz, 3H).

^{13}C NMR (101 MHz, $CDCl_3$) δ 141.2, 139.9, 138.7, 132.1, 132.0, 130.3, 128.3, 127.2, 127.0, 126.9, 125.7, 125.6, 124.6, 124.1, 26.2, 15.2.

HRMS (APPI) m/z: $[M]^+$ Calcd for $C_{18}H_{16}^+$ 232.1247; Found 232.1247.

IR (neat) cm^{-1} υ: 1592 (w), 1492 (w), 1459 (w), 1423 (w), 1392 (w), 1072 (w), 1033 (w), 845 (m), 764 (s), 700 (s).

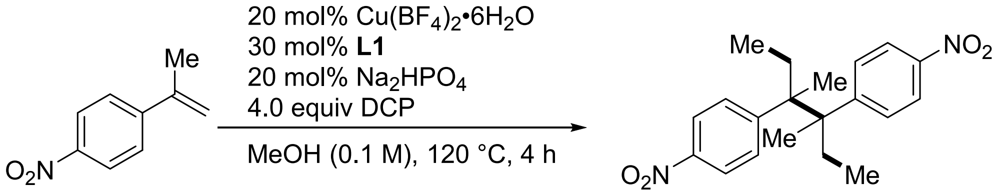

21 **22** 27%

A screw cap tube was charged with $Cu(BF_4)_2$•$6H_2O$ (13.8 mg, 0.0400 mmol), 4,4'-dimethoxy-2,2'-bipyridyl **L1** (13.0 mg, 0.0601 mmol), Na_2HPO_4 (5.7 mg, 0.0402 mmol) and MeOH (2.0 mL). The mixture was stirred at room temperature for 30 minutes, then substrate **21** (32.6 mg, 0.2 mmol, 1.0 equiv) and DCP (216.2 mg, 0.800 mmol) were added to the above mixture. After being stirred for 4 hours at 120 °C under N_2 atmosphere, the reaction mixture was quenched with water, extracted with EtOAc. The organic extracts were washed with brine, dried over Na_2SO_4. The solvent was removed under reduced pressure. The residue was purified by flash chromatography to give **22** (9.6 mg, 27% yield) as a colorless oil.

Characterization of 4,4'-(3,4-dimethylhexane-3,4-diyl)bis(nitrobenzene) (22)

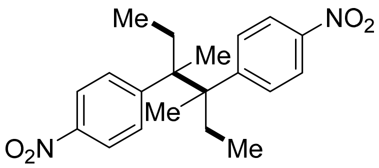

22

^{1}H NMR (400 MHz, $CDCl_3$) δ 8.10–8.02 (m, 4H), 7.16–7.02 (m, 4H), 2.27–2.89 (m, 1H), 2.09–1.98 (m, 1H), 1.72–1.57 (m, 2H), 1.34 (s, 6H), 0.53 (t, J = 7.3 Hz, 3H), 0.52 (t, J = 7.3 Hz, 3H).

^{13}C NMR (101 MHz, $CDCl_3$) δ 151.1, 151.0, 146.1, 130.5, 130.4, 122.0, 121.9, 49.2, 49.0, 27.8, 27.5, 21.3, 20.9, 9.0, 8.9.

HRMS (ESI) calcd for $C_{20}H_{25}N_2O_4^+$ $[M+H]^+$ 357.1809; found 357.1804.

IR (neat) cm^{-1} υ: 3081 (w), 2976 (w), 2934 (w), 2882 (w), 2851 (w), 1595 (w), 1512 (s), 1344 (s), 1112 (w), 1087 (w), 1013 (w), 860 (s), 707 (s).

Supplementary References

1. Alves, T. M. F.; Costa, M. O.; Bispo, B. A. D.; Pedrosa, F. L.; Ferreira, M. A. B. Cobalt-catalyzed oxidative cyclization of *gem*-disubstituted conjugated alkenols. *Tetrahedron Lett.* **2016**, *57*, 3334-3338.

2. Rösner, C.; Hennecke, U. Homohalocyclization: Electrophilic Bromine-Induced Cyclizations of Cyclopropanes. *Org. Lett.* **2015**, *17*, 3226–3229.

3. Zhou, L.; Tan, C. K.; Jiang, X.; Chen, F.; Yeung, Y.-Y. Asymmetric bromolactonization using amino-thiocarbamate catalyst. *J. Am. Chem.Soc.* **2010**, *132*, 15474–15476.

4. Ning, Z.; Jin, R.; Ding, J.; Gao, L. Enantioselective iodolactonizations of 4-pentenoic acid derivatives mediated by chiral salen-Co(II) complex. *Synlett* **2009**, 2291–2294.

5. Zhu, R.; Buchwald, S. L. Versatile Enantioselective Synthesis of Functionalized Lactones via Copper-Catalyzed Radical Oxyfunctionalization of Alkenes. *J. Am. Chem. Soc.* **2015**, *137*, 8069–8077.

6. Zhou, L.; Chen, J.; Tan, C. K.; Yeung, Y.-Y. Enantioselective bromoaminocyclization using amino-thiocarbamate catalysts. *J. Am. Chem. Soc.* **2011**, *133*, 9164–9167.

7. Dohi, T.; Takenaga, N.; Goto, A.; Maruyama, A.; Kita, Y. Direct Lactone Formation by Using Hypervalent Iodine(III) Reagents with KBr via Selective C−H Abstraction Protocol. *Org. Lett.* **2007**, *7*, 3129–3132.

8. Wang, Y.; Zhang, L.; Yang, Y.; Zhang, P.; Du, Z.; Wang, C. Alkene oxyalkylation enabled by merging rhenium catalysis with hypervalent iodine(III) reagents via decarboxylation. *J. Am. Chem. Soc.* **2013**, *135*,

9. Adamczyk, M.; Watt, D. S.; Netzel, D. A. Synthesis of biological markers in fossil fuels. 2. Synthesis and carbon-13 NMR studies of substituted indans and tetralins. *J. Org. Chem.* **1984**, *49*, 4226–4237.

10. Compound **15** was prepared according to the reported procedure: Um, C.; Chemler, S. R. Synthesis of 2-Aryl- and 2-Vinylpyrrolidines via Copper-Catalyzed Coupling of Styrenes and Dienes with Potassium β-Aminoethyl Trifluoroborates. *Org. Lett.* **2016**, *18*, 2515–2518.
